# Differential Evolution of Antiretroviral Restriction Factors in Pteropid Bats as Revealed by *APOBEC3* Gene Complexity

**DOI:** 10.1093/molbev/msy048

**Published:** 2018-03-29

**Authors:** Joshua A Hayward, Mary Tachedjian, Jie Cui, Adam Z Cheng, Adam Johnson, Michelle L Baker, Reuben S Harris, Lin-Fa Wang, Gilda Tachedjian

**Affiliations:** 1 Health Security Program, Life Sciences Discipline, Burnet Institute, Melbourne, VIC, Australia; 2 Department of Microbiology, Monash University, Clayton, VIC, Australia; 3 Australian Animal Health Laboratory, Health and Biosecurity Business Unit, CSIRO, Geelong, VIC, Australia; 4 Key Laboratory of Special Pathogens and Biosafety, Center for Emerging Infectious Diseases, Wuhan Institute of Virology, Chinese Academy of Sciences, Wuhan, China; 5 Department of Biochemistry, Molecular Biology, and Biophysics, Institute for Molecular Virology, University of Minnesota, Minneapolis, MN; 6 Howard Hughes Medical Institute, University of Minnesota, Minneapolis, MN; 7 Programme in Emerging Infectious Diseases, Duke-NUS Medical School, Singapore; 8 School of Science, College of Science, Engineering and Health, RMIT University, Melbourne, VIC, Australia; 9 Department of Microbiology and Immunology at the Doherty Institute for Infection and Immunity, The University of Melbourne, Melbourne, VIC, Australia

**Keywords:** restriction factors, bats, APOBEC3, evolution, antiviral immunity

## Abstract

Bats have attracted attention in recent years as important reservoirs of viruses deadly to humans and other mammals. These infections are typically nonpathogenic in bats raising questions about innate immune differences that might exist between bats and other mammals. The *APOBEC3* gene family encodes antiviral DNA cytosine deaminases with important roles in the suppression of diverse viruses and genomic parasites. Here, we characterize pteropid *APOBEC3* genes and show that species within the genus *Pteropus* possess the largest and most diverse array of *APOBEC3* genes identified in any mammal reported to date. Several bat APOBEC3 proteins are antiviral as demonstrated by restriction of retroviral infectivity using HIV-1 as a model, and recombinant A3Z1 subtypes possess strong DNA deaminase activity. These genes represent the first group of antiviral restriction factors identified in bats with extensive diversification relative to homologues in other mammals.

## Introduction

Bats are reservoirs of emerging viruses that are highly pathogenic to other mammals including humans. In recent years, viruses such as Ebola, Marburg, Hendra, Nipah, Sosuga, and severe acute respiratory syndrome coronavirus (SARS-CoV) have crossed species barriers from their natural hosts into humans (Calisher et al. 2006; Smith and Wang 2013; Amman et al. 2015; Hayman 2016). Intriguingly, experimental infections have demonstrated that specific bat species can be infected with Ebola, Marburg, SARS-CoV, Hendra, and Nipah viruses without the development of clinical symptoms (Swanepoel et al. 1996; Williamson et al. 1998; Middleton et al. 2007; Watanabe et al. 2010; Jones et al. 2015). This has led to efforts to uncover differences that might exist between the antiviral strategies of bats and other mammals (Baker et al. 2013; Zhang et al. 2013; Zhou et al. 2016).

Based upon studies of endogenous retroviruses, bats are an important source of mammalian retroviruses and many cross-species transmissions of retroviruses from bats to other mammals have been documented (Hayward et al. 2013; Cui et al. 2015). A number of antiviral restriction factors are known to be capable of inhibiting the normal replication of retroviruses; among the most well studied of these are the members of the APOBEC3 (A3) protein family. A3 proteins are capable of restricting the replication of several virus families including retroviruses, hepadnaviruses, and parvoviruses, in addition to retrotransposons which are genomic parasites (Chen et al. 2006; Muckenfuss et al. 2006; Renard et al. 2010). A3 proteins are DNA cytosine deaminases capable of hypermutating viral genomes and sterically hindering nascent DNA synthesis (Sheehy et al. 2002; Lecossier et al. 2003; Refsland and Harris 2013). All A3 proteins contain one or two zinc-coordinating cytosine deaminase domains (Z-domains) which are required for deaminase activity (Chiu and Greene 2008). A3 proteins are divided into three phylogenetic subgroups, Z1, Z2, and Z3, which are distinguished by the conservation of amino acid residues within the Z-domain (LaRue et al. 2009; Refsland and Harris 2013). We hypothesized that, given the prolonged history of coexistence between bats and their retroviruses, differences exist relative to other mammals in the antiviral genes responsible for retroviral restriction.

Pteropid bats are species within the family Pteropodidae, and are implicated as reservoirs of emerging human viral pathogens (Hayman 2016). Here, we report assemblies of the *A3* loci in the closely related pteropids, *Pteropus vampyrus* and *Pteropus alecto*, which harbor Nipah and Hendra viruses, respectively (Halpin et al. 2000; Rahman et al. 2010). Several bat genomes (Fang et al. 2015) have been published in recent years; however, no available assembly has resolved the A3 gene locus. This failure is likely due to the presence of many repetitive sequences, generated by extensive gene duplication and recombination, which are known to hinder genome assembly (Zhang et al. 2013; Fang et al. 2015).

In this study, pteropid *A3* loci were generated through the step-wise extension of cDNA-mapped gene scaffolds followed by remapping of sequence read archives, supported experimentally by an *A3* expression analysis of bat spleen tissue. The functionality of *P. alecto* A3 subtypes were investigated through analysis of their ability to deaminate DNA, and restrict the infectivity of a model retrovirus. The role of A3-mediated restriction of ancient bat retroviruses was assessed using a hypermutation analysis of endogenous retroviruses within the genome of *P. vampyrus*.

## Results

### Pteropid Bats Express a Diverse Range of *APOBEC3* Gene Products

Pteropid *A3* gene expression was initially determined by searching for *A3* homologues in the *P. alecto* transcriptome database through a tBLASTn analysis using the human A3 proteins A3A, A3C, and A3H, representing the three zinc-coordinating cytosine deaminase motif (Z-domain) subtypes A3Z1, A3Z2, and A3Z3, as search queries. A3 expression was subsequently assessed through analysis of cDNA (complementary DNA) generated from *P. alecto* spleen tissue, which harbors multiple immune cell types known to express *A3* genes in humans (Refsland et al. 2010). This analysis revealed 20 distinct *A3* mRNA transcripts derived from seven *A3Z1* genes, five *A3Z2* genes, and a single *A3Z3* gene (supplementary table S1, Supplementary Material online). Pairwise comparison of these A3 transcripts (supplementary fig. S1, Supplementary Material online) reveal a wide range of sequence identities between pteropid A3 genes, indicating considerable diversity among homologs: *A3Z1* genes range from 86% to 97% identity, whereas *A3Z2* genes range from 54% to 99% identity.

**Fig. 1.**
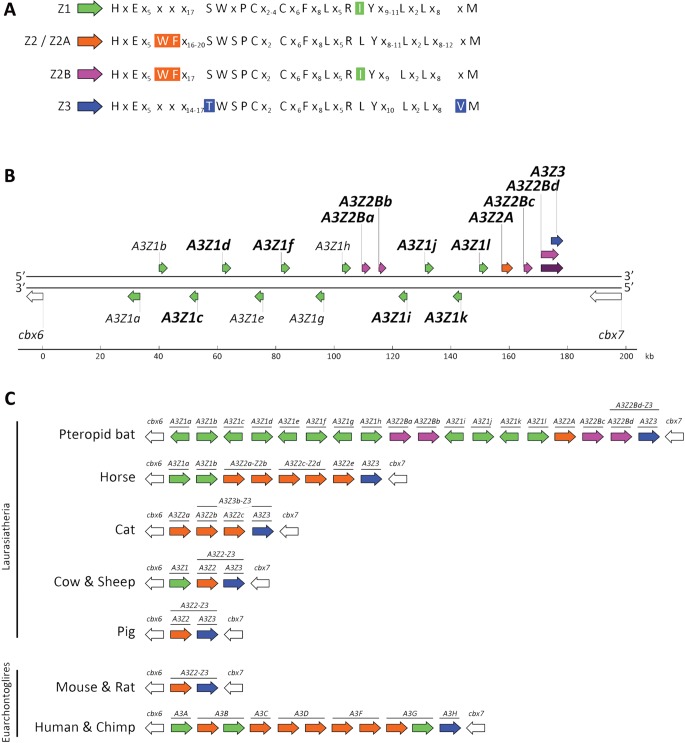
The pteropid bat A3 gene locus reveals the largest and most diverse known repertoire of *A3* genes in mammals. (*A*) The amino acid sequences of the four Z-domain subtypes are depicted from the first to the last invariant amino acid residue. Invariant amino acid residues are represented by their letter, “x” indicates positions of variable amino acid identity, and numbers indicate the length of the variable amino acid sequence. For each subtype, the defining residue(s) are colored. (*B*) The A3 gene locus of *Pteropus alecto* is shown to scale. *A3Z1*, *A3Z2A*, *A3Z2B*, and *A3Z3* gene coding regions are shown in green, orange, pink, and blue, respectively. The region encoding the *A3Z2Bd-Z3* double-domain gene product is shown in maroon. *A3* genes verified as being transcriptionally active are denoted in bold type. The *CBX6* and *CBX7* genes immediately upstream and downstream of the locus are shown in white. (*C*) Graphical schematic diagrams of sequenced mammalian A3 loci are depicted bound by the *CBX6* and *CBX7* genes, shown in white, positioned immediately upstream and downstream of the *A3* genes.

In addition to the expected A3Z1, A3Z2, and A3Z3 subtypes, several A3 amino acid sequences contained Z-domains distinct from the canonical domains as defined by a specific combination of key conserved residues (LaRue et al. 2009). This group, designated A3Z2B, carried both the A3Z2 domain-specific tryptophan-phenylalanine (WF) residues and the A3Z1 domain-specific arginine-adjacent isoleucine (RI) motif (fig. 1*A*). Of the 20 unique transcripts, 19 harbored a single Z-domain and one contained two Z-domains, an N-terminal A3Z2B domain paired with a C-terminal A3Z3 domain. These results indicate that pteropid bats express a more diverse repertoire of *A3* mRNA than reported to date for other mammals (fig. 1*C*).

### Generation of the Pteropid *APOBEC3* Gene Locus

The generation of the pteropid *A3* loci was accomplished in a step-wise manner outlined in supplementary fig. S2, Supplementary Material online. The *A3* gene locus in Eutherian mammals is located between the *CBX6* and *CBX7* genes (LaRue et al. 2008). BLASTn searches using the *P. alecto A3* cDNA products as queries revealed that publicly accessible bat genomes of *P. alecto* and *P. vampyrus* did not contain assembled *A3* gene loci located within the boundaries of *CBX6* and *CBX7*. However, the bat *A3* cDNA could be mapped, albeit across numerous discontiguous scaffolds, to the *P. vampyrus* genome accessible through the Ensembl database. The A3 locus contains many large, repetitive sequence elements that result in the failure of automated assembly. This problem was overcome by matching exons derived from sequenced cDNA, allowing the informed pairing of discontiguous scaffolds. This was not possible using the *P. alecto* genome as it did not contain sufficient assembled scaffolds to which *A3* cDNA products could be mapped. It was reasonable to map *P. alecto* cDNA products against the *P. vampyrus* genome as these species are very closely related, with a recent divergence of ∼4.4 Ma and 97–99% genomic nucleotide sequence identity (Almeida et al. 2014). The *P. vampyrus* A3 locus was then generated through a step-wise extension of preexisting *P. vampyrus* gene scaffolds to join the gaps between exon-paired scaffolds, followed by remapping of the sequence read archives (SRA) to validate and generate the 190 kB *P. vampyrus* A3 locus. The *P. alecto* A3 locus was generated by mapping *P. alecto* reads against the *P. vampyrus* A3 locus and was found to be slightly shorter at approximately 188 kB as a result of multiple short 3–20 nt insertions and deletions. These indels occurred in the *A3* loci since the divergence of *P. alecto* and *P. vampyrus*, likely as a result of nonhomologous recombination events, the frequency of which is known to be increased in regions of the genome that contain sections of repetitive DNA (Metzenberg et al. 1991).

Within the *P. alecto A3* locus 18 gene coding regions were identified including 12 *A3Z1*, one prototypical *A3Z2* (herein described as *A3Z2A*), four *A3Z2B*, and one *A3Z3* (fig. 1*B*). The organization and number of the *A3* genes was found to be identical between *P. vampyrus* and *P. alecto*. Neural network promoter prediction revealed promoter elements immediately upstream of each gene. Interferon-stimulated response element (ISRE) transcription factor binding sites for interferon regulatory factors and NF-κB were also identified in each promoter region (supplementary data S1, Supplementary Material online). These data indicate that each pteropid *A3* gene is most likely capable of being transcribed and that their expression is potentially upregulated as part of the antiviral interferon response.

All *P. alecto A3* cDNA sequences were successfully mapped against the assembled *P. alecto* loci, revealing various alternative splicing schemes that allow many of the A3 genes to express numerous transcripts (supplementary fig. S3 and table S1, Supplementary Material online). A comparison of the *A3* loci of *P. vampyrus* and *P. alecto* against the *A3* loci of other mammals (fig. 1*C*) reveals that pteropid bats possess the largest *A3* locus yet reported, containing 18 putative *A3* coding domains, 13 of which have been confirmed in this study as undergoing active transcription (fig. 1*B*).

### Bats Possess a Novel Subtype of APOBEC3

**Fig. 2.**
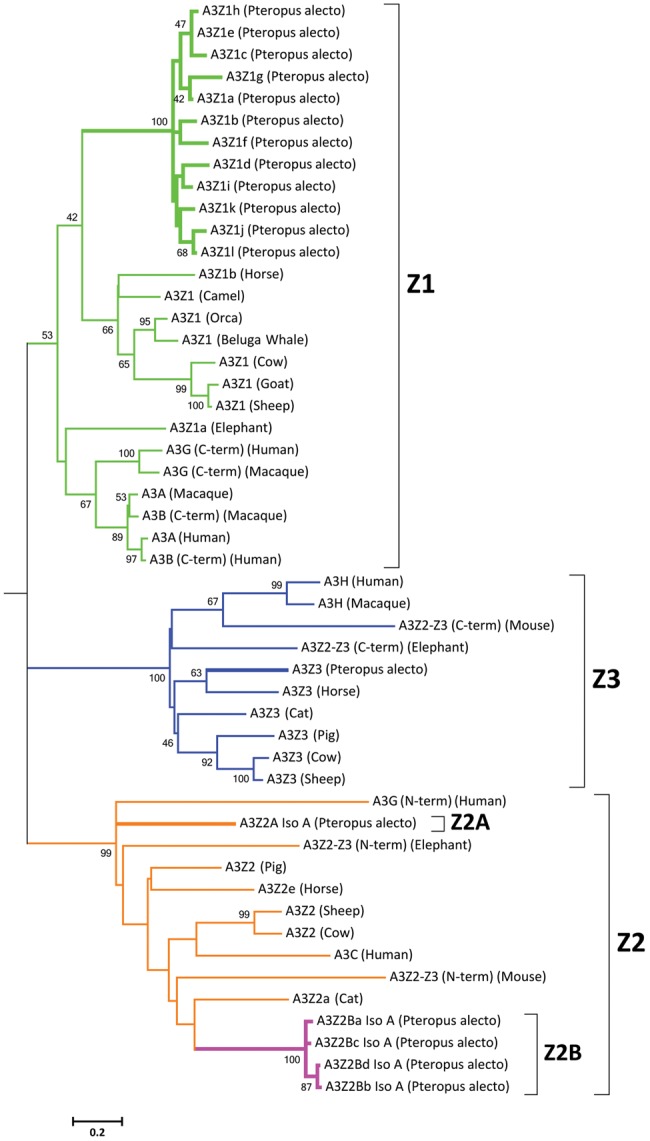
Mammalian APOBEC3 phylogeny. The phylogenetic relationships of mammalian A3 proteins were inferred using the maximum likelihood method and JTT + G model with 1,000 bootstrap replicates. A3 subgroups Z1, Z2, and Z3 are paralogs, which have expanded independently. Bootstrap values for nodes represented in >40% of trees are shown. A3Z1, A3Z2/Z2A, A3Z2B, and A3Z3 clades are shown in green, orange, pink, and blue, respectively. The scale represents the number of amino acid substitutions per site.

Analysis of the phylogenetic relationships between the A3 proteins of *P. alecto* and other mammals (fig. 2) confirms the expected phylogenetic positions of the A3Z1, A3Z2A, and A3Z3 proteins, and reveals that the A3Z2B proteins, containing both Z1- and Z2-defining sequence motifs, phylogenetically cluster within the broader A3Z2 clade. Notably, pteropid A3Z2A and A3Z2B are positioned a greater sequence distance from each other than from the A3Z2 proteins of distantly related mammals, revealing that pteropid bats possess four phylogenetically distinct A3 Z-domain subtypes, two of which appear to be derivatives of the Z2 clade.

### Bat APOBEC3 Proteins Are Potent DNA Mutators

**Fig. 3.**
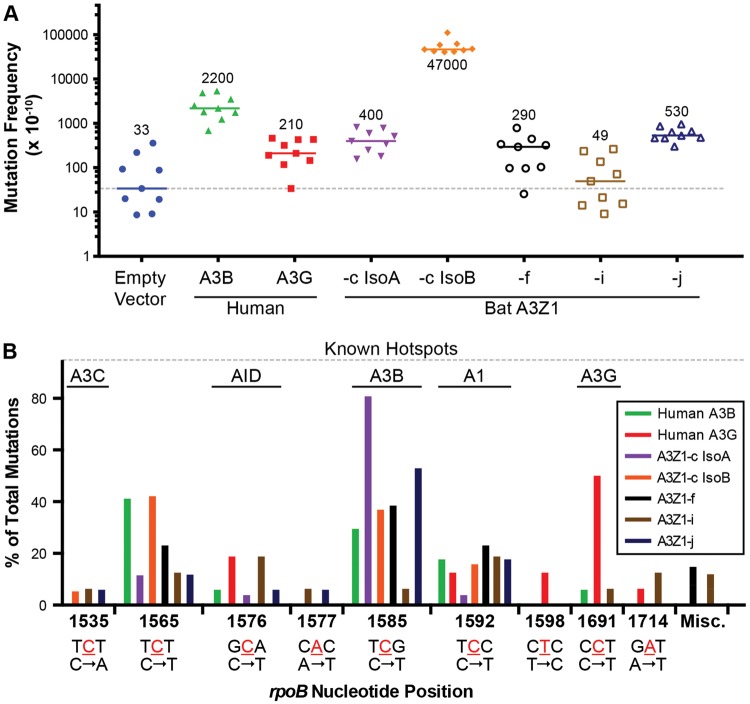
Functional analysis of bat A3 proteins. (*A*) A3 proteins were transformed in C43 (DE3) *E. coli* to measure mutational frequency in the *rpoB* gene. Each point represents an independent sample and bars represent median mutation frequency with numerical values above. (*B*) Mutation signatures in a portion of the *rpoB* gene by PCR amplification and Sanger sequencing. Position of the mutated nucleotide is underlined and in red with nucleotide changes below. Known hotspots targeted by related proteins are provided above. A1, APOBEC1; A3, APOBEC3; AID, Activation-induced deaminase.

We tested the activity of the bat A3 proteins for which expression was verified (supplementary table S1, Supplementary Material online) by assessing their ability to cause mutations in *E. coli* though a rifampicin mutagenesis assay, utilizing human A3B and A3G as positive controls because they are known to be expressed and enzymatically active in *E. coli* (Holden et al. 2008; Shi et al. 2015). Mutations in the *rpoB* gene increase with mutagenic stress mediated by A3 (Harris et al. 2002; Shi et al. 2015). Mutational frequencies above background levels were only observed for A3Z1 proteins, and we found that four bat A3Z1s showed a 10-fold or greater increase in mutation frequency (fig. 3*A*). In particular, A3Z1c isomer B showed over 1000-fold increase above empty vector and 100-fold above A3Z1c isomer A. To determine if the difference in mutation frequency correlated with differences in mutation signatures, we sequenced a PCR-amplified portion of the *rpoB* gene known to harbor hotspots for rifampicin resistance. Bat A3 mutation signatures were compared against human A3B and A3G (fig. 3*B*), which have dinucleotide sequence preferences for 5′-TC and 5′-CC, respectively. We found that bat A3Z1c isomer A was highly specific for causing mutation at a known 5′-TCG hotspot at nucleotide position 1585, which is also the preferred target site for A3B (Shi et al. 2015). In contrast, A3Z1c isomer B targeted sites at position 1565, 1585, and 1592, which have 5′-TCT, 5′-TCG, and 5′-TCC contexts, respectively. Decreased specificity may account for the high mutation frequency observed in the rifampicin mutagenesis assays. These results show that bat A3 proteins are functionally active and can mutate cytosines in a diverse range of dinucleotide contexts.

### Bat APOBEC3 Proteins Restrict HIV-1 Infectivity

**Fig. 4.**
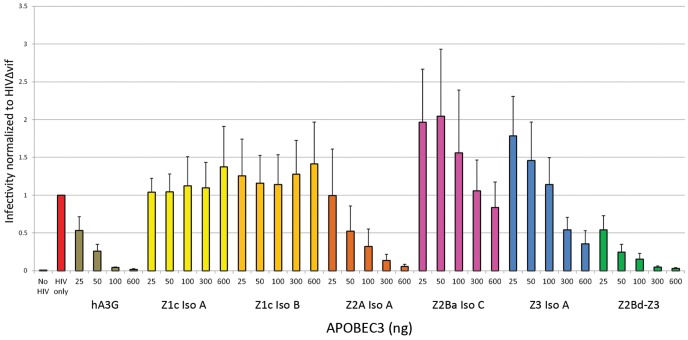
Antiviral activity of A3 proteins. Vif-deficient HIV-1 was generated in the presence of A3 proteins. Human 293 T cells were transfected with 400 ng of the HIVΔvif expression plasmid pIIIBΔvif and variable amounts (25–600 ng) of human or bat A3 expression plasmids. TZM-bl indicator cells were inoculated with 293 T cell culture supernatants normalized for viral particles by virion-associated RT activity. Infectivity relative to HIVΔvif alone was measured by quantifying luciferase activity in TZM-bl lysates. HIV, human immunodeficiency virus type 1; hA3G, human APOBEC3G; Z1c Iso A, *Pteropus alecto* APOBEC3Z1c isomer A; Z1c Iso B, *Pteropus alecto* APOBEC3Z1c isomer B; Z2A Iso A, *Pteropus alecto* APOBEC3Z2A isomer A; Z2Ba Iso C, *P. alecto* APOBEC3Z2Ba isomer C; Z3 Iso A, *P. alecto* APOBEC3Z3 isomer A; Z2Bd-Z3, *P. alecto* APOBEC3Z2Bd-Z3. Error bars represent the standard error of the mean from three independent assays.

The antiviral activity of bat A3 proteins was determined by assessing the capacity of representatives of each bat A3 subtype to restrict HIV-1 infectivity in target cells. HIV-1 encodes the anti-A3 counter-measure, Vif, a protein that causes the proteolytic degradation of A3 through the recruitment of the cellular E3 ubiquitin ligase (Marin et al. 2003; Sheehy et al. 2003; Kane et al. 2015). HIV-1 stocks were generated by cotransfection of 293 T producer cells with bat A3 and an HIV-1 variant that does not express Vif. HIV-1 particles were harvested from the cell culture supernatant, and virion production was determined by quantifying virion-associated reverse transcriptase (RT) activity. HIV-1 infectivity was assessed by inoculating human TZM-bl target cells, which express luciferase in the presence of HIV-1 infection, with virus normalized to equivalent levels of virion-associated RT activity. Four bat A3 proteins demonstrated a dose-dependent inhibition of HIV-1 infectivity (fig. 4). The sole double Z-domain bat A3 protein, A3Z2Bd-Z3, produced the largest reduction in HIV-1 infectivity, which was comparable to inhibition by the human A3 protein, A3G. These data indicate that bat A3 proteins are functional restriction factors.

### Ancient Bat Retroviruses Were Hypermutated by APOBEC3

**Fig. 5.**
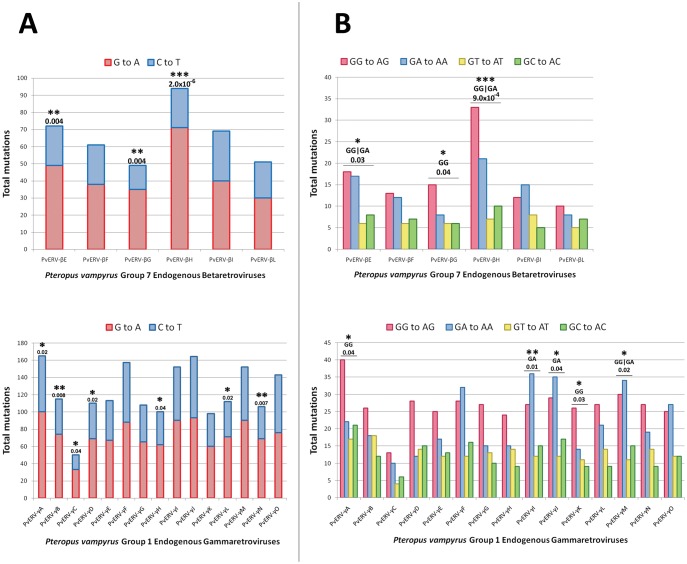
Hypermutation analysis of endogenized retroviruses reveals that ancient bat retroviruses were subjected to restriction by A3 proteins. (*A*) Comparison of guanine to adenine (G to A) versus cytosine to thymidine (C to T) mutations on the coding (+) strand of endogenous retroviruses reveals strand-biased cytosine deamination. (*B*) Subsequent analysis of the dinucleotide context of the G to A mutations in the coding strand reveals that the GG to AG and GA to AA mutations typical of human A3G are the most common mutations in the endogenous retroviruses of bats. Statistical significance was calculated using Fisher’s Exact Test. *P < 0.05; **P < 0.01; ***P < 0.001.

A3 activity results in C to U lesions in the negative (cDNA) strand, which lead to positive (genomic) strand G to A mutations in endogenous retroviruses (ERVs) within host genomes (Perez-Caballero et al. 2008). A3 proteins also exhibit local sequence-context bias in the nucleobases they deaminate. The presence of strand-biased, context-specific A3 mutagenic signatures in ERVs constitutes unambiguous evidence of a past encounter with one or more cellular A3 enzymes (Lee et al. 2008). To assess hypermutation of pteropid ERVs, we identified a group of closely related ERVs from each of the *Betaretrovirus* and *Gammaretrovirus* genera present in the genome of *P. vampyrus*. We interrogated the *P. vampyrus* genome since the *P. alecto* genome assembly does not contain sufficient contiguous endogenous retroviral sequences to conduct this analysis. A previously reported group of bat endogenous betaretroviruses, sub-classified as Group 7 betaretroviruses, was selected and a group of bat endogenous gammaretroviruses (herein described as Group 1 gammaretroviruses) was identified in the manner described previously (Hayward et al. 2013). Phylogenies were generated using the maximum likelihood method and an ancestral reconstruction was performed by extrapolation of the common ancestor of each group (see supplementary fig. S4, Supplementary Material online). Mutations in each ERV were identified by comparison against the group’s common ancestor. Strand-biased G to A hypermutation and context-specific GG and GA dinucleotide preferences were identified in both groups (fig. 5). These results are consistent with the hypothesis that ancient bat retroviruses were subjected to A3-mediated hypermutation, supporting the expected role of bat A3 proteins as antiviral restriction factors. The divergence times of the bat *A3Z1* genes were estimated using a molecular clock dating analysis that indicates these genes arose through duplication events beginning approximately 27 Ma (supplementary fig. S5, Supplementary Material online).

## Discussion

This study was undertaken to address the question of differential antiviral gene evolution in bats, and more specifically, whether important differences exist in a major class of genes that control and restrict retroviruses and genomic retroelements. We demonstrated that bats possess 18 *A3* genes, 13 of which are transcriptionally active in spleen tissue, with several proving active in a heterologous *E. coli* base mutation assay or an HIV-1 restriction assay. In addition to the expected A3Z1, A3Z2, and A3Z3 phylogenetic subtypes, we identified an additional division within the bat A3Z2 clade in which we designated A3Z2A as the prototypical A3Z2 subtype, and A3Z2B, as a subdivision that possesses a combination of invariant amino residues key to the amino acid character of both the A3Z1 and A3Z2 subtypes. Representative A3 loci have previously been delineated for every Eutherian order with the exceptions of Chiroptera (bats) and all orders within the clade Afrotheria (e.g., elephants, tenrecs, dugongs, and hyraxes) (Nakano et al. 2017). With the caveat that the A3 loci of the vast majority of mammalian species remain to be resolved, the pteropid bat species studied here are shown to possess the largest and most phylogenetically diverse antiviral A3 gene repertoire of any mammal reported to date.

Assessment of the functionality of bat A3 proteins through a rifampicin mutagenesis assay revealed several A3Z1 proteins as potent mediators of cytosine deamination with A3Z1 isomer B demonstrating a mutational frequency greater than human A3B and A3G. Although some A3Z1 proteins, and A3 proteins of other subtypes were not found to be catalytically active in this assay, numerous factors could account for inactivity within *E. coli*, such as protein misfolding or missing cellular cofactors and localization requirements. The capacity of bat A3 proteins to restrict retroviral infection was observed through the use of HIV-1 as a model target for A3 functionality. Four bat A3 proteins representing single and double Z-domain proteins of the Z2A, Z2B, and Z3 subtypes were observed to be capable of inhibiting HIV-1 infectivity in a dose-dependent manner. The bat A3Z1 proteins found to be mutagenic were not found to be capable of restricting HIV-1 infectivity. A possible explanation for this is that not all bat A3 proteins are capable of restricting this specific virus, as is the case for human A3 proteins such as A3B and A3C which inhibit SIV but not HIV, whereas A3F and A3G are potent inhibitors of HIV (Sheehy et al. 2003; Yu et al. 2004; Zheng et al. 2004). Although many endogenous retroviruses are present in bat genomes (Cui et al. 2012; Hayward et al. 2013), no exogenous bat retroviruses have yet been reported. To address our expectation that bat retroviruses could be subjected to restriction by bat A3 proteins we performed a hypermutation analysis of the endogenized retroviruses present in the genome of *P. vampyrus* and found evidence of strand-biased, context-dependent, cytosine deamination of both beta- and gammaretroviruses. These results implicate bat A3 proteins as the mediators of G to A hypermutation in bat retroviruses.

The large number of *A3Z1* genes present in the pteropid *A3* locus is surprising given the scarcity of this subtype among other mammals (LaRue et al. 2009). The presence of 12 *A3Z1* genes in pteropid bats indicates an evolutionary advantage for pteropid bats in the maintenance and expansion of this subtype. The most comparable APOBEC3 protein in humans is A3A, the sole APOBEC3 single-domain Z1 protein. A3B and A3G are double-domain proteins that have a Z1 paired with a Z2A domain (fig. 1*C*). Endogenous A3A is cytoplasmically located (Land et al. 2013) but accesses the nucleus during telophase, the final stage of mitosis (Lackey et al. 2013). It has restrictive activity against retrotransposons (Chen et al. 2006; Koito and Ikeda 2013) and viruses including HIV-1, adeno-associated virus, and human papillomavirus (Chen et al. 2006; Berger et al. 2011; Warren et al. 2015). In humans, evidence suggests that A3Z1 domain-containing proteins may represent a double-edged sword, as potent inhibitors of genomic retrotransposons and a possible threat to the cell’s own genomic sequence integrity (LaRue et al. 2008; Roberts et al. 2013; Taylor et al. 2013; Burns et al. 2015; Green et al. 2016). One possible reason for the observed expansion of *A3Z1* genes in bats may be related to the evolution of flight, which is hypothesized to be an important link to the physiological conditions allowing bats to be sources of high viral diversity (O’Shea et al. 2014). Increased metabolic capacity has been linked to the evolution of sustained flight in bats (Shen et al. 2010), and the by-products of oxidative metabolism include reactive oxygen species that are known to damage cellular DNA (Cooke et al. 2003). The most notable genetic adaptations involved in the development of flight in bats have been found to exist in the genes responsible for the detection and repair of cellular DNA damage (Zhang et al. 2013; Zhou et al. 2016). It is possible that enhanced tolerance to DNA damage would allow for the expansion of genes such as *A3* whose products are known to cause such damage.

Many studies have indicated that A3 enzymes restrict endogenous transposable elements such as LINE-1 (long interspersed nuclear element 1) and Alu (Muckenfuss et al. 2006; Stenglein and Harris 2006; Kinomoto et al. 2007; Wissing et al. 2011). Interestingly, LINE-1 elements became extinct in the pteropid bat lineage approximately 24 Ma (Cantrell et al. 2008). Preliminary dating of the A3 gene expansions (supplementary fig. S5, Supplementary Material online) indicate that timing of the initial expansion of the bat *A3Z1* subtype may coincide with this event and further investigation of a possible connection between pteropid A3 genes and the LINE-1 extinction may be warranted.

This study represents the first report of the differential evolution of an important antiviral gene family in bats relative to other mammals. The full account and explanation of the ability of bats to act as such effective hosts and transmitters of zoonotic viral pathogens will likely consist of many complex and interlinking factors beyond simple metrics such as gene numbers and amino acid sequence characteristics. Ongoing studies of this expanded gene family should further demystify the strategies and mechanisms utilized by bats that result in an impressive tolerance for viral infections.

## Materials and Methods

### *APOBEC3* and Retroviral DNA Sequences

The accession numbers and genome locations of the mammalian APOBEC3 and retroviral genome sequences utilized in this study are listed in supplementary table S2, Supplementary Material online.

### Generation of Bat Transcriptomes

Approval for the use of bat tissues was granted by the Commonwealth Scientific and Industrial Research Organization’s Australian Animal Health Laboratory Animal Ethics Committee (Protocol AEC1281). Transcriptome data sets of *P. alecto* were generated from pooled total RNA from mitogen-stimulated spleen, lymph node, white blood cells, and unstimulated bone-marrow and thymus from one pregnant female and one adult male, and the unstimulated thymus of a juvenile male as described previously (Papenfuss et al. 2012). The transcriptome of *P. alecto* is available at the NCBI Sequence Read Archive (SRA) (http://www.ncbi.nlm.nih.gov/Traces/sra/; last accessed April 3, 2018) [SRA: SRP008674].

### Transcriptome Analysis

To search for predictions of *A3* gene expression within the bat transcriptomes, sequences of human *A3* transcripts were obtained from Ensembl (http://www.ensembl.org/; last accessed April 3, 2018), specifically: *A3A* [Ensembl: ENST00000402255], *A3C* [Ensembl: ENST00000361441], and *A3H* [Ensembl: ENST00000348946]. The human *A3* transcripts were translated into protein sequences using the CLC Genomics Workbench 8.0 (https://www.qiagenbioinformatics.com/; last accessed April 3, 2018). To identify transcripts of interest the tBLASTn function of CLC Genomics Workbench was used with the following parameters: BLOSUM62 matrix, word size = 3, *E*-values < 1 × 10^−12^, gap costs of existence 11, extension 1, with no filtering of regions of low complexity. Transcripts were confirmed as A3 homologues by initial identification of, in their translated protein sequences, the presence of the conserved Z-domain, Hx_1_Ex_21__–__27_S/TWSPCx_2__–__4_C (where “x” indicates a nonconserved position [Conticello et al. 2005; LaRue et al. 2009]), and subsequent identification of the A3 subtype-specific conserved motifs described in detail in LaRue et al. (2008, 2009).

### cDNA Analysis

To confirm *A3* gene product expression, polymerase chain reaction (PCR) amplification assays were performed using cDNA generated from *P. alecto* spleen tissue and various combinations of forward and reverse primers designed using the *A3* transcript predictions identified in the transcriptome analysis. *P. alecto* trapping, tissue collection and RNA extraction was performed as reported previously (Cowled et al. 2011) except RNAlater (Ambion) preserved spleen from four male adult bats was pooled prior to tissue homogenization followed by total RNA extraction with the Qiagen RNeasy Mini kit with on-column removal of genomic DNA with DNase I. Total RNA was reverse transcribed into cDNA with the Qiagen Omniscript reverse transcriptase according to the manufacturer’s protocol except that the reaction contained 100 ng/μl total RNA, 1 μM oligo(dT)_18_ (Qiagen), and 10 μM random hexamers (Promega).

All PCR amplification assays were performed using the Roche FastStart High Fidelity PCR system (Cat # 04738292001) utilizing an annealing temperature gradient of 54–64°C in 2°C increments. Each reaction was made up to a total of 20 μl, containing 1 unit of polymerase, 2 ng of total cDNA, and 8 pmol of each primer. All other parameters for PCR amplification were performed according to the manufacturer’s recommended protocol. PCR reaction products were separated by agarose gel electrophoresis, using a 1% (w/v) agarose gel run at 120 V for 25 min. All DNA bands were excised from the gel and purified using the Qiagen QIAquick Gel Extraction kit (Cat # 28706) according to the manufacturer’s protocol. The gel extracted PCR products were cloned into the pCR2.1 plasmid vector using the Invitrogen TOPO TA Cloning kit (Cat # 450641) according to the manufacturer’s protocol and transformed into *Escherichia coli* TOP10 (Invitrogen). The nucleotide sequence of clones were determined by Sanger sequencing using the M13F (5′-GTAAAACGACGGCCAG-3′) and M13R (5′-CAGGAAACAGCTATGAC-3′) sequencing primers.

### APOBEC3 Gene Locus Assembly

To identify the gene scaffolds representing fragments of the *A3* gene locus in the *P. vampyrus* genome, a BLASTn analysis was performed using the *A3* gene products amplified in the cDNA analysis as queries. The *P. vampyrus* genome was downloaded from Ensembl and all BLASTn searches were performed locally using CLC Genomics Workbench with the following parameters: Match score 2, mismatch cost −3, word size = 5, *E*-values < 1 × 10^−5^, gap costs of existence 5, extension 2, with no filtering of regions of low complexity. To extend the sequence of each scaffold, 150 nt at both the 5′ and 3′ termini was used as the query sequence for a BLASTn search against the *P. vampyrus* Illumina whole genome sequencing (WGS) project SRA [SRA: SRP047390] using the NCBI BLASTn suite (http://blast.ncbi.nlm.nih.gov/Blast.cgi; last accessed April 3, 2018) with the following parameters: Program = megablast, maximum target sequences = 500, *E*-values < 1 × 10^−20^, word size = 28, maximum matches in a query range = 13, match score = 1, mismatch cost = 2, gap costs = linear, with no filtering or masking. All hits with a percentage identity of 97% or higher were downloaded and assembled using the CLC Genomics Workbench Assemble Sequences tool into a contiguous consensus sequence using the following parameters: Minimum aligned read length = 20, alignment stringency = high, conflicts = Vote (A, C, G, T). Consensus sequences were aligned with the original query to confirm that the assembly represented a terminally extended match of the original query sequence; they were subsequently used as a new query in an otherwise identical BLASTn search against the same SRA. This process was iteratively repeated until all scaffolds could be extended out far enough that their sequences overlapped with another scaffold. The extended and overlapping scaffolds were joined end to end to create a contiguous reference sequence construct representing the *P. vampyrus A3* gene locus.

To validate the draft *A3* locus reference sequence, all *P. vampyrus* WGS SRA were downloaded from NCBI and locally converted into paired FASTQ data sets using the NCBI SRA Toolkit 2.4.3 (http://www.ncbi.nlm.nih.gov/Traces/sra/sra.cgi; last accessed April 3, 2018). Each data set was subjected to a quality control check using the FastQC program 0.11.3 (Babraham Bioinformatics, Cambridge). Paired FASTQ data sets were imported into CLC Genomics Workbench using the Illumina import function and the CLC Genomics Workbench Next Generation Sequencing (NGS) Toolkit was used for all following tasks. All data sets were trimmed on the basis of quality using the default parameters. Data sets with short paired-read distances were assessed for the presence of overlapping pairs and these were merged where appropriate using the default parameters. The *P. vampyrus* data set included long paired reads with insert sizes of 2–20 kb which are helpful for the resolution of repetitive gene regions. Validation of the *P. vampyrus A3* locus was performed by mapping all reads to the reference sequence construct using the following parameters: Linear gap cost, length fraction = 0.9, similarity fraction 0.9, with automatic detection of paired read distances. Other parameters were left in their default settings. Potential gene coding regions were identified by mapping *P. alecto A3* cDNA sequences against the locus through a local BLASTn analysis using CLC Genomics Workbench with default settings. Promoter elements were predicted using the online Neural Network Promoter Prediction 2.2 tool (Reese 2001) (http://www.fruitfly.org/seq_tools/promoter.html; last accessed April 3, 2018) using the default parameters. ISRE were predicted using the online PROMO transcription factor binding site prediction tool (Farré et al. 2003) (http://alggen.lsi.upc.es/cgi-bin/promo_v3/promo/promoinit.cgi?dirDB=TF_8.3; last accessed April 3, 2018) using the parameters: Species: Mammalia, Factors: Mammalia.

### APOBEC3 Phylogenetic Analysis

The identities of the putative bat *A3* gene products were confirmed by phylogenetic analysis. All reference sequences were downloaded from Ensembl (https://www.ensembl.org/; last accessed April 3, 2018) and aligned with *P. alecto* sequences using MUSCLE (Edgar 2004). Regions of low conservation and uncertain alignment were removed using the Gblocks program (Talavera and Castresana 2007). Phylogenetic relationships were inferred using the maximum likelihood (ML) method available in the MEGA6 program (Tamura et al. 2013), employing nearest-neighbor interchange and branch swapping, and the best-fit model of amino acid substitution was found to be JTT + G (Tamura et al. 2013). To determine the robustness of each node 1,000 bootstrap replications were incorporated.

### Rifampicin Mutagenesis Assay

Bat A3 cDNAs were PCR amplified from parental vectors and subcloned into pET24(+) vectors (EMD Biosciences). Plasmids were transformed into calcium competent C43 (DE3) strain *E. coli* (gift from Dr. Do-Hyung Kim) and grown in LB plates with kanamycin. Single colonies were selected and grown in LB with kanamycin for 28 h to ensure stationary phase growth. Thereafter, cultures were spread on either rifampicin plates or serially diluted in M9 salt media and spread on kanamycin plates. Colony counts were obtained after 18 h. *rpoB* mutation frequency was obtained by dividing the number of viable colonies on rifampicin plates by colonies on kanamycin plates and correcting for the dilution factor. A portion of the *rpoB* gene was PCR amplified using primers: forward 5′-TTGGCGAAATGGCGGAAAACC and reverse 5′-CACCGACGGATACCACCTGCTG. PCR products were enzymatically purified using Exonuclease I and rSAP (NEB). Purified fragments were sequenced using the forward primer (GeneWiz, NJ).

### Human Cell Cultures

Human embryonic kidney cells (293 T cells) and human epithelial cervical adenocarcinoma cells modified to express constitutively high levels of HIV-1 receptors and coreceptors (TZM-bl cells), were utilized in the inhibition of infectivity assay. The TZM-bl cells were obtained through the NIH AIDS Research and Reference Reagent Program (Wei et al. 2002). All cell cultures were maintained at 37°C, 5% CO_2_ in Dulbecco’s modified Eagle’s medium (Thermo) supplemented with heat-inactivated fetal bovine serum (100 ml/l; Invitrogen), glutamine (292 μg/ml; Invitrogen), and the antibiotics penicillin (100 units/ml; Invitrogen) and streptomycin (100 units/ml; Invitrogen).

### A3 Retroviral Restriction Assay

The antiviral activity of bat A3 was assessed by examining the capacity to restrict HIV-1 infectivity. Bat A3 cDNAs were PCR amplified from parental vectors and subcloned into pcDNA3.1 mammalian expression vectors (Thermo Fisher, MA). 293 T cells were cotransfected with different quantities of bat A3 or human A3G (phu-A3GHA; [Mariani et al. 2003]) expression plasmids (25–600 ng) and 400 ng of pIIIBΔvif (Simon et al. 1995), which generates HIV-1 virions in the absence of Vif. Untransfected cells and cells transfected only with pIIIBΔvif were used as controls. Transfected cell cultures were incubated at 37°C, 5% CO_2_ for 48 h, and then virion-containing supernatants were collected and clarified by centrifugation at 200 × g for 5 min. Virus production was determined by quantifying virion-associated RT activity, as previously described (Wapling et al. 2005). TZM-bl cells were inoculated with RT normalized supernatant and incubated at 37°C, 5% CO_2_ for 48 h. HIV-1 infection of TZM-bl cells was assessed through quantitation of luciferase activity in cell lysates as previously described (Tyssen et al. 2010).

### Hypermutation Analysis

The impact of cytosine deamination on the genomes of ancient bat retroviruses was assessed by hypermutation analysis (Lee et al. 2008). The genomes of all ERVs used in the analysis were extracted from the *P. vampyrus* genome. Multiple outgroups were tested to confirm that the position of the ancestral node remained consistent. For the betaretroviruses, we tested Jaagsiekte sheep retrovirus (JSRV) [NCBI: NC_001494], Squirrel monkey retrovirus (SMR) [NCBI: M23385], and the Mouse mammary tumor virus (MMTV) [NCBI: AF033807]; for the gammaretroviruses we tested the Moloney murine leukemia virus (MMLV) [NCBI: NC_001501], the Feline leukemia virus (FLV) [NCBI: AF052723], and the Gibbon ape leukemia virus (GaLV) [NCBI: M26927]. The most closely related betaretrovirus and gammaretrovirus to our ERV groups were JSRV and MMLV, respectively, and were employed as outgroups for the phylogenies. Sequence alignments and phylogenies were conducted as described for the APOBEC3 phylogenetic analysis, and the best-fit model of nucleotide substitution was found to be T92 + G for both groups of retroviruses. Ancestral sequences were computationally extrapolated using the MEGA6 program (Tamura et al. 2013) using the mutations accumulated in each ERV since integration by estimating the ancestral state of each node in the maximum likelihood phylogenetic tree. The state is chosen to be the one that maximizes the probability of the given sequence data. Mutational biases were then evaluated by analyzing an alignment of the ancestral sequence and the ERVs (supplementary data S2 and S3, Supplementary Material online) using the online Hypermut tool (http://www.hiv.lanl.gov/content/sequence/HYPERMUT/hypermut.html; last accessed April 3, 2018) (Rose and Korber 2000) with context enforced on the ancestral reference sequence. The Hypermut tool incorporates Fisher’s exact test to determine statistical significance.

### Molecular Clock Dating Analysis

To estimate the time of the expansion of A3Z1 genes in bat genome, molecular dating was performed using the Bayesian evolutionary method as implemented in BEAST v1.8.2 (Drummond et al. 2012). All reference nucleotide sequences were aligned using MUSCLE and adjusted manually. A recombination test was then performed using the RDP, GENECONV and MaxChi methods implemented in RDP3 (Martin et al. 2010). After removing recombinant sequences, all sequences were submitted to BEAST for estimation of the divergence time. Fossil node calibrations were taken from published literature (Kumar et al. 2017): human and elephant, 100–111 Ma; human and bat, 91–102 Ma; human and macaque, 27.61–31.28 Ma; horse and camel, 74–81 Ma; camel and sheep, 61.1–67.2 Ma; goat and sheep, 8.18–12.62 Ma; sheep and cow, 21.41–29.46 Ma; beluga whale and orca, 16.36–20.43 Ma. Bayesian phylogeny was inferred using the GTR + G substitution model, the Yule speciation tree prior and lognormal relaxed clock (Uncorrelated) prior. The BEAST processes were run for ten million generations until all relevant parameters converged [effective sample size (ESS)>200], with 10% of the Bayesian Markov chain Monte Carlo (MCMC) chains discarded as burn-in.

## Supplementary Material

Supplementary data are available at *Molecular Biology and Evolution* online.

## Supplementary Material

Supplementary Data

## References

[msy048-B1] Almeida FC, Giannini NP, Simmons NB, Helgen KM. 2014 Each flying fox on its own branch: a phylogenetic tree for Pteropus and related genera (Chiroptera: Pteropodidae). Mol Phylogen Evol. 77:83–95. 10.1016/j.ympev.2014.03.009 24662680

[msy048-B2] Amman BR, Albariño CG, Bird BH, Nyakarahuka L, Sealy TK, Balinandi S, Schuh AJ, Campbell SM, Ströher U, Jones MEB et al, . 2015 A recently discovered pathogenic paramyxovirus, Sosuga virus, is present in *Rousettus aegyptiacus* fruit bats at multiple locations in Uganda. J Wildl Dis. 51 3: 774–779., 25919464 10.7589/2015-02-044 PMC5022529

[msy048-B3] Baker ML, Schountz T, Wang LF. 2013 Antiviral immune responses of bats: a review. Zoonoses Public Health 60:104–116. 23302292 10.1111/j.1863-2378.2012.01528.x PMC7165715

[msy048-B4] Berger G, Durand S, Fargier G, Nguyen X-N, Cordeil S, Bouaziz S, Muriaux D, Darlix J-L, Cimarelli A. 2011 APOBEC3A is a specific inhibitor of the early phases of HIV-1 infection in myeloid cells. PLoS Pathog. 7 9: e1002221. 21966267 10.1371/journal.ppat.1002221 PMC3178557

[msy048-B5] Burns MB, Leonard B, Harris RS. 2015 APOBEC3B: pathological consequences of an innate immune DNA mutator. Biomed J. 38 2: 102. 25566802 10.4103/2319-4170.148904

[msy048-B6] Calisher CH, Childs JE, Field HE, Holmes KV, Schountz T. 2006 Bats: important reservoir hosts of emerging viruses. Clin Microbiol Rev. 19 3: 531–545. 16847084 10.1128/CMR.00017-06 PMC1539106

[msy048-B7] Cantrell MA, Scott L, Brown CJ, Martinez AR, Wichman HA. 2008 Loss of LINE-1 activity in the megabats. Genetics 178 1: 393–404. 18202382 10.1534/genetics.107.080275 PMC2206088

[msy048-B8] Chen H, Lilley CE, Yu Q, Lee DV, Chou J, Narvaiza I, Landau NR, Weitzman MD. 2006 APOBEC3A is a potent inhibitor of adeno-associated virus and retrotransposons. Curr Biol. 16 5: 480–485. 16527742 10.1016/j.cub.2006.01.031

[msy048-B9] Chiu YL, Greene WC. 2008 The APOBEC3 cytidine deaminases: an innate defensive network opposing exogenous retroviruses and endogenous retroelements. Annu Rev Immunol. 26:317–353. 18304004 10.1146/annurev.immunol.26.021607.090350

[msy048-B10] Conticello SG, Thomas CJ, Petersen-Mahrt SK, Neuberger MS. 2005 Evolution of the AID/APOBEC family of polynucleotide (deoxy) cytidine deaminases. Mol Biol Evol. 22 2: 367–377. 15496550 10.1093/molbev/msi026

[msy048-B11] Cooke MS, Evans MD, Dizdaroglu M, Lunec J. 2003 Oxidative DNA damage: mechanisms, mutation, and disease. FASEB J. 17 10: 1195–1214. 12832285 10.1096/fj.02-0752rev

[msy048-B12] Cowled C, Baker M, Tachedjian M, Zhou P, Bulach D, Wang L-F. 2011 Molecular characterisation of Toll-like receptors in the black flying fox *Pteropus alecto*. Dev Comp Immunol. 35 1: 7–18. 20692287 10.1016/j.dci.2010.07.006 PMC7103217

[msy048-B13] Cui J, Tachedjian G, Tachedjian M, Holmes EC, Zhang S, Wang L-F. 2012 Identification of diverse groups of endogenous gammaretroviruses in mega and microbats. J Gen Virol. 93(Pt_9): 2037–2045. 22694899 10.1099/vir.0.043760-0 PMC7346494

[msy048-B14] Cui J, Tachedjian G, Wang L-F. 2015 Bats and rodents shape mammalian retroviral phylogeny. Sci Rep. 5:16561. 26548564 10.1038/srep16561 PMC4637884

[msy048-B15] Drummond AJ, Suchard MA, Xie D, Rambaut A. 2012 Bayesian phylogenetics with BEAUti and the BEAST 1.7. Mol Biol Evol. 29 8: 1969–1973. 22367748 10.1093/molbev/mss075 PMC3408070

[msy048-B16] Edgar RC. 2004 MUSCLE: multiple sequence alignment with high accuracy and high throughput. Nucleic Acids Res. 32 5: 1792–1797. 15034147 10.1093/nar/gkh340 PMC390337

[msy048-B17] Fang J, Wang X, Mu S, Zhang S, Dong D. 2015 BGD: a database of bat genomes. PLoS One 10 6: e0131296. 26110276 10.1371/journal.pone.0131296 PMC4482021

[msy048-B18] Farré D, Roset R, Huerta M, Adsuara JE, Roselló L, Albà MM, Messeguer X. 2003 Identification of patterns in biological sequences at the ALGGEN server: PROMO and MALGEN. Nucleic Acids Res. 31 13: 3651–3653. 12824386 10.1093/nar/gkg605 PMC169011

[msy048-B19] Green AM, Landry S, Budagyan K, Avgousti DC, Shalhout S, Bhagwat AS, Weitzman MD. 2016 APOBEC3A damages the cellular genome during DNA replication. Cell Cycle 15 7: 998–1008. 26918916 10.1080/15384101.2016.1152426 PMC4889253

[msy048-B20] Halpin K, Young P, Field H, Mackenzie J. 2000 Isolation of Hendra virus from pteropid bats: a natural reservoir of Hendra virus. J Gen Virol. 81(Pt 8): 1927–1932. 10900029 10.1099/0022-1317-81-8-1927

[msy048-B21] Harris RS, Petersen-Mahrt SK, Neuberger MS. 2002 RNA editing enzyme APOBEC1 and some of its homologs can act as DNA mutators. Mol Cell 10 5: 1247–1253. 12453430 10.1016/s1097-2765(02)00742-6

[msy048-B22] Hayman DT. 2016 Bats as viral reservoirs. Annu Rev Virol. 3 1: 77–99. 27578437 10.1146/annurev-virology-110615-042203

[msy048-B23] Hayward J, Tachedjian M, Cui J, Field H, Holmes E, Wang L-F, Tachedjian G. 2013 Identification of diverse full-length endogenous betaretroviruses in megabats and microbats. Retrovirology 10 1: 35. 23537098 10.1186/1742-4690-10-35 PMC3621094

[msy048-B24] Holden LG, Prochnow C, Chang YP, Bransteitter R, Chelico L, Sen U, Stevens RC, Goodman MF, Chen XS. 2008 Crystal structure of the anti-viral APOBEC3G catalytic domain and functional implications. Nature 456 7218: 121. 18849968 10.1038/nature07357 PMC2714533

[msy048-B25] Jones ME, Schuh AJ, Amman BR, Sealy TK, Zaki SR, Nichol ST, Towner JS. 2015 Experimental inoculation of Egyptian rousette bats (*Rousettus aegyptiacus*) with viruses of the ebolavirus and marburgvirus genera. Viruses 7 7: 3420–3442. 26120867 10.3390/v7072779 PMC4517108

[msy048-B26] Kane JR, Stanley DJ, Hultquist JF, Johnson JR, Mietrach N, Binning JM, Jónsson SR, Barelier S, Newton BW, Johnson TL et al, . 2015 Lineage-specific viral hijacking of non-canonical E3 ubiquitin ligase cofactors in the evolution of Vif anti-APOBEC3 activity. Cell Rep. 11 8: 1236–1250. 25981045 10.1016/j.celrep.2015.04.038 PMC4613747

[msy048-B27] Kinomoto M, Kanno T, Shimura M, Ishizaka Y, Kojima A, Kurata T, Sata T, Tokunaga K. 2007 All APOBEC3 family proteins differentially inhibit LINE-1 retrotransposition. Nucleic Acids Res. 35 9: 2955–2964. 17439959 10.1093/nar/gkm181 PMC1888823

[msy048-B28] Koito A, Ikeda T. 2013 Intrinsic immunity against retrotransposons by APOBEC cytidine deaminases. Front Microbiol. 4:28. 23431045 10.3389/fmicb.2013.00028 PMC3576619

[msy048-B29] Kumar S, Stecher G, Suleski M, Hedges SB. 2017 TimeTree: a resource for timelines, timetrees, and divergence times. Mol Biol Evol. 34 7: 1812–1819. 28387841 10.1093/molbev/msx116

[msy048-B30] Lackey L, Law EK, Brown WL, Harris RS. 2013 Subcellular localization of the APOBEC3 proteins during mitosis and implications for genomic DNA deamination. Cell Cycle 12 5: 762–772. 23388464 10.4161/cc.23713 PMC3610724

[msy048-B31] Land AM, Law EK, Carpenter MA, Lackey L, Brown WL, Harris RS. 2013 Endogenous APOBEC3A DNA cytosine deaminase is cytoplasmic and nongenotoxic. J Biol Chem. 288 24: 17253–17260. 23640892 10.1074/jbc.M113.458661 PMC3682529

[msy048-B32] LaRue R, Jonsson SR, Silverstein KAT, Lajoie M, Bertrand D, El-Mabrouk N, Hotzel I, Andresdottir V, Smith TPL, Harris RS. 2008 The artiodactyl APOBEC3 innate immune repertoire shows evidence for a multi-functional domain organization that existed in the ancestor of placental mammals. BMC Mol Biol. 9 1: 104. 19017397 10.1186/1471-2199-9-104 PMC2612020

[msy048-B33] LaRue RS, Andrésdóttir V, Blanchard Y, Conticello SG, Derse D, Emerman M, Greene WC, Jónsson SR, Landau NR, Löchelt M. 2009 Guidelines for naming nonprimate APOBEC3 genes and proteins. J Virol. 83 2: 494–497. 18987154 10.1128/JVI.01976-08 PMC2612408

[msy048-B34] Lecossier D, Bouchonnet F, Clavel F, Hance AJ. 2003 Hypermutation of HIV-1 DNA in the absence of the Vif protein. Science 300 5622: 1112. 12750511 10.1126/science.1083338

[msy048-B35] Lee YN, Malim MH, Bieniasz PD. 2008 Hypermutation of an ancient human retrovirus by APOBEC3G. J Virol 82 17: 8762–8770. 18562521 10.1128/JVI.00751-08 PMC2519637

[msy048-B36] Mariani R, Chen D, Schröfelbauer B, Navarro F, König R, Bollman B, Münk C, Nymark-McMahon H, Landau NR. 2003 Species-specific exclusion of APOBEC3G from HIV-1 virions by Vif. Cell 114 1: 21–31. 12859895 10.1016/s0092-8674(03)00515-4

[msy048-B37] Marin M, Rose KM, Kozak SL, Kabat D. 2003 HIV-1 Vif protein binds the editing enzyme APOBEC3G and induces its degradation. Nat Med. 9 11: 1398–1403. 14528301 10.1038/nm946

[msy048-B38] Martin DP, Lemey P, Lott M, Moulton V, Posada D, Lefeuvre P. 2010 RDP3: a flexible and fast computer program for analyzing recombination. Bioinformatics 26 19: 2462–2463. 20798170 10.1093/bioinformatics/btq467 PMC2944210

[msy048-B39] Metzenberg A, Wurzer G, Huisman T, Smithies O. 1991 Homology requirements for unequal crossing over in humans. Genetics 128 1: 143–161. 2060774 10.1093/genetics/128.1.143 PMC1204444

[msy048-B40] Middleton DJ, Morrissy CJ, van der Heide BM, Russell GM, Braun MA, Westbury HA, Halpin K, Daniels PW. 2007 Experimental Nipah virus infection in pteropid bats (*Pteropus poliocephalus*). J Comp Pathol. 136 4: 266–272. 17498518 10.1016/j.jcpa.2007.03.002

[msy048-B41] Muckenfuss H, Hamdorf M, Held U, Perković M, Löwer J, Cichutek K, Flory E, Schumann GG, Münk C. 2006 APOBEC3 proteins inhibit human LINE-1 retrotransposition. J Biol Chem. 281 31: 22161–22172. 16735504 10.1074/jbc.M601716200

[msy048-B42] Nakano Y, Aso H, Soper A, Yamada E, Moriwaki M, Juarez-Fernandez G, Koyanagi Y, Sato K. 2017 A conflict of interest: the evolutionary arms race between mammalian APOBEC3 and lentiviral Vif. Retrovirology 14 1: 31. 28482907 10.1186/s12977-017-0355-4 PMC5422959

[msy048-B43] O’Shea TJ, Cryan PM, Cunningham AA, Fooks AR, Hayman DT, Luis AD, Peel AJ, Plowright RK, Wood JL. 2014 Bat flight and zoonotic viruses. Emerging Infect Dis. 20 5: 741. 24750692 10.3201/eid2005.130539 PMC4012789

[msy048-B44] Papenfuss AT, Baker ML, Feng Z-P, Tachedjian M, Crameri G, Cowled C, Ng J, Janardhana V, Field HE, Wang L-F. 2012 The immune gene repertoire of an important viral reservoir, the Australian black flying fox. BMC Genomics 13 1: 261. 22716473 10.1186/1471-2164-13-261 PMC3436859

[msy048-B45] Perez-Caballero D, Soll SJ, Bieniasz PD. 2008 Evidence for restriction of ancient primate gammaretroviruses by APOBEC3 but Not TRIM5α proteins. PLoS Pathog. 4 10: e1000181. 18927623 10.1371/journal.ppat.1000181 PMC2564838

[msy048-B46] Rahman SA, Hassan SS, Olival KJ, Mohamed M, Chang L-Y, Hassan L, Saad NM, Shohaimi SA, Mamat ZC, Naim MS et al, . 2010 Characterization of Nipah virus from naturally infected Pteropus vampyrus bats, Malaysia. Emerging Infect Dis. 16 12: 1990., 21122240 10.3201/eid1612.091790 PMC3294568

[msy048-B47] Reese MG. 2001 Application of a time-delay neural network to promoter annotation in the *Drosophila melanogaster* genome. Comput Chem. 26 1: 51–56. 11765852 10.1016/s0097-8485(01)00099-7

[msy048-B48] Refsland EW, Harris RS. 2013 The APOBEC3 family of retroelement restriction factors In: Cullen BR, editor. Intrinsic immunity. Berlin Heidelberg: Springer p. 1–27. 10.1007/978-3-642-37765-5_1 PMC3934647 23686230

[msy048-B49] Refsland EW, Stenglein MD, Shindo K, Albin JS, Brown WL, Harris RS. 2010 Quantitative profiling of the full APOBEC3 mRNA repertoire in lymphocytes and tissues: implications for HIV-1 restriction. Nucleic Acids Res. 38 13: 4274–4284. 20308164 10.1093/nar/gkq174 PMC2910054

[msy048-B50] Renard M, Henry M, Guétard D, Vartanian J-P, Wain-Hobson S. 2010 APOBEC1 and APOBEC3 cytidine deaminases as restriction factors for hepadnaviral genomes in non-humans in vivo. J Mol Biol. 400 3: 323–334. 20546753 10.1016/j.jmb.2010.05.029

[msy048-B51] Roberts SA, Lawrence MS, Klimczak LJ, Grimm SA, Fargo D, Stojanov P, Kiezun A, Kryukov GV, Carter SL, Saksena G et al, . 2013 An APOBEC cytidine deaminase mutagenesis pattern is widespread in human cancers. Nat Genet. 45 9: 970–976. 23852170 10.1038/ng.2702 PMC3789062

[msy048-B52] Rose PP, Korber BT. 2000 Detecting hypermutations in viral sequences with an emphasis on G→A hypermutation. Bioinformatics 16 4: 400–401. 10869039 10.1093/bioinformatics/16.4.400

[msy048-B53] Sheehy AM, Gaddis NC, Choi JD, Malim MH. 2002 Isolation of a human gene that inhibits HIV-1 infection and is suppressed by the viral Vif protein. Nature 418 6898: 646. 12167863 10.1038/nature00939

[msy048-B54] Sheehy AM, Gaddis NC, Malim MH. 2003 The antiretroviral enzyme APOBEC3G is degraded by the proteasome in response to HIV-1 Vif. Nat Med. 9 11: 1404–1407. 14528300 10.1038/nm945

[msy048-B55] Shen Y-Y, Liang L, Zhu Z-H, Zhou W-P, Irwin DM, Zhang Y-P. 2010 Adaptive evolution of energy metabolism genes and the origin of flight in bats. Proc Natl Acad Sci U S A. 107 19: 8666–8671. 20421465 10.1073/pnas.0912613107 PMC2889356

[msy048-B56] Shi K, Carpenter MA, Kurahashi K, Harris RS, Aihara H. 2015 Crystal structure of the DNA deaminase APOBEC3B catalytic domain. J Biol Chem. 290 47: 28120–28130. 26416889 10.1074/jbc.M115.679951 PMC4653671

[msy048-B57] Simon J, Southerling TE, Peterson JC, Meyer BE, Malim MH. 1995 Complementation of vif-defective human immunodeficiency virus type 1 by primate, but not nonprimate, lentivirus vif genes. J Virol. 69 7: 4166–4172. 7769676 10.1128/jvi.69.7.4166-4172.1995 PMC189153

[msy048-B58] Smith I, Wang L-F. 2013 Bats and their virome: an important source of emerging viruses capable of infecting humans. Curr Opin Virol. 3 1: 84–91. 23265969 10.1016/j.coviro.2012.11.006 PMC7102720

[msy048-B59] Stenglein MD, Harris RS. 2006 APOBEC3B and APOBEC3F inhibit L1 retrotransposition by a DNA deamination-independent mechanism. J Biol Chem. 281 25: 16837–16841. 16648136 10.1074/jbc.M602367200

[msy048-B60] Swanepoel R, Leman PA, Burt FJ, Zachariades NA, Braack LE, Ksiazek TG, Rollin PE, Zaki SR, Peters CJ. 1996 Experimental inoculation of plants and animals with Ebola virus. Emerging Infect Dis. 2 4: 321. 8969248 10.3201/eid0204.960407 PMC2639914

[msy048-B61] Talavera G, Castresana J. 2007 Improvement of phylogenies after removing divergent and ambiguously aligned blocks from protein sequence alignments. Syst Biol. 56 4: 564–577. 17654362 10.1080/10635150701472164

[msy048-B62] Tamura K, Stecher G, Peterson D, Filipski A, Kumar S. 2013 MEGA6: molecular evolutionary genetics analysis version 6.0. Mol Biol Evol. 30 12: 2725–2729. 24132122 10.1093/molbev/mst197 PMC3840312

[msy048-B63] Taylor BJ, Nik-Zainal S, Wu YL, Stebbings LA, Raine K, Campbell PJ, Rada C, Stratton MR, Neuberger MS. 2013 DNA deaminases induce break-associated mutation showers with implication of APOBEC3B and 3A in breast cancer kataegis. Elife 2:e00534. 23599896 10.7554/eLife.00534 PMC3628087

[msy048-B64] Tyssen D, Henderson SA, Johnson A, Sterjovski J, Moore K, La J, Zanin M, Sonza S, Karellas P, Giannis MP et al, . 2010 Structure activity relationship of dendrimer microbicides with dual action antiviral activity. PLoS One 5 8: e12309. 20808791 10.1371/journal.pone.0012309 PMC2925893

[msy048-B65] Wapling J, Moore KL, Sonza S, Mak J, Tachedjian G. 2005 Mutations that abrogate human immunodeficiency virus type 1 reverse transcriptase dimerization affect maturation of the reverse transcriptase heterodimer. J Virol. 79 16: 10247–10257. 16051818 10.1128/JVI.79.16.10247-10257.2005 PMC1182633

[msy048-B66] Warren CJ, Xu T, Guo K, Griffin LM, Westrich JA, Lee D, Lambert PF, Santiago ML, Pyeon D. 2015 APOBEC3A functions as a restriction factor of human papillomavirus. J Virol. 89 1: 688–702. 25355878 10.1128/JVI.02383-14 PMC4301161

[msy048-B67] Watanabe S, Masangkay JS, Nagata N, Morikawa S, Mizutani T, Fukushi S, Alviola P, Omatsu T, Ueda N, Iha K et al, . 2010 Bat coronaviruses and experimental infection of bats, the Philippines. Emerging Infect Dis. 16 8: 1217–1223. 20678314 10.3201/eid1608.100208 PMC3298303

[msy048-B68] Wei X, Decker JM, Liu H, Zhang Z, Arani RB, Kilby JM, Saag MS, Wu X, Shaw GM, Kappes JC. 2002 Emergence of resistant human immunodeficiency virus type 1 in patients receiving fusion inhibitor (T-20) monotherapy. Antimicrob Agents Chemother. 46 6: 1896–1905. 12019106 10.1128/AAC.46.6.1896-1905.2002 PMC127242

[msy048-B69] Williamson MM, Hooper PT, Selleck PW, Gleeson LJ, Daniels PW, Westbury HA, Murray PK. 1998 Transmission studies of Hendra virus (equine morbilli-virus) in fruit bats, horses and cats. Aust Vet J. 76 12: 813–818. 9972433 10.1111/j.1751-0813.1998.tb12335.x

[msy048-B70] Wissing S, Montano M, Garcia-Perez JL, Moran JV, Greene WC. 2011 Endogenous APOBEC3B restricts LINE-1 retrotransposition in transformed cells and human embryonic stem cells. J Biol Chem. 286 42: 36427–36437. 21878639 10.1074/jbc.M111.251058 PMC3196128

[msy048-B71] Yu Q, Chen D, König R, Mariani R, Unutmaz D, Landau NR. 2004 APOBEC3B and APOBEC3C are potent inhibitors of simian immunodeficiency virus replication. J Biol Chem. 279 51: 53379–53386. 15466872 10.1074/jbc.M408802200

[msy048-B72] Zhang G, Cowled C, Shi Z, Huang Z, Bishop-Lilly KA, Fang X, Wynne JW, Xiong Z, Baker ML, Zhao W et al, . 2013 Comparative analysis of bat genomes provides insight into the evolution of flight and immunity. Science 339 6118: 456–460. 23258410 10.1126/science.1230835 PMC8782153

[msy048-B73] Zheng Y-H, Irwin D, Kurosu T, Tokunaga K, Sata T, Peterlin BM. 2004 Human APOBEC3F is another host factor that blocks human immunodeficiency virus type 1 replication. J Virol. 78 11: 6073–6076. 15141007 10.1128/JVI.78.11.6073-6076.2004 PMC415831

[msy048-B74] Zhou P, Tachedjian M, Wynne JW, Boyd V, Cui J, Smith I, Cowled C, Ng JH, Mok L, Michalski WP. 2016 Contraction of the type I IFN locus and unusual constitutive expression of IFN-α in bats. Proc Natl Acad Sci U S A. 201518240. 10.1073/pnas.1518240113 PMC4790985 26903655

