# Supplementary material for: Differential Evolution of Antiretroviral Restriction Factors in Pteropid Bats as Revealed by APOBEC3 Gene Complexity
Source: Mol Biol Evol. 2018 Mar 29;35(7):1626–37. doi: 10.1093/molbev/msy048 (PMC5995163; doi:10.1093/molbev/msy048)
Supplement: Supplementary Data [file msy048_supp.zip › Supplementary Data 2 - Betaretrovirus alignment.pdf]

```
                                                20                                        40                                        60
                                                |                                         |                                         |
Ancestral_G7_Beta   TGGCGCCCGAACAGGGACCTGAAAACGGGGAATAGCAAGGACCCACCGCAAGACGAGTGC  60
  scaffold_12699    TGGCGCCCGAACAGGGACCTGAAAACGGGGAATAGCAAGGACCCACCACAAGGCGAGTGC  60
   scaffold_2273    TGGCGCCCGAACAGGGACCTGAAAACGGGGAATAGCAAGGACCCACCGCAAGACGAGTGC  60
GeneScaffold_1344   TGGCGCCCGAACAGGGACCTGAAAATGGGGAAAAGCAAGGACCCACCGCAAGACGAGTGC  60
   scaffold_9648    TGGCGCCCGAACAGGGACCTGAAAACGGGGAAAAGCAAGGACCCACCGCAAGACGAGTGC  60
  scaffold_17393    TGGCGCCCAAACAGGGACCTGAAAACGGGGGGAAGCAAGGACCCACCGCAAGACGAGTGC  60
  scaffold_12793    TGGCGCCGGAACGGGGACCTGAAAACGGGGAATAGCAAGGACCCACCGCAAGACGAGTGC  60

                                                80                                        100                                       120
                                                |                                         |                                         |
Ancestral_G7_Beta   ACTCGAGTTGGCCAAAGAAGCCGTGGGCGGTGAAGGTGAGTACGCAGGAGTTTTTGTATA  120
  scaffold_12699    ACTCGAGTTGGCCAAAGAAGCCGTGGGCGGTGAAGGTGAGTATGCAGGAGTTTTTGTATA  120
   scaffold_2273    AGTCAAGTTGGCCAAAGAAGCCGTGGGCGGTGAAGGTGAGTACGCAGGAGTTTTTGTATA  120
GeneScaffold_1344   ACTCGAGTTGGCCAAAGAAGCCGTGGGCGGTGAAGGTGAGTACGCAGGAGTTTTTGTATA  120
   scaffold_9648    ACTCGAGTTGGCCAAAGAAGCCGTGGGCGGTGAAGGTGAGTACGCAGGAGTTTTTGTATA  120
  scaffold_17393    ACTAGAGTTGGCCAAAGAAGCCGTGGGCGGTGAAGGTGAGTACGCAGGAGTTTTTGTATA  120
  scaffold_12793    ACTCGAGTTGGCCAAAGAAGCCGTGGGCGGTGAAGGTGAGTATGCAGGAGTTTTTGTATA  120

                                                140                                       160                                       180
                                                |                                         |                                         |
Ancestral_G7_Beta   GAAACTGAAAGGAAAATATAATGGGCCAAGGAAATAGTCGACTGCTATTTGTACATATGC  180
  scaffold_12699    GAAACTGAAAGGAAGATATAATGGGCCAAGGAAATAGTCGACTGCTATTTGTACATATGC  180
   scaffold_2273    GAAACTGAAAGGAAAATATAATGGGCCAAGGAAATAGTCGACTGCTATTTGTGCATATGC  180
GeneScaffold_1344   GAAACTGTAAGGAAAATATAATGGGCCAAGGAAATAGTCGACTGCTATTTGTACATATGC  180
   scaffold_9648    GAAACTGTAAGGAAAATATAATGGGCCAAGGAAATAGTCGACTGCTATTTGTACATATGC  180
  scaffold_17393    GAAACTGTAAGGAAAATATAATGGGCCAAGGAAATAGTCGACTGCTATTTGTACATATGC  180
  scaffold_12793    GAAACTGAAAGGAAAATATAATGGGCCAAGGAAATAGTCGACTGCTATTTGTACATATGC  180

                                                200                                       220                                       240
                                                |                                         |                                         |
Ancestral_G7_Beta   TTAAGAATATGCTTAGAGTTAGAGGAGCTAAATAGGTCAGCAACAGTTAATGAAGTTCTT  240
  scaffold_12699    TTAAGAATATGCTTAGAGTTAGAGGAGCTAAATAGGTCAGCAACAGTTAATGAAGTTCTT  240
   scaffold_2273    TTAAGAATACGCTTAGAGTTAGAGGAGCTAAATAGGTCAGCAACAGTTAATGAAGTTCTT  240
GeneScaffold_1344   TTAAGAATATGCTTAGAGTTAGAGGAGCTAAATAGCTCAGCAACAGTTAATGAAGTTTTT  240
   scaffold_9648    TTAAGAATATGCTTAGAGTTAGAGGAGCTAAATAGCTCAGCAACAGTTAATGAAGTTTTT  240
  scaffold_17393    TTAAGAATATGCTTAGAGTTAGAGAAGCTAAATAGCTCAGCAACAGTTAATGAAGTTTTT  240
  scaffold_12793    TTAAGAATATGCTTAGAGTTAGAGGAGCTAAATAGGTCAGCAACAGTTAATGAAGTTCTT  240

                                                260                                       280                                       300
                                                |                                         |                                         |
Ancestral_G7_Beta   GCAATTTATAGAAGAAGTTTGTCCCTGGTTTCCTGAGGAAGGGACAATTGATTTAGAGAC  300
  scaffold_12699    GCAATTTATAGAAGAAGTTTGTCCCTGGTTCCCTGAGGAAGGGACAATTGATTTAGAGAC  300
   scaffold_2273    GCAATTTATAGAATAAGTTTGTCCCTGGTTTCCTGAGGAAGGGACAATTGATTTAGAGAC  300
GeneScaffold_1344   GCAATTTATAGAAGAAGTTTGTCCCTGGTTCCCTGAGGAAGGGACAATTGATTTAGAGAC  300
   scaffold_9648    GCAATTTATAGAAGAAGTTTGTCCCTGGTTCCCTGAGGAAGGGACAATTGATTTAGAGAC  300
  scaffold_17393    GCAATTTATAGAAGAAGTTTGTCCCTGGTTCCCTGAGGAAGGGACAATTGATTTAGAGAC  300
  scaffold_12793    GCAATTTATAGAAGAAGTTTGTCCCTGGTTTCCTGAGGAAGGGACAATTGATTTAGAGAC  300

                                                320                                       340                                       360
                                                |                                         |                                         |
Ancestral_G7_Beta   ATGGTTAAGAGTGGGTCAAAAGTTACAGGATTACTATGATGCCCACGGACCTACTAAGGT  360
  scaffold_12699    ATGGTTAAGAGTGGGTAAAAAGTTACAGGATTACTATGATGACCACGGACCTACTAAGGT  360
   scaffold_2273    ATGGTTAAAAGTGGGTCAAAAGTTACAGGATTACTATGATGCCCACGGACCTACTAAGGT  360
GeneScaffold_1344   ATGGTTAAGAGTGGGTAAAAAGTTACAGGATTACTATGATGACCACGGACCTACTAAGGT  360
   scaffold_9648    ATGGTTAAGAGTGGGTAAAAAGTTACAGGATTACTATGATGACCACGGACCTACTAAGGT  360
  scaffold_17393    ATGGTTAAGAGTGGGTAAAAAGTTACAGGATTACTATGATGACCACGGACCTACTAAGGT  360
  scaffold_12793    ATGGTTAAGAGTGGGTCAAAAGTTACAGGATTACTACGATGCCCACGGACCTACTAAGGT  360

                                                380                                       400                                       420
                                                |                                         |                                         |
Ancestral_G7_Beta   TCCGGTAGATACTTTTGGGCTTTGGACTTTGATTAGATTCTTTAGACCCTAGGCATGACA  420
  scaffold_12699    TCCGGCAGATACTTTTGGGCTTTGGACTTTGATTAGATTCTTCAGACCCTAGGCATGACA  420
   scaffold_2273    TCCGGTAGATACTTTTGGGCTTTTGACTTTGATTAGATTCTTTAGACCCTAGGCATGACA  420
GeneScaffold_1344   TCCGGCAGATACTCTTGGGCTTTGGACTCTGATTAGATTCTTTAGACCCTAGGCATGACA  420
   scaffold_9648    TCCGGCAGATACTTTTGGGCTTTGGACTTTGATTAGATTCTTTAGACCCTAGGCATGACA  420
  scaffold_17393    TCCGGCAGATACTTTTGGGCTTTGGACTTTGATTAGATTCTTTAGACCCTAGGCATGACA  420
  scaffold_12793    TCCGGTAGATACTTTTGGGCTTTGGACTTTGATTAGATTCTTTAGACCCTAGGCATGACA  420
```

```
                                    440                 460                 480
                                      |                   |                   |
Ancestral_G7_Beta  GATTAAAGAACAAATGTCACCTGTTAAAACATGTTCATGAGTATGACGAATCTGAGGCTG 480
   scaffold_12699  GATTAAAGAACAAATGTCACCTGTTAAAACATGTTCATGGGTATGATGAATCTGAGGCTG 480
    scaffold_2273  GATTAAAGAACGAATGTCACCTGTTAAAACATGTTCATGAGTATGACGAATCTGAGGCTG 480
GeneScaffold_1344  GATTAAAGAACAAATGTCTCCTGTTAAAACATGTTCATGAGTATGACGAATCTGAGGCTG 480
    scaffold_9648  GATTAAAGAACAAATGTCACCTGTTAAAACATGTTCATGAGTATGACGAATCTGAGGCTG 480
   scaffold_17393  GATTAAAGAACAAATGTCTCCTATTAAAACATGTTCATGAGTATGACGAATCTGAGGCTG 480
   scaffold_12793  GATTAAAGAACAAATGTCACCTGTTAAAAAATGTTCATGAGTATGACGAATCTGAGGCTG 480

                                    500                 520                 540
                                      |                   |                   |
Ancestral_G7_Beta  CTGGGGTTGGCCTTCTGCCCCTCCGATCTCCCCCTCCTTTCGACTCCATTTTTACTGATG 540
   scaffold_12699  CTGGGGTTGGCCTTCTGCCCCTCCGATCTCCCCCTCCTTTCGACTCCATTTTTACTGATG 540
    scaffold_2273  CTGGGGTTGGCCTTCTGCTCCTCCGATCTCCCCTTCCTTTCGACTCCATTTTTACTGATG 540
GeneScaffold_1344  CTGGGGTTGGCCTTCTGCTCCTCCGATCTCCCCCTCCTTTCAACTCCATTTTTACTGATG 540
    scaffold_9648  CTGGGGTTGGCCTTCTGCCCCTCCGATCTCCCCCTCCTTTCGACTCCATTTTTACTGATG 540
   scaffold_17393  CTAGGGTTGGCCTTCTGCTCCTCCGATCTCCCCCTCCTTTCAACTCCATTTTTACTGATG 540
   scaffold_12793  CTGGGGTTGGCCTTCTGCCCCTCCGATCTCCCTCTCCTTTCGACTCCATTTTTACTGATG 540

                                    560                 580                 600
                                      |                   |                   |
Ancestral_G7_Beta  ATGAGGAGGAGGAGGCCGCTAGATATCATTATGAAGATTCAGCTCCACTTGTAGCTATTG 600
   scaffold_12699  AGGAGGAGGAGGAGGCCGCTAGATATCATTATGAAGATTCAGCTCCACTTGTAGCTATAG 600
    scaffold_2273  ATAAGGAGGAGGAGGCCGCTAGATATCATTATGAAGATTCAGCTCCACTTGTAGCTATTG 600
GeneScaffold_1344  AGGAGGAGGAGGAGGCCGCTAGATATTATTATGAAAATTCAGCTCCACTTGTAGCTATTG 600
    scaffold_9648  ATACGGAGGAGGAGGCCGCTAGATATCATTATGAAAATTCAGCTCCACTTGTAGCTATTG 600
   scaffold_17393  AGGAGGAGGAGGAGGCCGCTAGATATTATTGTGAAAATTCAGCTCCACTTGTAGCTATTG 600
   scaffold_12793  ATGAGGAGGAGGAGGCCGCTAGATATCATTATGAAGATTCAGCTCCACTTGTAGCTATTG 600

                                    620                 640                 660
                                      |                   |                   |
Ancestral_G7_Beta  GAGGCGTTAACATTTAAAGATGATCAGCTTAAGCGCTTAAGCAACTTACTGTGCAAAAAG 660
   scaffold_12699  GAGGCGTTAACATTTAAAGATGATCAGCTTAAGCGCTTAAGCAACTTACTGAGCAAAAAA 660
    scaffold_2273  GAGGCGTTAACATTTAAAGATGATCAGCTTAAGCGCTTAAGCAACTTACTGTGCAAAAAG 660
GeneScaffold_1344  GAGGCGTTAACATTTAGAGATGATCAGCTTGAGCGCTTAAGCAACTTACTGTGCAAAAAA 660
    scaffold_9648  GAGGCGTTAACATTTAAAGATGATCAGCTTAAGCGCTTAAGCAACTTACTGTGCAAAAAG 660
   scaffold_17393  GAGGCGTTAACATTTAGAGATGATCAGCTTGAGCGCTTAAGCAACTTACTGTGCAAAAAA 660
   scaffold_12793  GAGGCGTTAACATTTAAAGATGATCAGCTTAAGCGCTTAAGCAACTTACTGTGCAAAAAG 660

                                    680                 700                 720
                                      |                   |                   |
Ancestral_G7_Beta  CGGGAAACAGGCCTGCTATGGACACTCCTAAAAATCTGACTCCGATTTTTTGCATCCTCT 720
   scaffold_12699  CGAGAAACAGGCCTGCTATGGACACTCCTAAAAATCTGACTCCGATTTTTTGCATCCTCT 720
    scaffold_2273  CGGGAAACAAGCCTGCTATGGACACTCCTAAAATTCTGACTCCGATTTTTTGCATCCTCT 720
GeneScaffold_1344  CAGGAAACAGGCCTCCTATGGACACTCCTAAAAATCTGACTCCGATTTTTCGCATCCTCT 720
    scaffold_9648  TGGGAAACAGGCCTGCTATGGACACTCTTAAAAATCTGACTAAGATTTTTTGCATCCTCT 720
   scaffold_17393  CGGGAAACAGGCCTCCTATGGACACTCCTAAAAATCTGACTCCGATTTTTCGCATCCTCT 720
   scaffold_12793  CAGGAAACAAGCCTGCTATGGACACTCCTAAAAATATGACTCCGATTTTTTGCATCCTCT 720

                                    740                 760                 780
                                      |                   |                   |
Ancestral_G7_Beta  ACATCCCACAGTTATAGCTGGTGTGGACCCTCCTCCAGTGCTTATTCCTGCACTTAAGGT 780
   scaffold_12699  ACATCCCACAGTTATAGCTGGTGTGGACCCTCCTCCAGTGCTTATTCCTGCACTTAAAGT 780
    scaffold_2273  ACATCCCACATCTATAGCTGGTATGGACCCTCCTCCAGTGCTTATTCCTGTATTTAAGGT 780
GeneScaffold_1344  TCATCCCACAGTTATAGCTGGTGTGGACCCTCCTCCAGTGCTTATACCTGCACTTAAGGT 780
    scaffold_9648  ACATCCCACAGTTATAGCTGGTGTGGACCCTCCTCCACTGCTTATTCCTGCACTTAAGGT 780
   scaffold_17393  ACATCCCACAGTTATAGCTGGTGTGGACCCTCCTCCAGTGCTTATACCTGCACTTAAGGT 780
   scaffold_12793  ACATCCCACAGTTATAGCTGGTGTGGACCCTCCTCCAGTGCTTATTCCTGCACTTAAGGT 780

                                    800                 820                 840
                                      |                   |                   |
Ancestral_G7_Beta  TGAGACTGGCTTACAGAAGGGGCTTAGAATAGCGCAGCTAATGGAGACAATATCGGAGAT 840
   scaffold_12699  TGAGACTGGCTTACAGAAGGGCCTTGGAATAGCGCAGCTAATGGAGACAATATCGGAGAT 840
    scaffold_2273  TGAGACTGGCTTACAGAAGGGGCTTAGAATAGCGCAGCTAATGGAGACAATATCGGAGAT 840
GeneScaffold_1344  TGAGACTGGCTTACAGAAGGGGCTTAGAATAGCGCAGCTAATGGAGACAATATCGGAGAT 840
    scaffold_9648  TGAGACTGGCTTACAGAAGGGGCTTAGAATAGCGCAGCTAATGGAGACAATATCGGAGAT 840
   scaffold_17393  TGAGACTGGCTTACAGAAGGGGCTTAGAATAGCGCAGCTAATGGAGACAATATCGGAGAT 840
   scaffold_12793  TGAGACTGGCTTACAGAAGGGGCTTAGAATAGCGCAGCTAATGGAGACAATATCGGAGAT 840
```

```
                                       860                                      880                                     900
                                        |                                        |                                       |
Ancestral_G7_Beta  TTTAAAATGGCTTTTCCAGTAATAGATCAGCATGATCAACGGGGACATGAGCCTTTGCCT  900
    scaffold_12699  TTTAGAATGGCTTTTCCAGTAATAGATCAGCATGATCAACGGGGACATGAGCCTTTGCCT  900
     scaffold_2273  TTTAAAATGGCTTTTCCAGTAATAGATCAGCATGATCAACGAGTACATGAGCCTTTGCCT  900
GeneScaffold_1344  TTTAAAATGGCTTTTCCAGTAATAGATCAGCATGATCAACGGGGACATGAGCCTTTACCT  900
     scaffold_9648  TTTAAAATGGCTTTTCCAGTAATAGATCAGCATGATCAACGGAGACATGAGCCTTTGCCT  900
    scaffold_17393  TTTAAAATGGCTTTTCCAGTAATAGATCAGCATGATCAACGGGGACATGAGCCTTTACCC  900
    scaffold_12793  TTTAAAATGGCTTTTCCAGTAATAGATCAGCATGATCAACGGGGACATGAGCCTTTGCCT  900

                                       920                                      940                                     960
                                        |                                        |                                       |
Ancestral_G7_Beta  TTTAAGGTATTAAAAGAATTGAAAATGGCTTGTGCTCAATATGGAGCTACTGCTCCATTT  960
    scaffold_12699  TTTAAGGTATTAAAAGAATTGAAAATGGCTTGTGCTCAATATGGAGCTACTGCTCCATTT  960
     scaffold_2273  TTTAAGGTATTAAAAGAATTGAAAGTGGCTTGTACTCAATATGGAGCTACTGCTCCATTT  960
GeneScaffold_1344  TTTAAGATATTAAAAGAATTGAAAACGGCTTGTGCTCAATATGGAGCTACCGCTCCGTTT  960
     scaffold_9648  TTTAGGGTATTAAAAGAATTGAAAATGGCTTGTGCTCAATATGGAGCTACTACTCCATTT  960
    scaffold_17393  TTTAAGATATTAAAAGAATTGAAAATGGCTTGTGCTCAATATGGAGCTACCGCTCCGTTT  960
    scaffold_12793  TTTAAGGTATTAAAAGAATTGAAAATGGCTTGTGCTCAATATGGAGCTACTGCTCCATTT  960

                                       980                                     1,000                                   1,020
                                        |                                        |                                       |
Ancestral_G7_Beta  ACTTTGGCTCAGGTGGAAGGACTTACGGCTTCTGCCCTGACGCCTTGGGATTGGAGAATT  1020
    scaffold_12699  ACTGTGGCTCGGGTGGAAGGACTTTCGGCTTCTGCCCTGACGCCTTGGGATTGGAGAATT  1020
     scaffold_2273  ACTTTGGCTCAGGTGGAAGGACTTACGGCTTCTGCCCTGACGCCTTGGGATTGGAGAATT  1020
GeneScaffold_1344  ACCTTGGCTCAGGTGGAAGGACTTACAGCTTCTGCCCTGACGCCTTGGGATTGGAGAATT  1020
     scaffold_9648  ACTTTGGCTCAGGTGGAAGGACTTACGGCTTCTGCTCTGACGCCTTGGGATTGGAGAATT  1020
    scaffold_17393  ACTTTGGCTCAGGTGGAAGGACTTACGGCTTCTGCCCTGATGCCTTGGGATTGGAGAATT  1020
    scaffold_12793  ACTTTGGCTCAGGTGGAAGGACTTACGGCTTCTGCCCTGACGCCTTGGGATTGGAGAATT  1020

                                      1,040                                    1,060                                   1,080
                                        |                                        |                                       |
Ancestral_G7_Beta  GTGGCAAGAGCTTGCTTATCCGATGGGGATTTTTTATTATGGGATACTTCTTTTAGAGAG  1080
    scaffold_12699  GTGGCAAGAGCTTGCTTATCCGATGAGGATTTTTTATTATGGGATACTTCTTTTAGAGAG  1080
     scaffold_2273  GTGGCAAGAGCTTGCTTATCTGATGAGGATTTTTTATTATGGGATACTTCTTTTAGAGAG  1080
GeneScaffold_1344  GTGGCAAGAGCTTGCTTACCCGGTGGGGATTTTTTATTACGGGATACTTCTTTTAGAGAG  1080
     scaffold_9648  GTGGCAAGAGCTTGCTTATCCGATGAGGATTTTTTATTATGGGATACTTCTTTTAGAGAG  1080
    scaffold_17393  GTGGCAAGAGCTTGCTTACCCGGTGGGGATTTTTTATTATGGGATACTTCTTTTAGAGAG  1080
    scaffold_12793  GTGGCAAGAGCTTGCTTATCCGATGAGGATTTTTTATTATGGGATACTTCTTTTAGAGAG  1080

                                      1,100                                    1,120                                   1,140
                                        |                                        |                                       |
Ancestral_G7_Beta  CATTGTGAAAGGACTGCTAAATTAAACAGCCAATAATGGAATTACTTATGATATGCTTGC  1140
    scaffold_12699  CATTGTGAAAGGACTGCTAAATTAAACAGCCAATAATGGAATTACTTATGATATGCTTGC  1140
     scaffold_2273  CATTGTGAAAGGACTGCTAAATTAAACAGCCAATAATGGAATTACTTATGATATGCTTGC  1140
GeneScaffold_1344  CATTGTGAAAGGACTGCTAAATTAAACAGCCAATAATGGAATTACTTACGATATGCTTGC  1140
     scaffold_9648  CATTGTGAAAGGACTGCTAAATTAAACAGCCAATAATGGAATTACTTACGATATGCTTGC  1140
    scaffold_17393  CATTGTGAAAGGACTGCTAAATTAAACAGCCAATAATGGAATTACTTACGATATGCTTGC  1140
    scaffold_12793  CATTGTGAAAGGACTGCTAAATTAAACAGCCAATAATGGAATTACTTATGATATGCTTGC  1140

                                      1,160                                    1,180                                   1,200
                                        |                                        |                                       |
Ancestral_G7_Beta  TGGTGAAGGTCCATATAAAGAAAATGTAAACCAATTAACTTATCCTGCTGGCGCTTATGC  1200
    scaffold_12699  TGGTGAAGGTCCATATAAAGAAAATGTAAACCAATTAACTTATCCTGCTGGCGCTTATGC  1200
     scaffold_2273  TGGTGAAGGTCCATATAAAGAAAATGTAAACCAATTAACTTATCCTGCTGGCGCTTATGC  1200
GeneScaffold_1344  TGGTGAAGGTCCATATAAAGAAAATGTAAACCAGTTAACTTATCCTGCTGGCGCTTATGC  1200
     scaffold_9648  TGGTGAAGGTCCATATAAAGAAAATGTAAACCAGTTAACTTATCCTGCTGGCGCTTATGC  1200
    scaffold_17393  TGGTGAAGGTCCATATAAAGAAAATGTAAACCAGTTAACTTATCCTGTTGGCGCTTATGC  1200
    scaffold_12793  TGGTGAAGGTCCATATAAAGAAAATGTAAACCAATTAACTTATCCTGCTGGCGCTTATGC  1200

                                      1,220                                    1,240                                   1,260
                                        |                                        |                                       |
Ancestral_G7_Beta  CCAGATCAATACTGCAGCTAAAGAAGCTTGGAAATCCCTACCTTGTAGTAACAGAAAGAC  1260
    scaffold_12699  CCAGATCAATACTGCAGCTAAAGAAGCTTGGAAATCCCTACCTGGTAGTAACAGAAAGAC  1260
     scaffold_2273  CCAGATCAATACTGCGGCTAAAGAAGCTTGGAAATCCCTACCTAATGGTAACAGAAAGAC  1260
GeneScaffold_1344  CCAGATCAATACTGCAGCTAAAGAAGCTTGGAAATCTCTACCTTGTAGTAACAGAAAGAC  1260
     scaffold_9648  CCAGATCAATACTGCAGCTAAAGAAGCTTGGAAATCTCTACCTTGTAGTAACAGAAAGAC  1260
    scaffold_17393  CCAAATCAATACTGCAGCTAAAGAAGCTTGGAAATCTCTACCTTGTAGTAACAGAAAGAC  1260
    scaffold_12793  CCAGATCAATACTGCAGCTAAAGAAGCTTGGAAATCCCTACCTTGTAGTAACAGAAAGAC  1260
```

```
                                       1,280                            1,300                          1,320
                                         |                                |                              |
Ancestral_G7_Beta  TGAAGAATTATCTAAGATTAGGCAAGGACCTGATGAGCCTTTTCAAGATTTTGTTGATAG 1320
   scaffold_12699  TGAAGAATTATCTAAGATTAGGCAAGGACCTGATGAGCCTTTTCAAGATTTTGTTGATAG 1320
    scaffold_2273  TGAAGAATTATCTAAGATTAGGCAAGGACCTGATGAGCCTTTTCAAGATTTTGTTGATAG 1320
GeneScaffold_1344  TGAAGAATTATCTAAGATTAGGCAAGGACCTGATGAGCCTTTTCAAGATTTTGTTGATAG 1320
    scaffold_9648  TGAAGAATTATCTAAGATTAGGCAAGGACCTGATGAGCCTTTTCAAGATTTTGTTGATAG 1320
   scaffold_17393  TGAAGAATTATCTAAGATTAGGCAAGGACCTGATGAGCCTTTTCAAGATTTTGTTGATAG 1320
   scaffold_12793  TGAAGAATTATCTAAGATTAGGCAAGGACCTGATGAGCCTTTTCAAGATTTTGTTGATAG 1320
                                       1,340                            1,360                          1,380
                                         |                                |                              |
Ancestral_G7_Beta  ATTGTTGAATGCAGCGGTCGGCTTATCAGTGATCCCGAGGCCGAAACTATTCTAGTAAAA 1380
   scaffold_12699  ATTGTTGAATGCAGCGGTCGGCTTACCAGTGATCCTGAGGCCGAAACTATTCTAGTAAAA 1380
    scaffold_2273  ATTGTTGAATGCAGTGGTCGGCTTATCAGTGATCCCGAGGCCAAAACTATTCTAGTAAAA 1380
GeneScaffold_1344  ATTGTTGAATGCAGCGGTCGGCTTATCAGTGATCCCGAGGCCGAAACTATTCTAGTAAAA 1380
    scaffold_9648  ATTGTTTAATGCAGCGGTCGGCTTATCAGTGATCCCGAGGCCGAAACTATTCTAGTAAAA 1380
   scaffold_17393  ATTGTTGAATGCAGCGGTCGGCTTATCAGTGATCCCGAGGCCGAAACTATTCTAGTAAAA 1380
   scaffold_12793  ATTGTTGAATGCAGCGGTCGGCTTATCAGTGATCCCGAGGCCGAAACTATTCTAGTAAAA 1380
                                       1,400                            1,420                          1,440
                                         |                                |                              |
Ancestral_G7_Beta  ATATTAGCTTATGAGAATGCTAATTCTGCTTGTCAGGCCGCAATTAGACCTTTTAAAAGG 1440
   scaffold_12699  CTACTAGCTTATGAGAATGCTAATTCTGCTTGTCAGGCCGCAATTAGACCTTTTAAAAAG 1440
    scaffold_2273  ATATTAGCTTATGAGAATGCTAATTCTGCTTGTCAGGCCGCAATTAGACCTTTTAAAAAG 1440
GeneScaffold_1344  ATATTAGCATATGAGAATGCTAATTCTGCTTGTCAGGCCGCAATTAGACCTTTTAAAAAG 1440
    scaffold_9648  ATATTAGCATATGAGAATGCTAATTCTGCTTGTCAGGCCGCAATTAGACCTTTTAAAAAG 1440
   scaffold_17393  ATATTAGCATATGAGAATGCTAATTCTGCTTGTCAGGCCGCAATTAGACCTTTTAAAAGG 1440
   scaffold_12793  ATATTAGCTTATGAGAATGCTAATTCTGCTTGTCAGGCCACAATTAGACCTTTTAAAAGG 1440
                                       1,460                            1,480                          1,500
                                         |                                |                              |
Ancestral_G7_Beta  AAAGGGGATCTTGGAGATTACATCCGACTTTGCTCGGACATTGGGCCCTCTTATACCCAA 1500
   scaffold_12699  AAAGGGGATCTTGGAGATTACATCCGACTTTGCTCGGACATTGGGCCCTCTTATACCCAA 1500
    scaffold_2273  AAAAGGGATCTTGGAGATTACATCCGACTTTGCTCGGACATTGGGCCTTCTTATACCCAA 1500
GeneScaffold_1344  AAAGGGGATCTTAAAGATTACATCCGACTTTGCTCGGACATTGGGCCCTCTTATACCCAG 1500
    scaffold_9648  AAAGGAGATCTTAAAGATTACATCCGACTTTGCTCGGACATTGGGCCCTCTTATACCCAG 1500
   scaffold_17393  AAAGGGGATCTTAGAGATTACATCCGACTTTGCTCGGACATTGGGCCCTCTTATACCCAA 1500
   scaffold_12793  AAAGGGGATCTTGGAGATTACATCCGACTTTGCTCGGACATTGGGCCCTCTTATACCCAA 1500
                                       1,520                            1,540                          1,560
                                         |                                |                              |
Ancestral_G7_Beta  GGAATAGCCATTGCTGCAGCACTGCAAGGAAAATCTATTAAGCAAGTATTAACAGCAAGC 1560
   scaffold_12699  GGAATAGCCATTGCTGCAGCACTGCAAGGAAAATCTATTAAGCAAGTATTAACAGCAAGC 1560
    scaffold_2273  GGAATAGCCATTGCTGCAGCACTGCAAGGAAAATCTATTAAGCAAGTATTAACAGCAAGC 1560
GeneScaffold_1344  GGAATAGCCATTGCTGCAGCACTGCAAGGAAAATCTATTAAGCAAGTATTAACAGCAAGC 1560
    scaffold_9648  GGAATAGCCATTGCTGCAGCACTGCAAGGAAAATCTATTAAGCAAGTATTAACAGCAAAC 1560
   scaffold_17393  GGAATAGCCATTGCTGCAGCACTGCAAGGAAAATCTATTAAGCAAGTATTAACAGCAAGC 1560
   scaffold_12793  GGAATAGCCATTGCTGCAGCACTGCAAGGAAAATCTATTAAGCAAGTATTAACAGCAAGC 1560
                                       1,580                            1,600                          1,620
                                         |                                |                              |
Ancestral_G7_Beta  AAAATATGGGAAAGGAAGAGGGATTAATGCTGGTTGCTTTGGATGTGGAAAATCAGGCCA 1620
   scaffold_12699  AAAATATGGGAAAGGAAGAGGGATTAATGCTGGTTGCTTTGGATGTGGAAAATCAGGCCA 1620
    scaffold_2273  AAAATATAGGAAAGAAAGAGGGATTAATGCTGGTTACTTTGGATGTGGAAAATCTGGCCA 1620
GeneScaffold_1344  AAAATATAGGAAAGGAAGAGGGATTAATGCTGGTTGCTTTGGATGTGGAAAATCAGGCCA 1620
    scaffold_9648  AAAATATGGGAAAGGAAGAGGGATTAATGCTGGTTGCTTTGGATGTGGAAAATCAGGCCA 1620
   scaffold_17393  AAAATATGGGAAAGGAAGAGGGATTAATGCTGGTTGCTTTGGATGTGGAGAATCAGGCCA 1620
   scaffold_12793  AAAATATGGGAAAGGAAGAGGGATTAATGCTGGTTGCTTTGGATGTGGAAAATCAGGCCA 1620
                                       1,640                            1,660                          1,680
                                         |                                |                              |
Ancestral_G7_Beta  TCAGATTACAGTGTCCCACTAGGGGCAATATCCCAAGGGTGCCTGGAGTGTGTCCGAAAT 1680
   scaffold_12699  TCAGATTACAGTGTCCCACTAGGGGCAATATCCCAAGGGCGCCTGGAGTGTGTCCGAAAT 1680
    scaffold_2273  TCAGATTACAGTATCCCACTAGGGGCAATATCCCAAGGGCGCCTGGAGTGTGTCCGAAAT 1680
GeneScaffold_1344  TCAGATTACAGTGTCCCACTAGGGGCAATGTCCCAAGGGTGCCTGAAGTGTGTCCAAAAT 1680
    scaffold_9648  TCAGATTACAGTGTCCCACTAGGGGCAATGTCCCAAGGGTGCCTGAAGTGTGTCCAAAAT 1680
   scaffold_17393  TCAGATTACAGTGTCCCACTAGGGGCAATATCCCAAGGGTGCCTGGAGTGTGTCCAAAAT 1680
   scaffold_12793  TCAGATTACAGTGTCCCACTAGGGGCAATATCCCAAGGGTGCCTGGAGTGTGTCCAAAAT 1680
```

```
                                       1,700               1,720               1,740
Ancestral_G7_Beta   GTGGAAAAGGAAAGCATTGGGCACGAGATTGTAAGTCTAAGACCAATGTGGCGGGCCAAT 1740
   scaffold_12699   GTGGAAAAGGAAAGCATTGGGCACGAGATTGTAAGTCTAAGACCAATGTGGCGGGCCAAA 1740
    scaffold_2273   GTGGAAAAGGAAAGCATTGGGCACGAGATTGTAAGTCTAAGACCAACGTGGCGGGCCAAT 1740
GeneScaffold_1344   GTAAAAAAGGAAAGCATTGGGCACGAGATTGTAAGTCTAAGACCAATGTAGCGGGCCAAT 1740
    scaffold_9648   GTAGAAAAGGAAAGCATTGGGCACGAGGTTGTAAGTCTAAGACCAATGTAGCGGGCCAAT 1740
   scaffold_17393   GTGGAAAAGGAAAGCATTGGGCACGAGATTGTAAGTCTAAGACCAATGTAGTGGGCCAAT 1740
   scaffold_12793   GTGGAAAAGGAAAGCATTGGGCACAAGATTGTAAGTCTAAGACCAATGTAGCAGGCCAAT 1740
                                       1,760               1,780               1,800
Ancestral_G7_Beta   TTATTCAGGGAAACGGATGGAGGGGCCCGCCCCGGGCCCCTGCACAATGTTACGGGGCAA 1800
   scaffold_12699   CTATTCAGGGAAACGGATGGAGGGGCCCGCCCCGGGCCCCTGCACAATGTTACAGGGCAA 1800
    scaffold_2273   CTATTCAGGGAAACGGATGGAGGGGCCCGCCCCGGGCCCCTGCACAATGTTACGGGGCAA 1800
GeneScaffold_1344   TTATTCAGGGAAACGGATGGAGGGGCCCGCCCCGGGCCCCTGCACAATGTTACGGGGCAA 1800
    scaffold_9648   TTATTCAGGGAAACGGATGGAGGGGCCCGCCCCGGGCCCCTGCACAATGTTACGGGGCAA 1800
   scaffold_17393   TTAGTCAGGGAAACGGACGGAGGGGCCCGCCCCGGGCCCCTGCACAATGTTACGAGGCAA 1800
   scaffold_12793   TTATTCAGGGAAACGGATGGAGGGGCCCGCCCCGGGCCCCTGCACAATGTTACGGGGCAA 1800
                                       1,820               1,840               1,860
Ancestral_G7_Beta   CTCAGCAGGCCCAGGGAGGTCTGTTCAGAACTTTTCCGAGCAACCTCAGGCAGCGCAGGA 1860
   scaffold_12699   CTCAGCAGGCCCAGGGAGGTCTGTTCAGAACTTTTCCGAGCAACCTCAGGCAGCGCAGGA 1860
    scaffold_2273   CTCAACAGGCCCAGGGAGGTCTGTTCAGATCTTTTCCGGGCAACCTCAGGCAGCGCAGGA 1860
GeneScaffold_1344   CTCAGCAGGCCCAGGGAGGTCTGTTCAGAACTTTTCCGAGCAACCTCAGGCAGCGCAGGA 1860
    scaffold_9648   CTCAGCAGGCCCAGGGAGGTCTGTTCAGAACTTTTCTGAGCAACCTCAGGCAGCGCAGGA 1860
   scaffold_17393   CTCAGCAGGCCCAGGGAGGTCTGTTCAGAACTTTTCCGAGCAACCTCAGGCAGCGCAGGA 1860
   scaffold_12793   CTCAGCAGACCCAGGGAGGTCTGTTCAGAATTTTTCCGAGCAACCTCAGGCAGCGCAGGA 1860
                                       1,880               1,900               1,920
Ancestral_G7_Beta   CTGGACCTCAGTTCCTCCACCTACGCAGTCTTAACCCCTGAAATGGGAATTCAAGCCCTA 1920
   scaffold_12699   CTGGACCTCAGTTCCTCCACCTACGCAGTCTTAACTCTTGAAATGGGAATTCAAGCCCTA 1920
    scaffold_2273   CTGGACCTCAGTTCCTCCACCTACGCAGTCTTAACCCCTGAAATGGGAATTCAAGCCCTA 1920
GeneScaffold_1344   CTGGACCTCAGTTCCTCCACCTACGCAGTTTTAACCCCTGAAATGGGAATTCAAGCCCTA 1920
    scaffold_9648   CTGGACCTCAGTTCCTCCACCTACGCAGTTTTAACCCCTGAAATGGGAATTCAAGCCCTA 1920
   scaffold_17393   CTGGACCTCAGTTCCTCCACCTACGCAGTTTTAACCCCTGAAATGGGAATTCAAGCCCTA 1920
   scaffold_12793   CTGGACCTCAGTTCCTCCACCTACGCAGTCTTAACCCCTGAAATGGGAATTCAAGCCCTA 1920
                                       1,940               1,960               1,980
Ancestral_G7_Beta   CCTACAGGAGTGTGGGGGCCTCTCCCATCAGGAACATTAGGATTGCTGTTAGGAAGAAGT 1980
   scaffold_12699   CCTACAGGAGTGTGGGGGCCTCTCCCATCAGGAACATTAGGATTGCTGTTAGGAAGAAGT 1980
    scaffold_2273   CCTACAGGAGTGTGGGGGCCTCTCCCATTAGGAACATTAGGATTGCTATTAGGAAGAAGT 1980
GeneScaffold_1344   CCTACAGGAGTGTGGGGGCCTCTCCCATCAAGAACATTAGGATTGCTGTTAGGAAGAAGT 1980
    scaffold_9648   CCTACAGGAGTGTGGGGGCCTCTCCCATCAGGAACATTAGGATTGCTGTTAGGAAGAAGT 1980
   scaffold_17393   CCTACAGGAGTGTGGGGGCCTCTCCCATCAGGAACATTAGGATTGCTGTTAGGAAGAAAT 1980
   scaffold_12793   CCTACAGGAGTGTGGGGGCCTCTCCCATCAGGAACATTAGGATTGCTGTTAGGAAGAAGT 1980
                                       2,000               2,020               2,040
Ancestral_G7_Beta   AGTACTACCATGCAAGGAATTATTGTTCATCCTGGCATAATTGATGCTGATTACACAGGA 2040
   scaffold_12699   AGTACTACCATGCAAGGAATTATTGTTCATCCTGGCATAATTGATGCTGATTACACAGGA 2040
    scaffold_2273   AGTACTACCATGCAAGGAATTATTGTTCATCCTGGCATAATTGATGCTGATCACACAGGA 2040
GeneScaffold_1344   AGTACTACCATGCAAGGAATTATTGTTCATCCTGGCATAATTAATGCTGATTACACAGGA 2040
    scaffold_9648   AGTACTACCATGCAAGGAATTATTGTTCATCCTGGCATAATTAATGCTGATTACACAGGA 2040
   scaffold_17393   AGTACTACCATGCAAGGAATTATTGTTCATCCTGGCATAATTAATGCTGATTACACAGGA 2040
   scaffold_12793   AGTACTACCATGCAAGGAATTATTGTTCATCCTGGCATAATTGATGCTGATTACACAGGA 2040
                                       2,060               2,080               2,100
Ancestral_G7_Beta   GAAATAAAAATTATGACTGGGTCCCCTTCTAAAATTTCTGCAATTCAAGCGGGACAACGA 2100
   scaffold_12699   GAAATAAAAATTTTGACTGGGTCCCCTTCTAAAATTTCTGCAATTCAAGCGGGACAGCGA 2100
    scaffold_2273   GAAATAAAAATTATGACTGGGTCCCCTTCTAAAATTTCTGCAATTCAAGCAGGACAACGA 2100
GeneScaffold_1344   GAAATAAAAATTATGACTGGGTCCCCTTCTAAAATTTCTGCAATTCAAGCGGGACAACGA 2100
    scaffold_9648   GAAATAAAAATTATGACTGGGTCCCCTTCTAAAATTTCTGTAATTCAAGCGGGACAACGA 2100
   scaffold_17393   GAAATAAAAATTATGACTGGGTCCCCTTCTAAAATTTCTGCAATTCAAGCGGGACAACGA 2100
   scaffold_12793   GAAATAAAAATTATGACTGGGTCCCCTCCTAAAATTTCTGCAATTCAAGCGGGACAACGA 2100
```

```
                                                   2,120                                    2,140                                    2,160
                                                     |                                        |                                        |
Ancestral_G7_Beta  ATAGCACAGCTTATTATAATCCAATTGGTCATGCTAAGAGTAGTAAACAAAGGGGTACAC  2160
    scaffold_12699  ATAGCACAGCTTATTATAATCCAATTGGTCATGCTAAGAGTAGTAAACTAAGGGGTACAC  2160
     scaffold_2273  ATAGCACAGCTTATTATAATCCAATTGGTCATGCTAAGAGTAGTAAGCAAAGGGGTACAC  2160
GeneScaffold_1344  ATAGCACAGCTTATTATAATCCAATTGGTCATGCTAAGAGTAGTAAACTAAGGGGTACAC  2160
     scaffold_9648  ATAGCACAGCTTATTATAATCCAATTGGTCATGCTAAGAGTAGTAAACTAAGGGGTACAC  2160
    scaffold_17393  ATAGCACAGCTTATTATAATCCAATTGGTCATGCTAAGAGTAGTAAACTAAGGGGTACAC  2160
    scaffold_12793  ATAGCACAGCTTATTATAATCCAATTGGTCATGCTAAGAGTAGTAAACAAAGGGGTACAC  2160

                                                   2,180                                    2,200                                    2,220
                                                     |                                        |                                        |
Ancestral_G7_Beta  AAGGTTTTGGCTCATCTGATCTCTATTGGATCCAAGCTATTGGGTCAAAGAGACCCGAAC  2220
    scaffold_12699  AAGGTTTTGGCTCATCTGATCTCTATTGGATCCAAGCTATTGGGTCAAAGAGACCCGAAC  2220
     scaffold_2273  AAGGTTTTGGCTCATCTGATCTCTATTGGATCCAAGCTATTGGGTCAAAGAAGCCCAAAC  2220
GeneScaffold_1344  AAGGTTTTGGCTCATCTGATCTCTATTGGATCCAAGCTATTGGGCAAAAGAGACCCAAAC  2220
     scaffold_9648  AAGGTTTTGGCTCATCTGATCTCTATTGGATCCAAGCTATTGGGCAAAAGAGACCTGAAC  2220
    scaffold_17393  AAGGTTTTGGCTCATCTGATCTCTATTGGATCCAAGCTATTGGGTCAAAGAGACCCGAAC  2220
    scaffold_12793  AAGGTTTTGGCTCATCTGATCTCTATTGGATCCAAGCTATTGGGTCAAAGCGACCCGAAC  2220

                                                   2,240                                    2,260                                    2,280
                                                     |                                        |                                        |
Ancestral_G7_Beta  TCAAGTTAAAAATTAGAGGAAAAGAATTCATAGGGTTACTAGATACAGGAGCTGATGTAT  2280
    scaffold_12699  TCAAGTTAAAAATTTGAGGAAAAGAATTCATAGGGTTACTAGAGCTTTGGACTTTGATTA  2280
     scaffold_2273  TCAAGTTAAAAATTAGAGGAAAAGAATTCATAGGGTTACTAGATATAGGAGATGATGTAT  2280
GeneScaffold_1344  TCAAGTTAAAAATTAGAGGAAAAGAATTCATAGGGTTACTAGATACAGGAGCTGATGTAT  2280
     scaffold_9648  TCAAATTAAAAATTAAAGGAAAAGAATTCATAGGGTTACTAGACACAGGAGCTGATGTAT  2280
    scaffold_17393  TCAAGTTAAAAATTTGAGGAAAAGAATTCTTAGGGTTACTAGATACAGGAGCTGATGTAT  2280
    scaffold_12793  TCAAGTTAAAAATTAGAGGAAAAGAATTCATAGGGTTACTAGATACAGGAGCTGATATAT  2280

                                                   2,300                                    2,320                                    2,340
                                                     |                                        |                                        |
Ancestral_G7_Beta  CTGTTGCTCTGCAGCATTGGCCCCACAGTGGCCTAAACAACCTACTATGACACAGTTGCA  2340
    scaffold_12699  GAGATTCTTTAGACCCTAGGCATGAAAGAGATCAGATTAAAGAACAATGTCACCGTTGCA  2340
     scaffold_2273  CTGTTGCTCTGCAGCATTGGCCCCACAGTGGCCTAAACAACCTACTATGACACAGTTGCA  2340
GeneScaffold_1344  CTGTTGCTCTGCAGCATTGGCCCCACAGTGGCCTAAACAACCTACTATGACACAGTTGCA  2340
     scaffold_9648  CTGTTGCTCTGCAGCATTGGCCCCACAGTGGCCTAAACAACCTACTATGACACAGTTGCA  2340
    scaffold_17393  CTGTTGCTCTGCAGCATTGGCCCCACAGTGGCCTAAACAACCTACTATGACACAGTTGCA  2340
    scaffold_12793  CTGTTGCTCTGCAGCATTGGCCCCACAGTGGCCTAAACAACCTACTATGACACAGTTGCA  2340

                                                   2,360                                    2,380                                    2,400
                                                     |                                        |                                        |
Ancestral_G7_Beta  GGGAATAGGGCAGCAAACCACTCCTGAGCAAAGTAGTGATTTATTAACCTGGGAAGATCA  2400
    scaffold_12699  GGGAATAGGGCAGCAAACCACTCCTGAGCAAAGTAGTGATTTATTAACCTGGGAAGATCA  2400
     scaffold_2273  GGGAATAGGGCAGCAAACCACTCCTGAGCAAAGTAGTGATTTATTAACCTGGGAAGATCA  2400
GeneScaffold_1344  GGGAATAGGGCAGCAAACCACTCCTGAGCAAAGTAGTGATTTATTAACCTGGGAAGACCA  2400
     scaffold_9648  GGGAATAGGGCAACAAACCACTCCTGAGCAAAGTAGTGATTTATTAACCTGGGAAGATCA  2400
    scaffold_17393  GGGAATAGGGCAGCAAACCACTCCTGAGCAAAGTAGTGATTTATTAACCTGGGAAGATCA  2400
    scaffold_12793  GGGAATAGGGCAGCAAACCACTCCTGAGCAAAGTAGTGATTTATTAACCTGGGAAGATCA  2400

                                                   2,420                                    2,440                                    2,460
                                                     |                                        |                                        |
Ancestral_G7_Beta  AGATAACAATAAAGGGACGTTCCAACCTTATATTGTTCCAGGATTACCTGTTAACCTCTG  2460
    scaffold_12699  AGATAACAATAAAGGGATGTTCCAACCTTATATTGTTCCAGGATTACCTGTTAACCTCTG  2460
     scaffold_2273  AGATAACAATAAAGGGACGTTCCAACCTTATATTGTTCCAGGATTACCTGTTAACCTCTG  2460
GeneScaffold_1344  AGATAACAATAAAGGGACATTCCAACCTTATTTTGTTCCAGGATTACCTGTTACCCTCTG  2460
     scaffold_9648  AGATAACAATAAAGGAACATTCCAACCTTATATTGTTCCAGGATTACCTGTTAACCTCTG  2460
    scaffold_17393  AGATAACAATAAAGGGACGTTCCAACCTTATATTGTTCCAGGATTACCTGTTAACCTCTG  2460
    scaffold_12793  AGATAACAATAAAGGGACGTTCCAACCTTATATTGTTCCAGGATTACCTGTTAACCTCTG  2460

                                                   2,480                                    2,500                                    2,520
                                                     |                                        |                                        |
Ancestral_G7_Beta  GGGAAGAGATATTATGGAAAAGATGGGAGTATATCTCCACAGCCCAAATAAGATAATTAC  2520
    scaffold_12699  GGGAAGAGATATTATGGAAAAGATGGGAGTATATCTCCACAGCCCAAATAAGATAATTAC  2520
     scaffold_2273  GGGAAGAGATATTATGGAAAAGATGGGAGTATATCTCCACAGCCCAAATAAGATAATTAC  2520
GeneScaffold_1344  GGGAAGAGATATTATGGAAAAGATGGGAGTATATCTCCACAGCCCAAATAAGAAAATTAC  2520
     scaffold_9648  GGGAAGAGATATTATGGAAAAGATGGGAGTATATCTCCATAGCCCAAATAAGATAATTGC  2520
    scaffold_17393  GGGAAGAGATATTATGGAAAAGATGGGAGTATATCTCCACAACCCAAATAAGATAATTAC  2520
    scaffold_12793  GGGAAGAGATATTATGGAAAAGATGGGAGTATATCTCCACAGCCCAAATAAGATAATTAC  2520
```

	2,540 2,560 2,580	
Ancestral_G7_Beta	TCAACAAATGTTTGATCAGGGACAACTCCCATTTCAGGGGATGGGGAGATATAATCAAGG	2580
scaffold_12699	TCAACAAATGTTTGATCAAGGACAACTCCCATTTCAGGGGATGGGGAGATGTAATCAAGG	2580
scaffold_2273	TCAACAAATGTTTGATCAGGGACAACTCCCATTTCAGGGGATGGGGAGATATAATCAAGG	2580
GeneScaffold_1344	TCAACAAATGTTTGATCAGGGACAACTCCCATTTCAGGGGATGGGGAGATATAATCAAGG	2580
scaffold_9648	TCAACAAATGTTTGATCAGGGACAGCTCCCATTTCAGGGGATGGGGAGATATAATCAAGG	2580
scaffold_17393	TCAACAAATGTTTGATCAGGGACAGCTCCCATTTCAGGGGATGGGGAGATATAATCAAGG	2580
scaffold_12793	TCAACAAATGTTTGATCAGGGACAACTCCCATTTCAGGGGATGGGGAGTTATAATCAAGG	2580
	2,600 2,620 2,640	
Ancestral_G7_Beta	AATAATTAACCCTTTGCAGCCTAATTGACATTGAGCAGGGCTTGGATATTTTTGACAAGG	2640
scaffold_12699	AATAATTAACCCTTTGCAGCCTAATTGACATCGAGCAGGGCTTGGATATTTTTGACAAGG	2640
scaffold_2273	AATAATTAACCCTTTGCAGCCTAATTGACATTGAGCAGGGCTTGGATAGTTTTGACAAGG	2640
GeneScaffold_1344	AATAATTAACTCTTTGCAGCCTAATTGACATCGAGCAGGGCTTGGATATTTTTAACAGGG	2640
scaffold_9648	AATAATTAACCCTTTGCAGCCTAATTGACATCGAGCAGGGCTTGTATATTTTTAACAAGG	2640
scaffold_17393	AATAATTAACCCTTTGCAGCCTAATTGACATCGAGCAGGGCTTGGATATTTTTGACAAGG	2640
scaffold_12793	AATAATTAACCCTTTGCAGCCTAATTGACATTGAGCAGGGCTTGGATATTTTTGACAAGG	2640
	2,660 2,680 2,700	
Ancestral_G7_Beta	GTCATTGAACAACCTGTACCCCATGCGGATCCAATAACCTGGAAAGCGAGGATCCTGTAT	2700
scaffold_12699	GTCATTGAACAACCTGTACCCCATGCGGATCCAATAACCTGGAAAGCGAGGATCCTGTAT	2700
scaffold_2273	GTCATTGAACAACCTGTACCCCATGTGGATCCAATAACCTGGAAAGCGAGGATCCTGTGT	2700
GeneScaffold_1344	GTCATTGAACAACCTGTACCCCATGCGGATCCAATAACCTGGAAAGCGAGGATCCTGTAT	2700
scaffold_9648	GTCATTGAACAACCTGTACCCCATGCGGATCCAATAACCTGGAAAGCGAGGATCCTGTAT	2700
scaffold_17393	GTCATTGAACAGCCTGTATCCCATGCGGATCCAATAACCTGGAAAGCGAGGATCCTGTAT	2700
scaffold_12793	GTCATTGAACAACCTGTACCCCATGCGGATCCAATAATCTGAAAAGCGAGGATCCTGTAT	2700
	2,720 2,740 2,760	
Ancestral_G7_Beta	GGGTGGATCAGTGGCCCTTGACTGAGGAAAAAATACAAGCCACTCGGCAGTTAGTACAGG	2760
scaffold_12699	GGGTGGATCAGTGGCCCTTGACTGAGGAAAAAATATAAGCCACTCGGCAGTTAGTACAGG	2760
scaffold_2273	GGGTGAATCAGTGGCCCTTGACTGAGAAAAAAATGCAAGCCACTCGGCAGTTAGTACAGG	2760
GeneScaffold_1344	GGGTGGATCAGTGGCCCTTGACTGAGGAAAAAATACAAGCCGCTCAGCAGTTAGTACAGG	2760
scaffold_9648	GGGTGGATCAGTGGCCCTTGACTGAGGAAAAAATACAAGCCGCTCAGCAGTTAGTACAGG	2760
scaffold_17393	GGGTGGATCAGTGGCCCTTGACTGAGGAAAAACTACAAGCCGCTCAGCAGTTAGTACAGG	2760
scaffold_12793	GGGTGGATCAGTGGCCCTTGACTGAGGAAAAAATACAAGCCTCTCGGCAGTTAGTACAGG	2760
	2,780 2,800 2,820	
Ancestral_G7_Beta	AACAGCTGGAGAGCGGACATATTGAGGAATCTAATTCCCCTTGGAATTCTCCTATATTTG	2820
scaffold_12699	AACAGCTGGAGAGCGGACATATTGAGGAATCTAATTCCCCTTGGAATTCTCCTATATTTG	2820
scaffold_2273	AACAGCTGGAGAGCGGACATATTGAGGAATCTAATTCCCCTTGGAATTCTCCTATATTTG	2820
GeneScaffold_1344	AACAGCTAGAGAGCGGACATATTGAGGAATCTAATTCCCCTTGGAATTCTCCTATATTTG	2820
scaffold_9648	AACAGCTAGAGAGCGGACATATTGAGGAATCTAATTCCCCTTGGAATTCTCCTATATTTG	2820
scaffold_17393	AACAGCTGGAGAGCAGACATATCGAGGAATCTAATTCCCTTTGGAATTCTCCTATATTTG	2820
scaffold_12793	AACAGCTGGAGAGCGGACATATTGAGGAATCTAATTCCCCTTGGAATTCTCCTATATTTG	2820
	2,840 2,860 2,880	
Ancestral_G7_Beta	TAATAAGAAAAAATCAGGAAAATGGAGACTCTTACAAGATTTAAGGGCTGTAAACCAGAC	2880
scaffold_12699	TAATAAAAAAAAATCAGGAAAATGGAGACTCTTACAAGATTTAAGGGCTGTAAACCAGAC	2880
scaffold_2273	TAATAAGAAAAAATCAGGAAAATGGAGACTCTTACAAGATTTAAGGGCTGTAAACCAGAC	2880
GeneScaffold_1344	TAATAAGAAAAAATCAGGAAAATGGAGACTCTTACAAGATTTAAGGGCTGTAAACCAGAC	2880
scaffold_9648	TAATAAGAAAAAATCAGGAAAATGGAGACTCTTACAAGATTTAAGGGCTGTAAACCAGAC	2880
scaffold_17393	TAATAAGAAAAAATCAGGAAAATGGAGACTCTTACAAGATTTAAGGGCTGTAAACCAGAC	2880
scaffold_12793	TAATAAAAAAAAATCAGGAAAATGGAGACTCTTACAAGATTTAAGGGCTGTAAACCAGAC	2880
	2,900 2,920 2,940	
Ancestral_G7_Beta	AATGGAACTAATGGGAGCATTGCAGCCTGGGTTGCCTTCTCCGGCTGCCATTCCTAAAGA	2940
scaffold_12699	AATGGAACTAATGGGAGCATTGCAGCCTGGATTGCCTTCTCCGGCTGCCATTCCTAAAGA	2940
scaffold_2273	AATGGAACTAATGGGAGCATTGCAGCCTGGGTTGCCTTCTCCGGCTGCCATTCCTAAAGA	2940
GeneScaffold_1344	AATGGAACTAATGGGAGCATTGCAGCCTGGGTTGCCTTCTCCGGCTGCCATTCCTAAAGA	2940
scaffold_9648	AATGGGACTAATGGGAGCATTGCAGCCTGGGTTGCCTTCTCCGGCTGCCATTCCTAAAGA	2940
scaffold_17393	AATGGAACCAATGGGAGCATTGCAGCCTGGGTTGCCTTCTCCGGCTGCCATTCCTAAAGA	2940
scaffold_12793	AATGGAACTAATGCGAGCATTGCAGCTTGGGTTGCCTTCTCCGGCTGCCATTCCTAAAGA	2940

```
                                      2,960                      2,980                      3,000
Ancestral_G7_Beta   TACTTATAAGATCATTATTGATTTGAAAGATTGTTTTTATACTGTTCCCTTATGCCCTGT 3000
    scaffold_12699  TACTTATAAGATCATTATTGATTTGAAAGATCGTTTTTATACTGTTCCCTTATGCCCCGT 3000
     scaffold_2273  TACTTATAAAATCATTATTGATTTGAAAGATTGTTTTTATACTGTTCCCTTATGCCCTGT 3000
GeneScaffold_1344   TACTTATAAGATCATTATTGATTTGAAAGATTGTTTTTATACTGTTCCCTTATGCCCTGT 3000
     scaffold_9648  TACTTATAAGATCATTATTGATTTGAAAGATTGTTTTTATACTGTTCCCTTATGCCCTGT 3000
    scaffold_17393  TACTTATAAGATCATTATTGATTTGAAAGATTGTTTTTATACTGTTCCCTTATGCCCTGT 3000
    scaffold_12793  TACTTATAAGATCATTATTGATTTGAAAGATTGTTTTTATACTTTTCCCTTATGCCCTGT 3000

                                      3,020                      3,040                      3,060
Ancestral_G7_Beta   TGATTGTAAGCGGTTTGCCTTCAGTGTGCCTTCATCTAATTTTAAAGAACCTATGACAAG 3060
    scaffold_12699  TGATTGTAAGCGGTTTGCCTTCAGTGTGCCTTCATCTAATTTTAAAAAACCAATGACAAG 3060
     scaffold_2273  TGATTGTAAGCGGTTTGCCTTCAGTGTGCCTTCATCTAATTTTAAAGAACCTATGACAAG 3060
GeneScaffold_1344   TGATTGTAAGCGGTTTGCCTTCAGTGTGCCTTCATCTAATTTTAAAAAACCAATGACAAG 3060
     scaffold_9648  TGATTGTAAGTGGTTTGCCTTCAGTGTGCCTTCATCTAATTTTAAAAAACCAATGACAAG 3060
    scaffold_17393  TGATTGTAAGCGGTTTGCCTTCAGTGTGCCTTCATCTAATTTTAAAAAACCAATGACAAG 3060
    scaffold_12793  TGATTGTAAGCGGTTTGCCTTCAGTGTGCCTTCATCTAATTTTAAAGAACCTATGACAAG 3060

                                      3,080                      3,100                      3,120
Ancestral_G7_Beta   GTATCACTGGAAAGTTTTGCCACAAGGCATGGCCAATAGTCCTACACTATGTCAAAAATT 3120
    scaffold_12699  GTATCACTGGAAAGTTTTGCCACAAGGCATGGCCAAGAGTCCTACACTATGTCAAAAATT 3120
     scaffold_2273  GTATCACTGGAAAGTTTTGCCACAAGGCATGGCCAATAGTCCTATACTATGTCAAGAATT 3120
GeneScaffold_1344   GTATCACTGGAAAGTTTTGCCACAAGGCATGGCCAATAGTCCTACACTATGTCAAAAATT 3120
     scaffold_9648  GTATCACTGGAAAGTTTTGCCACAAGGCATGGCCAATAGTCCTACACTATGTCAAAAATT 3120
    scaffold_17393  GTATCACTGGAAAGTTTTGCCACAAGGCATGGCCAATAGTCCTATACTATGTCAAAAATT 3120
    scaffold_12793  GTATCACTGGAAAGTTTTGCCACAAGACATGGCCAATAGTCCTACACTATGTCAAAAATT 3120

                                      3,140                      3,160                      3,180
Ancestral_G7_Beta   TGTGGCTCAAGCATTGGCTTCCATACGGAAAAATTTTCAACAGTATATTTAATTCATTAT 3180
    scaffold_12699  TGTGGCTCGAGCATTGGCTTCCATACGGAAAAATTTTCAACAGTATATTTAATTCATTAT 3180
     scaffold_2273  TGTGGCTCAAGCATTGGCTTCCATACGGAAAAATTTTCAACAGTATATTTAATTCATTAT 3180
GeneScaffold_1344   TGTGGCTCAAGCATTGGCTTCCATACGGAAAAATTTTCAACAGTCTATTTAATTCATTAT 3180
     scaffold_9648  TGTGGCTCAAGCATTCGCTTCCATACGGAAAAATTTTCAACAGTCTATTTAATTCATTAT 3180
    scaffold_17393  TGTGGCTCAAGCATTGGCTTCCATACGGAAAAATTTTCAACAGTCTATTTAATTCATTAT 3180
    scaffold_12793  TGTGGCTCAAGCATTGGCTTCCATACGGAAAAATTTTCAACAGTATATTTAATTCATTAT 3180

                                      3,200                      3,220                      3,240
Ancestral_G7_Beta   ATGGATGATATCCTGTTAGCTCATAAAAACGAGCCTGCACTTTTACAGTGCTATTCAATT 3240
    scaffold_12699  ATGGATGATATCCTGTTAGCTCATAAAAACGAGCCTGCACTTTTACAGTGCTATTCAATT 3240
     scaffold_2273  ATGGATGATATCCTGTTAGCTCATAAAAACAAGCCTGCACTTTTACAGTGCTATTCAATT 3240
GeneScaffold_1344   ATGGATGATATCCTGTTAGCTCATAAAAACGAGCCTGCACTTTTACAGTGCTATTCAATT 3240
     scaffold_9648  ATGGATGATATCCTGTTAGCTCATAAAAACGAGCCTGCACTTTTACAGTGCTATTCAATT 3240
    scaffold_17393  ATGGATGATATCCTGTTAGCTCATAAAAACGAGCCTGCACTTTTACAGTGCTATTCAATT 3240
    scaffold_12793  ATGGATGATATCCTGTTAGCTCATAAAAACGAGCCTGCACTTTTACAGTGCTATTCAATT 3240

                                      3,260                      3,280                      3,300
Ancestral_G7_Beta   ACAGAAGGAATTGGAACACTGGGGACTAAAAATTGCCCCTGAAAAGGTACAACGGGAAGA 3300
    scaffold_12699  ACAAAAGGAATTGGAACACTGGGGACTAAAAATTGCCCCTGTAAAGGTACAATGGGAAGA 3300
     scaffold_2273  ACAGACGGAATTGGAACATTGGGGACTAAAAATTGCCCCTGAAAAGGTACAACGGGAAGA 3300
GeneScaffold_1344   ACAGAAGGAATTAGAACATTAGGGACTAAAAATTGCCCCTGAAAAGGTACAACGAGAAGA 3300
     scaffold_9648  ACAGAAGGAATTGGAACATTGGGGACTAAAAATTGCCCCTGAAAAGGTACAACGAGAAGA 3300
    scaffold_17393  ACAGAAGGAATTGGAACATTGGGGACTAAAAATTGCCCCTGAAAAGGTACAACGAGAAGA 3300
    scaffold_12793  ACAGAAGGAATTGGAACACTGGGGACTAAAAATTGCCCCTGAAAAGGTACAACGGGAAGA 3300

                                      3,320                      3,340                      3,360
Ancestral_G7_Beta   GCCTTATACTTATCTGGGATACATGTTATATCCCAAGGTGATAATGCCTCAAAAGATACA 3360
    scaffold_12699  GCCCTATACTTATCTAGGATACATGTTATATCCCAAGGTGATAATGCCTCAAAAGATACA 3360
     scaffold_2273  GCCTTATACTTATCTGGGATACATGTTATATCCCAAGGTGATAATGCCTCAAAAGATACA 3360
GeneScaffold_1344   GCCTTATACTTATCTAGGATACATGTTATATCCCAAGGTGATAATGCCTCAAAAGATACA 3360
     scaffold_9648  GCCTTATACTTATCTAGGATACATGTTATATCCCAAGGTGATAATGCCTCAAAAGATACA 3360
    scaffold_17393  GCCTTATACTTATCTAGGATACATGTTATATCCCAAGGTGATAATGCCTCAAAAGATACA 3360
    scaffold_12793  GCCTTATACTTATCTGGGATACACGTTATATCCCAAGGTGATAATGCCTCAAAAGATACA 3360
```

```
                                            3,380                   3,400                   3,420
                                              |                       |                       |
Ancestral_G7_Beta  GATCGATCATTTGAAAACTCTCAATGATTTTCAAAAATTTCTGGGGGACATTAATTGGGT 3420
    scaffold_12699  GATCGATCATTTGAAAACTCTCAATGATTTTCAAAAATTTCTGGGGGACATTAATTGGGT 3420
     scaffold_2273  GATCGATCATTTAAAAACTCTCAATGATTTTCAAAAATTTCTGGGGGACATTAATTGGGT 3420
GeneScaffold_1344  GATCGATCATTTGAAAACTCTCAATGATTTTCAAAAATTTCTAGGGGACATTAATTAGGT 3420
     scaffold_9648  GTTCGATCATTTGAAAACTCTCAATGATTTTCAAAAATTTCTGGGGGATATTAATTGGGT 3420
    scaffold_17393  GATCGATCATTTGAAAACTCTCAATGATTTTCAAAAATTTCTGGGGGACATTAATTGGGT 3420
    scaffold_12793  GATCGATCATTTAAAAACTCTCAATGATTTTCAAAAATTTCTGGGAGACATTAATTGGGT 3420

                                            3,440                   3,460                   3,480
                                              |                       |                       |
Ancestral_G7_Beta  AAGACCTCATTTAAAGATTACCACTGGAGAATTAAAACCGGTTTGATATATTAAAAGGCG 3480
    scaffold_12699  AAGACCTCATTTAAAGATTACCACTGGAGAATTAAAACCGGTTTGATATACTAAAAGGTG 3480
     scaffold_2273  AAGACCTCATTTAAAGATTACCACTGGAGAATTAAAACCAGTTTGATGTATTAAAAGGCG 3480
GeneScaffold_1344  AAGACCTCATTTAAAGATTACCACTAGAGAATTAAAACCGGTTTGATATATTAAAAGGCG 3480
     scaffold_9648  AAGACCTCATTTAAAGATTACCACTGGAGAATTAAAACCGGTTTGATATATTAAAAGGCG 3480
    scaffold_17393  AAGACCTCATTTAAAGATTACCACTGGAGAATTAAAACTGGTTTGATATATTAAAAGGCG 3480
    scaffold_12793  AAGACCTCATTTAAAGATTACCACTGGAGAATTAAAACCAGTTTGATATATTAAAAGGCG 3480

                                            3,500                   3,520                   3,540
                                              |                       |                       |
Ancestral_G7_Beta  ATGCGTCTCCTACATCACCTAGACAATTGACACAGGAAGCTTTACAGCTGGTAGAATCCG 3540
    scaffold_12699  ATGCGTCTCCTACATCACCTAGACAATTGACACAGGAAGCTTTACAGCTGGTAGAATCCG 3540
     scaffold_2273  ATGCGTCTCCTACATCACCTAGACAATTTACACAGGAAGCTTTACAGCTGGTAGAATCCG 3540
GeneScaffold_1344  ATGCGTCTCCTACATCACCTAGACAATTGACACAGGAAGCTTTACAGCTAGTAGAATCTG 3540
     scaffold_9648  ATGCGTCTCCTACATCACCTAGACAATTGACACAGGAAGCTTTACAGCTAGTAGAATCTG 3540
    scaffold_17393  ATGCATCTCCTACATCACCTAGACAATTGACACAGGAAGCTTTACAGCTGGTAGAATCTG 3540
    scaffold_12793  ATGTATCTCCTACATCACCTAGACAATTGACACAGGAAGCTTTACAGCTGGTAGAATCCA 3540

                                            3,560                   3,580                   3,600
                                              |                       |                       |
Ancestral_G7_Beta  CTATATCTGCCCAGCAGGTACAATATATTGATTATACTCAAGAGTGGTATGGATATGTTC 3600
    scaffold_12699  CTATATCTGCCCAGCAGGTACAATATATTGATTATACTCAAGAGTGGTATGGATATGTTC 3600
     scaffold_2273  CTATATCTGCCCAGCAGGTACAATATATTGATTATACTCAAGAGTGGTATGAATATGTTC 3600
GeneScaffold_1344  CTATATCTGCCCAGCAGGTACAATATATTGATTATACTCAAGAGTAGTATGGATATGTTC 3600
     scaffold_9648  CTATATCTGCCCAGCAGGTACAATATATTGATTATACTCAAGAGTGGTATGGATATGTTC 3600
    scaffold_17393  CTATATCTGCCCAGCAGGTACAATATATTGATTATACTCAAGAGTGGTATGAATATGTTC 3600
    scaffold_12793  CTATATCTGCCCAGCGGGTACAATATGTTGATTATACTCAAGAGTGGTATGGATATGTTC 3600

                                            3,620                   3,640                   3,660
                                              |                       |                       |
Ancestral_G7_Beta  TCGCTACAAAACATACACCTACTGGCGTTTTGTGGCAGCATGGACCCTTACGATGGATCC 3660
    scaffold_12699  TCGCTACAAAACATACACCTACTGGCGTTTTGTGGCAGCATGGACCCTTACGATGGATCC 3660
     scaffold_2273  TCGCTACAAAACATACACCTACTGGCGTTTTGTGGCAGCATGGACCCTTACGATGCATCC 3660
GeneScaffold_1344  TCGCTACAAAACATACACCTACTGGCGTTTTGTGGCAGCATGGACCCTTACGATAGATCC 3660
     scaffold_9648  TCGCTACAAAACATACACCTACTGGCATTTTGTGGCAGCATGGACCCTTACGATGGATCC 3660
    scaffold_17393  TCGCTACAAAACATACACCTACTGGCGTTTTGTGGCAGCATGGACCCTTACAATGGATCC 3660
    scaffold_12793  TCGCTACAAAACATAAACCTACTGGCATTTTGTGGCAGCATGGACCCTTACGATGGATCC 3660

                                            3,680                   3,700                   3,720
                                              |                       |                       |
Ancestral_G7_Beta  ACCTCCCTGTGTCGCCTTCTAAAGTTCTCAATCCATATCATGAAGCAGTGGCTTCTCTCA 3720
    scaffold_12699  ACCTCCCTGTGTCGCCTTCTAAAGTTCTCAGTCCATATCATGAAGCAGTGGCTTCTCTCA 3720
     scaffold_2273  ACCTCCCTGTGTCCCCTTCTAAAGTTCTCAATCCATATCATGAAGCAGTGGCTTCTCTCA 3720
GeneScaffold_1344  ACCTCCCTGTGTCACCTGCTAAAGTTCTCAATCCATATCATAAAGCAGTGGCTTCTCTCA 3720
     scaffold_9648  ACCTCCCTGTGTCACCTGCTAAAGTTCTCAATCCATATCATGAAGCAGTGGCTTCTCTCA 3720
    scaffold_17393  ACCTCCCTGTGTCCCCTTCTAAAGTTCTCAATCCATATCATGAAGCAGTGGCTTCTCTCA 3720
    scaffold_12793  ACCTCCCTGTGTCGCCTTCTAAAGTTCTCAATCCTTATCATGAAGCAGTGGCTTCTCTCA 3720

                                            3,740                   3,760                   3,780
                                              |                       |                       |
Ancestral_G7_Beta  TACAGCTGTTAAGAGTCAATACTTTGGCAAAGAGCCACATATCATAGTAGTACCCTTTAC 3780
    scaffold_12699  TACAACTGTTAAGAGTCAATACTTTGGCAAAGAGCCACATATCATAGTAGTACCCTTTGC 3780
     scaffold_2273  TACAGCTGTTAAGAGTCAATACTTTGACAAAGAGCCACGTATCATAGTAGTACCCTTGAC 3780
GeneScaffold_1344  TACAGCTGTTAAGAGTCAATACTTTGGCAAAGAGCCACATATTATAGTAGTACCCTTTAC 3780
     scaffold_9648  TACAGCTGTTAAGAGTCAATACTTTGGCAAAGAGCCACATATTATAGTAGTACCCTTTAC 3780
    scaffold_17393  TACAGCTATTAAGAGTCAATACTTTGGCAAAGAGCCACATATCATAGTAGTACCCTTTAC 3780
    scaffold_12793  TACAGCTGTTAAGAGTCAATACTTTGGCAAAGAGCCACATGTCATAGTAGTACCCTTTAC 3780
```

```
                                         3,800                                    3,820                                    3,840
Ancestral_G7_Beta  TCAACAGCAATTAAATTGGTTATTTCAAAATTCTGACTCATGGAACATAGCCTTTGCATA 3840
    scaffold_12699  TCAACAGCAATTAAATTGGCTATTTCAAAATTCTGACTCATGGAACATAGCCTTTGCATA 3840
     scaffold_2273  TCAACAGCAATTAAATTGGTTATTTCAAAATTCTGACTCATGGAACATAGCCTTTGCATA 3840
GeneScaffold_1344  TCAGCAGCAATTAAATTGGCTATTTCAAAATTCTGATTCATGGAACATAGCCTTTGCATA 3840
     scaffold_9648  TCAACAGCAATTAAATTGGTTATTTCAAAATTCTGACTCATGGAACATAGCCTTTGCATA 3840
    scaffold_17393  TCAACAGCAATTAAATTGGTTATTTCAAAATTCTGACTCATGGAACATAGCCTTTGCATA 3840
    scaffold_12793  TCAACAGCAATTAAATTGGTTATTTCAAAATTCTGACTCATGGAACATAGCCTTTGCATA 3840
                                         3,860                                    3,880                                    3,900
Ancestral_G7_Beta  TTACAGGAAAAATTGACAATCATTATGCTGGTGACAAACTTTTACAGTTTGCTAATATGC 3900
    scaffold_12699  TTACAGGAAAAATTGACAATCATTATGCTAGTAACAAACTTTTACAGTTTGCTAATATGC 3900
     scaffold_2273  TTACAGGAAAAATTGACAATCATTATGCTGGTGACAAACTTTTACAGTTTGCTAATATGC 3900
GeneScaffold_1344  CTACAGGAAAAATTGACAATCATTATGCTAGCAACAAACTTTTACAGTTTGCTAATATAC 3900
     scaffold_9648  TTACAGGAAAAATTGACAATCACTATACTGGTGACAAACTTTTACAGTTTGCTAATATGC 3900
    scaffold_17393  TTACAGGAAAAATTGACAATCACTATACTGGTGACAAACTTTTACAGTTTGCTAATATGC 3900
    scaffold_12793  TTACAGGAAAAATTGACAATCATTATGCTGGTGACAAACTTTTACAGTTTGCTAATATGC 3900
                                         3,920                                    3,940                                    3,960
Ancestral_G7_Beta  AATCATTTATCTTTCCTCGCGTAATTAAGACCACCCCGATACAGAATGCTCTTTATATTT 3960
    scaffold_12699  AATCATTTATCTTTCCTCGCGTAATTAAGACCACCCCGATACAGAATGCTCTTTTTATTT 3960
     scaffold_2273  AATCATTTATCTTTCCTCGTATAATTAAGACCACCCCGATACAGAATGTTCTTTATATTT 3960
GeneScaffold_1344  AGTCATTTATCTTTCCTCGTGTAATTAAGACCACCCCGATACAGAATGCTCTTTATATTT 3960
     scaffold_9648  AATCATTTATCTTTCCTCTTGTAATTAAGACCACCCCGATACAGAATGCTCTTTATATTT 3960
    scaffold_17393  AATCATTTATCTTTCCTCTTGTAATTAAGACCACCCCGATACAGAATGCTCTTTATATTT 3960
    scaffold_12793  AATCATTTATCTTTCCTCGCGTAATTAAGACTACCCCGATACAGAATGCTCTTTGTATTT 3960
                                         3,980                                    4,000                                    4,020
Ancestral_G7_Beta  ACTGATGGAAAAGCCGTGTATGCTGTTGACAACCAAACCTACTGCATACAAACATCCCCT 4020
    scaffold_12699  ACTGATGGGAAAGCCGTGTATGCTGTTGACAACCAAACCTATTGCATGCAAACATCCCCT 4020
     scaffold_2273  ACTGATGGAAAAGCCGTGTATGCTGTTGACAATCAAACCTACTGCATACAAACATCCCCT 4020
GeneScaffold_1344  ACTGATGGAAAAGCCGTATATGCTGTTGACAACCAAACCTATTGCATACAAACATCCCCT 4020
     scaffold_9648  ACTGATGGAAAAGCCGTGTATGCTGTTGACAACCAAACCTATTGCATACAAACATCCCCT 4020
    scaffold_17393  ACTGATGGAAAAGCCGTGTATGCTGTTGACAACCAAACCTATTGCATACAAACATCCCCT 4020
    scaffold_12793  ACTGATGGAAAAGCCATGTATGCTGTTGACAACCAAACCTACTGCATACAAACATCCCCT 4020
                                         4,040                                    4,060                                    4,080
Ancestral_G7_Beta  GCATCTGCTCAGATTGTAGAATTGCGGGCAGTTGCTGCGGTTTTTCAAATATTTGCTCCC 4080
    scaffold_12699  GCATCTGCTCAGATTGTAGAATTGCGGGCAGTTGCTGCGGTTTTTCAAATATTTGCTCCC 4080
     scaffold_2273  GCATCTGCTCAGATTGTAGAATTGCAGGAAGTTGCTGCGGTTTTTCAAATACTTGCTCCC 4080
GeneScaffold_1344  GCATCTGCTCAGATTGTAGAATTGCGGGCAGTTGCTGCAATTTTTCAAATATTCGCTCCC 4080
     scaffold_9648  GCATCTGCTCAGATTGTAGAATTGCGGGCAGTTGCTGCAATTTTTCAAATATTCGCTCCC 4080
    scaffold_17393  GCATCTGCTCAGATTGTAGAATTGCGGGCAGTTGCTGCAATTTTTCAAATATTTGCTCCC 4080
    scaffold_12793  GCATCTGCTCAGATTGTAGAATTGCGAGCAGTTGCTGCGGTTTTTCAAATATTTGCTCCC 4080
                                         4,100                                    4,120                                    4,140
Ancestral_G7_Beta  CTTAACCTATATACTGATAGTCAATACATTGCTAAAGCTTTAATCATATTGGAGACTGTA 4140
    scaffold_12699  CTTAACCTATATACTGATAGTCAATACATTGCTAAAGCTTTAATTATATTGGAGACTGTA 4140
     scaffold_2273  CTTAACTTACATACTGATAGTCAATACGTTGCTAAAACTGTAATCATATTGGAGACTGTA 4140
GeneScaffold_1344  CTTAACCTATATACTGATAGTCAATACATTGCTAAAGCTTTAATTATATTGGAGACTATA 4140
     scaffold_9648  CTTAACCTATATACCGATAGTCAATACATTGCTAAAGCTTTAATTATATTGGAGACTGTA 4140
    scaffold_17393  CTTAACCTATATACCGATAGTCAATACATTGCTAAAGCTTTAATTATATTGGAGACTGTA 4140
    scaffold_12793  CTTATCCTATATACTGATAGTCAATACATTGCTAAAGCTTTAATCATATTGGAGACTGTA 4140
                                         4,160                                    4,180                                    4,200
Ancestral_G7_Beta  CCTTATATTGCTACCCACAATTCTGAGGTGCATTCTTATTGGCGCAAATTCAATTGGCTA 4200
    scaffold_12699  CCTTATATTGCTACCCACAATTCTGAGGTGCATTCTTATTGGCGCAAATTCAATTGGCTA 4200
     scaffold_2273  CCTTATATTGCTACCCACAATTCTGAGGTGCATTTTTATTGGCGCAAATTCAATTGGCTA 4200
GeneScaffold_1344  CCTTATATTGCTACCCACAATTCTGAGGTGCATTCTTATTAGCGCAAATTCAATTAGCTA 4200
     scaffold_9648  CCTTATATTGCTACCAACAATTCTAAGGTGCATTCTTATTGGCGCAAATTCATTTGGCTA 4200
    scaffold_17393  CCTTATATTGCTACCCACAATTCTGAGGTGCATTCTTATTGGCGCAAATTCAATTGGCTA 4200
    scaffold_12793  CCTTATATTGCTACCCACAATTCTGAGGTGCATTCTTATTGGCACAAATTCAATTGACTA 4200
```

```
                                               4,220                                            4,240                                            4,260
Ancestral_G7_Beta   TACACAACAGAAAAGAATTCTGCTATGTGACTCATTCGAGCTCATACAAATCTCCCTGGT  4260
scaffold_12699      TACACAACAGAAAAGAATTCTGCTATGTGACTCATTCGAGCTCGTACAAATCTCCCTGGT  4260
scaffold_2273       TACACAACAGAAAAGAATTCTGCTATGTGACTCATTCGAGCTTATACAAATCTCCCTGGT  4260
GeneScaffold_1344   TGCACAACAGAAAAGAATTCTGCTATGTGACTCATTCGAGCTCATACAAATCTCCCTGGT  4260
scaffold_9648       TGCACAACAGAAAAGAATTCTGCTATGTGACTCATTCGAGCTCATACAAATCTCCCTGGT  4260
scaffold_17393      TGCACAACAGAAAAGAATTCTGCTATGTGACTCATTCGAGCTCATACAAATCTCCCTGGT  4260
scaffold_12793      TACACAACAGAAAAGAATTCTGCTATGTGACTCATTCGAGCTCATACAAATCTCCCTGGT  4260

                    4,280  4,300  4,320
Ancestral_G7_Beta   CCTTTGTCTAAGGCTAATGCACTGTTGATGCAGCCACCCACCTACTTACTAGTAATGTTG  4320
scaffold_12699      CCTTTGTCTAAGGCTAATGCACTGTTGATGCAGCCACCCACCTACTTACTAGTAATGTTG  4320
scaffold_2273       CCTTTGTCTAAGGCTAATGCACTGTTGATGCAGCCACCCACCTACTTACTAGTAATGTTG  4320
GeneScaffold_1344   CCTTTGTCTAAGGCTAATACACTGTTGATGCAGCCACCCACCTACTTGCTAGTAATGTTG  4320
scaffold_9648       CCTTTGTCTAAGGCTAATGCACTGTTGATGCAGCCACCCACCTACTTACTAGTAATGTTG  4320
scaffold_17393      CCTTTGTCGAAGGCTAATGCACTGTTGATGCAGCCACCCACCTACTTGCTAGTAATGTTG  4320
scaffold_12793      CCTTTGTCTAAGGCTAATGCACTGTTGATGCAGCCACCCACCTACTTACTAGTAATGTTG  4320

                    4,340  4,360  4,380
Ancestral_G7_Beta   AACAAGCACGACAATCTCACGCTATACATCATCAAAATAGCTCTTATCTAAGAAGGCAAT  4380
scaffold_12699      AACAAGCACGACAACCTCACGCTATACATCATCAAAATAGCTCTTATCTAAGAAAGCAAT  4380
scaffold_2273       AACAAGCACGACAATCTCATGCTATACATCATCAAAATAGCTCTTATCTAAGAAAGCAAT  4380
GeneScaffold_1344   AACAAGCACGACAATCTCATGCTATACATCATCAAAATAGTTCTTATCTAAAAAGGCAAT  4380
scaffold_9648       AACAAGCACGGCAATCTCACGCTATACATCATCAAAATAGCTCTTATCTAAGAAAGCAAT  4380
scaffold_17393      AACAAGCACGACAATCTCATGCTATACATCATCAAAATAGTTCTTATCTAAGAAAGCAAT  4380
scaffold_12793      AACAAGCATGACAATCTCACGCTATACATCATCAAAATAGCTCTTATCTAAGAAAGCAAT  4380

                    4,400  4,420  4,440
Ancestral_G7_Beta   TTCATCTCACCAGAGAAGCAGCAAGACAAATTATAAAAAGTTGTCCCACTTGCCCTCAGT  4440
scaffold_12699      TTCATCTCACAAGAGAAGCAGCAAGACAAATTATAAAAAGTTGTCCCACGTGCCCTCAGT  4440
scaffold_2273       TTCATCTCACCAGAGAAGCAGCAAGACAAATTATAAAAAGTTGTCCCACTTGCCCTCAGT  4440
GeneScaffold_1344   TTCATCTCACCAGAGAAGCAGCAAGACAAATTATAAAAAGTTGTCCCACTTGCCCTCAGT  4440
scaffold_9648       TTCATCTCACCAGAGAAGCAGCAAGACAAATTATAAAATGTTGTCCCACTTGCCTTCAGT  4440
scaffold_17393      TTCATCTCACCAGAGAAGCAGCAAGACAAATTATAAAAAGTTGTCCCACTTGCCCTCAGT  4440
scaffold_12793      TTCATCTCACCAGAGAAGCAGCAAGACAAATTATAAAAAGTTGTCCCACTTGCCCTCAGT  4440

                    4,460  4,480  4,500
Ancestral_G7_Beta   TTTTTCATGTTCCTCATTATGGTATTAATCCCCGTGGTTTGATGCCGAATCAAATTTGGC  4500
scaffold_12699      TTTTTCATGTTCCTCATTATGGTATTAATCCCCGTGATTTGATGCCGAATCAAATTTGGC  4500
scaffold_2273       TTTTTCATGTTCCTCATTATGGTATTAATCCCCATGGTTTGATGCCGAATCAAATTTGGC  4500
GeneScaffold_1344   TTTTTCATGTTCCTCATTATAGTATTAATCCCCATGGTTTGATACCGAATCAAATTTGGC  4500
scaffold_9648       TTTTTCATGTTCCTCATTATGGTATTAATCCCCGTGGTTTGATACCGAATCAAATTTGGC  4500
scaffold_17393      TTTTTCATGTTCCTCATTATGGTATTAATCCCCGTGGTTTGATACCGAATCAAATTTGGC  4500
scaffold_12793      TTTTTCATGTTCCTCATTATGGTATTAATCCCTGTGATTTGATGCCAAATCAAATTTGGC  4500

                    4,520  4,540  4,560
Ancestral_G7_Beta   AGATGGACGTAACCCACATTCCTGAATTTGGTAGATTAAAATATGTACATGTTACTGTAG  4560
scaffold_12699      AGATGGACGTAACCCACATTCCTGAATTTGGTAGATTAAAATATGTACATGTCACTGCAG  4560
scaffold_2273       AGATGGACGTAACCCACATTCCTGAATTTGGTAGATTAAAATATGTACATGTCACTGTAG  4560
GeneScaffold_1344   AAATGGATGTAACCCACATTCCTGAATTTGGTAAATTAAAATATGTACATGTTACTGTAG  4560
scaffold_9648       AAACGGACGTAACCCACATTCCTGAATTTGGTAGATTAAAATATGTACATGTTACTGTAG  4560
scaffold_17393      AGATGGATGAAACCCACATTCCTGAATTTGGTAAATTAAAATATGTACATGTTACTGTAG  4560
scaffold_12793      AGATGGATGTAACCCACATTCCTGAATTTGGTAGATTAAAATATGTACATGTTACTGTAG  4560

                    4,580  4,600  4,620
Ancestral_G7_Beta   ATACATTTTCTGGATTCATTTTTGCTTCTGCTTTGACTGGTGAAGCTACTAAACATGTTA  4620
scaffold_12699      ATACATTTTCTGGATTCATTTTTGCTTCTGCTTTGACTGGTGAAGCTGCTAAACATGTTA  4620
scaffold_2273       ATACATTTTCTGGATTCATTTTTGCTTCTGCTTTGACTGGTGAAGCTACTAAACATGTTA  4620
GeneScaffold_1344   ATACATTTTCTGGATTCATTTTCACTTCTGCTTTGACTGGTGAAGCCACTAGACATGTTA  4620
scaffold_9648       ATACATTTTCTGGATTCATTTTCGCTTCTGCTTTGACTGGTGAAGCCACTAAACATGTTA  4620
scaffold_17393      ATACATTTTCTGGATTCATTTTCGCTTCTGCTTTGACTGGTGAAGCCACTAAACATGTTA  4620
scaffold_12793      ATACATTTTCTGGATTCATTTTTGCTTCTGCTTTGACTGGTGAAGCTACTAAACATGTTA  4620
```

	4,640 4,660 4,680	
Ancestral_G7_Beta	TTAATCACTGCTTACGCTGCTTTGCAGCTATTGGATGTCCTCAAATTCTTAAGACAGACA	4680
scaffold_12699	TTAATCACTGCTTACGCTGCTTTGCAGCTATTGGATGTCCTCAAATTCTTAAAACAGATA	4680
scaffold_2273	TTAATCACTGCTTACGCTGCTTTGCAGCTATTGGATGTCCTTAAATTCTTAAAACAGACA	4680
GeneScaffold_1344	TTAATCACTGCTTACGCTGCTTTGCAGCTATTAGATGTCCTCAAATTCTTAAGACAGACA	4680
scaffold_9648	TTAATCACTGCTTACGCTGCTTTGCAGCTATTGGATGTCCTCAAATTCTTAAGACAGACA	4680
scaffold_17393	TTGATCACTGCTTACGCTGCTTTGCAGCTATTGGATGTCCTCAAATTCTTAAGACAGACA	4680
scaffold_12793	TTAATCACTGCTTACGCTGCTTTGCAGCTATTGGATGTCCTCAAATTCTTAAAACAGATA	4680

	4,700 4,720 4,740	
Ancestral_G7_Beta	ATGGCTCTGGTTATATTAGTGCTGCTTTTAAAACATTTTGCTCTCAGTTACATATTGAAC	4740
scaffold_12699	ACGGCTCTGGTTATATTAGTGCCGCTTTTAAAACATTTTGCTCTCAGTTACATATTGAAC	4740
scaffold_2273	ACGGCTCTGGTTATATTAGTGCTGCTTTTAAAACATTTTGCCCTCAGTTACATATTGAAC	4740
GeneScaffold_1344	ATGGCTCTAGTTATACTAGTGCCGCTTTTAAAACATTTTGCTCTCAGTTACATATTGAAC	4740
scaffold_9648	ATGGCTCTGGTTATACTAGTGCCGCTTTTAAAACATTTTGCTCTCAGTTACATATTGAAC	4740
scaffold_17393	ATGGCTCTGGTTATACTAGTGCCGCTTTTAAAGCATTTTGCTCTCAGTTACATATTGAAC	4740
scaffold_12793	ATGGCTCTGGTTATATTAGTGCTGCTTTTAAAACATTTTGCTCTCAGTTACATATTGAAC	4740

	4,760 4,780 4,800	
Ancestral_G7_Beta	AACTCAATTGATAAAATAAAAAAGGGGGAGTTATACCCCTTCTCCACAAAATTATTTAAA	4800
scaffold_12699	AACTCAAGTGAGAAAATAAAAAAGGGGGAATTATACCCCTTCTCCACAAAATTATTTAAA	4800
scaffold_2273	AACTCAATTGATAAAATAAAAAAGGGGGAGTTATACCCCTTCTCCACAAAATTATTTAAA	4800
GeneScaffold_1344	AACTCAATTGAGAAAATAAAAAAGGGGGAGTTATACCCCTTCTCCACAAAATTATTTAAA	4800
scaffold_9648	AACTCAATTAAGAAAATAAAAAAGGGGGAGTTATACCCCTTCTCCACAAAATTATTTAAA	4800
scaffold_17393	AACTCAATTGAGAAAATAAAAAAGGGGGAGTTATACCCCTTCTCCACAAAATTATTTAAA	4800
scaffold_12793	AACTCAATTGATAAAATAAAAAAGGGGGAGTTATACCCCTTCTCCACAAAATTATTTAAA	4800

	4,820 4,840 4,860	
Ancestral_G7_Beta	TCATGCCTTATTCATTTTAAATTTTTAAATTTGGATGCCAAAGGGCACTCTGCAGCTGAG	4860
scaffold_12699	TCATGCCTTATTCATTTTAAATTTTTAAATTTGGATGCCAAAGGGCACTCTGCAGCTGAG	4860
scaffold_2273	TCATGCCTTATTCATTTTAAATTTTTAAATTTGGATGCCAAAGGGCACTCTGCAGCTGAG	4860
GeneScaffold_1344	TCATGCCTTATTCATTTTAAATTTTTAAATTTGGATGCCAAAGGGCACTCTGCAGCTGAG	4860
scaffold_9648	TCATGCCTTATTCATTTTAAATTTTTAAATTTGGATGCCAAAGGGCACTCTGCAGCTGAG	4860
scaffold_17393	TCATGCCTTATTCATTTTAAATTTTTAAATTTGGATGCCAAAGGGCACTCTGCAGCTGAG	4860
scaffold_12793	TCATGCCTTATTCATTTTAAATTTTTAAATTTGGATGCCAAAGGGCACTCTGGAGCTGAG	4860

	4,880 4,900 4,920	
Ancestral_G7_Beta	CGCTTATGGCATCCCACTACTACATGCCACTGTAAGATGGAAAGATCCGTTAACTGGACA	4920
scaffold_12699	CGCTTATGGCATCCCACTACTACATGCCACTGTAAGATGGAAAGATCCGTTAACTGGACA	4920
scaffold_2273	CGCTTATGGCATCCCACTACTACAAGCCACTGTAAAATGGAAAGATCCATTAACCGGACA	4920
GeneScaffold_1344	CGCTTATGGCATCCCACTACTACATGCCACTGTAAGATGGAAAGATCCATTAACTGGACA	4920
scaffold_9648	CGCTTATGGCATCCCACTACTACATGCCACTGTAAGATGGAAAGATCCATTAACTGGACA	4920
scaffold_17393	CGCTTATGGCATCCCACTACTACATGCCACTGTAAGATGGAAAGATCCGTTAACTGGACA	4920
scaffold_12793	CGCTTATGGCATCCCACTACTACATGCCACTGTAAGATGGAAAGATCCGTTAACGGGACA	4920

	4,940 4,960 4,980	
Ancestral_G7_Beta	ATGGAATGGACCAGATCCTGTACTAATATGGGGACGAGGGCATGTTTGTGTTTTTCCGCA	4980
scaffold_12699	ATGGAATGGACCAGATCCTGTACTAATATGGGGACGAGGGCATGTTTGTGTTTTCCCGCA	4980
scaffold_2273	ATGGAATGGACCAGATCCTGTATTAATATGGGGACGAGGGCATGTTTGTGTTTTTCCGCA	4980
GeneScaffold_1344	ATAGAATAGACCAGATCCTGTACTAATATGGGGACGAGGGCATGTTTGTGTTTTTCCGCA	4980
scaffold_9648	ATGGAATGGACCAGATACTGTACTAATATGGGGACGAGGGCATGTTTGTGTTTTTCCGCA	4980
scaffold_17393	ATGGAATGGACCAGATCCTGTACTAATATGGGGGCGAGGGCATGTTTGTGTTTTTCCGCA	4980
scaffold_12793	ATGGAATGGACCAGATCCTGTACTAATATGGGGACGAGGGCATGTTTGTGTTTTTCCGCA	4980

	5,000 5,020 5,040	
Ancestral_G7_Beta	GGGAGCTGACGGAGCACGCTGGTTGCCTGAACGACTAGTGCGCATGCCGAAAATGAACAT	5040
scaffold_12699	GGGAGCTGACGGAGCACGCTGGTTACCTGAACGACTAGTGCGCATGCCGAAAATGAACAT	5040
scaffold_2273	GGGAGCTGATGGAGCACGCTGGTTGCCTGAGCGACTAGTGCGCATGCTGAAAATGAACAT	5040
GeneScaffold_1344	GGGAGCTGACGGAGCACGCTGGTTGCCTGAACGACTAGTGCGCATGCCGAAAATGAACAT	5040
scaffold_9648	GGGAGCTGACGGAGCACGCTGGTTGCCTGAACGACTAGTGCGCATGCCGAAAATGAACAT	5040
scaffold_17393	GGGAGCTGACGGAGCACGCTGGTTGCCTGAACGACTAGTGCGCATGCCGAAAATGAACAT	5040
scaffold_12793	GGGAGCTGACGGAGCACGCTGGTTGCCTGAACGACTAGTGCGCATGCCGAAAATGAACAT	5040

```
                                       5,060               5,080               5,100
                                           |                   |                   |
Ancestral_G7_Beta  CGAGATTATTCTTCTGATGGGAATACTGATTAATCCAACAGTTTATGCCGCAGTGCATTG  5100
    scaffold_12699  CGAGATTATTCTTCTGATGGGAATACTGATTAATCCAACAGTTTATGCCGCAGTGCATTG  5100
     scaffold_2273  CGAGATTATTCTTCTGATGGGAATACTGATTAATCCAACAGTTTATGCCGCAGTGCATTG  5100
GeneScaffold_1344  CGAGATTATTCTTCTGATGGGAATACTAATTAATCCAACAGCTTATGCTGCAGTGCATTG  5100
     scaffold_9648  CGAGATTATTCTTCTGATGGGAATACTGATTAATCCAACAGCTTATGCTGCAGTGCATTG  5100
    scaffold_17393  CGAGATTATTCTTCTGATGGGAATACTGATTAATCCAACAGTTTATGCCGCAGTGCATTG  5100
    scaffold_12793  CGAGATTATTCTTCTGATGGGAATACTGATTAATCCAACAGTTTATGCCGCAGTGCATTG  5100

                                       5,120               5,140               5,160
                                           |                   |                   |
Ancestral_G7_Beta  GGCTTACATCCCGGATCCTCCGGTACTACATCCTTTGGTCTGGGCCACAGATCCAGGTTT  5160
    scaffold_12699  GGCTTACATCCCGGATCCTCCGGTACTACATCCTTTGGTCTGGGCCACAGATCCAGGTTT  5160
     scaffold_2273  GGCTTACATCCCGGAACCTCCGGTACTACATCCTTTGGTCTGGGCCACAGATCCGGGTGT  5160
GeneScaffold_1344  GGCTTACATTCCGGATCCTCCGGTACTACATCCTTTGGTCTGGGCCACAGATCCAGGTTT  5160
     scaffold_9648  GGCTTACATTCCGGATCCTCCGGTACTACATCCTTTGGTCTGGGCCACAGATCCAGGTTT  5160
    scaffold_17393  GGCTTACATCCCAGATCCTCCGGTACTACATCCTTTGGTCTGGGCCACAGATCCAGGTTT  5160
    scaffold_12793  GGCTTACATCTCAGATCCTCCGGTACTACATCCTTTGGTCTGGGCCACAGATCCAGGTTT  5160

                                       5,180               5,200               5,220
                                           |                   |                   |
Ancestral_G7_Beta  TTTCTAATAACTTTTAGCAGCAATAGGACTACCTTTTGTTTTCTTTCTACTTCCCAACCT  5220
    scaffold_12699  TTTCTAATAACTTTTAGCAGCAATAGGACTACCTTTTGTTTTCTTTCTACTTCCCGACCT  5220
     scaffold_2273  TTTCTAATAACTTCCAGCAGCAATAGGACTACCTTTTGTTTTCTTTCTACTTCCCAACCT  5220
GeneScaffold_1344  TTTCTAATAACTTTTAGCAGCAATAGGACTACCTTTTGTTTTCTTTCTACTTCCCAACCT  5220
     scaffold_9648  TTTCTAATAACTTTTAGCAGCAATAGGACTACCTTTTGTTTTCTTTCTACTTCCCAACCT  5220
    scaffold_17393  TTTCTAATAACTTTTAGCAGCAATAGGACTACCTTTTGTTTTCTTTCTACTTCCCAACCT  5220
    scaffold_12793  TTTCTAATAACTTTTAGCAGCAATAGGACTACCTTTTGTTTTCTTTCTATTTACCAACCT  5220

                                       5,240               5,260               5,280
                                           |                   |                   |
Ancestral_G7_Beta  GGGTGTTTGAATATCACGGAGCAGCCTTGGCCTAGGAAAGGAGGAGCTGTTATGCTTGGG  5280
    scaffold_12699  GGGTGTTTAAATATCACGGAGCAGCCTTGGCCTAAGAAAGGAGGAGCTGTTATGCTTGGG  5280
     scaffold_2273  GGGTGTTTGAATATCACGGAGCAGCCTTGGCCTAAGAAAGGAGGGGCTGTTATGCTTGGG  5280
GeneScaffold_1344  GGGTGTTTGAATATCACGGAGCAGCCTTGGCCTAGGAAAAGAGGAGCTGTTATGCTTGGG  5280
     scaffold_9648  GGGTGTTTGAATATCACGGAGCAGCCTTGGCCTAGGAAAGGAGGAGCTGTTATGCTTGGG  5280
    scaffold_17393  GGGTGTTTGAATATCACGGAGCAGCCTTGGCCTAAGAAAGGAGGAGCTGTTATGCTTGGG  5280
    scaffold_12793  GAGTGTTTGAATATCACGGAGCAGCCTTGGCCTAAGAAAGGAGGAGCTGTTATGCTTGGG  5280

                                       5,300               5,320               5,340
                                           |                   |                   |
Ancestral_G7_Beta  GCTCCTGATGGCGAAGCTGTAAGCAACAATGATACGATCCCGTTTGAACTTTGTCACTCG  5340
    scaffold_12699  GCTCCTGATGGCGACGCTGTAAGCAACAACGATACGATCCTGTTTGAACTTTGTCACTGG  5340
     scaffold_2273  GCTCCTGATGGCGAAGCTGTAAGCAACAATGATACGATCTCGTTTGAACTTTGTCACTTG  5340
GeneScaffold_1344  GCTCCTGATGGCAAAGCTGTCAGCAATAATGATACAATTCCGTTTGAACTTTGTCACTCA  5340
     scaffold_9648  GCTCCTGATGGCAAAGCTGTAAGCAACAATGATACGATCCCGTTTGAACTTTGTCACTCA  5340
    scaffold_17393  GCTCCTGATGGCGAAGCTGTAAGCAATAATGATACGATCCCGTTTGAACTTTGTCACTCG  5340
    scaffold_12793  GCTCCTGATGGCGAAGCTGTAAGCAACAATGATACGATCCCGTTTGAACTTTGTCACTCG  5340

                                       5,360               5,380               5,400
                                           |                   |                   |
Ancestral_G7_Beta  AAGAGGCATGTGCAAAAAGATTCAGTATGGTGGCATCGTTGTCATCAAGAATGCCGGTAA  5400
    scaffold_12699  AAGAGGCATGTGCAAAAAGATTCAGTATGGTGGCATCGTTGTCATCAAGAATGCGGGTAA  5400
     scaffold_2273  AAGAGGCATGTGCAAAAAGATTCGGTATGGTGGCATCGCTGTCATCAAGAATGTGGGTAA  5400
GeneScaffold_1344  AAGAAGCCTGTGCAAAAAGATTCAGTATGGTGGCATCGTTGTCATCAAGAATGCCAGTAA  5400
     scaffold_9648  AAGAAGCCTGTGCAAAAAGATTCAGTATGGTGGCATCGTTGTCATCAAGAATGCCAGTAA  5400
    scaffold_17393  AAGAGGCATCTGCAAAAAGATTCAGTATGGTGGCATCGTTGTCATCAAGAATGCCAGTAA  5400
    scaffold_12793  AAGAGGCATGTGCAAAAAGATTCAGTATGGTGGCATCGTTGTCATCAAGAATGCCAGTAA  5400

                                       5,420               5,440               5,460
                                           |                   |                   |
Ancestral_G7_Beta  CAGGATTTAATGAAACTGTTTGGGACTTCTCTCGGCCTTATGACACTGGGACTGCTTTAT  5460
    scaffold_12699  CAGGATTTAATGAAACTCTTTGGGACTTCTCTCCGCCTTATGACACTGGGACTGCTTTAT  5460
     scaffold_2273  CAGGATTTAATGAGACTGTTTGGGACTTCTCTTGGCCTTATGACTCTGAGACTGCTTTAT  5460
GeneScaffold_1344  CAGGATTTAATGAAACTGTTTGGGACTTCTCTCGGCCTTATAACACTGAGACTGCTTTAT  5460
     scaffold_9648  CAGGATTTAATGAAACTGTTTGGGACTTCTCTCGGCCTTATAACACTGAGACTGCTTTAT  5460
    scaffold_17393  CAGGATTTAATGAAACTGTTTGGGACTTCTCTCGGCCTTATGACACTGGGACTGCTTTAT  5460
    scaffold_12793  CAGGATTTAATGAAACTGTTTGGGACTTCTCTCAGCCTTATGACACTGGGACTGCTTTAT  5460
```

```
                                  5,480                5,500                5,520
                                    |                    |                    |
Ancestral_G7_Beta  CTGCTGGACTCTGGAAAACAAGCTCTGGCGTGGCAGACTCATATTTGGAAACTGGCTGCC 5520
   scaffold_12699  CTGCCGGACTCTGGAAAACAAGCTCTGGCGTGGCAGACTCATATTTCGAAACTGGCTGCC 5520
    scaffold_2273  CTGCTGGACTCTGGAAAACAAGCTCTGGCGTGGCAGACTCATATTTGGAAACTGGCTGCC 5520
GeneScaffold_1344  CTGCTGGACTCTGGAAAACAAGCTCTGGCGTGGCAGACTCATATTTGGAAACTGGCTGCC 5520
    scaffold_9648  CTGCTGGACTCTGGAAAACAAGCTCTGGCGTGGCAGACTCATATTTGGAAACTGGCTGCC 5520
   scaffold_17393  CTGCTGGACTCTGGAAAACAAGCTCTGGCGTGGCAGACTCATATTTGGAAACTGGCTGCC 5520
   scaffold_12793  CTGCTGGACTCTGGAAAACAAGCTCTGGCGTGGCAGACTCATATTTGGAAACTGGCTGCC 5520
                                  5,540                5,560                5,580
                                    |                    |                    |
Ancestral_G7_Beta  GGACTTGTGACAATGAGTATTAGCAGACTAATGAATCTCGGACCAATTGTAGTGGTTCAG 5580
   scaffold_12699  GGACTTGTGACAATGAGTATTAGCAAACTAATGAATCTCGGACCAATTGTAGTGGTTCAG 5580
    scaffold_2273  GGACTTGTGACAATGAGTATTAGCAGACTAATGAATCTCGGACCAATTGTAGTGGTTCAG 5580
GeneScaffold_1344  GGACTTGTGACAATGAGTATTAGCAGACTAATGAATCTCGGACCAATTGTAGTGGTTCAG 5580
    scaffold_9648  GGACTTGTGACAATGAGTATTAGCAGACTAATGAATCTCGGACCAATTGTAGTGGTTCAG 5580
   scaffold_17393  GGACTTGTGACAATGAGTATTAGCAGACTAATGAATCTCTGACCAATTGTAGTGGTTCAG 5580
   scaffold_12793  AGACTTGTGACAATGAGTATTAGCAGACTAATGAATCTCTGACCAATTGTAGTGGTTCAG 5580
                                  5,600                5,620                5,640
                                    |                    |                    |
Ancestral_G7_Beta  GAGAAAGATTCTCATATGCCCGAACTTGTGTTAATGTCCCATATATATTATTGTTAGGAA 5640
   scaffold_12699  GAGAAAGATTCTTATATGCCCGAACTTGTGTTAATGTCCCATACATATTATTGTTAGGAA 5640
    scaffold_2273  GAGAAAGATTCTCATATGCCCGAACTTGTGTTAATGTCCCGTATATATTATTATTACGAA 5640
GeneScaffold_1344  GAGAAAGATTCTCATATGCCCGAACTTGTGTTAATGTCCCATATATGTTATTGTTAGGAA 5640
    scaffold_9648  GAGAAAGATTCTCATATGCCCGAACTTGTGTTAATGTCCCATATATGTTATTGTTAGGAA 5640
   scaffold_17393  GAGAAAGATTCTCAGATGCCCGAACTTGTGTTAATGTCCCATATATATTATTGTTAGGAA 5640
   scaffold_12793  GAGAAAGATTCTCATATGCCCGAACTTGTGTTAATGTCCCATATATATTATTGTTAGGAA 5640
                                  5,660                5,680                5,700
                                    |                    |                    |
Ancestral_G7_Beta  ATTTCAATATTACCGTAGATAAATCAATTTAATTGTAATAATTGTCAGCTGTCTAATTGT 5700
   scaffold_12699  ATTTTAATATTACCATAGATAAATCAATTTAATTGTAATAATTGTCAGCTGTCTAATTGC 5700
    scaffold_2273  ATTTCAATATTACTGTAGATAAATCAATTTAATTGTAATAATTGTCAGCTGTCTAATTGT 5700
GeneScaffold_1344  ATTTCAATATTACCGTAGATAAATCAATTTAATTGTAATAATTGTCAGCTGTCTAATTGT 5700
    scaffold_9648  ATTTCAATATTACCGTAGATAAATCAATTTAATTGTAATAATTGTCAGCTGTCTAATTGT 5700
   scaffold_17393  ATTTCAATATTACCGTAGATAAATCAATTTAATTGTAATAATTGTCAGCTGTCTAATTGT 5700
   scaffold_12793  ATTTCAATATTACCATAGATAAATCAATTTAATTGTAATAATTGTCAGCTGTCTAATTGT 5700
                                  5,720                5,740                5,760
                                    |                    |                    |
Ancestral_G7_Beta  GTTACTCCTGCTAACACGTTCTCGTGCTTAGACAACCGCCATTTATAATGGTACCTTACA 5760
   scaffold_12699  GTTACTCCTGTTAACACGTTCTCGTGCTTAGACAACCGCCATTTATAATGGTACCTTACA 5760
    scaffold_2273  GTTACTCCTGCTAACACGTTCTCGTGCTTAGACAACCGCCATTTATAATGGTACCTTACA 5760
GeneScaffold_1344  GTTACTCCTGTTAACACGTTCTCGTGCTTAGACAACCACCATTTATAATGGTACCTTACA 5760
    scaffold_9648  GTTACTCCTGTTAACACGTTCTCGTGCTTAGACAACCACCATTTATAATGGTACCTTACA 5760
   scaffold_17393  GTTACTCCTGCTAACACGTTCTCGTGCTTAGACAACCGCCATTTATCATGGTACCTTACA 5760
   scaffold_12793  GTTACTCCTGCTAACACGTTCTCGTGCTTAGACAACCGCCATTTATAATGGTACCTTACA 5760
                                  5,780                5,800                5,820
                                    |                    |                    |
Ancestral_G7_Beta  GGCCCTTGGTTTGATGAATATGGGTTACAGGTTTTACAAGAGATTAACATGGCATTAGGA 5820
   scaffold_12699  GGCCCTTGGTTTAATGAATATGGGTTACAGGTTTTACAAGAGATTAACATGGCATTAGGA 5820
    scaffold_2273  GGCCCTTGGTTTGATGAATATGGGTTACAGGTTTTACAAGAGATTAACATGGCATTAGGA 5820
GeneScaffold_1344  GGCCCTTGGTTTGATGAATATGGGTTACAGGTTTTACAAGAAATTAACACGGCCTTAGGA 5820
    scaffold_9648  GGCCCTTGGTTTGATGAATATGGGTTACAGGTTTTACAAGAAATTAACACGGCCTTAGGA 5820
   scaffold_17393  GGCTCTTGGTTTGATGAATATGGGTTACAGGTTTTACAAGAAATTAACATGGCATTAGGA 5820
   scaffold_12793  GGCCCTTGGTTTGATGAATATGGGTTACAGGTTTTAAAAGAAATTAACATGGCATTAGGA 5820
                                  5,840                5,860                5,880
                                    |                    |                    |
Ancestral_G7_Beta  AGATCTAAAAGGGCTGTGGCTGTTGTTGTGGCAGGTATTCTTGCAGTTGTCGCCTTGGTG 5880
   scaffold_12699  AGATCTAAAAGGGCTGTGGCTGTTGTTGTGGCAGGTATTCTTGCAGTTGTCGCCTTGGTG 5880
    scaffold_2273  AGATCTAAAAGGGCTGTGGCTGTTGTTGTGGTAGGTATTCTTGCGGTTGTCGCCTTGGTC 5880
GeneScaffold_1344  AGATCTAAAAGGGCTGTGGCTGTTGTTGTGGCAGGTATTCTTGCAGTTGTCACCTTGGTG 5880
    scaffold_9648  AGATCTAAAAGGGCTGTGGCTATTGTTGTGGCAGGTATTCTTGCAGGTGTCGCCTTGGTG 5880
   scaffold_17393  AGATCTAAAAGGGCTGTGGCTGTTGTTGTGGCAGGTATTCTTGCAGTTGTCACCTTGGTG 5880
   scaffold_12793  AGATCTAAAAGGGCTGTGGCTGTTGTTGTGGCAGCTATTCTTGCAGTTGTCGCCTTGGTG 5880
```

```
                                           5,900                5,920                5,940
                                             |                    |                    |
Ancestral_G7_Beta  GTTACAGCCACCACTGCTGCCATATCCTTGTCCAACTCTGTGCAAACTGCAACATTTGTT  5940
    scaffold_12699  GTTACAGCCACCACTGCTGCCATATCCTTGTCCAACTCTGTGCGAACTGCAACATTTGTT  5940
     scaffold_2273  GTTACGGCCGCCACTGCTGCCATATCCTTGTCCAACTCTGTGCAAACTGCAACATTTGTT  5940
GeneScaffold_1344  GTTGCAGCCACCACTGCTGCCATATCCTTGTCCAACTCTGTGCAAACTGCAACATTTGTT  5940
     scaffold_9648  GTTGCAGCCACCACTGCTGCCATATCCTTGTCCAACTCTGTGCAAACTGCAACATTTGTT  5940
    scaffold_17393  GTTACAGCCACCACTGCTGCCATATCCTTGTCCAACTCTGTGCAAACTGCAACATTTGTT  5940
    scaffold_12793  GTTACAGCCACCACTGCTGCCATATCCTTGTCCAACTCTGTGCAAACTGCAACATTTGTT  5940
                                           5,960                5,980                6,000
                                             |                    |                    |
Ancestral_G7_Beta  AATGATCTGACAAAAAATGTTTCATTAGCTATGGGATCTCAAGAAAGTATTGATGAAAAA  6000
    scaffold_12699  AATGATCTGACAAAAAATGTTTCATTAGCTATGGGATCTCAAGAAAGTATTGATGAAAAA  6000
     scaffold_2273  AATGATCTGACAAAAAATGTTTCATTAGCTATGGGATCTCAAGAAAGTATTGATGAAAAA  6000
GeneScaffold_1344  AATGATCTGACAAAAAATGTTTCATTAGCTATGGGATCTCAAGAAAGTATTGATGAAAAA  6000
     scaffold_9648  AATGATCTGACAAAAAATGTTTCATTAGCTATGGGATCTCAAGAAAGTATTGATGAAAAA  6000
    scaffold_17393  AATGATCTGACAAAAAATGTTTCATTAGCTATGGGATCTCAAGAAAGTATTGATGAAAAA  6000
    scaffold_12793  AATGATCTGACAAAAAATGTTTCATTAGCTATGGGATCTCAAGAAAGTATTGATGAAAAA  6000
                                           6,020                6,040                6,060
                                             |                    |                    |
Ancestral_G7_Beta  ATTGAACAAAAATTAGATGCACTATATGAAACAGTAAACTTTTTGGGAGAAGAAGTTCAA  6060
    scaffold_12699  ATTGAACAAAAATTAGGTGCACTACATGAAACAGTAAACTTTTTGGGAGAAGAAGTTCAA  6060
     scaffold_2273  ATTGAACAAAAATTAGATGCACTATATGAAACAGTAAACTTTTTGGGAGAAGCAGATCAA  6060
GeneScaffold_1344  ATTGAACAAAAATTAGATGCACTATATGAAACAGTAAACTTTTTGGGAGAAGAAGTTCAA  6060
     scaffold_9648  ATTGAACAAAAATTAGATGCACTATATGAAACAGTAAACTTTTTGAGAGAAGAAGTTCAA  6060
    scaffold_17393  ATTGAACAAAAATTAGATGCACTATATGAAACAGTAAACTTTTTGGGAGAAGAAGTTCAA  6060
    scaffold_12793  ATTGAACAAAAATTAGATGCACTATATGAAACAGTAAACTTTTTGGGAGAAGAAGTTCAA  6060
                                           6,080                6,100                6,120
                                             |                    |                    |
Ancestral_G7_Beta  GCCTTAAAATTGTCCTCCCGCTTCGTTGCCATGTACAATATCGATGGATTTGTGTTCCAA  6120
    scaffold_12699  GCCTTAAAGTTGTCCTCCCGCTTCGTTGCCATGCACAATATCGATGGATTTGTGTTCCAA  6120
     scaffold_2273  GCCTTAAAATTGTCCTCCAGCTTCGTTGCCATGTACAATATCGACGGATTTGTGTTCCAA  6120
GeneScaffold_1344  GCCTTAAAATTGTCCTCCCACTTCGTTGCCATGTACAATATCGATGGATTTGTGTTCCAA  6120
     scaffold_9648  GCCTTAAAATTGTCCTCCCACTTCGTTGCCATGTACAATATCGATGGATTTGTGTTCCAA  6120
    scaffold_17393  GCCTTAAAATTGTCCTCCCACTTCGTTGCTATGTACAATATCGATGGATTTGTGTTCCAA  6120
    scaffold_12793  GCCTTAAAATTGTCCTCCCGCTTCGTTGCTATGTACAATATCGATGGATTTGTGTTCCAA  6120
                                           6,140                6,160
                                             |                    |
Ancestral_G7_Beta  AAAGGTATAATAGTACCACCCATCCTTGGGATAAGATAAAAAATCATTTA  6170
    scaffold_12699  AAAGGTATAATAGTACCACCCATCCTTGGGATAAGATAAAAAATCATTTA  6170
     scaffold_2273  AAAGGTATAATAGTACCACCCATCCTTGGGATAAGGTAAAAAGTCATTTA  6170
GeneScaffold_1344  AAAGGTATAATAGTACCACTCATCCTTGGGATAAGATAAAAAATCATTTA  6170
     scaffold_9648  AAAGGTATAATAGTACCACCCATTCTTGGGATAAGATAAAAAATCATTTA  6170
    scaffold_17393  AAAGGTATAATAGTACCACCCATCCTTGGGATAAGATAAAAAATCATTTA  6170
    scaffold_12793  AAAGGTATAATAGTACCACCCATCCTTGGGATAAGATAAAAAATCATTTA  6170
```
