# Supplementary material for: Differential Evolution of Antiretroviral Restriction Factors in Pteropid Bats as Revealed by APOBEC3 Gene Complexity
Source: Mol Biol Evol. 2018 Mar 29;35(7):1626–37. doi: 10.1093/molbev/msy048 (PMC5995163; doi:10.1093/molbev/msy048)
Supplement: Supplementary Data [file msy048_supp.zip › Supplementary Data 3 - Gammaretrovirus alignment.pdf]

```
                                          20                   40                   60
                                           |                    |                    |
Ancestral_Gamma_G1  TGGAGGCCCC AGCGAGATCT TCTCGTTGTG GGAAAATTGA TCAGTGCACC GGGGCGTTGG 60
 GeneScaffold_3363  TGGAGGCCCC AGCGAGATCT TCTCGTTGTG GGAAAATTGA TCAGTGCACC GGGGCCTTGG 60
     scaffold_1753  TGGAGGCCCC AGCGAGATCT TCTCGTTGTG GGAAAATTGA TCAGTGCACC GGGGCGTTGG 60
    scaffold_13050  TGGAGGCCCC AGCGAGATCT TCTCGTTGTG GGAAAATTGA TCAGTGCACT GGAGCGTTGG 60
    scaffold_24606  TGGAGGCCCC AGCGAGATCT TCTCGTTGTG GGAAAATTGA TCAGTGCACC GGAGCGTTGG 60
    scaffold_10119  TGGAGGCCCC AGCGAGATCT TCTCGTTGTG GGAAAATTGA TCAGTGCACC GGGGCGTTGG 60
    scaffold_23277  TGGAGGCCCC AGCGAGATCT TCTCGTTGTG GGAAAATTGA TCAGTGCACC GGGGCGTTGG 60
    scaffold_12630  TGGAGGCCCC AGCGAGATCT TCTCGTTGTG GGAAAATTGA TCAGTGCACC GGGGCGTTGG 60
     scaffold_2151  TGGAGGCCCC AGCGAGATCT TCTCGTTGTG GGAAAATTGA TCAGTGCACC GGAGCGTTGG 60
     scaffold_7237  TGGAGGCCCC AGCGAGATCT TCTCGTTGTG GGAAAATTGA TCAGTGCACC GGAGCGTTGG 60
    scaffold_12163  TGGAGGCCCC AGCGAGATCT TCTCGTTGTG GGAAAATTGA TCAGTGCACC GGGGCGTTGG 60
      scaffold_941  TGGAGGCCCC AGCGAGATCT TCTCGTTGTG GGAAAATTGA TCAGTGCACC AGGGCGTTGG 60
    scaffold_22661  TGGAGGCCCC AGCGAGATCT TCTCGTTGTG GGAAAATTGA TCAGTGCACC AGGGCGTTGG 60
     scaffold_7076  TGGAGGCCCC AGCGAGATCT TCTCGTTGTG GGAAAATTGA TCAGTGCACC AGGGCGTTGG 60
    scaffold_22354  TGGAGGCCCC AGCGAGATCT TCTCGTTGTG GGAAAATTGA TCAGTGCACC AGGGCGTTGG 60
    scaffold_14383  TGGAGGCCCC AGCGAGATCT TCTCGTTGTG GGAAAATTGA TCAGTGCACC AGGGCGTTGG 60
                                          80                  100                  120
                                           |                    |                    |
Ancestral_Gamma_G1  ACCATTTGCG GTTGGGGAGG CCTCTAGGGC ACTTGCCCTA GCCCGCCGCC TGTGGTTTCT 120
 GeneScaffold_3363  ACCATTTGCG GTTGGGGAGG CCTCTAGGGC ACTTGCCCTA GCCCACCGCC TGTGGTTTCT 120
     scaffold_1753  ACCATTTGCG GTTGGGGAGG CCTCTAGGGC ACTTGCCCTA GCCCGCTGCC TGTGGTTTCT 120
    scaffold_13050  ACCATATGCG GTTGGGGAGG CCTCTAGGGC ACTTGCCCTA GCCCGCCGCC TGTGGTTTCT 120
    scaffold_24606  ACCATTTGCG GTTGGGGAGG CCTCTAGGGC ACTTGCCCTA GCCCGCCGCC TGTGGTTTCT 120
    scaffold_10119  ACCATTTGCG GTTGGGGAGG CCTCTAGGGC ACTTGCCCTA GCCCGCCGCC TGTGGTTTCT 120
    scaffold_23277  ACCATTTGCG GTTGGGGAGG CCTCTAGGGC ACTTGCCCTA GCCCGCCGCC TGTGGTTTCT 120
    scaffold_12630  ACCATTTGCG GTTGGGGAGG CCTCTAGGGC ACTTGCCCTA GCCCGCCGCC TGTGGTTTCT 120
     scaffold_2151  ACCATTTGCG GTTGGGGAGG CCTCTAGGGC ACTTGCCCTA GCCCGCCGCC TGTGGTTTCT 120
     scaffold_7237  ACCATTTGCG GTTGGGGAGG CCTCTAGGGC ACTTGCCCTA GCCCGCCGCC TGTGGTTTCT 120
    scaffold_12163  ACCATTTGCG GTTGGGGAGG CCTCTAGGGC ACTTGCCCTA GCCCGCCGCC TGTGGTTTCT 120
      scaffold_941  ACCATTTGCG GTTGGGGAGG CCTCTAGGGC ACTTGCCCTA GCCCACCGCC CGTGGTTTCT 120
    scaffold_22661  ACCATTTGCG GTTGGGGAGG CCTCTAGGGC ACTTGCCCTA GCCCACCGCC CGTGGTTTCT 120
     scaffold_7076  ACCATTTGCG GTTGGGGAGG CCTCTAGGGC ACTTGCCCTA GCCCACCGCC CGTGGTTTCT 120
    scaffold_22354  ACCATTTGCG GTTGGGGAGG CCTCTAGGGC ACTTGCCCTA GCCCACCGCC CGTGGTTTCT 120
    scaffold_14383  ACCATTTGCG GTTGGGGAGG CCTCTAGGGC ACTTGCCCTA GCCCACCGCC CGTGGTTTCT 120
                                         140                  160                  180
                                           |                    |                    |
Ancestral_Gamma_G1  CGCTTTGCGG AGCACGATCC TGGGAGTGCT GAGGCAGTTT CTGGTAGGGG GCCCAGGACC 180
 GeneScaffold_3363  CGCTTTGCGG AGCACGATCC TGGGAGTGCT GAGGCAGTTT CTGGTAGGGG GCCCAGGACC 180
     scaffold_1753  CGCTTTGCGG AGCACGATCC TGGGAGTGCT GAGGCAGTTT CTGGTAGGGG GCCCAGGACC 180
    scaffold_13050  CGCTTTGCGG AGCACGATCC TGGGAGTGCT GAGGCAGTTT CTGGTAGGGG GCCCAGGACC 180
    scaffold_24606  CGCTTTGCGG AGCACGATCC TGGGAGTGCT GAGGCAGTTT CTGGTAGGGG GCCCAGGACC 180
    scaffold_10119  CGCTTTTCGG AGCACGATCC TGGGAGTGCT GAGGCAGTTT CTGGTAGGGG GCCCAGGACC 180
    scaffold_23277  CGCTTTGCGG AGCACGATCC TGGGAGTGCT GAGGCAGTTT CTGGTAGGGG GCCCAGGACC 180
    scaffold_12630  CGCTTTGCGG AGCACGATCC TGGGAGTGCT GAGGCAGTTT CTGGTAGGGG GCCCAGGACC 180
     scaffold_2151  CGCTTTTCGG AGCACGATCC TGGGAGTGCT GAGGCAGTTT CTGGTAGGGG GCCCAGGACC 180
     scaffold_7237  CACTTTGCGG AGCACGATCC TGGGAGTGCT GAGGCAGTTT CTGGTAGGGG GCCCAGGACC 180
    scaffold_12163  CGCTTTGCGG AGCACGATCC TGGGAGTGCT GAGGCAGTTT CTGGTAGGGG GCCCAGGACC 180
      scaffold_941  TGCTTTGTGG AGCGTGATCT TGGGAGTGCT GAGGCAGTTT TTGGTAGGGG CCCCAGGACC 180
    scaffold_22661  CGCTTTGTGG AGCATGATCT TGGGAGTGCT GAGGCAGTTT TTGGTAGGGG CCCCAGGACC 180
     scaffold_7076  CGCTTTGTGG AGCGTGATCT TGGGAGTGCT GAGGCAGTTT TTGGTAGGGG CCCCAGGACC 180
    scaffold_22354  CGCTTTGTGG AGCGTGATCT TGGGAGTGCT GAGGCAGTTT TTGGTAGGGG CCCCAGGACC 180
    scaffold_14383  CACTTTGTGG AGCGTGATCT TGGGAGTGCT GAGGCAGTTT TTGGTAGGGG CCCCAGGACC 180
                                         200                  220                  240
                                           |                    |                    |
Ancestral_Gamma_G1  GTGCGGACGT CGGACGCGAC AAGTAAGTCG GCGGTCGACT GAGACTGTGA CGACAGTTTT 240
 GeneScaffold_3363  GTGCGGACGT CGGACGCGAC AAGTAAGTCG GCGGTCGACT GAGACTGTGA CGACAGTTTT 240
     scaffold_1753  GTGCGGACGT CGGACGCGAC AAGTAAGTCG GCGGTCGACT GAGACTGTGA CGACAGTTTT 240
    scaffold_13050  GTGCGGACGT CGGACGCGAC AAGTAAGTCG GCGGTCGACT GAGACTGTGA CGACAGTTTT 240
    scaffold_24606  GTGCGGACGT CGGACGCGAC AAGTAAGTCG GCGGTCGACT GAGACTGTGA CGACAGTTTT 240
    scaffold_10119  GTGCGGACGT CGGACGCGAC AAGTAAGTCG GCGGTCGACT GAGACTGTGA CGACAGTTTT 240
    scaffold_23277  GTGCGGACGT CGGACGCGAC AAGTAAGTCG GCGGTCGACT GAGACTGTGA CGACAGTTTT 240
    scaffold_12630  GTGCGGACGT CGGACGCGAC AAGTAAGTCG GCGGTCGACT GAGACTGTGA CGACAGTTTT 240
     scaffold_2151  GTGCGGACGT CGGACGCGAC AAGTAAGTCG GCGGTCGACT GAGACTGTGA CGACAGTTTT 240
     scaffold_7237  GTGCGGACGT CGGACGCGAC AAGTAAGTCG GCGGTCGACT GAGACTGTGA CGACAGTTTT 240
    scaffold_12163  GTGCGGACGT CGGACGCGAC AAGTAAGTCG GCGGTCGACT GAGACTGTGA CGACAGTTTT 240
      scaffold_941  GTGCGGACGT CGGACGCGAC AAGTAAGTCG GCGGTCGACT GAGACTGTGA CGACAGTTTT 240
    scaffold_22661  GTGCGGACGT CGGACGCGAC AAGTAAGTCG GCGGTCGACT GAGACTGTGA CGACAGTTTT 240
     scaffold_7076  GTGCGGACGT CGGACGCGAC AAGTAAGTCG GCGGTCGACT GAGACTGTGA CGACAGTTTT 240
    scaffold_22354  GTGCGGACGT CGGACGCGAC AAGTAAGTCG GCGGTCGACT GAGACTGTGA CGACAGTTTT 240
    scaffold_14383  GTGCGGACGT CGGACGCGAC AAGTAAGTCG GCGGTCGACT GAGACTGTGA CGACAGTTTT 240
```

```
                                             260                  280                  300
                                               |                    |                    |
Ancestral_Gamma_G1  CGTCCTGTTC TGGTTTGTTA GTCTGTGTCT GTCTTTTTTG TTTTGCTTCT GCTTTATTGT 300
 GeneScaffold_3363  CGTCCTGTTC TGGTTTGTTA GTCTGTGTCT GTCTTTTTTG TTTTGCTTCT GCTTTATTGT 300
     scaffold_1753  CGTCCTGTTC TGGTTTGTTA GGCTGTGTCT GTCTTTTTTG TTTTGCTTCT GCTTTATTGT 300
    scaffold_13050  CGTCCTGTTC TGGTTTGTTA GTCTGTGTCT GTCTTTTCTG TTTTGCTTCT GCTTTATTGT 300
    scaffold_24606  CGTCCTGTTC TGGTTTGTTA GTCTGTGTCT GTCTTTTTTG TTTTGCTTCT GCTTTATTGT 300
    scaffold_10119  CGTCCTGTTC TGGTTTGTTA GTCTGTGTCT GTCTTTTTTG TTTTGCTTCT GCTTTATTGT 300
    scaffold_23277  CGTCCTGTTC TGGTTTGTTA GTCTGTGTCT GTCTTTTTTG TTTTGCTTCT GCTTTATTGT 300
    scaffold_12630  CGTCCTGTTC TGGTTTGTTA GTCTGTGTCT GTCTTTTTTG TTTTGCTTCT GCTTTATTGT 300
     scaffold_2151  CGTCCTGTTC TGGTTTGTTA GTCTATGTCT GTCTTTTTTG TTTTGCTTCT GCTTTATTGT 300
     scaffold_7237  CGTCCTGTTC TGGTTTGTTA GTCTGTGTCT GTCTTTTTTG TTTTGCTTCT GCTTTATTGT 300
    scaffold_12163  CGTCCTGTTC TGGTTTGTTA GTCTGTGTCT GTCTTTTTTG TTTTGCTTCT GCTTTATTGT 300
      scaffold_941  CGTCTTGTTT TGGTTTGTTA GTCTGTGTCT GTTTTTCTTG TGTTGCTTCT GCTTTACTGT 300
    scaffold_22661  CGTCTTGTTT TGGTTCGTTA GTCTGTGTCT GTTTTTCTTG TGTTGCTTCT GCTTTACTGT 300
     scaffold_7076  CGTCTTGTTT TGGTTCGTTA GTCTGTGTCT GTTTTTCTTG TGTTGCTTCT GCTTTACTGT 300
    scaffold_22354  CGTCTTGTTT TGGTTCGTTA GTCTGTGTCT GTTTTTCTTG TGTTGCTTCT GCTTTACTGT 300
    scaffold_14383  CGTCTTGTTT TGGTTCGTTA GTCTGTGTCT GTTTTTCTTG TGTTGCTTCT GCTTTACATT 300
                                             320                  340                  360
                                               |                    |                    |
Ancestral_Gamma_G1  GGAACTATGG GACAATCCAA ACTAACTCCG TTACAGTGCA TGCTTAACCA CTTTAAAGAT 360
 GeneScaffold_3363  GGAACTATGG GACAATCCAA ACTAACTCCG TTACAGTGCA TGCTTAACAA CTTTAAAGAT 360
     scaffold_1753  GGAACTATGG GACAATCCAA ACTAACTCCG TTACAGTGCA TGCTTAACAA CTTTAAAGAT 360
    scaffold_13050  GGAACTATAG GACAATCCAA ACTAACTCCG TTACAGTGCA TGCTTAACAA CTTTAAAGAT 360
    scaffold_24606  GGAACTATGG GACAATCCAA ACTAACTCCG TTACAGTGCA TGCTTAACAA CTTTAAAGAT 360
    scaffold_10119  GGAACTATGG GACAATCCAA ACTAACTCCG TTACAGTGCA TGCTTAACAA CTTTAAAGAT 360
    scaffold_23277  GGAACTATGG GACAATCCAA ACTAACTCCG TTACAGTGCA TGCTTAACAA CTTTAAAGAT 360
    scaffold_12630  GGAACTATGG GACAATCCAA ACTAACTCCG TTACAGTGCA TGCTTAACAA CTTTAAAGAT 360
     scaffold_2151  GGAACTATGG GACAATCCAA ACTAACTCCG TTACAGTGCA TGCCTAACAA CTTTAAAGAT 360
     scaffold_7237  GGAACTATGG GACAATCCAA ACTAACTCCG TTACAGTGCA TGCTTAACAA CTTTAAAGAT 360
    scaffold_12163  GGAACTATGG GACAATCCAA ACTAACTCCG TTAGAGTGCA TGCTTAACAA CTTTAAAGAT 360
      scaffold_941  GGAACTATGG GACAATCTAA GCTAACACCG CTTCGATGCA TGCTTAATCA CTTTAAAGAT 360
    scaffold_22661  GGAACTATGG GACAATCTAA GCTAACACCG CTTCAATGCA TGCTTAATCA CTTTAAAGAT 360
     scaffold_7076  GGAACTATGG GACAATCTAA GCTAACACCG CTTCAATGCA TGCTTAATCA CTTTAAAGAT 360
    scaffold_22354  GGAACTATGG GACAATCTAA GCTAACACCG CTTCAATGCA TGCTTAATCA CTTTAAAGAT 360
    scaffold_14383  GGAACTATGG GACAATCTAA GCTAACACCG CATCAATGCA TGCTTAATCA CTTTAAAGAT 360
                                             380                  400                  420
                                               |                    |                    |
Ancestral_Gamma_G1  TTTCGTCGTA GGGCAAGGGC CTATGGTGCT TCAGTAACCC CTTTTGATTT GCAGCGCTTT 420
 GeneScaffold_3363  TTTCGTCGTA GGGCAAGGGC CTATGGTGCT TCAGTAACCC CTTTTGATTT GCAGCGCTTT 420
     scaffold_1753  TTTCATCGTA GGGCAAGGGC CTATGGTGCT TCAGTAACCC CTTTTGATTT GCAGCGCTTT 420
    scaffold_13050  TTTCGTTGTA GGGCAAGGGC CTATGGTGCT TCAGTAACCC CTTTTGATTT GCAGCGCTTT 420
    scaffold_24606  TTTCGTCGTA GGGCAAGGGC CTATGGTGCT TCAGTAACCC CTTTTGATTT GCAGCACTTT 420
    scaffold_10119  TTTCATCGTA GGGCAAGGGC CTATGGTGCT TCAGTAACCC CTTTTGATTT GCAGCGCTTT 420
    scaffold_23277  TTTCGTCGTA GGGCAAGGGC CTATGGTGCT TCAGTAACCC CTTTTGATTT GCAGCGCTTT 420
    scaffold_12630  TTTCGTCGTA GGGCAAGGGC CTATGGTGCT TCAGTAACCC CTTTTGATTT GCAGCGCTTT 420
     scaffold_2151  TTTCGTTGTA GGGCAAGGGC CTATGGTGCT TCAGTAACCC CTTTTGATTT GCAGCGCTTT 420
     scaffold_7237  TTTCGTCGTA GGGCAAGGGC CTATGGTGCT TCAGTAACCC CTTTTGATTT GCAGCGCTTT 420
    scaffold_12163  TTTCGTCGTA GAGCAAGGGC CTATGGTGCT TCAGTAACCC CTTTTGATTT GCAGCGCTTT 420
      scaffold_941  TTCCGTCACA GGGCAAAGGC TTATGGTGCT TTAGTAACCC CTTTTGATTT GCAGCGGTTT 420
    scaffold_22661  TTCCGTCACA GGGCAAAGGC TTATGGTGCT TTAGTAACCC CTTTTGATTT GCAGCGGTTT 420
     scaffold_7076  TTCCGTCACA GGGCAAAGGC TTATGGTGCT TTAGTAACCC CTTTTGATTT GCAGCGGTTT 420
    scaffold_22354  TTCCGTCACA GGGCAAAGGC TTATGGTTCT TTAGTAACCC CTTTTGATTT GCAGCGGTTT 420
    scaffold_14383  TTCCGTCACA GGGCAAAGGC TTATGGTGCT TTAGTAACCC CTTTTGATTT GCAGCGGTTT 420
                                             440                  460                  480
                                               |                    |                    |
Ancestral_Gamma_G1  TGTCAATTAG ATTGGCCGAC CTTCGGAGTA GGATGGCCCT CCGAGGGATC ATTTAACATG 480
 GeneScaffold_3363  TGTCAATTAG ATTGGCCGAC CTTCGGAGTA GGATGGCCCT CCGAGGGATC ATTTAACATG 480
     scaffold_1753  TGTCAATTAG ATTGGCCGAC CTTCGGAGTA GGATGGCCCT CCGAGGGATC ATTTAACATG 480
    scaffold_13050  TGTCAATTAG ATTGGCCGAC CTTCGGAGTA GGATGGCCCT CCGAGGGATC ATTTAACATG 480
    scaffold_24606  TGTCAATTAG ATTGGCCGAC CTTCGGAGTA GGATGGCCCT CCGAGGGATC ATTTAACATG 480
    scaffold_10119  TGTCGATTAG ATTGGCCGAC CTTCGGAGTA GGATGGCCCT CCGAGGGATC ATTTAACATG 480
    scaffold_23277  TGTCCATTAG ATTGGCCGAC CTTCGGAGTA GGATGGCCCT CCGAGGGATC ATTTAACATG 480
    scaffold_12630  TGTCAATTAG ATTGGCCGAC CTTCGGAGTA GGATGGCCCT CCGAGGGATC ATTTAACATG 480
     scaffold_2151  TGTCAATTAG ATTGGCCGAC CTTCGGAGTA GGATGGCCCT CCGAGGGATC ATTTAACATG 480
     scaffold_7237  TGTCAATTAG ATTGGCCGAC CTTCGGAGTA GGATGGCCCT CCGAGGGATC ATTTAACATG 480
    scaffold_12163  TGTCAATTAG ATTGGCCGAC CTTCGGAGTA GGATGGCCCT CCGAGGGATC ATTTAACATG 480
      scaffold_941  TGTCAACTAG ATTGGCCGAC CTTCGGAGTA GGATGGCCCT CCGAGGGATC ATTTAATTTA 480
    scaffold_22661  TGTCAACTAG ATTGGCCGAC CTTTGGAGTA GGATGGCCCT CCGAGGGATC ATTTAATTTA 480
     scaffold_7076  TGTCAACTAG ATTGGCCGAC CTTCGGAGTA GGATGGCCCT CCGAGGGATC ATTTAATTTA 480
    scaffold_22354  TGTCAACTAG ATTGGCCGAC CTTCGGAGTA GGATGGCCCT CCGAGGGATC ATTTAATTTA 480
    scaffold_14383  TGTCAACTAG ATTGGCCGAC CTTCGGAGTA GGATGGCCCT CTGAGGGATC ATTTAATTTA 480
```

```
                                                  500                  520                  540
                                                    |                    |                    |
Ancestral_Gamma_G1  CAGACTGCAT TCCGGGTTCG AGGTGTGATT TATGGAAACC CTGGGCATCC TGACCAAATT 540
 GeneScaffold_3363  CAGACTGCAT TCCGGGTTCG AAGTGTGATT TATGGAAAAC CTGGGCATCC CGACCAAATT 540
     scaffold_1753  CAGACTGCAT TCTGGGTTCT AGGTGTGATT TATGGAAAAC CTGGGCATCC CGACCAAATT 540
    scaffold_13050  CAGACTGCAT TCCGGGTTTG AGGTGTGATT TATGGAAACC CTGGGCATCC TGACCAAATT 540
    scaffold_24606  CAGACTGCAT TCCGGGTTCG AGGTGTGATT TATAGAAACC CTGGGCATCC TGACCAAATT 540
    scaffold_10119  CAGACTGCAT TCCGGGTTCG AAGTGTGATT TATGGAAAAC CTGGGCATCC CGACCAAATT 540
    scaffold_23277  CAGACTGCAT TCCGGGTTCG AGGTGTGATT TATGGAAAAC CTGGGCATCC CGACCAAATT 540
    scaffold_12630  CAGACTGCAT TCCGGGTTCG AGGTGTGATT TATGGAAACC CTGGGCATCC TGACCAAATT 540
     scaffold_2151  CAGACTGCAT TCCGGGTTCG AGGTGTGATT TATGGAAACC CTGGGCATCC TGACCAAATT 540
     scaffold_7237  CAGACTGCAT TCCGGGTTCG AAGTGTGATT TATGGAAAAC CTGGGCATCC CGACCAAATT 540
    scaffold_12163  CAGACTGCAT TCCGGGTTCG AGGTGTGATT TATGGAAATC CTGGGCATCC TGACCAAATT 540
      scaffold_941  TAGACTGCAT TTCGGGTTCG GGGTGTTGTT TGTGGGAACC CGGGGCATCC TGACCAAATT 540
    scaffold_22661  CAGACTGCAT TTCGGGTTCG GGGTGTTGTT TATGGGAACC CGGGGCATCC TGACCAAATT 540
     scaffold_7076  CAGACTGCAT TTCGGGTTCG GGGTGTTGTT TATGGGAACC CGGGGCATCC TGACCAAATT 540
    scaffold_22354  CAGACTGCAT TTCGGGTTCG GGGTGTTGTT TATGGGAACC CGGGGCATCC TGACCAAATT 540
    scaffold_14383  CAGACTGCAT TTCGGGTTCG GGGTGTTGTT TATGGGAACC CGGGGCATCC TGACCAAATT 540

                                                  560                  580                  600
                                                    |                    |                    |
Ancestral_Gamma_G1  CCTTATATTG ATGTTTGGAT TGATGTTGTT TCTGATGCCC CTAAATACTT ACAGAATTGC 600
 GeneScaffold_3363  CCTTATATTG ATGTTTGGAT TGATGTTGTT TCTGATGCCC CTAAATACTT ACAGAGTTGC 600
     scaffold_1753  CCTTATATTG ATGTTTGGAT TGATGTTGTT TCTGATGCCC CTAAATACTT ACAGAGTTGC 600
    scaffold_13050  CCTTATATTA ATGTTTGGAT TGATGTTGTT TCTGATGCCC CTAAATACTT ACAGAATTGC 600
    scaffold_24606  CCTTATGTTG ATGTTTGGAT TGATGTTGTT TCTGATGCCC CTAAATACTT ACAGAATTGC 600
    scaffold_10119  CCTTATGTTG ATGTTTGGAT TGATGTTGTT TCTGATGCCC CTAAATACTT ACAGAATTGC 600
    scaffold_23277  CCTTATGTTG ATGTTTGGAT TGATGTTGTT TCTGATGCCC CTAAATACTT ACAGAGTTGC 600
    scaffold_12630  CCTTATATTG ATGTTTGCAT TGATGTTGTT TCTGATGCCC CTAAGTACTT ACAGAGTTGC 600
     scaffold_2151  CCTTATATTG ATGTTTGGAT TGATGTTGTT TCTGATGCCC CTAAATACTT ACAGAATTGC 600
     scaffold_7237  CCTTATATTG ATGTTTGGAT TGATGTAGTT TCTGATGCCC CTAAATACTT ACAGAGTTGC 600
    scaffold_12163  CCTTATATTG ATGTTTGGAT TGATGTTGTT TCTGATGCCC CTAAATACTT ACAGAATTGC 600
      scaffold_941  CCTTATATTG ATGTATGGAT TGACGTTGTT TCTGATGCCC CTAAGTACTT ACAGAATTGC 600
    scaffold_22661  CCTTATATTG ATGTATGGAT TGACGTTGTT TCTGATGCCC CTAAGTACTT ACAGAATTGC 600
     scaffold_7076  CCTTATATTG ATGTATGGAT TGACGTTGTT TCTGATGCCC CTAAGTACTT ACAGAATTGC 600
    scaffold_22354  CCTTATATTG ATGTATGGAT TGACGTTGTT TCTGATGCCC CTAAGTACTT ACAGAATTGC 600
    scaffold_14383  CCTTATATTG ATGTATGGAT TGACGTTGTT TCTGATGCCC CTAAGTACTT ACAGAATTGT 600

                                                  620                  640                  660
                                                    |                    |                    |
Ancestral_Gamma_G1  AGTCGTGCAC CCGTTACTGT AATGGCAGCC ATACCCCAGG GTTTTAAGAA GCCCCCACCA 660
 GeneScaffold_3363  AGTCGTGCAC CCGTTACTGT AATGGCAGCC ATACCCCAGG GTTTTAAGAA GCCCCCACTG 660
     scaffold_1753  AGTTGTGCAC CCGTTACTGT AATGGCAGCC ATACCCCAGG GTTTTAAGAA GCCCCCACCG 660
    scaffold_13050  AGTCGTGCAC CCGTTACTGT AATGGCAGCC ATACCCCAGG GTTTTAAGAA GCCCGCACCG 660
    scaffold_24606  AGTCGTGCAC CCGTTACTGT AATGGCAGCC ATACCCCAGG GTTTTAAGAA GCCCCCACCG 660
    scaffold_10119  AGTCGTGCAC CCATTACTGT AATGGCAGCC ATATCCCAGG ATTTTAAGAA GCCCCCACCG 660
    scaffold_23277  AGTCGTGCAC CCGTTACTGT AATGGCAGCC ATACCCCAGG GTTTTAAGAA GCCCCCACTG 660
    scaffold_12630  AGTCGTGCAC CCGTTACTGT AATGGCAGCC ATACCCCAGG GTTTTAAGAA GCCCCCACCA 660
     scaffold_2151  AGTCGTGCAC CCGTTACTGT AATGGCAGCC ATACCCCAGG GTTTTAAGAA GCCCCCACCG 660
     scaffold_7237  AGTCGTGCAC CCGTTACTGT AATGGCAGCC ATACCCCAGA GTTTTAAAAA GCCCCCACCG 660
    scaffold_12163  AGTCGTGCAC CCGTTACTGT AATGGCAGCC ATACCCCAGG GTTTTAAGAA GCCCCCACCA 660
      scaffold_941  AGTCGCGCCC CCGTTACTGT AATGGCAGCC ATACCCCAAG GTCTTAAGAA GCCCCCACCA 660
    scaffold_22661  AGTCGCGCCC CCGTTACTGT AATGGCAGCC ATACCCCAAG GTCTTAAGAA GCCCCCACCA 660
     scaffold_7076  AGTCGCGCCC CCGTTACTGT AATGGCAGCC ATACCCCAAG GTCTTAAGAA GCCCCCACCA 660
    scaffold_22354  AGTCGCGCCC CCGTTACTGT AATGGCAGCC ATACCCCAAG GTCTTAAGAA GCCCCCACCA 660
    scaffold_14383  AGTCGCGCCC CCATTACTGT AATGGCAGCC ATACCCCAAG GTCTTAAGAA GCCCCCACCA 660

                                                  680                  700                  720
                                                    |                    |                    |
Ancestral_Gamma_G1  GTGTTGCAGG GACCTCCCTC GGAGGATGAC TGGTATCCGC CACCCTATAA TGGTAATGGG 720
 GeneScaffold_3363  GTGTTGCAGG GACCTCCCTC GGAGGATGAC TGGTATCCGC CATCCTATAA TGGAAATGGG 720
     scaffold_1753  GTGTTGCAGG GACCTCCCTC GGAGGATGGC TGGTATCCGC CACCCTTTAA TGGAAATGGG 720
    scaffold_13050  GTGTTGCAGG GACCTCCCTC GGAGGATAAC TGGTATCCGC CACCCTACAA TGGAAATGGG 720
    scaffold_24606  GTGTTGCAGG GGCCTCCCTC GGAGAATGAC TGGTATCCGC CACCCTATAA TGGAAATGGG 720
    scaffold_10119  GTGTTGCAGG GACCTCCCTC GGAGGATGAC TGGTATCCGC CACCCTATAA TGGAAATGGG 720
    scaffold_23277  GTGTTGCAGG GACCTCCCTC GGAGGATGGC TGGTATCCGC CACCCTATAA TGGAAATGGG 720
    scaffold_12630  GTGTTGCAGG GACCTCCCTC GGAGGATGGC TGGTATCCGC CACCCTATAA TGGAAATGGG 720
     scaffold_2151  GTGTTGCAGG GACCTCCCTC GGAGGATGAC TGGTATCCGC CACCCTATAA TGGAAATGGG 720
     scaffold_7237  GTGTTGCAGG GACCTCCCTC AGAGGATGAC TGGTATCCGC CACCCTATAA TGGAAATGGG 720
    scaffold_12163  GTGTTGCAGG GACCTCCCTC GGAGGATGAC TGGTATCCGC CACCCTATAA TGGAAATGGG 720
      scaffold_941  GTGTTGCAGG GCCCTCCGTC GGAGGATGAC TGGTATCCAC CACACCATCA TGGTGATGGG 720
    scaffold_22661  GTGTTGCAGG GCCCTCCGTC GGAGGATGAC TGGTATCCAC CACCCTATCA TGGTGACGGG 720
     scaffold_7076  GTGTTGCAGG GCCCTCCGTC GGAGGATGAC TGGTATCCAC CACCCTATCA TGGTGACGGG 720
    scaffold_22354  GTGTTGCAGG GCCCTCCGTC GGAGGATGAC TGGTATCCAC CACCCTATCA TGGTGATGGG 720
    scaffold_14383  GTGTTGCAGG GCCCTCCGTC GGAGGATGAC TGGTATCCAC CACCCTATCA TGGTGATGGG 720
```

```
                                                    740                    760                    780
                                                      |                      |                      |
Ancestral_Gamma_G1  GGTAGACGTC CTCCTCCGGA GGGACCGGGA CCTCCTGTAA CCCGGTCTAG GGGTCATAGG 780
 GeneScaffold_3363  GGTAGACATC CTCCTCCGGA GGGACCGGAA CCTCCTGTAA CCCGGTCTAG GGGTCGTAGG 780
     scaffold_1753  GGTAGACGTC CTCCTCCGGA GGGACCGGGA CCTCCTGTAA CCCGGTCTAG GGGTCATTGG 780
    scaffold_13050  GGTAGACGTC CTCCTCCGGA GGGACCGGAA CCTCCTGTAA CCTGGTCTAG GGGTCGTAGG 780
    scaffold_24606  GGTAGACGTC CTCCTCCGGA GGGATCGGGA CCTCCTGTAA CTCGGTCTAG GGGTCACAAG 780
    scaffold_10119  GGTAGACGTC CTCCTCCGGA GGGACCGGGA CCTCCTGTAA CCCGGTCTAG GGGTCACAAG 780
    scaffold_23277  GGTAGACGTC CTCCTCCGGA GGGACCGGGA CCTCCTGTAA CCCGGTCTAG GGATCATAGG 780
    scaffold_12630  GGTAGACGTC CTCCTCCGGA GGGACCAGGA CCTCCTGTAA CCCGGTCTAG GGGTCATAGG 780
     scaffold_2151  GGTAGACGTC CTCCTCCGGA GGGACCGGGA CCTCCTGTAA CCCGGTCTAG GGGTCATAGG 780
     scaffold_7237  GGTAGACGTC CTCCTCCGGA GGGACCGGGA CCTCCTGTAA CCCGGTCTAG GGATCATAGG 780
    scaffold_12163  GGTAGACGTC CTCCTCCGGA GGGACCGGGA CCTCCTGTAA CCCGGTCTAG GGGTCATAGG 780
      scaffold_941  AGTGGACGTC CTCCTCCGGA AAGACCAGAA CCTCCTGTAA CCTGGTCTAG GGGTCGTAGG 780
    scaffold_22661  AGTGGACGTC CTCCTCTGGA AAGACCAGAA CCTCCTGTAA CCTGGTCTAG GGGTCGTAGG 780
     scaffold_7076  AGTGGACGTC CTCCTCCGGA AAGACCAGAA CCTCCTGTAA CCCGGTCTAG GGGTCGTAGG 780
    scaffold_22354  AGTGGACGTC CTCCTCCGGA AAGACCAGAA CCTCCTGTAA CCCGGTCTAG GGGTCGTAGG 780
    scaffold_14383  AGTGGACGTC CTCCTCCGGA AAGACCAGAA CCTCCTGTAA CCCGGTCTAG GGGTCGTAGG 780
                                                    800                    820                    840
                                                      |                      |                      |
Ancestral_Gamma_G1  CCACCTTCCC CTGATTCAAA AGAGCCAGTA TCTGTTCAAG CCCCATTGAA GGTGATTCCA 840
 GeneScaffold_3363  CCACCTTCCC CTGATTCAAA AGAGCGAGTA TCTGTTCAAG CCCCATTGAA GGTGATTCCA 840
     scaffold_1753  CCACCTTCCC CTGATTCAAA AGAGCCAGTA TCTGTTCAAG CCCCATTGAA GGTGATTCCA 840
    scaffold_13050  CCACCTTCCC CTGATTCAAA AGAGCCAGTA TCTGTTCAAG CCCCATTGAA GGTGATTCCA 840
    scaffold_24606  CCACCTTCCT CTGATTCAAA AGAGCCAGTA TCTGTTCAAG CTCCATTGAA GGTGATTCCA 840
    scaffold_10119  CCACCTTCCC CTGATTCAAA AGAGCCAGTA TCTGTTCAAG CCCCATTGAA GGTGATTCCA 840
    scaffold_23277  CCACCTTCCC CTGATCAAAA AGAGCCAGTA TCTGTTCAAG CCCCATTGAA GGTGATTGCA 840
    scaffold_12630  CCACCTTCCC CTGATTCAAA AGAGCCAGTA TCTGTTCAAG CCCCATTGAA GGTGATTCCA 840
     scaffold_2151  CCACCTTCCC CTGATTCAAA AGAGCCAGTA TCTGTTCAAG CCCCATTGAA GGTGATTCCA 840
     scaffold_7237  CCACCTTCCC CTGATTCAAA AGAGCCAGTA TCTGTTCAAG CCCCATTGAA GGTGATTCCA 840
    scaffold_12163  CCACCTTCCC CTGATTCAAA AGAGCCAGTA TCTGTTCAAG CCCCATTGAA GGTGATTGCA 840
      scaffold_941  CCACCTTCCC CTGATTCAAA AGAGTCAGTA TCTGTTCAAG CCCCATTGAA GGTGATTCCA 840
    scaffold_22661  CCACCTTCCC CTGATTCAAA AGAGCCAGTA TCTGTTCAAG CCCCATTGAA GGTGATTCCA 840
     scaffold_7076  CCACCTTCCC CTGATTCAAA AGAGCCAGTA TCTGTTCAAG CCCCATTGAA GGTGATTCCA 840
    scaffold_22354  CCACCTTCCC CTGATTCAAA AGAGCCAGTA TCTGTTCAAG CCCCATTGAA GGTGATTCCA 840
    scaffold_14383  CCACCTTCCC CTGATTCAAA AGAGCCAGTA TCTGTTCAAG CCCCATTGAA GGTGATTCCA 840
                                                    860                    880                    900
                                                      |                      |                      |
Ancestral_Gamma_G1  GGAGGTGGCG GTGAGCCTAC TGTCATCTAC TCGCCATTCA GTACTAGTGA TTTATATAAT 900
 GeneScaffold_3363  GGAGGTGGAG GTGAGCCTAC TGTCATCTAC TCGCCATTCA GTACTAGTGA TTTATATAAT 900
     scaffold_1753  GGAGGTGGCG GTGAGCCTAC TGTCATCTAC TCGCCATTCA GTACTAGTGA TTTATATAAT 900
    scaffold_13050  GGAGGTGGAG GTGAGCCTAC TGTCATCTAC TCGCCATTCA GTACTAGTGA TTTATATAAT 900
    scaffold_24606  GGAGGTGGCG GTGAGCCTAC TGTCATCTAC TCGCCATTCA GTACTAGTGA TTTATATAAT 900
    scaffold_10119  GGAGGTGGCG GTGAGCCTAC TGTCATCTAC TCGCCATTCA GTACTAGTGA TTTATATAAT 900
    scaffold_23277  GGAGGTGGCG GTGAGCCTAC TGTCATCTAC TCGCCATTCA GTACTAGTGA TTTATATAAT 900
    scaffold_12630  GGAGGTGGCG GTGAGCCTAC TGTCATCTAC TCGCCATTCA GTACTAGTGA TTTATATAAT 900
     scaffold_2151  GGAGGTGGCG GTGAGCCTAC TGTCATCTAC TCGCCATTCA GTACTAGTGA TTTATATAAT 900
     scaffold_7237  GGAGGTGGCG GTGAGCCTAC TGTCATCTAC TTGCCATTCA GTACTAGTGA TTTATATAAT 900
    scaffold_12163  GGAGGTGGCG GTGAGCCTAC TGTCATCTAC TCGCCATTCA GTACTAGTGA TTTATATAAT 900
      scaffold_941  GGAGGTGGAG GTGAGCCTAC TGTCATCTAC TCGCCGTTCA GTACTAGTGA TTTATATAAT 900
    scaffold_22661  GGAGGTGGAG GTGAGCCTAC TGTCATCTAC TCGCCATTCA GTACTAGTGA TTTATATAAT 900
     scaffold_7076  GGAGGTGGAG GTGAGCCTAC TGTCATCTAC TCGCCATTCA GTACTAGTGA TTTATATAAT 900
    scaffold_22354  GGAGGTGGAG GTGAGCCTAC TGTCATCTAC TCGCCGTTCA GTACTAGTGA TTTATATAAT 900
    scaffold_14383  GGAGGTGGAG GTGAGCCTAC TGTCATCTAC TCGCCATTCA GTACTAGTGA TTTATATAAT 900
                                                    920                    940                    960
                                                      |                      |                      |
Ancestral_Gamma_G1  TGGAAATTGC AAACGCCATC CTTTTCAGAG AAACCACAAG GACTAACCTC CCTTTTAGAG 960
 GeneScaffold_3363  TGGAAATTGC AAACGCCATC CTTTTCAGAG AAACCACAAG GACTAACCTC CCTTTTAGAG 960
     scaffold_1753  TGGAAATTGC AAACGCCATC CTTTTCAGAG AAACCATAAG GACTAACCTC CCTTTTAGAG 960
    scaffold_13050  TGGAAATTGC AAACGCCATC CTTTTCAGAG AAACCACAAG GACTAACCTC CCTTTTAGAG 960
    scaffold_24606  TGGAAATTGC AAACGCCATC CTTTTCAGAG AAACCATAAG GACTAACCTC CCTTTTAGAG 960
    scaffold_10119  TGGAAATTGC AAACGCCATC CTTTTCAGAG AAACCACAAG GACTAACCTC CCTTTTAGAG 960
    scaffold_23277  TGGAAATTGC AAACGCCATC CTTTTCAGAG AAACCACAAG GACTAACCTC CCTTTTAGAG 960
    scaffold_12630  TGGAAATTGC AAACGCCATC CTTTTCAGAG AAACCATAAG GACTAACCTC CCTTTTAGAG 960
     scaffold_2151  TGGAAATTGC AAACGCCATC CTTTTCAGAG AAACCACAAG GACTAACCTC CCTTTTAGAG 960
     scaffold_7237  TGGAAATTGC AAACGCCATC CTTTTCAGAG AAACCATAAG GACTAACCTC CCTTTTAGAG 960
    scaffold_12163  TGGAAATTGC AAACGCCATC CTTTTCAGAG AAACCACAAG GACTAACCTC CCTTTTAGAG 960
      scaffold_941  TGGAAATTGC AAACGCCATC CTTTTCAGAA AAACCACAAG GATTAACCTC CCTTCTAGAG 960
    scaffold_22661  TGGAAATTGC AAACGCCATC CTTTTCAGAA AAACCACAAG GATTAACCTC CCTTCTAGAG 960
     scaffold_7076  TGGAAATTGC AAACGCCATC CTTTTCAGAA AAACCACAAG GATTAACCTC CCTTCTAGAG 960
    scaffold_22354  TGGAAATTGC AAACGCCATC CTTTTCAGAA AAACCACAAG GATTAACCTG CCTTCTAGAG 960
    scaffold_14383  TGGAAATTGC AAATGCCATC CTTTTCAGAA AAACCACAAG GATTAACCTG CCTTCTAGAG 960
```

```
                                                    980                          1,000                         1,020
                                                      |                            |                             |
Ancestral_Gamma_G1  TCTATTTTCT  TACCCATCAG  CCCACTTGGG  ATGATTGCCA  ACAGCTCTTA  CAGGTCTTAT  1020
 GeneScaffold_3363  TCTATTTTCT  TACCCATCAG  CCCACTTGGG  ATGATTGCCA  GCAGCTTTTA  CAGATTTTAT  1020
     scaffold_1753  TCTATTTTCT  TACCCATCAG  CCCACTTGGG  ATGATTGCCA  ACAGCTCTTA  CAGGTCTTAT  1020
    scaffold_13050  TCTATTTTCT  TACCCATCAG  CCCACTTGGG  ATGATTGCCA  ACAGCTCTTA  CAGGTCTTAT  1020
    scaffold_24606  TCTATTTTCT  TACCCATCAG  CCCACTTGGG  ATGATTTCCA  ACAGCTCTTA  CAGGTCTTAT  1020
    scaffold_10119  TCTATTTTCT  TACCCATCAG  CCCACTTGGG  ATGATTGCCA  ACAGCTCTTA  CAGGTCTTAT  1020
    scaffold_23277  TCTATTTTCT  TACCCATCAG  CCCACTTAGG  ATGATTGCCA  ACAGCTCTTA  CAGGTCTTAT  1020
    scaffold_12630  TCTATTTTCT  TACCCATCAG  CCCACTTGGG  ATGATTGCCA  ACAGCTCTTA  CAGGTCTTAT  1020
     scaffold_2151  TCTATTTTCT  TACCCATCAG  CCCACTTGGG  ATGATTGCCA  ACAGCTCTTA  CAGGTCTTAT  1020
     scaffold_7237  TCTATTTTCT  TACCCATCAG  CCCACTTGGG  ATGATTTCCA  ACAGCTCTTA  CAGGTCTTAT  1020
    scaffold_12163  TCTATTTTCT  TACCCATCAG  CCCACTTGGG  ATGATTGCCA  ACAGCTCTTA  CAGGTCTTAT  1020
      scaffold_941  TCTATTTTTT  TACCCATCAG  CCCACTTGGG  ATGATTGTCA  GCAGCTTTTG  CCGGTTTTAT  1020
    scaffold_22661  TCTATTTTTT  TACCCATCAG  CCCACTTGGG  ATGATTGTCA  GCAGCTTTTG  CCGGTTTTAT  1020
     scaffold_7076  TCTATTTTTT  TACCCATCAG  CCCACTTGGG  ATGATTGTCA  GCAGCTTTTG  CAGGTTTTAT  1020
    scaffold_22354  TCTATTTTTT  TACCCATCAG  CCCACTTGGG  ATGATTGTCA  GCAGCTTTTG  CAGGTTTTAT  1020
    scaffold_14383  TCTATTTTTT  TACCCATCAG  CCCACTTGGG  ATGATTGTCA  GCAGCTTTTG  CAGGTTTTAT  1020

                                                  1,040                          1,060                         1,080
                                                      |                            |                             |
Ancestral_Gamma_G1  TCACTACTGA  AGAAAAGGAA  AGAATTTTGC  GGGAGGCGGC  TAGAGGAGTA  ACAGACCCAA  1080
 GeneScaffold_3363  TCACTACTGG  AGAAAAGGAA  AGAATTTTGC  GGGAGGCGGC  TAGAGGAGTA  ACAGACCCAA  1080
     scaffold_1753  TCACTACTGG  AGAAAAGGAA  AGAATTTTGC  GGGAGGCGGC  TAGAGGAGTA  ACAGACCCAA  1080
    scaffold_13050  TCACTACTGA  AGAAAAGGAA  AGAATTTTGC  GGGAGGCGGC  TAGAGGAGTA  ACAAACCCAA  1080
    scaffold_24606  TCACTACTGA  AGAAAAGGAA  AGAATTTTGC  GGGAGGCGGC  TAGAGGAGTG  ACAAACCCAA  1080
    scaffold_10119  TCACTACTGA  AGAAAAGGAA  AGAATTTTGC  GGGAGGCGGC  TAGAGGAGTA  ACAAACCCAA  1080
    scaffold_23277  TCACTACTGA  AGAAAAGGAA  AGAATTTTGC  GGGAGGCGGC  TAGAGGAGTA  ACAAACCCAA  1080
    scaffold_12630  TCACTACTGA  AGAAAAGGAA  AGAATTTTGC  GGGAGGCGGC  TAGAGGAATA  ACAGACCCAA  1080
     scaffold_2151  TCACTACTGA  AGAAAAGGAA  AGAATTTTGC  GGGAGGCGGC  TAGAGGAGTA  ACAAACCCAA  1080
     scaffold_7237  TCACTACTGA  AGAAAAGGAA  AGAATTTTGC  GGGAGGCGGC  TAGAGGAGTA  ACAAACCCAA  1080
    scaffold_12163  TCACTACTGA  AGAAAAGGAA  AGAATTTTGC  GGGAGGCGGC  TAGAGGAGTA  ACAGACCCAA  1080
      scaffold_941  TCACCACTGA  GGAAAAAGAA  AGGATTTTGC  GGGAGGCAGC  CAGAGGAGTC  ACGGACCCGA  1080
    scaffold_22661  TCACCACTGA  GGAAAAAGAA  AGGATTTTGC  GGGAGGCAGC  CAGAGGAGTC  ACGGACCCGA  1080
     scaffold_7076  TCACCACTGA  GGAAAAAGAA  AGGATTTTGC  GGGAGGCAGC  CAGAGGAGTC  ACGGACCCGA  1080
    scaffold_22354  TCACCACTGA  GGAAAAAGAA  AGGATTTTGC  GGGAGGCAGC  CAGAGAAGTC  ACGGACCCGA  1080
    scaffold_14383  TCACCACTGA  GGAAAAAGAA  AGGATTTTGC  GGGAGGCAGC  CAGAGGAGTC  ACGGACCCGA  1080

                                                  1,100                          1,120                         1,140
                                                      |                            |                             |
Ancestral_Gamma_G1  ATGGGCAGCC  TACAGCAGAC  TTAACCAGGT  TACAGGCTGT  GTTTCCGACT  CAACGTCCCC  1140
 GeneScaffold_3363  ATGGGCAGCC  CACAGCAGAC  TTAACCAGGT  TACAGGCTGT  GTTTCCGACT  CAACGTCCCC  1140
     scaffold_1753  ATGGGCAGCC  CACAGCAGAC  TTAACCAGGT  TACAGGCTGT  GTTTCCGACT  CAACGTCCCC  1140
    scaffold_13050  ATGGGCAGCC  TACAGCAGAC  TTAACCAGGG  TACAGGCTGT  GTTTCCGACT  CAACGTCCCC  1140
    scaffold_24606  ATGGGCAGCC  TACAGCAGAC  TTAACCAGGT  TACAGGCTGT  GTTTCCGACT  CAACGTCCCC  1140
    scaffold_10119  ATGGGCAGCC  TACAGCAGAC  TTAACCAGGG  TACAGGCTGT  GTTTCCGATT  CAACGTCCCC  1140
    scaffold_23277  ATGGGCAGCC  TACAGCAGAC  TTAACCAGGT  TACAGGCTGT  GTTTCCGACT  CAACGTCCCC  1140
    scaffold_12630  ATGGGCAGCC  CACAGCAGAC  TTAACCAGGT  TACAGGCTGT  GTTTCCGACT  CAACGTCCCC  1140
     scaffold_2151  ATGGGCAGCC  TACAGCAGAC  TTAACCAGGG  TACAGGCTGT  GTTTCCGACT  CAACGTCCCC  1140
     scaffold_7237  ATGGGCAGCC  TACAGCAGAC  TTAACCAGGT  TACAGGCTGT  GTTTCCGACT  CAACGTCCCC  1140
    scaffold_12163  ATGGGCAGCC  TACAGCAGAC  TTAACCAGGT  TACAGGCTGT  GTTTCCGACT  CAACGTCCCC  1140
      scaffold_941  ATGGGCTGCC  AACAGCAGAC  TTAACCAGGA  TACAGGCTGT  GTTTCCTACT  CAGCATCCCC  1140
    scaffold_22661  ATGGGCTGCC  AACAGCAGAC  TTAACCAGGA  TACAGCCTGT  GTTTCCTACT  CAGCATCCCC  1140
     scaffold_7076  ATGGGCTGTC  AACAGCAGAC  TTAACCAGGA  TACAGGCTGT  GTTTCCTACT  CAGCGTCCCC  1140
    scaffold_22354  ATGGGCTGCC  AACAGCAGAC  TTAACCAGGA  TACAGGCTGT  GTTTCCTACT  CAGCGTCCCC  1140
    scaffold_14383  ATGGGCTGCC  AACAGCAGAC  TTAACCAGGA  TACAGGCTGT  GTTTCCTACT  CAGCGTCCCC  1140

                                                  1,160                          1,180                         1,200
                                                      |                            |                             |
Ancestral_Gamma_G1  AGTGGGATCC  CAATACGGAT  CAAGGTAAGG  AGGCTCTCCT  AAGGTATCGC  CAGTTCTGCT  1200
 GeneScaffold_3363  AGTGGGATCC  AAATACAGAT  CAAGGTAAGG  AGGCTCTCTA  AGTGTATCGC  CAGTTCTGCT  1200
     scaffold_1753  AGTGGGATCC  AAATACAGAT  CAAGGTAAGG  AGGCTCTCCT  AAGGTATCGC  CAGTTCTGCT  1200
    scaffold_13050  AGTGGGATCC  AAATACAGAT  CGAGGTAAGG  AGGCTCTCCT  AAGGTATCGC  CAGTTTTGCT  1200
    scaffold_24606  AGTGGGATCC  AAATACAGAT  CAAGGTAAGG  AGGCTCTCCT  AAGGTATTGC  CAGTTCTGCT  1200
    scaffold_10119  AGTGGGATCC  AAATACAGAT  CAAGGTAAGG  AGGCTCTCCT  AAGGTATCGC  CAGTTCTGCT  1200
    scaffold_23277  AGTGGGATCC  AAATACAGAT  GAAGGTAAGG  AGGCTCTCCT  AAGGTATCGC  CAGTTCTGCT  1200
    scaffold_12630  AGTGGGATCC  AAATACAGAT  CGAGGTAAGG  AGGCTCTCCT  AAGGTATTGC  CAGTTCTGCT  1200
     scaffold_2151  AGTGGGATCC  AAATACAGAT  CAAGGTAAGG  AGGCTCTCCT  AAGGTATCGC  CAGTTCTGCT  1200
     scaffold_7237  AGTGGGATCC  AAATGCAGAT  CAAGGTAAGG  AGGCTCTCCT  AAGATATCGC  CAGTTCTGCT  1200
    scaffold_12163  AGTGGGATCC  AAATACGGAT  CAAGGTAAGG  AGGCTCTCCT  AAGGTATTGC  CAGTTCTGCT  1200
      scaffold_941  AGTGGGATCC  CAATACGGAT  CAAGGTAAGG  AGGCTCTCCT  AAGGTATCGC  AAGTTCTGCT  1200
    scaffold_22661  AGTGGGATCC  CAATACAGAT  CAAGGTAAGG  AGGCTCTCCT  AAGGTATCGC  CAGTTCTGCT  1200
     scaffold_7076  AGTGGGATCC  CAATACGGAT  CAAGGTAAGG  AGGCTCTCCT  AAGGTATCGC  CAGTTCTGCT  1200
    scaffold_22354  AGTGGGATCC  CAATACGGAT  CAAGGTAAGG  AGGCTCTCCT  AAGGTATCGC  CAGTTCTGCT  1200
    scaffold_14383  AGTGGGATCC  CAATACGGAT  CAAGGTAAGG  AGGCTCTCCT  AAGGTATCGC  CAGTTCTGCT  1200
```

```
                                         1,220                         1,240                   1,260
                                             |                             |                       |
Ancestral_Gamma_G1  ACAGGGACTC  CGGGCTGCTG  CACGTAGACC  AATAAATCTG  TCCAAGGTAA  GTGAAGTAAT  1260
 GeneScaffold_3363  GCAGGGACTC  CGAGCTGCTG  CACGTAGACC  AATAAATCTG  TCCAAGGTAA  GTGAAGTAAT  1260
     scaffold_1753  ACAGGGACTC  CGGGCTGCTG  CACGTAGACC  AATAAATCTG  TCCAAGGTAA  GTGAAGTAAT  1260
    scaffold_13050  ACAGGGACTC  CGGGGTGCTG  CACGTAGACC  AATAAATCTG  TCCAAGGTAA  GTGAAGTAAT  1260
    scaffold_24606  GCAGGGACTC  CGGGCTGCTG  CACGTAGACC  AATACATCTG  TCCAAGGTAA  GTGAAGTAAT  1260
    scaffold_10119  ACAGGGACTC  CGGGCTGCTG  CACGTAGACC  AATAAATCTG  TCCAAGGTAA  GTGAAGTAAT  1260
    scaffold_23277  ACAGGGACTC  CGGGCTGCTG  CACGTAGACC  AATAAATCTG  TCCAAGGTAA  GTGAAGTAAT  1260
    scaffold_12630  GCAGGGACTC  CGGGCTGCTG  CACGTAGACC  AATAAATCTG  TCCAAGGTAA  GTGAAGTAAT  1260
     scaffold_2151  ACAGGGACTC  CGGGCTGCTG  CACGTAGACC  AATAAATCTG  TCCAAGGTAA  GTGAAGTAAT  1260
     scaffold_7237  ACAGGGACTC  CGGGCTGCTG  CACGTAGACC  AATAAATCTG  TCCAAGGTAA  GTGAAGTAAT  1260
    scaffold_12163  GCAGGGACTC  CGGGCTGCTG  CACGTAGACC  AATAAATCTG  TCCAAGGTAA  GTGAAGTAAT  1260
      scaffold_941  ACAGGGACTC  CGGGCTGCTG  CACGTAGACC  AATAAATCTG  TCCAAGGTAA  GCGAAGTAAT  1260
    scaffold_22661  ACAGGGACTC  CGGGCTGCTG  CACGTAGACC  AATAAATCTG  TCCAAGGTAA  GCGAAGTAAT  1260
     scaffold_7076  ACAGGGACTC  CGGGCTGCTG  CACGTAGACC  AATAAATCTG  TCCAAGGTAA  GCAAAGTAAT  1260
    scaffold_22354  ACAGGGACTC  TGGGCTGCTG  CACGTAGACC  AATAAATCTG  TCCAAGGTAA  GCAAAGTAAT  1260
    scaffold_14383  ACAGGGACTC  CGGGCTGCTG  CACGTAGACC  AATAAATCTG  TCCAAGGTAA  GCGAAGTAAT  1260

                                         1,280                         1,300                   1,320
                                             |                             |                       |
Ancestral_Gamma_G1  TCAGACTAAG  GACGAGTCAC  CTGCAGCATT  CTTGGAAAGA  CTATTAGAGT  CTTACCGCTT  1320
 GeneScaffold_3363  TCAGACTAAG  GACGAGTCAC  CTGCAGCATT  CTTGGTAAGA  CTATTAGAGT  CTTACCGCCT  1320
     scaffold_1753  TCAGACTAAG  GACGAGTCAC  CTGCAGCATT  CTTGGAAAGA  CTATTAGAGT  CTTACCGCCT  1320
    scaffold_13050  TCAGAATAAG  GACGAGTCAC  CTGCAGTATT  TTTGGAAAGA  CTATTAGAGT  TTTACCGCCT  1320
    scaffold_24606  TCAGACTAAG  GACGAGTCAC  CTGCAGCATT  CTTGGAAAGA  CTATTAGAGT  CTTACCGCCT  1320
    scaffold_10119  TCAGACTAAG  GACGAGTCAC  CTGCAGCATT  CTTGGAAAGA  CTATTAGAGT  CTTACCGCCT  1320
    scaffold_23277  TCAGACTAAG  GACGAGTCAC  CTGCAGCATT  CTTGGAAAGA  CTATTAGAGT  CTTACCGCCT  1320
    scaffold_12630  TCAGACTAAG  GACGAGTCAC  CTGCAGCATT  CTTGGAAAGA  CTATTAGAGT  CTTACCGCCT  1320
     scaffold_2151  TCAGACTAAG  GACGAGTCAC  CTGCAGCATT  CTTGGAAAGA  CTATTGAAGT  CTTACCGCTT  1320
     scaffold_7237  TCAGACTAAG  GACGAGTCAC  CTGCAGCATT  CTTGGAAAGA  CTATTAAAGT  CTTACCGCTT  1320
    scaffold_12163  TCAGACTAAG  GACGAGTCGC  CTGCAGCATT  CTTGGAAAGA  CTATTAGAGT  CTTACCGCTT  1320
      scaffold_941  TCAGACCAAG  GATGAGTCAC  CTGCAGCATT  TTTAGAGAGA  CTATTAGAAT  CTTACCGCTT  1320
    scaffold_22661  TCAGACCAAG  GATGAGTCAC  CTGCAGCATT  TTTAGAGAGA  CTATTAGAAT  CTTACCGCTT  1320
     scaffold_7076  TCAGACCAAG  GATGAGTCAC  CTGCAGCATT  TTTAGAGAGA  CTATTAGAAT  CTTACCGCTT  1320
    scaffold_22354  TCAGACCAAG  GATGAGTCAC  CTGCAGCATT  TTTAGAGAGA  CTATTAGAAT  CTTACCGCTT  1320
    scaffold_14383  TCAGACCAAG  GATGAGTCAC  CTGCAGCATT  TTTAGAGAGA  CTATTAGAAT  CTTACCGCTT  1320

                                         1,340                         1,360                   1,380
                                             |                             |                       |
Ancestral_Gamma_G1  ATATACACCT  ATTGACCCGG  AGGACCCCAA  CAATAGAAGA  GCCATTAATT  TGGCCTTTGT  1380
 GeneScaffold_3363  ATATACACCT  ATTGACCCGG  AGGACCCCAA  CAATAGAAGA  GCCATTAATT  TGGCCTTTGT  1380
     scaffold_1753  ATATACACCT  ATTGACCCGG  AGGACCCCAA  CAATAGAAGA  GCCATTAATT  TGGCCTTTGT  1380
    scaffold_13050  ATATACACCT  ATTGACCCGG  AGGACCCCAA  CAATAGAAGA  GCCATTAATT  TGGCCTTTAT  1380
    scaffold_24606  ATATACACCT  ATTGACCCGG  AGGACCCCAA  CAATAGAAGA  GCCATTAATT  TGGCCTTTAT  1380
    scaffold_10119  ATATACACCT  ATTGATCCGG  AGGACCCCAA  CAATAGAAGA  GCCATTAATT  TGGCCTTTAT  1380
    scaffold_23277  ATATACACCT  ATTGACCCGG  AGGACCCCAA  CAATAGAAGA  GCCATTAATT  TGGCCTTTGT  1380
    scaffold_12630  ATATACACCT  ATTGACCCGG  AGGACCCCAA  CAATAGAAGA  GCCATTAATT  TGGCCTTTAT  1380
     scaffold_2151  ATATACACCT  ATTGACCCGG  AGGACCCCAA  CAATAGAAGA  GCCATTAATT  TGGCCTTTGT  1380
     scaffold_7237  ATATACACCT  ATTGACCCGG  AGGACCCCAA  CAATAGAAGA  GCCATTAATT  TGGCCTTTGT  1380
    scaffold_12163  ATATACACCT  ATTGACCCGG  AGGACCCCAA  CAATAGAAGA  GCCATTAATT  TGGCCTTTGT  1380
      scaffold_941  GTATACACTT  ATTGACCCGG  AGGACCCCAA  CAATAGAAGA  GCCATTAATT  TGGCCTTTGT  1380
    scaffold_22661  GTATACACCT  ATTGACCCGG  AGGACCCCAA  CAATAGAAGA  GCCATTAATT  TGGCCTTTGT  1380
     scaffold_7076  GTATACGCCT  ATTGACCCGG  AGGACCCCAA  CAATAGAAGA  GCCATTAATT  TGGCCTTTGT  1380
    scaffold_22354  GTATACACCT  ATTGACCCGG  AGGACCCCAA  CAATAGAAGA  GCCATTAATT  GGGCCTTTGT  1380
    scaffold_14383  GTATACACCT  ATTGACCCGG  AGGACCCCAA  CAATAGAAGA  GCCATTAATT  TGGCCTTTGT  1380

                                         1,400                         1,420                   1,440
                                             |                             |                       |
Ancestral_Gamma_G1  GAGCCAGTCG  GCCCCAGATA  TTAGAAAAAA  GTTACAGAAG  CTAGAAGGTT  TTGAGGGAGA  1440
 GeneScaffold_3363  TAGCCAGTCG  GCCCCTGACA  TTAGAAAGAA  GTTACAGAGA  TTGGAAGGTT  TTGAGGGAGA  1440
     scaffold_1753  TAGCCAGTCG  GCCCCTGACA  TTAGAAAGAA  GTTACAGAGA  TTGGAAGGTT  TTGAGGGAGA  1440
    scaffold_13050  TAGTCAGTCG  GCCCCTGACA  TTAGAAAGAA  GTTACAGAGA  TTGGAAGATT  TTGAGGGAGA  1440
    scaffold_24606  TAGCCAGTCG  GCCCCTGACA  TTAGAAAGAA  GTTACAGAGA  TTGGAAGGTT  TTGAGGGAGA  1440
    scaffold_10119  TAGCCAGTCG  GCCCCTGACA  TTAGAAAGAA  GTTACAGAGA  TTGGAAGGTT  TTGAGGGAGA  1440
    scaffold_23277  TAGCCAGTCG  GCCCCTGACA  TTAGAAAGAA  GTTACAGAGA  TTGGAAGGTT  TTGAGGGAGA  1440
    scaffold_12630  TAGCCAGTCG  GCCCCTGACA  TTAGAAAGAA  GTTACAGAGA  TTGGAAGGTT  TTGAGGGAGA  1440
     scaffold_2151  TAGTCAGTCG  GCCCCTGACA  TTAGAAAGAA  GTTACAGAGA  TTGGAAGGTT  TTGAGGGAGA  1440
     scaffold_7237  TAGCCAGTCG  GCCCCTGACA  TTAGAAAGAA  GTTACAGAGA  TTGGAAGGTT  TTGAGGGAGA  1440
    scaffold_12163  GAGCCAGTCG  GCCCCAGATA  TTAGAAAAAA  GTTACAAAAG  CTAGAAGGTT  TTGAGGGAGA  1440
      scaffold_941  GAGCCAGTCG  GCCCCAGATA  TTAGGAGAAA  GTTACAGAAG  CTAGAAGGTT  TTGAGGGAGA  1440
    scaffold_22661  GAGCCAGTCG  GCCCCAGATA  TTAGGAGAAA  GTTACAAAAG  CTAGAAGGTT  TTGAGGGAGA  1440
     scaffold_7076  GAGCCAGTCG  GCCCCAGATA  TTAGAAGAAA  GTTACAGAAG  CTAGAAGGTT  TTGAGAGAGA  1440
    scaffold_22354  GAGCCAGTCG  GCCCCAGATA  TTAGGAGAAA  GTTACAGAAG  CTAGAAGGTT  TTGAGGGAGA  1440
    scaffold_14383  GAGCCAGTCG  GCCCCAGATA  TTAGGAGAAA  GTTACAGAAG  CTAGAAGGTT  TTGAGGGAGA  1440
```

```
                                              1,460                      1,480                      1,500
                                                |                          |                          |
Ancestral_Gamma_G1  AAATTTATCC AAATTGCTAG AAATAGCCCA GAAAGTTTTC AACAATAGAG ACGATGTGCA 1500
 GeneScaffold_3363  AAATTTATCC AAATTATTAG AAATAGCTCA GAAGGTCTTT AACAATAGGG ACGGTGTGCA 1500
     scaffold_1753  AAATTTATCC AAATTATTAG AAATAGCTCA GAAGGTCTTT AACAATAGGG ACGGTGTGCA 1500
    scaffold_13050  AAATTTATTT AAATTATTAG AAATAGCTCA GAAGGTCTTT AACAATAGGG ACGGTGTGCA 1500
    scaffold_24606  AAATTTATCC AAATTATTAG AAATAGCTCA GAAGGTCTTT AACAATAGGG ACGGTGTGCA 1500
    scaffold_10119  AAGTTTATCC AAATTATTAG AAATAGCTCA GAAGGTCTTT AACAATAGGG ACGGTGTGCA 1500
    scaffold_23277  AAATTTATCC AAATTATTAG AAATAGCTCA GAAGGTCTTT AACAATAGGG ACGGTGTGCA 1500
    scaffold_12630  AAATTTATCC AAATTATTAG AAATAGCTCA GAAGGTCTTT AACAATAGGG ACGGTGTGCA 1500
     scaffold_2151  AAATTTATCC AAATTATTAG AAATAGCTCA GAAGGTCTTT AACAATAGGG ACGGTGTGCA 1500
     scaffold_7237  AAATTTATCC AAATTATTAG AAATAGCTCA GAAGGTCTTT AACAATAGGG ACGGTGTGCA 1500
    scaffold_12163  AAATTTATCC AAATTGCTAG AAATAGCCCA GAAAGTTTTC AACAATAGAG ACGATGTGCA 1500
      scaffold_941  GAATTTATCC AAATTGCTAG AAATAGTCCA GAGAGTTTTC AACAATAGAG ACGATGTGCA 1500
    scaffold_22661  GAATTTATCC AAATTGCTAG AAATAGCCCA GAGAGTTTTC AACAATAGAG ACGATGTGCA 1500
     scaffold_7076  GAATTTATCC AAATTGCTAG AAATAGCCCA GAAAGTTTTC AACAATAGAG ACGATGTGCA 1500
    scaffold_22354  GAATTTATCC AAATTGCTAG AAATAGCCCA GAAAGTTTTC AACAATAGAG ACGATGTGCA 1500
    scaffold_14383  GAATTTATCC AAATTGCTAG AAATAGCCCA GAAAGTTTTC AACAATAGAG ACGATGTGCA 1500

                                              1,520                      1,540                      1,560
                                                |                          |                          |
Ancestral_Gamma_G1  AAAAATAATG TTTGTGGGAA GGGACCTAGA GGAAGCCGAG GAGGATCAGG ATTTGTCACC 1560
 GeneScaffold_3363  AAAAGTCATG TTTGTGGGAA CCGACCTAAA AAAAACCCTA ACCATGGGGG ACCTAGCAGA 1560
     scaffold_1753  AAAGGTCATG CTTGTGGGAA CCGACCTAAA AAAAAACCTA ACCATGGGGG ACCTAGCAGA 1560
    scaffold_13050  AAAAGTCATG TTTGTGGGAA CCGACCTAAA AAAAACCCTA ACCATGTGGG ACCTAGCAGA 1560
    scaffold_24606  AAAGGTCATG TTTGTGGGAA CCGACCTAAA AAAAACCCTA ACCATGGGGG ACCTAGCAGA 1560
    scaffold_10119  AAAGGTCATG TTTGTGGGAA CCAACCTAAA AAAAACCCTA ACCATGGGGG ACCTAGCAGA 1560
    scaffold_23277  AAAAGTCATG TTTGTGGGAA CCGACCTAAA AAAAACCCTA ACCATGGAGG ACCTAGCAGA 1560
    scaffold_12630  AAAAGTCATG TTTGTGGGAA CCGACCTAAA AAAAACCCTA ACCATGGGGG ACCTAGCAGA 1560
     scaffold_2151  AAAAGTCATG TTTGTGGGAA CCGACCTAAA AAAAACCCTA ACCATGGGGG ACCTAGCAGA 1560
     scaffold_7237  AAAGGTCATG TTTGTGGGAA CCGACCTAAA AAAAACCCTA ACCATGGGGG ACCTAGCAGA 1560
    scaffold_12163  AAAAATAATG TTTGTGGGAA GGGACCTAGA GGAAGCCGAG GAGGATCAGG ATTTGTCACC 1560
      scaffold_941  GAAAATAATG TTTGTGGGAA CGGACCTAGA GGAAGCCGAG GAGGATCAGG ATTTGTCACC 1560
    scaffold_22661  GAAAATAATG TTTGTGGGAA GGGACCTAGA GGAAGCCGAG GAGGATCAGG ATTTGTCACC 1560
     scaffold_7076  GAAAATAATG TTTGTGGGAA GGGAACTAGA AGAAGCCGAG GAGGATCAGG ATTTGTCACC 1560
    scaffold_22354  GAAAATAATG TTTGTGGGAA GGGACCTAGA GGAAGCCGAG GAGGATCAGG ATTTGTCACC 1560
    scaffold_14383  GAAAATAATG TTTGTGGGAA GGGACCTAGA GGAAGCCGAG GAGGATCAGG ATTTGTCACC 1560

                                              1,580                      1,600                      1,620
                                                |                          |                          |
Ancestral_Gamma_G1  TGGACCAAAT AAACAGGTTG GCCTTGAAAA AGACCAATGT GCCTATTGCA AACAGAAAGG 1620
 GeneScaffold_3363  AGGCCAAAAT AGAAAACCTA CGCTAGGAAG AGACCAATGT GCTATTTGCA AAAAGAAAGG 1620
     scaffold_1753  AGGCCAAAAT AGAAAACCTA GGCTAGGAAG AGACCAATGT GCTATTTGCA AAAAGAAAGG 1620
    scaffold_13050  AGGCCAAAAT AGAAAACCTA CGCTAGGAAG AGACCAATGT GCTATTTGCA AAAAGAAAGG 1620
    scaffold_24606  AGGCCAAAAT AGAAAACCTA CGCTAGGAAG AGACCAATGT GCTATTTGCA AAAAGAAAGG 1620
    scaffold_10119  AGGCCAAAAT AGAAAACCTA GGCTAGGAAG AGACCAATGT GCTATTTGCA AAAAGAAAGG 1620
    scaffold_23277  AGGCCAAAAT AGAAAACCTA CGCTAGGAAG AGACCAATGT GCTATTTGCA AAAAGAAAGG 1620
    scaffold_12630  AGGCCAAAAT AGAAAACCTA GGCTAGGAAG AGACCAATGT GCTATTTGCA AAAAGAAAGG 1620
     scaffold_2151  AGGCCAAAAT AAAAAACCTA CGCTAGGAAG AGACCAATGT GCTATTTGCA AAAAGAAAGG 1620
     scaffold_7237  AGGCCAAAAT AGAAAACCTA GGCTAGGAAG AGACCAATGT GCTATTTGCA AAAAGAAAGG 1620
    scaffold_12163  TGGACCAAAT AAACAGGTTG GCCTTGAAAA AGACCAGTGT GCCTATTGCA GGCAGAAAGG 1620
      scaffold_941  TGGACCAGGT AAACAGGTTG GCCTTGAAAA AGACCAATGT GCATATTGCA AACAGAAAGG 1620
    scaffold_22661  TGGACCAGGT AAACAGGTTG GCCTTGAAAA AGACCAATGT GCATATTGCA AACAGAAAGG 1620
     scaffold_7076  TGGACCAGGT AAACAGGTTG GCCTTGAAAA AGACCAATGT GCATATTGCA AACAGAAAGG 1620
    scaffold_22354  TGGACCAGGT AAACAGGTTG GCCTTGAAAA AGACCAATGT GCATATTGCA AACAGAAAGG 1620
    scaffold_14383  TGGACCAGGT AAACAGGTTG GCCTTGAAAA AGACCAATGT GCATATTGCA AACATAAAGG 1620

                                              1,640                      1,660                      1,680
                                                |                          |                          |
Ancestral_Gamma_G1  GCATTGGAAT GAGTGTCCTA AGCAACAAGG AGGTAAAAAG ACAAGCTCCC TAATTCTTTT 1680
 GeneScaffold_3363  ACATTGGAAT GAGTGCCCTA AGGAGCAACC AGGAGAAGAA AAAAGGCCCC TCATTCTTTT 1680
     scaffold_1753  ACATTGGAAT GAGTGCCCTA AGGAGCAACC AGGAGAAGAA AAAAGGCCCC TCATTCTTTT 1680
    scaffold_13050  ACATTGGAAT GAGTGCCCTA AGGAACAACC AGGAGAAGAA AAAAGGCCCC TCATTCTTTT 1680
    scaffold_24606  ACATTGGAAT GAGTGCCCTA AGGAGCAACC AGGAGAAGAA AAAAGGCCCC TCATTCTTTT 1680
    scaffold_10119  ACATTGGAAT GAGTGCCCTA AGGAGCAACC AGGAGAAGAA AAAAGGCCCC TCATTCTTTT 1680
    scaffold_23277  ACATTGGAAT GAGTGCCCTA AGGAGCAACC AGGAGAAGAA AAAAGGCCCC TCATTCTTTT 1680
    scaffold_12630  ACATTGGAAT GAGTGCCCTA AGGAGCAACC AGGAGAAGAA AAAAGGCCCC TCATTCTTTT 1680
     scaffold_2151  ACATTGGAAT GAGTGCCCTA AGGAGCAACC AGGAGAAGAA AAAAGGCCCC TCATTCTTTT 1680
     scaffold_7237  ACATTGGAAT GAGTGCCCTA AGGAGCAACC AGGAGAAGAA AAAAGGCCCC TCATTCTTTT 1680
    scaffold_12163  GCATTGGAAT AAATGTCCTC GAACAGAAGG TGAAAAAAAG CCAGACTCCC TAATTCTTTT 1680
      scaffold_941  GCACTGGAAT GAGTGTCCCA AGCAACCAAA AGGTAAAAAT ACAAGCACCC CAATTCTTCT 1680
    scaffold_22661  GCACTGGAAT GAGTGTCCCA AGCAACCAAA AGGTAAAAAT ACAAGCACCC CAATTCTTCT 1680
     scaffold_7076  GCACTGGAAT GAGTGTCCCA ACCAACCAAA AGGTAAAAAT ACAAGCACCC CAATTCTTCT 1680
    scaffold_22354  GCACTGGAAT GAGTGTCCCA ACCAACCAAA AGGTAAAAAT ACAAGCACCC CAATTCTTCT 1680
    scaffold_14383  GCACTGGAAT GAGTGTCCCA ACCAACCAAA AGGTAAAAAT ACAAGCACCC CAATTCTTCT 1680
```

```
                                              1,700                 1,720                1,740
                                                |                     |                    |
Ancestral_Gamma_G1  GGAAGATGAC  TAGGGGTGTC  AGGGCTCTGA  CCCCGAGAGC  CCCGGATAAC  CTTACAAGTA  1740
 GeneScaffold_3363  GGAAGATGAA  TAGGGGCGTC  AGGGCTCTTA  CCCTGAGAGC  CCAAGGTAAC  TTTACAAGTA  1740
     scaffold_1753  GGAAGATGAA  TAAGGATGTC  AGGGCTCTTA  CCCCAAGAGC  CCAAGGTAAC  TTTGCAAGTA  1740
    scaffold_13050  GGAGGATGAA  TAGGGGCGTC  AGGGCTCTTA  CCCTGAGAGC  CCAAGGTAAC  TTTACAAGTA  1740
    scaffold_24606  GGAAGATGAA  TAGGGGCGTC  AGGGCTCTTA  CCCCGAGAGC  CCAAGGTAAC  TTTACAAGTA  1740
    scaffold_10119  GGAAGATGAA  TAGGGGCGTC  AGAGCTCTTA  CCCCGAGAGC  CCAAGGTAAC  TTTGCAAGTA  1740
    scaffold_23277  GGAAGATGAA  TAGGGGCGTC  AGGGCTCTTA  CCCCGAGAGC  CCAAGGTAAC  TTTACAAGTA  1740
    scaffold_12630  GGAAGATGAA  TAGGGGTGTC  AGGGCTCTTA  CCCCGAGAGC  CCAAGGTAAC  TTTACAAGTA  1740
     scaffold_2151  GGAAGATGAA  TAAGGACGTC  AGGGCTCTTA  CCCCGAGAGC  CCAAGGTAAC  TTTACAAGTA  1740
     scaffold_7237  GGAAGATGAA  TAAGGGCATC  AGGGCTCTTA  CCCCAAGAGC  CCAAAGTAAC  TTTACAAGTA  1740
    scaffold_12163  GGAAGATGAA  TAGGGTTGTC  AGGGCTCTGA  CCCCGGGAGC  CTCGGATAAC  CTTAAATGTA  1740
      scaffold_941  GGAAGATAAC  TAGGGGTGTC  AGGGCTCTAA  CCCCGAGAGC  CCCAGATAAC  CTTGCAAGTG  1740
    scaffold_22661  GGAAGATAAC  TAGGGGTGTC  AGGGCTCTAA  CCCCGAGAGC  CCCGGATAAC  CTTGCAAGTG  1740
     scaffold_7076  GGAAGATAAC  TAGGGGTGTC  AGGGCTCTAA  CCCCGAGAGC  CCCGGATAAC  CTTGCAAGTG  1740
    scaffold_22354  GGAAGATAAC  TAGGGGTGTC  AGGGCTCTAA  CCCCGAGAGC  CCCGGATAAC  CTTGCAAGTG  1740
    scaffold_14383  GGAAGATAAC  TAGGGGTGTC  AGGGCTCTAA  CCCCGAGAGC  CCCGGATAAC  CTTGCAAGTG  1740
                                              1,760                 1,780                1,800
                                                |                     |                    |
Ancestral_Gamma_G1  GGGGGCCAAC  CTGTGAACTT  TCTGGTAGAT  ACTGGAGCAA  CATTCTGTTT  TAAATCAACC  1800
 GeneScaffold_3363  GGGGGCAAAC  CTGTGAACTT  TTTATCTGAT  ACTGGTGCAA  CTTTCAGTTT  TAACAGAAAC  1800
     scaffold_1753  GGGGGCAAAC  CTGTGAACTT  TTTAGCTGAT  ACTGGTGCAA  CTTTCAGTTT  TAACAGAAAC  1800
    scaffold_13050  GGGGGCAAAC  CTGTGAACTT  TTTAGCTGAT  ACTGGTGCAA  CTTTCAGTTT  TAACAGAAAC  1800
    scaffold_24606  GGGGGCAAAC  CTGTGAACTT  TTTAGCTGAT  ACTGGTGCAA  CTTTCAGTTT  TAACAGAAAC  1800
    scaffold_10119  GGGGGCAAAC  CTGTGAACTT  TTTAGCTGAT  ACTGGTGCAA  CTTTCAGTTT  TAACAGAAAC  1800
    scaffold_23277  GGGGGCAAAC  CTGTGAACTT  TTTAGCTGAT  ACTGGTGCAA  CTTTCAGTTT  TAACAGAAAC  1800
    scaffold_12630  GGGGGCAAAC  CTGTGAACTT  TTTAGCTGAT  ACTGGTGCAA  CTTTCAGTTT  TAACAGAAAC  1800
     scaffold_2151  GGGGGCAAAC  CTGTGAACTT  TTTAGCTGAT  ACTGGTGCAA  CTTTCAGTTT  TAACAGAAAC  1800
     scaffold_7237  GGGGGCAAAC  CTGTGAACTT  TTTAGCTGAT  ACTGGTGCAA  CTTTCAGTTT  TAACAGAAAC  1800
    scaffold_12163  GGGGGCCAAC  CTGTGAATTT  TCTGGTAGAC  ACTGGAGCGA  CATTCTGTTT  TAAATCAACC  1800
      scaffold_941  GGGGGCCAAC  CTGTGGACTT  TCTGGTAGAT  ACTGGAGCAA  CATTCTGTTT  TAAATCAGCC  1800
    scaffold_22661  GGGGGCCAAC  CTGTGGACTT  TCTGGTAGAT  ACTGGAGCAA  CATTCTGTTT  TAAATCAGCC  1800
     scaffold_7076  GGGGGCCAAC  CTGCGGACTT  TCTGGTAGAT  ACTGGAGCAA  CATTCTGTTT  TAAATCAGCC  1800
    scaffold_22354  GGGGACCAAC  CTGTGGACTT  TCTGGTAGAT  ACTGGAGCAA  CATTCTGTTT  TAAATCAGCC  1800
    scaffold_14383  GGGGGCCAAC  CTGTGGACTT  TCTGGTAGAT  ACTGGAGCAA  CATTCTGTTT  TAAATCAGCC  1800
                                              1,820                 1,840                1,860
                                                |                     |                    |
Ancestral_Gamma_G1  TGTTGGCCCT  GTGAGTAAAG  AAAAAACCAC  CATTCTGGGG  GCAACTGGAG  GAAAAGCATA  1860
 GeneScaffold_3363  TGCTGGTCTG  TTATCCAAAG  AAAAGACTGT  AATTCAAGGA  GCCACTGGAG  GGAAGGCATA  1860
     scaffold_1753  TACTGGTCTG  TTATCCAAAG  AAAAGACTGT  AATTCAAGGA  GCCACTGGAG  GGAAGGCATA  1860
    scaffold_13050  TGCTGGTCTG  TTATCCAAAG  AAAAGACTGT  AATTCAAGGA  GCCACTGGAG  GGAAGGCATA  1860
    scaffold_24606  TGCTGGTCTG  TTATCCAAAG  AAAAGACTGT  AATTCAAGGA  GCCACTGGAG  GGAAGGCATA  1860
    scaffold_10119  TGCTGGTCTG  TTATCCAAAG  AAAAGACTGT  AATTCAAGGA  GCCACTGGAG  GGAAGGCATA  1860
    scaffold_23277  TGCTGGTCTG  TTATCCAAAG  AAAAGACTGT  AATTCAAGGA  GCCACTGGAA  GGAAGGCATA  1860
    scaffold_12630  TGCTGGTCTG  TTATCCAAAG  AAAAGATTGT  AATTCAAGGA  GCCACTGGAG  GGAAGGCATA  1860
     scaffold_2151  TGCTGGTCTG  TTATCCAAAG  AAAAGACTGT  AATTCAAGGA  GTCACTGGAG  GGAAGGCATA  1860
     scaffold_7237  TGCTGGTCTG  TTATCCAAAG  AAAAGACTGT  AATTCAAGGA  GCCACTGGAG  GGAAGGCATA  1860
    scaffold_12163  TGTGGGGCCT  GTGAGTAAAG  AAAAAACCAC  CATTCTGGGG  GCAACTGGAG  GAAAAGCATA  1860
      scaffold_941  TGTTGGCCCT  GTGAGTAAAG  AAAAAACCAC  TATTCTTGGG  GCAACTGGAG  GAAGAGCATA  1860
    scaffold_22661  TGTTGGCCCT  GTGAGTAAAG  AAAAAACCAC  TATTCTTGGG  GCAACTGGAG  GAAGAGCATA  1860
     scaffold_7076  TGTTGGCCCT  GTGAGTAAAG  AAAAAACCAC  TATTCTTGGG  GCAACTGGAG  GAAGAGCATA  1860
    scaffold_22354  TGTTGGCCCT  GTAAGTAAAG  AAAAAACCAC  TATTCTTGGG  GCAACTGGAG  GAAGAGCATA  1860
    scaffold_14383  TGTTGGCCCT  GTGAGTAAAG  AAAAAACCAC  TATTCTTGGG  GCAACTGGAG  GAAGAGCATA  1860
                                              1,880                 1,900                1,920
                                                |                     |                    |
Ancestral_Gamma_G1  CCCTTGGACT  CAGGCTCGTA  TAACTGACTT  GGGAAAAGGA  ACCATCACCC  ATTCCTTTTT  1920
 GeneScaffold_3363  CCCATGGACA  CAGGCAAAAA  TTACTGACTT  GGGAAAGGGA  ACCATCACAC  ATTCGTTTTT  1920
     scaffold_1753  CCCAAGGACA  CAGGCAAAAA  TTACTGACTT  GGGAAAGGGA  ACCATCACAC  ATTCGTTTTT  1920
    scaffold_13050  CCCATGGACA  CAGGCAAAAA  TTACTGACTT  GGGAAAGGGA  ACCATCACGA  ATTCGTTTTT  1920
    scaffold_24606  CCCATGGACA  CAGGCAAAAA  TTACTGACTT  GGGAAAGGGA  ACCATCACGC  TTTCGTTTTT  1920
    scaffold_10119  CCCATGGACA  CAGGCAAAAA  TTACTGACTT  GGGAAAGGGA  ACCATCACGC  ATTCGTTTTT  1920
    scaffold_23277  CCCATGGACA  CAGGCAAAAA  TTACTGACTT  GGGAAAGGGA  ACCATCACGC  ATTCGTTTTT  1920
    scaffold_12630  CCCATGGACA  CAGGCAAAAA  TTACTGACTT  GGGAAAGGGA  ACCATCACGC  ATTCGTTTTT  1920
     scaffold_2151  CCCATGGACA  CAGGCAAAAA  TTACTGACTT  GGGAAAGGGA  ACCATCACAC  ATTCGTTTTT  1920
     scaffold_7237  CCCATGGACA  CAGGCAAAAA  TTACTGACTT  GGGAAAGGGA  ACCATCACGC  ATTCGTTTTT  1920
    scaffold_12163  TCCTTGGACT  CAGGCTCGTA  TAACTGATTT  AGGAAAAGGA  ACCATCACCC  ATTCCTTTTT  1920
      scaffold_941  CCCTTGGACT  CGGGCTCGTA  TAACTGACCT  GGGAAAAGGG  ACCATCACCC  ATTCCTTTTT  1920
    scaffold_22661  TCCTTGGACT  CGGGCTCGTA  TAACTGACCT  GGGAAAAGGG  ACCATCACCC  ATTCCTTTTT  1920
     scaffold_7076  CTCTTGGACT  CGGGCTCGTA  TAACTGACCT  GGGAAAAGGG  ACCATCACCC  ATTCCTTTTT  1920
    scaffold_22354  CCCTTGGACC  CGGGCTCGTA  TAACTGACCT  GAGAAAAGGG  ACCATCACCC  ATTCCTTTTT  1920
    scaffold_14383  CCCTTGGACT  CGGGCTCGTA  TAACTGACCT  GGGAAAAGGG  ACCATCACCC  ATTCCTTTTT  1920
```

```
                                          1,940                         1,960                    1,980
                                            |                             |                        |
Ancestral_Gamma_G1  GGTTATGCCT  GAATGCCCCT  ATCCTTTGTT  GGGAAGAGAT  TTGCTACAGA  AATTACAAGC  1980
 GeneScaffold_3363  AGTTATGCCT  GAATGTCCTT  TTCCTTTGCT  TGGAAGAGAT  TTGCTCCAGA  AATTAAAAGC  1980
     scaffold_1753  AGTTATGCCT  GAATGTCCCT  TTCCTTTGCT  CGGAAGAGAT  TTGCTCCAGA  AATTAAAAGC  1980
    scaffold_13050  AGTTATGCCT  GAATGTCCCT  TTCCTTTGCT  TGGAAGAGAT  TTGCTCCAGA  AATTAAAAGC  1980
    scaffold_24606  AGTTATGCCT  GAATGTCCTT  TTCCTTTGCT  TGGAAAAGAT  TTGCTCCAGA  AATTAAAAGC  1980
    scaffold_10119  AGTTATGCCT  GAATGTCCTT  TTCCTTTGCT  TGGAAGAGAT  TTGCTCCAGA  AATTAAAAGC  1980
    scaffold_23277  AGTTATGCCT  GAATGTCCTT  TTCCTTTGCT  TGGAAGAGAT  TTGCTCCAGA  AATTAAAAGC  1980
    scaffold_12630  AGTTATGCCT  GAATGTCCTT  TTCCTTTGCT  TGGAAGAGAT  TTGCTCCAGA  AATTAAAAGC  1980
     scaffold_2151  AGTTATGCCT  GAATGTCCTT  TTCCTATGCT  TGGAAGAGAT  TTGCTCCAGA  AATTAAAAGC  1980
     scaffold_7237  AGTTATGCCT  GAATGTCCTT  TTCCTTTGCT  TGGAAGAGAT  TTGCTCCAGA  AATTAAAAGC  1980
    scaffold_12163  GGTTATGCCT  GATTGCCCCT  ATCCTCTGTT  GGGAAGAGAC  TTGTTACAGA  AATTACAAGC  1980
      scaffold_941  GGTTATGCCT  GAATGCCCCT  ATCCTTTGTT  AGGAAGAGAT  TTGCTACAAA  AATTACAGGC  1980
    scaffold_22661  GGTTTTGCCT  GAATGCCCCT  ATCCTTTGTT  AGGAAGAGAT  TTGCTACAAA  AATTACAGGC  1980
     scaffold_7076  GGTTATGCCT  GAATGCCCCT  ATCCTTTGTT  AGGAAGAGAT  TTGCTACAAA  AATTACAGGC  1980
    scaffold_22354  GGTTTTGCCT  GAATGCCCCT  ATCCTTTGTT  AGGAAGAGAT  TTGCTACAAA  AATTACAGGC  1980
    scaffold_14383  GGTTTTGCCT  GAATGCCCCT  ATCCTTTGTT  AGGAAGAGAT  TTGCTACAAA  AATTACAGGC  1980
                                          2,000                         2,020                    2,040
                                            |                             |                        |
Ancestral_Gamma_G1  CACTATTAAG  AGACCAGTTG  CATACCTATC  TAAAAGACTA  GATTATGTGG  CTGCAGGGTG  2040
 GeneScaffold_3363  CACTATTAAG  CAACGAGTGG  CATATCTCTC  AAAGAGACTA  GATCTGGTGG  CTGGAGGAAG  2040
     scaffold_1753  CACTATTAAG  CAACCAGTGG  CATATCTCTC  AAAGAGACTA  GATCTGGTGG  CTGGAGGAAG  2040
    scaffold_13050  CACTATTAAG  CAACGAGTGA  CATATCTCTC  AAAGAGACTA  GATCCGGTGG  CTGGAGGAAG  2040
    scaffold_24606  CACTATTAAG  CAACGAGTGG  CATATCTCTC  AAAGAGACTA  GATCCGGTGG  CTGGAGGAAG  2040
    scaffold_10119  CACTATTAAG  CAACGAGTGG  CATATCTCTC  AAAGAGACTA  GATCCGGTGG  CTGGAGGAAG  2040
    scaffold_23277  CACTATTAAG  CAACGAGTGG  CATATCTCTC  AAAGAGACTA  GATCCGGTGG  GTGGAGGAAG  2040
    scaffold_12630  CACTATTAAG  CAACGAGTGG  CATATCTCTC  AAAGAGACTA  GATCCGGTGG  CTGGAGGAAG  2040
     scaffold_2151  CACTATTAAG  CAACGAGTGG  CATATCTCTC  AAAGAGACTA  GATCCGGTGG  CTGGAGGAAG  2040
     scaffold_7237  CACTATTAAG  CAACGAGTGG  CATATCTCTC  AAAGAGACTA  GATCCGGTGG  CTGGAGGAAG  2040
    scaffold_12163  CACAATTAAG  AGACCGGTTG  CCTACCTATC  TAAAAGACTA  GATTATGTAG  CAGCAGGGTG  2040
      scaffold_941  CACTATTAAA  AGACCAGTTG  CATACTTATC  TAAAAGGTTA  GATTGCGTGG  CTGCAGGGTG  2040
    scaffold_22661  CACTATTAAA  AGACCAGTTG  CATACTTATC  TAAAAGGTTA  GATTGCGTGG  CTGCAGGGTG  2040
     scaffold_7076  CACTATTAAA  AGACCAGTTG  CATACTTATC  TAAAAAGTTA  GATTGCGTGG  CTGCAGGGTG  2040
    scaffold_22354  CACTATTAAA  AGACCAGTTG  CATATTTATC  TAAAAGGTTA  GATTGCGTGG  CTGCAGGGTG  2040
    scaffold_14383  CACTATTAAA  AGACCAGTTG  CATACTTATC  TAAAAGGTTA  GATTGCGTGG  CTGCAGGGTG  2040
                                          2,060                         2,080                    2,100
                                            |                             |                        |
Ancestral_Gamma_G1  GCCTAGTTGT  CTGAGAGCTG  TTGCAGCTAC  GGCGGTTCTA  GTGAAAGAAG  CCCACAATTG  2100
 GeneScaffold_3363  GCCTAGTTGC  CTGAGAGCAC  TAGCTGCTAC  TGCTATATTG  ACCAAAGACG  CTTTAAATTG  2100
     scaffold_1753  GCCTAGTTGC  CTGAGAGCAC  TAGCTGCTAC  TGCTATATTG  ACCAAAGACG  CTTTAAATTG  2100
    scaffold_13050  GCCTAGTTGC  CTGAGAGCAC  TAGCTGCTAC  TGCTATATTG  ACCAAAGACG  CTTTAAATTG  2100
    scaffold_24606  GCCTAGTTGC  CTGAGAGCAC  TAGCTGCTAC  TGCTATATTG  ACCAAAGACG  CTTTAAATTG  2100
    scaffold_10119  GCCTAGTTGC  CTGAGAGCAC  TAGCTGCTAC  TGCTATATTG  ACCAAAGACG  CTTTAAATTG  2100
    scaffold_23277  GCCTAGTTGC  CTGAGAGCAC  TAGCTGCTAC  TGCTATATTG  ACCAAAGACG  CTTTAAATTG  2100
    scaffold_12630  GCCTAGTTGC  CTGAGAGCAC  TAGCTGCTAC  TGCTATATTG  ATCAAAGACG  CTTTAAATTG  2100
     scaffold_2151  GCCTAGTTGC  CTGAGAGCAC  TAGCTGCTAC  TGCTATATTG  ACCAAAGACG  CTTTAAATTG  2100
     scaffold_7237  GCCTAGTTGC  CTGAGAGCAC  TAGCTGCTAC  TGCTCTATTG  ACCAAAGACG  CTTTAAATTG  2100
    scaffold_12163  GCCCAGTTGT  CTGCGAGCTG  TTGCAGCTAC  GGCGGTTCTA  GTGAAAGAAG  CCCATAATTG  2100
      scaffold_941  GCCTAATTGT  CTGAGGGCCG  TTGCAGCCAC  GGCAGTTCTA  GTGAAAGAAG  CCCACAATTG  2100
    scaffold_22661  GTCTAATTGT  CTGAGGGCCG  TTGCAGCCAC  GGCAGTTCTA  GTGAAAGAAG  CCCACAATTG  2100
     scaffold_7076  GCCTAATTGT  CTGAGGGCCT  TTGCAGCCAC  GGCAGTTCTA  GTGAAAGAAG  CCCACAATTG  2100
    scaffold_22354  GCCTAATTGT  CTGAGGGCCG  TTGCAGCCAC  GGCAGTTCTA  GTGAAAGAAG  CCCACAATTG  2100
    scaffold_14383  GTCTAATTGT  CTGAGGGCCG  TTGCAGCCAC  GGCAGTTCTA  GTGAAAGAAG  CCCACAATTG  2100
                                          2,120                         2,140                    2,160
                                            |                             |                        |
Ancestral_Gamma_G1  ACGCTTGGGC  AGCCTGTGAC  TGTTATTGGA  CCTCACTTTG  TGGAGCGACT  CCTGAAGGGA  2160
 GeneScaffold_3363  ACCTTGGGCC  AAAATATTGA  AATTGTGGCT  CCACATGCAG  TTGAAGCCAT  GCTGCGCAGC  2160
     scaffold_1753  ACCTTGGGCC  AAAATATTGA  AATTGTGGCT  CCACATGCAG  TTGAAGCCAT  GCTGCGCAGC  2160
    scaffold_13050  ACCTTGGGCC  AAAATATTGA  AATTGTGGTT  CCACATGCAG  TTGAAGCCAT  GCTGCGCAGC  2160
    scaffold_24606  ACCTTGGGCC  CAAATATTGA  AATTGTGGCT  CCACATGCAG  TTGAAGCCAT  GCTGCGCAGC  2160
    scaffold_10119  ACCTTGGGCC  AAAATATTGA  AATTGTGGCT  CCACATGCAG  TTGAAGCCAT  GCTGCGCAGC  2160
    scaffold_23277  ACCTTGGGCC  AAAATATTGA  AATTGTGGCT  CCACATGCAG  TTGAAGCCAT  GCTGCGCAGC  2160
    scaffold_12630  ACCTTGGGCC  AAAATATTGA  AATTGTGGCT  CCACATGCAG  TTGAACCCAT  GCTGCGCAGC  2160
     scaffold_2151  ACCTTGGGCC  AAAATATTGA  AATTGTGGTT  CCACATGCAG  TTGAAGCCAT  GCTGCGCAGC  2160
     scaffold_7237  ACCTTGGGCC  AAAATATTGA  AATTGTGGCT  CCACATGCAG  TTGAAGCCAT  GCTGCGCAGC  2160
    scaffold_12163  ACACTTGGGC  AGCCTGTGAC  TGTTATTGGA  CCTCACTTTG  TGGAGCGACT  CCTGAAGGGA  2160
      scaffold_941  ACGCTTGGGC  AGCCTGTGAC  TGTTATCGGA  CCTCACTTTG  TGGAGCGGCT  CCTGAAGGGA  2160
    scaffold_22661  ACGCTTGGGC  AGCCTGTGAC  TGTTATCGGA  CCTCACTTTG  TGGAGCGGCT  CATGAGGGGA  2160
     scaffold_7076  ACGCTTGGGC  AGCCTGTGAC  TGTTATCGGA  CCTCACTTTG  TGGAGCGGCT  CCTGAAGGGA  2160
    scaffold_22354  ACGCTTGGGC  AGCCTGTGAC  TGTTATCGGA  CCTCACTTTG  TGGAGCGGCT  CCTGAAGGGA  2160
    scaffold_14383  ACGCTTGGGC  AGCCTGTGAC  TGTTATCGGA  CCTCACTTTG  TGGAGCGGCT  CCTGAAGGGA  2160
```

```
                                           2,180                      2,200                    2,220
                                               |                          |                        |
Ancestral_Gamma_G1  CCACCCGAGC GATGGATATC GAACGCTCGG ATCACCCAGT ACCAGGTACT CTTATTAAAC 2220
GeneScaffold_3363   CCTCCTGAAA GATGGATTTC CAACGCCCGG ATTACTCAGT ACCAGGTACT ATTGCTTAAT 2220
scaffold_1753       CCTCCTGAAA GATAGATTTC CAACGCCCGG ATTACTCAGT ACCAGGTACT GTTGCTTAAC 2220
scaffold_13050      CCTCCTGAAA GATGGATTTC CAACGCCCGG ATTACTCAGT ACCAGGTACT GTTGCTTAAT 2220
scaffold_24606      CCTCCTGAAA GATGGATTTC CAATGCCCGG ATTACTCAGT ACCAGGTACT GTTGCTTAAC 2220
scaffold_10119      CCTCCTGAAA GATGGATTTC CAACGCCCGG ATTACTCAGT ACCAGGTACT GTTGCTTAAT 2220
scaffold_23277      CCTCCTGAAA GATGGATTTC CAACGCCCGG ATTACTCAGT ACCAGGTACT GTTGCTTAAT 2220
scaffold_12630      CCTCCTGAAA GATGGATTTC CAATGCCCGG ATTACTCAGT ACCAGGTACT GTTGCTTAAT 2220
scaffold_2151       CCTCCTGAAA GATGGATTTC CAACGCCCGG ATTACTCAGT ACCAGGTACT GTTGCTTAAT 2220
scaffold_7237       CCTCCTGAAA GATAGATTTC CAACGCCCGG ATTACTCAGT ACCAGGTACT GTTGCTTATT 2220
scaffold_12163      CCACCCGAGC GATGGATATC GAACACTCGG ATCACCCAGT ACCAGGTACT CTTATTAAAC 2220
scaffold_941        CCACCCGAGC GATGGATATC AAACGCTCGA ATCACCCAAT ACCAGGTACT CTTATTAAAC 2220
scaffold_22661      CCACTCGAGC GATGGATATC GAACGCTTGA ATCACCCTAT ACCAGGTACT CTTATTAAAC 2220
scaffold_7076       CCACCCGAGC GATGGATATC GAACGCTCGA ATCAGCCAAT ACCAGGTACT CTTATTAAAC 2220
scaffold_22354      CCACCCGAGC AATGGATATC GAACGCTTGA ATCACCCAAT ACCAGGTACT CTTATTAAAC 2220
scaffold_14383      CCACTCGAGC GATGGATATC GAACGCTCGA ATCACCCTAT ACCAGGTACT CTTATTAAAC 2220

                                           2,240                      2,260                    2,280
                                               |                          |                        |
Ancestral_Gamma_G1  CCTCACAGGT TCAATTTGCA AAACCAACAG TGTTGAATCC GCGACTTTGC TCCCGGATGA 2280
GeneScaffold_3363   CCCCACAGAC CCAGTTTTTG AAAACTGCAG CCCTGAACCC GCCACGCTGC TCCCGGATGA 2280
scaffold_1753       CCCCACAGAC CCAGTTTTTG AAAACTGCAG CCCTGAACCC GCCACACTGC TCCCGGATGA 2280
scaffold_13050      CCCCACAGAC CCAGTTTTTG AAAACTGCAG CCCTGAACCC GCCACGCTGC TCCCGGATAA 2280
scaffold_24606      CCCCACAGAC CCAGTTTTTG AAAACTGCAG CCCTGAACCC GCCACGCTGC TCCCGGATGA 2280
scaffold_10119      CCCCACAGAC CCAGTTTTTG AAAACTGCAG CCCTGAACCC GCCACGCTGC TCCCGGATGA 2280
scaffold_23277      CCCCACAGAC CCAGTTTTTG AAAACTGCAG CCCTGAACCC GCCACACTGC TCCCGGATGA 2280
scaffold_12630      CCCCACAGAC CCAGTTTTTG AAAACTGCAG CCCTGAACCC GCCACGCTGC TCCCGGATGA 2280
scaffold_2151       CCCCACAGAC CCAGTTTTTG AAAACTGCAG CCCTGAACCC GCCACGCTGC TCCCGGATGA 2280
scaffold_7237       CCCCACAGAC CCAGTTTTTG AAAACTGCAG CCCTGAACCC GCCACGCTGC TCCCGGATGA 2280
scaffold_12163      CCTCACAGGT TCAATTTGCA AAACCAACGG TGTTAAATCC GCGACTTTGC TCCCGGATGA 2280
scaffold_941        CCTCACAGGT TCGATTTGCT AAACCAACAA TATTGAATCC GCAACCTTGC TCCCAGATGA 2280
scaffold_22661      CCTCACAGGT TCAATTTGCT AAACCGACAA TATTGAATCC GCAACCTTGC TCCCAGATGA 2280
scaffold_7076       CCTCACAGGT TCAATTTGCT AAACCGACAA TATTGAATCC GCAACCTTGC TCCCAGATGA 2280
scaffold_22354      CCTCACAGGT TCAATTTGCT AAATCGACAA TATTGAATCC GCAACCTTGC TCCCAGATGA 2280
scaffold_14383      CCTCACAGGT TCAATTTGCT AAACCGACAA TATTGAATCT GCAACCTTGC TCCCAGATGA 2280

                                           2,300                      2,320                    2,340
                                               |                          |                        |
Ancestral_Gamma_G1  TGAAGGAACC TGTACATGAT AACTGGGGTA AGACCTGACT TGCGGGATGA GCCCTGGCCT 2340
GeneScaffold_3363   TGAAGGCTCC ATTGCATCAT AACTGGACTA CGGCCAGACT TGAAGGATGA GCCGTGGCCT 2340
scaffold_1753       TGAAGGCTCC ATTGCATCAT AACTGGACTA CGGCCAGACT TGAAGGATGA GCCGTGGCCT 2340
scaffold_13050      TGAAGGCTCC ATTGCATCAT AACTGGACTA CGGCCAGACT TGAAGGATGA ACCGTAGCCT 2340
scaffold_24606      TGAAGGCTCC ATTGCATCAT AACTGGAGTA CGGCCAGACT TGAAGGAAGA GCCGTGGCCT 2340
scaffold_10119      TGAAGGCTCC ATTGCATCAT AACTGGACTA CGGCCAGACT TGAAGGATGA GCCGTGGCCT 2340
scaffold_23277      TGAAGGCTCC ATTGCATCAT AACTGGACTA CGGCCAGACT TGAAGGATGA GCCGTGGCCT 2340
scaffold_12630      TGAAGGCTCC ATTGCATCAT AACTGGAATA CGGCCAGACT TGAAGGATGA GCCGTGGCCT 2340
scaffold_2151       TGAAGGCTCC ATTGCATCAT AACTGGAATA CGGCCAGACT TGAAGGATGA GCCGTGGCCT 2340
scaffold_7237       TGAAGGCTCC ATTGCATCAT AACTGGACTA CGGCCAGACT TGAAGGATGA GCCATGGCCT 2340
scaffold_12163      TGAAGGAACC TGTACATGAT AACTGGGGTA AGACCTGATT TGCGGGATGA GCCCTGGCCT 2340
scaffold_941        TGAAGGAACC TGTACATGAT AACTGGGGTA AGACCTGACT TACGAGATGA GCCTTGGCTC 2340
scaffold_22661      TGAAGGAACC TGTACATGAT AACTGGGGTA AGACCTGACT TACGAGATGA GCCTTGGCTC 2340
scaffold_7076       TGAAGGAACC TGTACATGAT AACTGGGGTA AGACCTGACT TACGAGATGA GCCTTGGCTC 2340
scaffold_22354      TGAAGGAACC TGTACATGAT CACTGGGGTA AGACCTGACT TACGAGATGA GCCTTGGCTC 2340
scaffold_14383      TGAAGGAACC TGTACATGAT AACTGGGGTA AGACCTGACT TACGAGATGA GCCTTGGCTC 2340

                                           2,360                      2,380                    2,400
                                               |                          |                        |
Ancestral_Gamma_G1  AACCCAGATG CCACCCTATA TACGGACGGA AGCAGTTTTG TCACTGAAGG CGTCAGGTAT 2400
GeneScaffold_3363   AATCCGGATG CGGTATTATT TACCAATGGA AGCAGTTTTG TATCCGACGG AGTAAGGTAT 2400
scaffold_1753       AATCCGGATG TGGTATTATT TACCAATGGA AACAGTTTTG TATCCGACGG AGTAAGGTAT 2400
scaffold_13050      AATCCGGATG CGGTATTATT TACCAAAGGA AGCAGTTTTG TATCCGACGG AGTAAGGTAT 2400
scaffold_24606      AATCCGGATG GGGTATTATT TACCAATGGA AGCAGTTTTG TATCCGACGG AGTAAGGTAT 2400
scaffold_10119      AATCCGGATG CGGTATTATT TACCAATGGA AGCAGTTTTG TATCCGACGG AGTAAGGTAT 2400
scaffold_23277      AATCCGGATG CCGTATTATT TACCAATGGA AGCAGTTTTG TATCCGATGG AGTAAGGTAT 2400
scaffold_12630      AATCCGGATG TGGTATTATT TACCAATGGA AGCAGTTTTG TATCCGACGG AGTAAGGTAT 2400
scaffold_2151       AATCCGGATG CGGTATTATT TACCAATGGA AGCAGTTTTG TATCCGACGG AGTAAGGTAT 2400
scaffold_7237       AATCCGGATG CGGTATTATT TACCAATGGA AGCAGTTTTG TATCTGACGG AGTAAGGTAT 2400
scaffold_12163      AACCCCGATG TCACTCTATA TACGGACGGA AGCAGTTTTG TCACTGAAGG CATCAGGTAT 2400
scaffold_941        AACCCAGACG CCACCCTGAA TACAGACGGA AGCAGTTATG TCACAGAAGG CGTCAGGTAT 2400
scaffold_22661      AACCCAGACG CCACCCTGTA CACGGACGGA AGCAGTTATG TCACAGAAGG CGTCAGGTAT 2400
scaffold_7076       AACCCAGACG CCACCCTGTA CACGGACGGA AGCAGTTATG TCACAGAAGG CGTCAGGTAT 2400
scaffold_22354      AACCCAGACG CCACCCTGTA CACGGACGGA AGCAGTTATG TCACAGTAGG CGTCAGGTAT 2400
scaffold_14383      AACCCAGACG CCACCCTGTA CACGGACGGA AGCAGTTATG TCACAGAAGG CGTCAGGTAT 2400
```

```
                                            2,420                         2,440                       2,460
                                              |                             |                           |
Ancestral_Gamma_G1  GCGGGAGCAG  CGGTAGTTGC  CGATGACACT  GTGGTTTGGG  CTCAGGCTCT  TGGACATGGG  2460
 GeneScaffold_3363  GCCGGGGCAG  CCGTAGTAAC  AGTCAGGGAT  GTGATATGGG  CACAGGCCCT  AAGCCATGGG  2460
     scaffold_1753  GCCGGGGCAG  CCGTAGTAAC  AGTCAAGGAT  GTGATATGGG  CACAGGCCCT  AAGCCATGGG  2460
    scaffold_13050  GCCGGGGCAG  CCGTAGTAAC  AGTCAGGGAT  GTGATATGGG  CACAGGCCCT  AAGCCATGGG  2460
    scaffold_24606  GCCGGGGCAG  CCATAGTAAC  GATCAGGGAT  GTGATATGGG  CACAGGCCCT  AAGCCATGGG  2460
    scaffold_10119  GCCGGGGCAG  CCATAGTAAC  GGTCAGGGAT  GTGATATGGG  CACAGGCCCT  AAGCCATGGG  2460
    scaffold_23277  GCCGGGGCAG  CTGTAGTAAC  AGTCAGGAAT  GTGATATGGG  CACAGGCCCT  AAGCCATGGG  2460
    scaffold_12630  GCCGGGACAG  CCATAGTAAC  GGTCAGGGAT  GTGATATGGA  CACAGGCCCT  AAGCCATGGG  2460
     scaffold_2151  GCCGGGGCAG  CCATAGTAAC  GGTCAGGGAT  GTGATATGGG  CACAGGCCCT  AAGCCATGGG  2460
     scaffold_7237  GCCGGGGCAG  CCATAGTAAC  GGTCAGGGAT  GTGATATGGG  CACAGGCCCT  AAGCCATGGG  2460
    scaffold_12163  GCGGGAGCAG  CAGTAGTTGC  TGATGATACT  GTTGTTTGGG  CTCAGGCTCT  TGGACATGGG  2460
      scaffold_941  GCGGGAGCAG  CGGTAGTTGC  CGATGACACT  GTGGTTTGGG  CTCAGGCTCT  CGGACAAGGG  2460
    scaffold_22661  GTGGGAGCAG  CGGTAGTTGC  CAATGACACT  GTGGTTTGGG  CTCAGGCTCT  CGGACAAGGG  2460
     scaffold_7076  GTGGGAGCAG  CGGTAGTTGC  CGATGACACT  GTGGTTTGGG  CTCAGGCTCT  CGGACAAGGG  2460
    scaffold_22354  GCGGGAGCAG  CGGTAGTTGC  CGATGACACT  GTGGTTTGGG  CTCAGGCTCT  CGGACAAGGG  2460
    scaffold_14383  GTGGGAGCAG  CGGTAGTTGC  CGATGACACT  GTGGTTTGGG  CTCAGGCTCT  CGGACAAGGG  2460
                                            2,480                         2,500                       2,520
                                              |                             |                           |
Ancestral_Gamma_G1  ACATCAGCCC  AGAAGGCTGA  ACTCATTGCT  CTAACTGAAG  CTCTTAAATG  GGCTCAAGGG  2520
 GeneScaffold_3363  ACATCGGCAC  AGAGGGCTGA  ACTTACAGCT  CTAACACAAG  CTTTACGGTG  GGGAAAGGAT  2520
     scaffold_1753  ACATCGGCAC  GGAGGGCTGA  ACTTATAGCT  CTAACACAAG  CTTTACGGTG  GGGAAAGGAT  2520
    scaffold_13050  ACATCGGCAC  AGAGGGCTGA  ACTTATAGCT  CTAACACAAG  CTTTACGGTG  GGGAAAGGAT  2520
    scaffold_24606  ACATTGGCAC  AGAGGGCTAA  ACTTATAGCT  CTAACACAAG  CTTTACGGTG  GGGAAAGGAT  2520
    scaffold_10119  ACATTGGCAC  AGAGGGCTAA  ACTTATAGCT  CTAACACAAG  CTTTACGGTG  GGGAAAGGAT  2520
    scaffold_23277  ACATCGGCAC  AGAGGGCTGA  ACTTATAGCT  CTAACACAAG  CTTTACGGTG  GGGAAAGGAT  2520
    scaffold_12630  ACATTGGCAC  AGAGGGCTAA  ACTTATAGCT  CTAACACAAG  CTTTACGGTG  GGGAAAGGAT  2520
     scaffold_2151  ACATTGGCAC  AGAGGGCTAA  ACTTATAGCT  CTAACACAAG  CTTTACGGTG  GGGAAAGGAT  2520
     scaffold_7237  ACATTGGCAC  AGAGGGCTAA  ACTTATAGCT  CTAACACAAG  CTTTACGGTG  GGGAAAGGAT  2520
    scaffold_12163  GCATCAGCCC  AAAAGGCTGA  ACTCATTGCT  CTGACTGAAG  CTCTTAAATG  GGCTCAAGGA  2520
      scaffold_941  ACTTCAGCCC  AGAAGGCCGA  GCTCATTGCA  TTAACTGAAA  CTCTTAAATG  GGCTCGAGGG  2520
    scaffold_22661  ACTTCAGCCC  AGAAGGCCGA  GCTCATTGCA  TTAACTGAAG  CTCTTAAATG  GGCTCGAGGG  2520
     scaffold_7076  ACTTCAGCCC  AGAAGGCCGA  GCTCATGGCA  TTAACTGAAG  CTCTTAAATG  GGCTCGAGGG  2520
    scaffold_22354  ACTTCAGCCC  AGAAGGCCGA  GCTCATGGCA  TTAACTGAAG  CTCTTAAATG  GGCTCGAGAG  2520
    scaffold_14383  ACTTCAGCCC  AGAAGGCCGA  GCTCATTGCA  TTAACTGAAG  CTCTTAAATG  GGCTCGAGGG  2520
                                            2,540                         2,560                       2,580
                                              |                             |                           |
Ancestral_Gamma_G1  AAAACTGTTA  ACATCTACAC  TGATAGCCGA  TATGCCTTTG  CAACGGCACA  TGTACATGGA  2580
 GeneScaffold_3363  AAAACTGTAA  ACATCTATAC  TGACAGTAGA  TATGCCTTTG  CTACTGCCCA  CATACATGGA  2580
     scaffold_1753  AAAACTGTAA  ACATCTATAC  TGACAGTAGA  TATGCCTTTG  CAATGGCACA  TGTACATGGA  2580
    scaffold_13050  AAAACTGTAA  ACATCTATAC  TGACAGTAGA  TATGCCTTTG  TAACGGCACA  TGTACATGGA  2580
    scaffold_24606  AAAACTGTAA  ACATCTATAC  TGATAGCCGA  TATGCTTTTG  CAACGGCACA  TGTACATGGA  2580
    scaffold_10119  AAAACTGTAA  ACATCTATAC  TGATAGTAGA  TATGCCTTTG  CAACGGCACA  TGTACATGGA  2580
    scaffold_23277  AAAACTGTAA  ACATCTATAC  TGACAGTAGA  TATGCCTTTG  CAACGGCACA  TGTACATGGA  2580
    scaffold_12630  AAAACTGTAA  ACATCTATAC  TGATAGTAGA  TGTGCCTTTG  CAACGGCACA  TGTACATGGA  2580
     scaffold_2151  AAAACTGTAA  ACATCTACAC  TGATAGCCGA  TATGCCTTTG  CAACGGCACA  TGTACATGGA  2580
     scaffold_7237  AAAACTGTAA  ACATCTATAC  TGATAGTAGA  TATGCCTTTG  CAACGGCACA  TGTACATGGA  2580
    scaffold_12163  AAGACTGTTA  ACATCTACAC  TGATAGCCGA  TATGCTTTTG  CAACGGCACA  TGTACATGGA  2580
      scaffold_941  AAAACTGTTA  ACATCTACAC  TGATAGCCGA  TATGCCTTTG  CAACGGCACA  TGTACATGGA  2580
    scaffold_22661  AAAACTGTTA  ACATCTACAC  TGATAGCCGA  TATGCCTTTG  CAACGGCACA  TGTACATGGA  2580
     scaffold_7076  AAAACTGTTA  ACATCTACAC  TGATAGCCGA  TATGCCTTTG  CAACGGCACA  TGTACATGGA  2580
    scaffold_22354  AAAACTGTTA  ACATCTACAC  TGATAGCCGA  TATGCCTTTG  CAACGGCACA  TGTACATGGA  2580
    scaffold_14383  AAAACTGTTA  ACATCTACAC  TGATAGCCGA  TATGCCTTTG  CAACGGCACA  TGTACATGGA  2580
                                            2,600                         2,620                       2,640
                                              |                             |                           |
Ancestral_Gamma_G1  GCCCTGTACA  AGGAAAGAGG  ACTATTAACA  TCAGGGGGAA  AGGAAATTAA  GAACAAGGAG  2640
 GeneScaffold_3363  GCTTTATATA  AGGAAAGAGG  ACTTTTAACA  TCAGGAGGCA  AAAAAATTAA  AAATAGGGAA  2640
     scaffold_1753  GCCCTGTACA  AGGAAAGAGG  ACTATTGACA  TCAGGGGGAA  AGAAAATTAA  GAACAAGGAG  2640
    scaffold_13050  GCCCTGTACA  AGGAAAGAGG  ACTATTAACA  TCAGGGGGAA  AGGAAATTAA  AAACAAAGAG  2640
    scaffold_24606  GCCCTGTACA  AGGAAAGAGG  ACTATTAACA  TCAGGGGGAA  AGGAAATTAA  AAACAAAGAG  2640
    scaffold_10119  GCCCTATACA  AGGAAAGAGG  ACTATTGACA  TCAGGGGGAA  AGGAAATTAA  GAACAAGGAG  2640
    scaffold_23277  GCCCTGTACA  AGGAAAGAGG  ACTATTGACA  TCAGGGGGAA  AGAAAATTAA  GAACAAGGAG  2640
    scaffold_12630  GCCCTGTACA  AGGAAAGAGG  ACTATTGACA  TCAGGGGGAA  AGGAAATTAA  GAACAAGGAG  2640
     scaffold_2151  GCCCTGTACA  AGGAAAGAGG  ACTATTGACA  TCAGGGGGAA  AGAAAATTAA  GAACAAGGAG  2640
     scaffold_7237  GCCCTGTACA  AGGAAAGAGG  ACTATTGACA  TCAGGGGGAA  AGGAAACTAA  GAACAAGGAG  2640
    scaffold_12163  GCCCTGTACA  AGGAAAGAGG  ACTATTAACA  TCAGGGGGAA  AGGAAATTAA  AAACAAAGAG  2640
      scaffold_941  GCCCTGGACA  AGGAAAGAGG  ACTATTAACA  TCAGGGGGAA  AGGAAATTAA  GAACAAGGAG  2640
    scaffold_22661  GCCCTGTACA  AGGAAAGAGG  ACTATTAACA  TCAGAGGGAA  AGGAAATTAA  GAACAAGGAG  2640
     scaffold_7076  GCCCTGGACA  AGGAAAGAGG  ACTATTAACA  TCAGGGGGAA  AGGAAATTAA  GAACAAGGAG  2640
    scaffold_22354  GCCCTGTACA  AGGAAAGAGG  ACTATTAACA  TCAGGGGGAA  AGGAAATTAA  GAACAAGGAG  2640
    scaffold_14383  GCCCTGTACA  AGGAAAGAGG  ACTATTAACA  TCAGAGGGAA  AGGAAATTAA  GAACAAGGAG  2640
```

```
                                            2,660                            2,680                       2,700
                                              |                                |                           |
Ancestral_Gamma_G1  CAGATTTTGG  CTCTTTTGGA  GGCTATCTGG  CTACCTAAGA  AAGTGGCTAT  TATCCATTGC  2700
  GeneScaffold_3363  GAGATCTTAG  CCCTGTTAGA  AGCCATTTGG  CTACCAAAAA  GAGTAGCCAT  AATTCATTGT  2700
      scaffold_1753  CAGATTTTTG  CTCTTTTGGA  GGCTATCTGG  CTACCTAAGA  AAGTGGCTAT  TATCCATTGC  2700
     scaffold_13050  CAGATTTTGG  CTCTTTTGGA  GGCAATCTGG  CTACCTAAGA  AAGTGGCTAT  TATCCATTGC  2700
     scaffold_24606  CAGATTTTGG  CTCTTTTGGA  GGCAATCTGG  CTACCTAAGA  AAGTGGCTAT  TATCCATTGC  2700
     scaffold_10119  CAGATTTTGG  CTCTTTTGGA  GGCTATCTGG  CTACCTAAGA  AAGTGGCTAT  TATCCATTGC  2700
     scaffold_23277  CAGATTTTGG  CTCTTTTGGA  GGCTATCTGG  CTACCTAAGA  AAGTGGCTAT  CATCCATTGC  2700
     scaffold_12630  CAGATTTTGG  CTCTTTTGGA  GGCTATCTGG  CTACCTAAGA  AAGTGGCTAT  TATCCATTGC  2700
      scaffold_2151  CAGATTTTGG  CTCTTTTGGA  GGCTATCTGG  CTACCTAAGA  AAGTGGCTAT  TATCCATTGC  2700
      scaffold_7237  CAGATTTTGG  TTCTTTTGGA  GGCTATCTGG  CTACCTAAGA  AAGTGGCTAT  TATCCATTGC  2700
     scaffold_12163  CAGATTTTGG  CTCTTTTGGA  GGCAATCTGG  CTACCTAAGA  AAGTGGCTAT  TATCCATTGC  2700
       scaffold_941  CAGATTTTGG  CTCTTTTGGA  GGCTATCTGG  CTACCTAAGA  AAGTGGCTAT  CATCCATTGC  2700
     scaffold_22661  CAGATTTTGG  CTCTTTTGGA  GGCTATCTGG  CTACCTAAGA  AAGTGGCTAT  TATCCATTGC  2700
      scaffold_7076  CAGATTTTAG  CTCTTTTGGA  GGCTATCTGG  CTACCTAAGA  AAGTGGCTAT  TATCCATTGC  2700
     scaffold_22354  CAGATTTTGG  CTCTTTTGGA  GGCTATCTGG  CTACCTAAGA  AAGTGGCTAT  TATCCATTGC  2700
     scaffold_14383  CAGATTTTGG  CTCTTTTGGA  GGCTATCTGG  CTACCTAAGA  AAGTGGCTAT  TATCCATTGC  2700
                                            2,720                            2,740                       2,760
                                              |                                |                           |
Ancestral_Gamma_G1  CCCGGCCATC  AAAAAGGGGA  AGGAGCGGTA  GAAAAAGGAA  ACCGCTTGGC  AGATAAAACA  2760
  GeneScaffold_3363  CCTGGACACC  AAAATGCAGA  GACTGAGGCG  GCACAAGGAA  ATTCCTTTGC  AGATCAGATA  2760
      scaffold_1753  CCTGGCCATC  AAAAAGGGGA  AGGAGCGGTA  GAAAAAGGAA  ACCGCTTGGC  AGATAAAACA  2760
     scaffold_13050  CCCGGCCATC  AAAAAGGGGA  AGGAGCGGTA  GAAAAAGGAA  ACCGCTTGGC  AGATAAAACA  2760
     scaffold_24606  CCCGGCCATC  AAAAAGGGGA  AGGAGCGGTA  GAAAAAGGAA  ACCGCTTGGC  AGATAAAACA  2760
     scaffold_10119  CCCGGCCATC  AAAAAGGGGA  AAGAGCGGTA  GAAAAAGGAA  ACCGCTTGGC  AGATAAAACA  2760
     scaffold_23277  CCCGGCCATC  AAAAAGGGGA  AGGAGCGGTA  GAAAAAGGAA  ACCGCTTGGC  AGATAAAACA  2760
     scaffold_12630  CCCGGCCATC  AAAAAGGGGA  AAGAGCGGTA  GAAAAAAGAA  ACCGCTTGGC  AGATAAAACA  2760
      scaffold_2151  CCCGGCCATC  AAAAAGGGGA  AGGAGCGGTA  AAAAAAGGAA  ACCGCTTGGC  AGATAAAACA  2760
      scaffold_7237  CCCGGCCATC  AAAAAGGGGG  AGGAGCGGTA  GAAAAAGGAA  ACCGCTTGGC  AGATAAAACA  2760
     scaffold_12163  CCCGGCCATC  AAAAAGGGGA  AGGAGCGGTA  GAAAAAGGAA  ACCGCTTGGC  AGATAAAACA  2760
       scaffold_941  CCCAGCCATC  AAAAAGGGGA  AGAAGCGGTA  GAAAAAGGAA  ACTGCTTGGC  AGATAAAACA  2760
     scaffold_22661  CCCGGCCATC  AAAAAGGGAA  AGGAGCGGTA  GAAAAAGGAA  ACCGCTTGGC  AGATAAAACA  2760
      scaffold_7076  CCCGGCCATC  AAAAAGGGGA  AGGAGCGGTA  GAAAAAGGAA  ACCGCTTGGC  AGATAAAACA  2760
     scaffold_22354  CCCGGCCATC  AAAAAGGGAA  AGGAGCGGTA  GAAAAAGGAA  ACCGCTTGGC  AGATAAAACA  2760
     scaffold_14383  CCCGGCCATC  AAAAAGGGAA  AGGAGCGGTA  GAAAAAGGAA  ACCGCTTGGC  AGATAAAACA  2760
                                            2,780                            2,800                       2,820
                                              |                                |                           |
Ancestral_Gamma_G1  GCAAAGGAAG  TAGCCAGAAA  ACCAGTGGGG  ACTACTTGTG  CCCCCCTTGG  TACAGGCTCC  2820
  GeneScaffold_3363  GCAAAGGATG  CTGCCAGAAA  ACCAGTGGGG  CCCGCTTCCC  CCAGCCTTCG  AAGAAAGGCC  2820
      scaffold_1753  GCAAAGGAAG  TAGCCAGAAA  ACCAGTGGGG  ACTACTTGTG  CCCCCCTTGG  TACAGGCTCC  2820
     scaffold_13050  GCAAAGGAAG  TAGCCAGAAA  ACCAGTGGGG  ACTACTTGTG  CCCCACTTGG  TACAGGCTCC  2820
     scaffold_24606  GCAAAGGAAG  TAGCCAGAAA  ACCAGTGGGG  ACTACTTGTG  CCCCCCTTGG  TACAGGCTCC  2820
     scaffold_10119  GCAAAGGAAG  TAGCCAGAAA  ACCAGTGGGG  ACTACTTGTG  CCCCCCTTGG  CACAGGCTCC  2820
     scaffold_23277  GCAAAGGAAG  TAGCCAGAAA  ACCAGTGGGG  ACTACTTGTG  CCCCCCTTGG  TACAGGCTCC  2820
     scaffold_12630  GCAAAGGAAG  TAGCCAGAAA  ACCAGTGGGG  ACTACTTGTG  CCCCCCTTGG  CACAGGCTCC  2820
      scaffold_2151  GCAAAGGAAG  TAGCCAGAAA  ACCAGTGGGG  ACTACTTGTG  CCCCCCTTGG  CACAGGCTCT  2820
      scaffold_7237  GCAAAGGAAG  TAGCCAGAAA  ACCAGTGGGG  ACTACTTGCG  CCCCCCTTGG  CACAGGCTCC  2820
     scaffold_12163  GCAAAGGAAG  TAGCCAGAAA  ACCAGTGGGG  ACTACTTGTG  CCCCCCTTGG  TACAGGCTCC  2820
       scaffold_941  GCAAAGGAAG  TAGCCAGAAA  ACCAGTGGGG  ACTACTTGTG  CCCCCCTTGG  TACAGGCTTC  2820
     scaffold_22661  GCAAAGGAAG  TAGCCAGAAA  ACCAGTGGGG  ACTACTTGTG  CCCCCCTTGG  TACAGGCTCC  2820
      scaffold_7076  GCAAAGGAAG  TAGCCAGAAA  ACCAGTGGGG  ACTACTTGTG  CCCCCCTTGG  TACAGGCTCC  2820
     scaffold_22354  GCAAAGGAAG  TAGCCAGAAA  ACCAGTGGGG  ACTACTTGTG  CCCCCCTTGG  TACAGGCTCC  2820
     scaffold_14383  GCCAAGGAAG  TAGCCAGAAA  ACCAGTGGGG  ACTACTTGTG  CCCCCCTTGG  TACAGGCTCC  2820
                                            2,840                            2,860                       2,880
                                              |                                |                           |
Ancestral_Gamma_G1  AAACTATAGC  CCCCAAGATA  TAGAGTTGGG  GAAGCAACTG  CACGGGAAGT  TTGAACATGG  2880
  GeneScaffold_3363  TGAGTATACC  CCATTCGACT  TGGAATTAAG  TGAAAGACTG  GAAAAAGAGG  GAAAACAAGA  2880
      scaffold_1753  AAACTATAGC  CCCCAAGACA  TAGAGTTGGG  GAAGCAACTG  CACGAGAAGT  TTGAACATGG  2880
     scaffold_13050  AAACTATAGC  CCCCAAGATA  TAGAGTTGGG  GAAGCAACTG  CACGGGAAGT  TTGAACATGG  2880
     scaffold_24606  AAACTATAGC  CCCCAAGATA  TAAAGTTGGG  GAAGCAACTG  CATGGGAAGT  TTGAACATGG  2880
     scaffold_10119  AGACTATAGC  CCCCAAGATA  TAGAGTTGGG  GAAGCAACTG  CACGGGAAGT  TTGAACATGG  2880
     scaffold_23277  AAACTATAGC  CCCCAAGATA  TAGAGTTGGG  GAAGCAACTG  CACGAGAAGT  TTGAACATGG  2880
     scaffold_12630  AGACTATAGC  CCCCAAGATA  TAGAGTTGGG  GAAGCAACTG  CACAGGAAGT  TTGAACATGG  2880
      scaffold_2151  AGACTATAGC  CCCCAAGATA  TAGAGTTGGG  GAAACAACTG  CACGGGGAGT  TTGAACATGG  2880
      scaffold_7237  AGACTATAGC  CCCCAAGATA  TAGAGTTGGG  GAAGCAACTG  CACGGAAAGT  TTGAACATGG  2880
     scaffold_12163  AAACTATAGC  CCCCAAGATA  TAGAGTTGGG  GAAGCAACTG  CACGGGAAGT  TTGAACATGG  2880
       scaffold_941  AAACTATAGC  CCCCAAGATA  TAGAGTTGGG  GAAGCAACTG  CACGGGAAGT  TTGAACATGG  2880
     scaffold_22661  AAACTATAGC  CCCCAAGATA  TAGAGTTGGG  GAAGCAACTG  CACGGGAAGT  TTGAAAATGG  2880
      scaffold_7076  AAACTATAGC  CCCCAAGATA  TAGAGTTGGG  GAAGCAACTG  CACGGGAAGT  TTAAAAATGG  2880
     scaffold_22354  AAACTATAGC  CCCCAAGATA  TAGAGTTGGG  GAAGCAACTG  CACGGGAAGT  TTGAAAATGG  2880
     scaffold_14383  AAACTATAGC  CCCCAAGATA  TAGAGTTGGG  GAAGCAACTG  CACGGGAAGT  TTGAAAATGG  2880
```

			2,900		2,920	2,940	
Ancestral_Gamma_G1	GTGGCTTTGG	ATTCCAGATG	GAAGGCCGGT	CCTCCCTGCT	GCGCTTGGTA	ACACCTTAAT	2940
GeneScaffold_3363	ATGGAGAATG	CTCCCTGATA	GAAGATCGCT	CCTTCCTCAG	GCTTTGGGAA	AGACCTTAAT	2940
scaffold_1753	GTGGCTTTGG	ATTCCAGATG	GAAGGCCAGT	CCTCCCTGCT	GCGCTTGGTA	ACACCTTAAT	2940
scaffold_13050	GCGGCTTTGG	ATTCCAGATG	GAAGGCCGGT	CCTCCCTGCT	GCGCTCGGTA	ACACCTTAAT	2940
scaffold_24606	GTGGCTTTGG	ATTCCAGATG	GAAGGCCGGT	CCTCCCTGCT	GCGCTTGGTA	ACACCTTAAT	2940
scaffold_10119	GTGGCTTTGG	ATTCCAGATG	GAAGGCCGGT	CCTCCCTGCT	GCGCTCGGTA	ACTCCTTAAT	2940
scaffold_23277	GTGGCTTTGG	ATTCCAGATG	GAAGGCCAGT	CCTCCCTGCT	GCGCTTGGTA	ACACCTTAAT	2940
scaffold_12630	GTGGCTTTGG	ATTCCAGATG	GAAGGCCGGT	CCTCCCTGCT	GCGCTTGGTA	ACACCTTAAT	2940
scaffold_2151	GTGGCTTTGG	ATTCCAGATG	GAAGGCTGGT	CCTCCCTGCT	GTGCTTGGTA	ACACCTTAAT	2940
scaffold_7237	GTGGCTTTGG	ATTCCAGATG	GAAGGCCGGT	CCTCCCTGCT	GCGCTTGGTA	ACACCTTAAT	2940
scaffold_12163	GTGGCTTTGG	ATTCCAGATG	GAAGGCCGGT	CCTCCCTGCT	GCGCTCGGTA	ACACCTTAAT	2940
scaffold_941	GTGGCTTTGG	ATTCCAGATG	GAAGGCCGGT	CCTCCCTGCT	GTGCTTGGTA	ACACCTTAAT	2940
scaffold_22661	GTGGCTTTGG	ATTCCAGATG	GAAAGCCACT	CCTCCCTGCC	GCTCTTGGCA	GCCATTTAAT	2940
scaffold_7076	GTGGCTTTGG	ATTCCAGATG	GAAGGCCACT	CCTCCCTGCC	GCTCTTGGCA	GCCATTTAAT	2940
scaffold_22354	GTGGCTTTGG	ATTCCAGATG	GAAGGCCACT	CCTCCCTGCC	GCTCTTGGCA	GCCATTTAAT	2940
scaffold_14383	GTGGCTTTGG	ATTCCAGATG	GAAGGCCACT	CCTCCCTGCC	GCTCTTGGCA	GCCATTTAAT	2940

			2,960		2,980	3,000	
Ancestral_Gamma_G1	TAGCCAGATG	CATAATGCTA	CCCATCTTGG	AGGAACTAAG	TTAATTGAAC	TGCTAAAAAG	3000
GeneScaffold_3363	AGACTTAACA	CACCAGTCCA	CCCATCTAGG	AGGAACCAAG	TTAATTGAAC	TATTGAAAAG	3000
scaffold_1753	TAGCCAGATG	CATAATGCTA	CCCATCTTGG	AGGAACTAAG	TTAATTGAAC	TGCTAAAAAG	3000
scaffold_13050	TAGCCAGATG	CATAATGCTA	CCCATCTTGG	AGGAACTAAG	TTAATTGAAC	TGCTAAAAAG	3000
scaffold_24606	TAGCCAGATG	CATAATGCTA	CCCATCTTGG	AGGAACTAAG	TTAATTGAAC	TGCTAAAAAG	3000
scaffold_10119	TAGCCAGATG	CATAATGCTA	CCCATCTTGG	AGGAACTAAG	TTAATTGAAC	TGCTAAAAAG	3000
scaffold_23277	TAGCCAGATG	CATAACGCTA	CCCATCTTGG	AGGAACTAAA	TTAATCGAAC	TGCTAAAAAG	3000
scaffold_12630	TAGCCAGATG	CATAATGCTA	CCCATCTTGG	AGGAACTAAG	TTAATTGAAC	TGCTAAAAAG	3000
scaffold_2151	TAGCCAGATG	CATAATGCTA	CCCATCTTGG	AGGAACTAAG	TTAATTGAAC	TGCTAAAAAG	3000
scaffold_7237	TAGCCAGATG	CATAATGCTA	CCCATCTTGG	AGGAACTAAG	TTAATTGAAC	TGCTAAAAAG	3000
scaffold_12163	TAGCCAGATG	CATAATGCTA	CCCATCTTGG	AGGAACTAAG	TTAATTGAAC	TGCTAAAAAG	3000
scaffold_941	TAGCCAGATG	CATAATGCTA	CCCATCTTGG	AGGAACTAAG	TTAACTGAAC	TGCTAAAAAG	3000
scaffold_22661	CAATCAAATG	CATAACGCTA	CCCATCTTGG	AGGAACTAAG	TTAATTGAAC	TGCTAAAAAG	3000
scaffold_7076	CAATCAAATG	CATAACGCTA	CCCATCTTGG	AGGAACTAAG	TTAATTGAAC	TGCTAAAAAG	3000
scaffold_22354	CAATCAAATG	CATAACGCTA	CCCATCTTGG	AGGAACTAAG	TTAATTGAAC	TGCTAAAAAG	3000
scaffold_14383	CAATCAAATG	CATAACGCTA	CCCATCTTGG	AGGAACTAAG	TTAACTGAAC	TGCTAAAAAG	3000

			3,020		3,040	3,060	
Ancestral_Gamma_G1	AGACTATTAT	ATTCCTGGAC	TAACCTCAAA	GCTCGGGATG	TGAGTGCACG	ATGCCCAATA	3060
GeneScaffold_3363	GGATTATTAT	GTTCCAGGAC	TGAAAATTTA	GCCAAGGACG	TGGCTAATAG	GTGTGCAGTA	3060
scaffold_1753	AGACTCTTAT	ATTCCTGGAC	TAACCTCAAA	GCTCGGGATG	TGAGTGCACG	ATGCCCAATA	3060
scaffold_13050	AGACTATTAT	ATTCCTGGAC	TAACCTCAAA	GCTCGGGATG	TGAGTGCACG	ATGCCCAATA	3060
scaffold_24606	AGACTATTAT	ATTCCTGGAC	TAACCTCAAA	GCTCGGGATG	TGAGTGCACA	ATGCCCAATA	3060
scaffold_10119	AGACTATTAT	ATTCCTGGAC	TAACCTCAAA	GCTCGGGATG	TAAGTGCACG	ATGCCCAATA	3060
scaffold_23277	AGACTATTAT	ATTCCTGGAC	TAACCTCAAA	GCTCGGGATG	TGAGTGCACG	ATGCCCAATA	3060
scaffold_12630	AGACTATTAT	ATTCCTGGAC	TAACCTCAAA	GCTCGGGATG	TAAGTGCACG	ATGCCCAATA	3060
scaffold_2151	AGACTATTAT	ATTCCTGGAC	TAACCTCAAA	GCTCGGGATG	TGAGTGCACG	ATGCCCAATA	3060
scaffold_7237	AGACTATTAT	ATTCCTGGAC	TAACCTCAAA	GCTCGGGATG	TGAGTGCACG	ATGCCCAATA	3060
scaffold_12163	AGACTATTAT	ATTCCTGGAC	TAACCTCAAA	GCTCGGGATG	TAAGTGCACG	ATGCCCAATA	3060
scaffold_941	AGACTATTAT	GTTCCTGGAC	TAACCTCAAA	GCTCGGGATG	TGAGTGCACG	ATGCCCAATA	3060
scaffold_22661	AGACTATTAT	ATTCCTGGAC	TAACCTCAAA	GCTCGGGATG	TAAGTGCGCG	ATGCCCAATA	3060
scaffold_7076	AGACTATTAT	ATTCCTGGAC	TAACCTCAAA	GCTCGGGATG	TAAGTGCGCG	ATGCCCAATA	3060
scaffold_22354	AGACTATTAT	ATTCCTGGAC	TAACCTCAAA	GCTCGGGATG	TAAGTGCGCG	ACGCCCAATA	3060
scaffold_14383	AGACTATTAT	ATTCCTGGAC	TAACCTCAAA	GCTCGGGATG	TAAGTGCGCG	ATGCCCAATA	3060

			3,080		3,100	3,120	
Ancestral_Gamma_G1	TGTGCACAGG	TCAACTCTCG	CCCCATCCCA	AAGGAAAATG	GCACTCGATT	AAGAGGACAA	3120
GeneScaffold_3363	TATGCACAAG	TAGACTAAGG	AAGGACCTCC	ACGGAACAAG	GAGTGAGACT	CAGGGGAAAG	3120
scaffold_1753	TGTGCACAGG	TCAACTCTCG	CCCCATCCCA	AAGGAAAATG	GCACTCAATT	AAGAGGACAA	3120
scaffold_13050	TGTACACAGG	TCAACTCTCG	CCCCATCCCA	AAGGAAAATG	GCACTCGATT	AAGAGGACAA	3120
scaffold_24606	TGTGCACAGG	TCAACTCTCG	CCCCATCCCA	AAGGAAAATG	GCACTCGATT	AAGAGGACAA	3120
scaffold_10119	TGTGCACAGG	TCAACTCTCA	CCCCATCCCA	AAGGAAAATG	GCACTCGATT	AAGAGGACAA	3120
scaffold_23277	TGTGCACAGG	TCAACTCTCG	CCCCATCCCA	AAGGAAAATG	GCACTCGATT	AAGAGGACAA	3120
scaffold_12630	TGTGCACAGG	TCAACTCTCA	CCCCATCCCA	AAGGAAAATG	GCACTCGATT	AAGAGGACAA	3120
scaffold_2151	TGTGCACAGG	TCAACTCTCG	CCCCATCCCA	AAGGAAAATG	GCACTCGATT	AAGAGGACAA	3120
scaffold_7237	TGTGCACAGG	TCAACTCTCG	CCCCATCCCA	AAGGAAAATG	GCACTCGATT	AAGAGGACAA	3120
scaffold_12163	TGTGCACAGG	TCAACTCTCA	CCCCATCCCA	AAGGAAAATG	GCACTCGATT	AAGAGGACAA	3120
scaffold_941	TGCGCACAGG	TCAACTCTCG	CCCCATCCCA	AAGGAAAATG	GCACTCGATT	AAGAGGACAA	3120
scaffold_22661	TGTGCACAAG	TCAACTCTTG	CTCCACCGCA	ACAGGAGGCG	GCGTGCGACA	GAAAGGACAA	3120
scaffold_7076	TGTGCACAAG	TCAACTCTTG	CTCCACCGCA	ACAGGAGGCG	GCGTGCGACA	GAAAGGACAA	3120
scaffold_22354	TGCGCACAAG	TCAACTCTTG	CTCCACCGCA	ACAGGAGGCG	GCGTGCGACA	GAAAGGACAA	3120
scaffold_14383	TGTGCACAAG	TCAACTCTTG	CTCCACCGCA	ACAGGAGGCG	GCGTGCGACA	GAAAGGACAA	3120

```
                                             3,140                     3,160                     3,180
                                                 |                         |                         |
Ancestral_Gamma_G1  GCTCCTGGAG AGCATTGGGA AGTGGACTTT ACAGAAGTTG CTCTGGCACT TTTGGATTTA 3180
GeneScaffold_3363   AGACCTGGAG AGCACTGGGA GCTTGATTTT ACAGAAGTTA CGCTGGAACT TTTGGATTTA 3180
scaffold_1753       GCTCCTGGAG AGCATTGGGA AGTGGACTTT ACAGAAGTTG CTCTGGCACT TTTGGATTTA 3180
scaffold_13050      GCTCCTGGAG AGCATTGGGA AGTGGACTTT ACAGAAGTTG CTCTGGCACT TTTGGATTTA 3180
scaffold_24606      GCTCCTGGAG AGCATTGGGA AGTGGACTTT ACAGAAGTTG ATCTGGCACT TTTGGATTTA 3180
scaffold_10119      GCTCCTGGAG AGCATTGGGA AGTGGACTTT ACAGAAGTTG CTCTGGCACT TTTGGATTTA 3180
scaffold_23277      GCTCCTGGAG AGCATTGGGA AGTGGACTTT ACAGAAGTTG CTCTGGCACT TTTGGATTTA 3180
scaffold_12630      GCTCCTGGAG AGCATTGGGA AGTGGACTTT ACAGAAGTTG CTCTGGCACT TTTGGATTTA 3180
scaffold_2151       GCTCCTGGAG AGCATTGGGA AGTGGACTTT ACAGAAGTTG CTCTGGCACT TTTGGATTTA 3180
scaffold_7237       GCTCCTGGAG AGCATTGGGA AGTGGACTTT ACAGAAGTTG CTCTGGCACT TTTGGATTTA 3180
scaffold_12163      GCTCCTGGAG AGCATTGGGA AGTGGACTTT ACAGAAGTTG CTCTGGCACT TTTGGATTTA 3180
scaffold_941        GCTCCTGGAG AGCATTGGGA AGTGGACTTT ACAGAAGTTG CTCTGGCACT TTTGGATTTA 3180
scaffold_22661      GCTCCTGGAG AACATTGGGA GGTAGACTTT ACAGAAGTTG CCCTGGCACT TTCGGGTTTA 3180
scaffold_7076       GCTCCTGGAG AACATTGGGA GGTAGACTTT ACAGAAGTTG CCCTGGCACT TTCGGGTTTA 3180
scaffold_22354      GCTCCTGGAG AACATTGGGA GGTAGACTTT ACAGAAGTTG CCCTGGCACT TTCGGGTTTA 3180
scaffold_14383      GCTCCTGGAG AACATTGGGA GGTAGACTTT ACAGAAGTTG CCCTGGCCCT TTCGGGTTTA 3180

                                             3,200                     3,220                     3,240
                                                 |                         |                         |
Ancestral_Gamma_G1  AATATCTTCT GGTATTCATT GACACCTATT CTGGATGGAC TGAGGCCTTC CCCACAAAGA 3240
GeneScaffold_3363   AGTACCTATT AGTTTTTATT GACACATACT CTGGATGGAC TGAGGCCTAT CCCACAAAGA 3240
scaffold_1753       AATATCTTCT GGTATTCATT GACACCTATT CTGGATGGAC TGAGGCCTTC CCCACAAAGA 3240
scaffold_13050      AATATCTTCT GGTATTTATT GACACCTATT CTGGATGGAC TGAGGCCTTC TCCACAAAAA 3240
scaffold_24606      AATATCTTCT GGTATTCATT GACACCTATT CTGGATGGAC TGAGGCCTTC CCCACAAAGA 3240
scaffold_10119      AATATCTTCT GGTATTCATT GACACCTATT CTGGATGGAC TGAGGCCTTC CCCACAAAGA 3240
scaffold_23277      AATATCTTCT GGTATTCATT GACACCTATT CTGGATGGAC TGAGGCCTTC CCCACAAAGA 3240
scaffold_12630      AATATCTTCT GGTATTCATT GACACCTATT CTGGATGGAC TGAGGCCTTC CCCACAAAGA 3240
scaffold_2151       AATATCTTCT GGTATTCATT GACACCTATT CTGGATGGAC TGAGGCCTTC CCCACAAAGA 3240
scaffold_7237       AATATCTTCT GGTATTCATT GACACCTATT CTGGATGGAC TGAGGCCTTC CCCACAAAGA 3240
scaffold_12163      AATATCTTCT GGTATTCATT GACACCTATT CTGGATGGAC TGAGGCCTTC CCCACAAAGA 3240
scaffold_941        AATATCTTCT GGTATTCATT GACACCTATT CTGGATGGAC TGAGGCCTTC CCCACAAAGA 3240
scaffold_22661      AGTATCTTCT GGTTTTCATC GACACCTACT CTGGATGGAC CAAGGCCTTC CCCACTAGAA 3240
scaffold_7076       AGTATCTTCT GGTTTTCATT GACACCTACT CTGGATGGAC CGAGGCCTTC CCCACTAGAA 3240
scaffold_22354      AGTATCTTCT GGTTTTCATC GACACCTACT CTGGATGGAC CGAGGCCTTC CCCACTAGAA 3240
scaffold_14383      AGTATCTTCT GGTTTTCATC GACACCTACT CTGGATGGAC CGAGGCCTTC CCCACTAGAA 3240

                                             3,260                     3,280                     3,300
                                                 |                         |                         |
Ancestral_Gamma_G1  AGGAAACTGC ACAAGTAGTA GCTAAGAAAT TAAAGAAATA GTGCCTCGAT TTGGCTTGCC 3300
GeneScaffold_3363   AGGAAACTGC ACAAGTAGTA GCTAAGAAAT TAAAGAAATA GTGCCTCGAT TTGGCTTGCC 3300
scaffold_1753       AGGAAACTGC ACAAGTAGTA GCTAAGAAAT TAAAGAAATA GTGCCTCGAT TTGGCTTGCC 3300
scaffold_13050      AGGAAACTGC ACAAGTAGTA GCTAAGAAAT TAAAGAAATA GTGCCTCTAT TTGGCTTGCC 3300
scaffold_24606      AGGAAACTGC ACAAGTAGTA GCTAAGAAAT TAAAGAAATA GTGCCTCGAT TTGGCTTGCC 3300
scaffold_10119      AGGAAACTGC ACAAGTAGTA GCTAAGAAAT TAAAGAAATA GTGCCTCGAT TTGGCTTGCC 3300
scaffold_23277      AGGAAACTGC ACAAGTAGTA GCTAAGAAAT TAAAGAAATA GTGCCTCGAT TTGGCTTGCC 3300
scaffold_12630      AGGAAACTGC ACAAGTAGTA GCTAAGAAAT TAAAGAAATA GTGCCTAGAT TTGGCTGGTC 3300
scaffold_2151       AGGAAACTGC ACAAGTAGTA GCTAAGAAAT TAAAGAAATA GTGCCTCGAT TTGGCTGGCC 3300
scaffold_7237       AGGAAACTGC ACAAGTAGTA GCTAAGAAAT TAAAGAAATA GTGCCTCGAT TTGGCTGGCC 3300
scaffold_12163      AGGAAACTGC ACAAGTAGTA GCTAAGAAAT TAAAGAAATA GTGCCTCGAT TTGGCTTGCC 3300
scaffold_941        AGGAAACTGC ACAAGTAGTA GCTAAGAAAT TAAAGAAATA GTGCCTCGAT TCGGTTTGCC 3300
scaffold_22661      AGGAGACTGC ACAAGTAGTA GCTAAGAAAT TACAGAAATA GTACCTCCAT TCGGTTTGCC 3300
scaffold_7076       AGGAGACTGC ACAAGTAGTA GCTAAGAAAT TACAGAAATA GTACCTCGAT TCGGTTTGCC 3300
scaffold_22354      AGGAGACTGC ACAAGTAGTA GCTAAGAAAT TACAGAAATA GTACCTCGAT TCGGTTTGCC 3300
scaffold_14383      AGGAGACTGC ACAAGTAGTA GCTAAGAAAT TACAGAAATA GTACCTCGAT TCGGTTTGCC 3300

                                             3,320                     3,340                     3,360
                                                 |                         |                         |
Ancestral_Gamma_G1  CTTATCATTG GGGTCCGACA ATGGCCCGGC ATTCATTGCC CAAACAAGTC AAATGTTAGC 3360
GeneScaffold_3363   CTTATCATTG GGGTCCGACA ATGGCCCGGC ATTCATTGCC CAAACAAGTC AAATGTTAGC 3360
scaffold_1753       CTTATCATTG GGGTCCGACA ATGGCCCGGC ATTCATTGCC CAAACAAGTC AAATGTTAGC 3360
scaffold_13050      CTTACCATTG GGGTCCGACA ATGGCCCGGC ATTCATTGCC CAAACAAGTC AAATGTTAGC 3360
scaffold_24606      CTTATCATTG GGGTCCGACA ATGGCCCGGC ATTCATTGCC CAAACAAGTC AAATGTTAGC 3360
scaffold_10119      CTTATCATTG GGGTCCGACA ATGGCCCGGC ATTCATTGCC CAAACAAGTC AAATGTTAGC 3360
scaffold_23277      CTTATCATTG GGGTCCGACA ATGGCCCGGC ATTCATTGCC CAAACAAGTC AAATGTTAGC 3360
scaffold_12630      CTTATCATTG GGGTCCGACA ATGGCCCGGC ATTCATTGCC CAAACAAGTC AAATGTTAGC 3360
scaffold_2151       CTTATCATTG GGGTCCGACA ATGGCCCGGC ATTCATTGCC CAAACAAGTC AAATGTTAGC 3360
scaffold_7237       CTTATCATTG GGGTCCGACA ATGGCCCGGC ATTCATTGCC CAAACAAGTC AAATGTTAGC 3360
scaffold_12163      CTTATCATTG GGGTCCGACA ATGGCCCGGC ATTCATTGCC CAAACAAGTC AAATGTTAGC 3360
scaffold_941        CTTATCACTG GAGTCCGACA ATGGCCCGGC ATTCATTGCC CAAACAAGTC AAATGTTAGC 3360
scaffold_22661      CTTATCACTG GGGTCCGACA ATGGCCCGGC ATTCATTGCC CAAACAAGTC AAATGTTAGC 3360
scaffold_7076       CTTATCACTG GGGTCCGACA ATGGCCCGGC ATTCATTGCC CAAACAAGTC AAATGTTAGC 3360
scaffold_22354      CTTATCACTG GGGTCCGACA ATGGCCCGGC ATTCATTGCC CAAACAAGTC AAATGTTAGC 3360
scaffold_14383      CTTATCACTG GGGTCCGACA ATGGCCCGGC ATTCATTGCC CAAACAAGTC AAATGTTAGC 3360
```

```
                                           3,380                         3,400                         3,420
Ancestral_Gamma_G1  TAAGGTATTA GGGATTAATT GGAAATTACA TTGTATTTAC AGACCTCAGA GTTCAGGACA 3420
GeneScaffold_3363   TAAGGTATTA GGGATTAATT GGAAATTACA TTGTATTTAC AAACCTCAGA GTTCAGGACA 3420
scaffold_1753       TAAGGTATTA GGGATTAATT GGAAATTACA TTGTATTTAC AGACCTCAGA GTTCAGGACA 3420
scaffold_13050      TAAGGTATTA GGGATCAATT GGAAATTACA TTGTATTTAC AGACCTCAGA GTTCAGGACA 3420
scaffold_24606      TAAGGTATTA GGGATCAATT GGAAATTACA TTGTATTTAC AGACCTCAGA GTTCAGGACA 3420
scaffold_10119      TAAGGTATTA GGGATTAATT GGAAATTACA TTGTATTTAC AGACCTCAGA GTTCAGGACA 3420
scaffold_23277      TAAGGTATTA GGGATCAATT GGAAATTACA TTGTATTTAC AGACCTCAGA GTTCAGGACA 3420
scaffold_12630      TAAGGTATTA GGGATTAATT GGAAATTACA TTGTATTTAC AGACCTCAGA GTTCAGGACA 3420
scaffold_2151       TAAGGTATTA GGGATCAATT GGAAATTACA TTGTATTTAC AGACCTCAGA GTTCAGGACA 3420
scaffold_7237       TAAGGTATTA GGGATCAATT GGAAATTACA TTGTATTTAC AAACCTCAGA GTTCAGGACA 3420
scaffold_12163      TAAGGTATTA GGGATTAATT GGAAATTACA TTGTATTTAC AGACCTCAGA GTTCAGGACA 3420
scaffold_941        TAAGGTATTA GGGATTAATT GGAAATTACA TTGTATTTAC AGACCTCAGA GTTCAGGACA 3420
scaffold_22661      TAAGGTATTA GGGATTAGTT GGAAATTACA TTGTATTTAC AGACCTCAGA GTTCAGGACA 3420
scaffold_7076       TAAGGTATTA GGGATTAATT GGAAATTACA TTGTATTTAC AGACCTCAGA GTTCAGGACA 3420
scaffold_22354      TAAGGTATTA GGGATTAATT GGAAATTACA TTGTATTTAC AGACCTCAGA GTTCAGGACA 3420
scaffold_14383      TAAGGTATTA GGGATTAGTT GGAAATTACA TTGTATTTAC AGACGTCAGA GTTCAGGACA 3420
                                           3,440                         3,460                         3,480
Ancestral_Gamma_G1  AGTAGAAAGG ATGAATAGAA CACTTAAGGA AACTAACTAA ATTTAAGCTG GAAACTGGGG 3480
GeneScaffold_3363   AGTAGAAAGG ATGAATAGAA CACTTAAGGA AACTAACTAA ATTTAAGCTG GAAACTGGGG 3480
scaffold_1753       AGTAGAAAGG ATGAATAGAA CACTTAAGGA AACTAACTAA ATTTAAGCTG GAAACTGAGG 3480
scaffold_13050      AGTAAAAAGG ATGAATAGAA CACATAAGGA AACTAACTAA ATTTAAGGTG GAAACTGGGG 3480
scaffold_24606      AGTAGAAAGG ATGAATAGAA CACTTAAGGA AACTAACTAA ATTTAAGCTG GAAACTGGGG 3480
scaffold_10119      AGTAGAAAGG ATGAATAGAA CAATTAAGGA AACTAACTAA ATTTAAGCTG GAAACTGGGG 3480
scaffold_23277      AGTAGAAAGG ATGAATAGAA CACTTAAGGA AACTAACTAA GTTTAAGCTG GAAACTGGGG 3480
scaffold_12630      AGTAGAAAGG ATGAATAGAA CACATAAGGA AACTAACTAA ATTTAAGCTG GAAACTGGGG 3480
scaffold_2151       AGTAGAAAGG ATGAATAGAA CACTTAAGGA AACTAACTAA ATTTAAGCTG GAAACTGGGG 3480
scaffold_7237       AGTAGAAAGG ATGAATAGAA CACTTAAGGA AACTAACTAA ATTTAAGCTG GAAACTGGGG 3480
scaffold_12163      AGTAGAAAGG ATGAATAGAA CACTTAAGGA AACTAACTAA ATTTAAGCTG GAAACTGGGG 3480
scaffold_941        AGTAGAAAGG ATGAATAGAA CACTTAAGGA AACTAACTAA ATTTAAGCTG GAAACCGGGG 3480
scaffold_22661      AGTAGAAAGG ATGAATAGAA CACTTAAGGA AACTAACTAA ATTTAAGCTG GAAACTGGGG 3480
scaffold_7076       AGTAGAAAGG ATGAATAGAA CACTTAAGGA AACTAACTAA ATTTAAGCTG GAAACTGGGG 3480
scaffold_22354      AGTAGAAAGG ATGAATAGAA CACTTAAGGA AACTAACTAA ATTTAAGCTG GAAACTGGGG 3480
scaffold_14383      AGTAGAAAGG ATGAATAGAA CACTTAAGGA AACTAACTAA ATTTAAGCTG GAAACTGGGG 3480
                                           3,500                         3,520                         3,540
Ancestral_Gamma_G1  AGAATTGGGT GAGCCTTCTT CCTTTTGCTT TGTTACGGGC CCGGTGTACT CCATATATTA 3540
GeneScaffold_3363   AGAATTGGGT GAGCCTTCTG CCTTTTGCTT TGTTACGGGC CCGGTGTACT CCATATATTA 3540
scaffold_1753       AGAATTGGGT GAGCCTTCTT CCTTTTGCTT TGTTACGGGC CCGGTGTACT CTATATATTA 3540
scaffold_13050      AGAATTGGGT GAGCGTTCTT CCTTTTGCTT TGTTACGGGC CCGGTGTACT CCATATATTA 3540
scaffold_24606      AGAATTGGGT GAGCCTTCTG CCTTTTGCTT TGTTACGGGC CCGGTGTACT CCATATATTA 3540
scaffold_10119      AGAATTGGGT GAGCCTTCTT CCTTTTGCTT TGTTACGGGC CCGGTGTACT CCATATATTG 3540
scaffold_23277      AGAATTGGGT GAGCCTTCTT CCTTTTGCTT TGTTATGGGC CCAGTGTACT CCATATATTA 3540
scaffold_12630      AGAATTGGGT GAGCCTTCTT CCTTTTGCTT TGTTATGGGC CCAGTGTACT CCATATATTA 3540
scaffold_2151       AGAATTGGGT GAGCCTTCTT CCTTTTGCTT TGTTATGGGC CCAGTGTACT CCATATATTA 3540
scaffold_7237       AGAATTGGGT GAGCCTTCTG CCTTTTGCTT TGTTACGGGC CCGGTGTACT CCATATATTA 3540
scaffold_12163      AGAATTGGGT GAGCCTTCTG CCTTTTGCTT TGTTACGGGC CCGGTGTACT CCATATATTA 3540
scaffold_941        AGAATTGGGT GAGCCTTCTT CCTTTTGCTT TGTTACGGGC CCGGTGTACT CCATATATTA 3540
scaffold_22661      AGAATTGGGT GAGCCTTCTT CCTTTTGCTT TGTTAAGGGC CCGGTGTACT CCATATATTA 3540
scaffold_7076       AGAATTGGGT GAGCCTTCTT CCTTTTGCTT TGTTACGGGC CCAGTGTACT CCATATATTA 3540
scaffold_22354      AGAATTGGGT GAGCCTTCTT CCTTTTGCTT TGTTACGGGC CCGGTGTACT CCATATATTA 3540
scaffold_14383      AGAATTGGGT GAGCCTTCTT CCTTTTGCTT TGTTACGGGT CCGGTGTACT CCATATATTA 3540
                                           3,560                         3,580                         3,600
Ancestral_Gamma_G1  TGGGAATTTC CCCTTATGAA ATGTTTGGGA GACCTCCCCC TTTACTCCCT AAAATTGGAA 3600
GeneScaffold_3363   TGGGAATTTC CCCTTATGAA ATGTTTGGGA GACCTCCCCC TTTACTCCCT AAAATTGGAA 3600
scaffold_1753       TGGGAATTTC CCCTTGTGAA ATGTTTGGGA GACCTCCCCC TTTACTCCCT AAAATTGGAA 3600
scaffold_13050      TGGGAATTTC CCCTTATGAA ATGTTTGGGA GACCTCCCCC TTTACTCCCT AAAATTGGAA 3600
scaffold_24606      TGGGAATTTC CCCTTATGAA ATGTTTGGGA GACCTCCCCC TTTACTCCCT AAAATTGGAA 3600
scaffold_10119      TGGGAATTTC CCCTTATGAA ATGTTTGGGA GACCTCCCCC TTTACTCCCT AAAATTGGAA 3600
scaffold_23277      TGGGAATTTC CCCTTATGAA ATGTTTGGGA GACCTCCCCC TTTACTCCCT AAAATTGGAA 3600
scaffold_12630      TGGGAATTTC CCCTTATGAA ATGTTTGGGA GACCTCCCCC TTTACTCCCT AAAATTGGAA 3600
scaffold_2151       TGGGAATTTC CCCTTATGAA ATGTTTGGGA GACCTCCCCC TTTACTCCCT AAAATTGGAA 3600
scaffold_7237       TGGGAATTTC CCCTTATGAA ATGTTTGGGA GATCTCCCCC TTTACTCCCT AAAATTGGAA 3600
scaffold_12163      TGGGAATTTC CCCTTATGAA ATGTTTGGGA GACCTCCCCC TTTACTCCCT AAAATTGGAA 3600
scaffold_941        TGGGAATTTC CCCTTATGAA ATGTTTGGGA GACCTTCCCC TTTACTCCCT AAAATTGGAA 3600
scaffold_22661      TGGGAATTTC CCCTTATGAA ATGTTTGGGA GACCTCTCCC TTTATTCCCT AAAATTGGAA 3600
scaffold_7076       TGGGAATTTC CCCTTATGAA ATGTTTGGGA GACCTCCCCC TTTACTCCCT AAAATTGGAA 3600
scaffold_22354      TGGGAATTTC CCCTTATGAA ATGTTTGGGA GACCTCCCCC TTTACTCCCT AAAATTGGAA 3600
scaffold_14383      TGGGAATTTC CCCTTATGAA ATGTTTGGGA GACCTCCCCC TTTACTCCCT AAAATTGGAA 3600
```

```
                                           3,620                           3,640                       3,660
                                               |                               |                           |
Ancestral_Gamma_G1  TTGAAAAGGT  TACAATGAGT  AATAATACAA  GTCTCCTTAA  GTCTCTACAG  GCCTTGCAAC  3660
 GeneScaffold_3363  TTGAAAAGGT  TACAATGAGT  AATAATACAA  GTCTCCTTAA  GTCTTTACAG  GCCTTGCAAC  3660
     scaffold_1753  TTGAAAAGGT  TACAATGAGT  AATAATACAA  GTCTCCTTAA  GTCTCTACAG  GCCTTGCAAC  3660
    scaffold_13050  TTGAAAAGGT  TACAATGAGT  AATAATACAA  GTCTCCTTAA  GTCTCTACAG  GCCTTGCAAC  3660
    scaffold_24606  TTGAAAAGGT  TACAATGAGT  AATAATACAA  GTCTCCTTAA  GTCTCTACAG  GCCTTGCAAC  3660
    scaffold_10119  TTGAAAAGGT  TACAATGAGT  AATAATACAA  GTCTCCTTAA  GTCTCTACAG  GCCTTGCAAC  3660
    scaffold_23277  TTGAAAAGGT  TACAATAAGT  AATAATACAA  GTCTCCTTAA  GTCTCTACAG  GCCTTGCAAC  3660
    scaffold_12630  TTGAAAAGGT  TACAATGAGT  AATAATACAA  GTCTCCTTAA  GTCTCTACAG  GCCTTGCAAC  3660
     scaffold_2151  TTGAAAAGGT  TACAATGAGT  AATAATACAA  GTCTCCTTAA  GTCTCTACAG  GCCTTGCAAC  3660
     scaffold_7237  TTGAAAAGGT  TACAATGAGT  AATAATACAA  GTCTCCTTAA  GTCTCTACAG  GCCTTGCAAC  3660
    scaffold_12163  TTGAGAAGGT  TACAATGAGT  AATAATACAA  GTCTCCTTAA  GTCTCTACAG  GCCTTGCAAC  3660
      scaffold_941  TTGAAAAGGT  TACAATAAGT  AATAACACAA  GTCTCCTTAA  GTCTCTACAG  GCCTTGCAAC  3660
    scaffold_22661  TTGAAAAGGT  TACAATAAGT  AATAACACAA  GTCTCCTTAA  GTCTCTACAG  GCCTTGCAAC  3660
     scaffold_7076  TTGAAAAGGT  TACAATAAGT  AATAACACAA  GTCTCCTTAA  GTCTCTACAG  GCCTTGCAAC  3660
    scaffold_22354  TTGAAAAGGT  TACAATAAGT  AATAACACAA  GTCTCCTTAA  GTCTCTACAG  GCCTTGCAAC  3660
    scaffold_14383  TTGAAAAGGT  TACAATAAGT  AATAACACAA  GTCTCCTTAA  GTTTCTACAG  GCCTTGCAAC  3660
                                           3,680                           3,700                       3,720
                                               |                               |                           |
Ancestral_Gamma_G1  AAACCCAAAA  TCAAGTCAAG  GGCTTGATTA  AAGCTGCTAG  ACCGACTCCG  GGGACCTCAC  3720
 GeneScaffold_3363  AAACCCAAAA  TCAAGTCAAG  GGCTTGATTA  AAGCTGCTAG  ACCGACTCCG  GGGACCTCAC  3720
     scaffold_1753  AAACCCAAAA  TCAAGTCAAG  GGCTTAATTA  AAGCTGCTAG  ACCAACTCCG  GGGACCTCAC  3720
    scaffold_13050  AAACCCAAAA  TCAAGTCAAG  GGCTTGATTA  AAGCTGCTAG  ACCGACTCCG  GGGACCTCAC  3720
    scaffold_24606  AAACCCAAAA  TCAAGTCGAG  GGCTTGATTA  AAGCTGCTAG  ACCGACTCCG  GGGACCTCAC  3720
    scaffold_10119  AAACCCAAAA  TCAAGTCAAG  GGCTTGATTA  AAGCTGCTAG  ACCGACTCCG  GGGACCTCAC  3720
    scaffold_23277  AAACCCAAAA  TCAAGTCAAG  GGCTTGATTA  AAGCTGCTAG  ACCGACTCCG  GGGACCTCAC  3720
    scaffold_12630  AAACCCAAAA  TCAAGTCAAG  GGCTTGATTA  AAGCTGCTAG  ACCGACTCCG  GGGACCTCAC  3720
     scaffold_2151  AAACCCAAAA  TCAAGTCGAG  GGCTTGATTA  AAGCTGCTAG  ACCGACTCCG  GGGACCTCAC  3720
     scaffold_7237  AAACCCAAAA  TCAAGTCAAG  GGCTTGATTA  AAGCTGCTAG  ACCGACTCCG  GGGACCTCAC  3720
    scaffold_12163  AAACCCAAAA  TCAAGTCGAG  GGCTTGATTA  AAGCTGCTAG  ACCGACTCCG  GGGACCTCAC  3720
      scaffold_941  AAACCCAAAA  TCAAGTCAAG  GGCTTGATTA  AAGCTACTAG  ACTGACTCCG  GGGACCTCAC  3720
    scaffold_22661  AAACCCAAAA  TCAAGTCAAG  GGCTTGATTA  AAGCTACTAG  ACCGACTCTG  GGGACCTCAC  3720
     scaffold_7076  AAACCCAAAA  TCAAGTCAAG  GGCTTGATTA  AAGCTACTAG  ACCGACTCCG  GGGACCTCAC  3720
    scaffold_22354  AAACCCAAAA  TCAAGTCAAG  GGCTTGATTA  AAGCTACTAG  ACCGACTCCG  GGGACCTCAC  3720
    scaffold_14383  AAACCCAAAA  TCAAGTCAAG  GGCTTGATTA  AAGCTACTAG  ACCGACTCCG  GGGACCTCAC  3720
                                           3,740                           3,760                       3,780
                                               |                               |                           |
Ancestral_Gamma_G1  CCACATCATC  CAGGCGATTG  GGTGTGGATA  AAGAAGATCC  AGCCTGCTAG  CCTAGAGCCC  3780
 GeneScaffold_3363  CCACATCATC  CAGGCGATTG  GGTGTGGATA  AAGAAGATCC  AGCCTGCTAG  CCTAGAGCCC  3780
     scaffold_1753  CCACATCATC  CAGGTGATTG  GGTGTGGATA  AAGAAGATCC  AGCCTGCTAG  CCTAGAGCCC  3780
    scaffold_13050  CCACATCATC  CAGGCGATTG  GGTGTGGATA  AAGAAGATCC  AGCCTGCTAG  CCTAGAGCCC  3780
    scaffold_24606  CCACATCATC  CAGGCGATTG  GGTGTGGATA  AAGAAGATCC  AGCCTGCTAG  CCTACAGCCC  3780
    scaffold_10119  CCACATCATC  CAGGTGATTG  GGTGTGGATA  AAGAAGATCC  AGCCTGCTAG  CTTAGAGCCT  3780
    scaffold_23277  CCACATCATC  CAGGTGATTG  GGTGTGGATA  AAGAAGATCC  AGTCTGCTAG  CCTAGAGCCC  3780
    scaffold_12630  CCACATCATC  CAGGCGATTA  GGTGTGGATA  AAGAAGATCC  AGCCTGCTAG  CCTAGAACCC  3780
     scaffold_2151  CCACATCATC  CAGGCGATTG  GGTGTGGATA  AAGAAGATCC  AGCCTGCTAG  CCTAGAGCCC  3780
     scaffold_7237  CCACATCATC  CAGGCGATTG  GGTGTGGATA  AAGAAGATCC  AGCCTGCTAG  CCTAGAGCCC  3780
    scaffold_12163  CCACATCATC  CAGGCGATTG  GGTGTGGATA  AAGAAGATCC  AGCCTGCTAG  CCTAGAGCCC  3780
      scaffold_941  CCACATCATC  CAGGTGATTG  GGTGTGGATA  AAGAAGATTC  AGCCTGCTAG  CCTAGAGCCC  3780
    scaffold_22661  CCACATCATC  CAGGTGATTG  GGTGTGGATA  AAGAAGATTC  AGCCCGCTAG  CCTAGAGCCC  3780
     scaffold_7076  CCACATCATC  CAGGTGATTG  GGTGTGGATA  AAGAAGATTC  AGCCTCCTAG  CCTAGAGCCC  3780
    scaffold_22354  CCACATCATC  CAGGCGATTG  GGTGTGGATA  AAGAAGATTC  AGCCTGCTAG  CCTAGAGCCC  3780
    scaffold_14383  CCACATCATC  CAGGTGATTG  GGTGTAGATA  AAGAAGATTC  AGCCTGCTAG  CCTAGAGCCC  3780
                                           3,800                           3,820                       3,840
                                               |                               |                           |
Ancestral_Gamma_G1  CGGTGGGATG  GTCCGTTTCC  AGTAATCCTG  ACCACACCAA  CGGCTGTCAA  AGTTGCAGGA  3840
 GeneScaffold_3363  CGGTGGGATG  GTCCGTTTCC  AGTAATCCTG  ACCACACCAA  CGGCTGTCAA  AGTTGCAGGA  3840
     scaffold_1753  CAGTGGGATG  GTCCGTTTCC  AGTAATCCTG  ACCACACCAA  CGGCTGTCAA  AGTTGCAGGA  3840
    scaffold_13050  CGGTGGGATG  GTCCATTTCC  AGTAATCCTG  ACCACACCAA  CGGCTGTCAA  AGTTGCAGGA  3840
    scaffold_24606  CGGTGGGATG  GTCCGTTTCC  AGTAACTCTA  ACCACACCAA  CAGCTGTCGA  GGTTGCAGGG  3840
    scaffold_10119  CAGTGGGATG  GTCCGTTTCC  AGTAACTCTA  ACCACACCAA  CAGCTGTCGA  GGTTGCAGGG  3840
    scaffold_23277  CGGTGGGATG  GTCCGTTTCC  AGTAACTCTA  ACCACACCAA  CAGCTGTCGA  GGTTGCAGGG  3840
    scaffold_12630  CGGTGGGATG  GTCCGTTTCC  AGTAATCCTG  ACCACACCAA  CGGCTGTCAA  AGTTGCAGGA  3840
     scaffold_2151  CGGTGGGATG  GTCCGTTTCC  AGTAATCCTG  ACCACACCAA  CGGCTGTCAA  AGTTGCAGGA  3840
     scaffold_7237  CGGTGGGATG  GTCCATTTCC  AGTAATCCTG  ACCACACCAA  CGGCTGTCAA  AGTTGCAGGA  3840
    scaffold_12163  CGGTGGGATG  GTCCGTTTCC  AGTAACTCTA  ACCACACCAA  CAGCTGTCGA  GGTTGCAAGG  3840
      scaffold_941  CGGTGGGATG  GTCCGTTTCC  AGTAATCCTG  ACCACACCAA  CGGCTGTCAA  AGTTGCAGGA  3840
    scaffold_22661  CGGAGGGATG  GTCCGTTTCC  AGTAATCCTG  ACCACACCAA  CGGCTGTCAA  AGTTGCAGGA  3840
     scaffold_7076  CGGTGGGATG  GTCCGTTTCC  AGTAATCCTG  ACCACACCAA  CGGCTGTCAA  AGTTGCAGGA  3840
    scaffold_22354  CGGTGGGATG  GTCCGTTTCC  AGTAATCCTG  ACCACACCAA  CGGCTGTCAA  AGTTGCAGGA  3840
    scaffold_14383  CGGTGGGATG  GTCCGTTTCC  AGTAATCCTG  ACCACACCAA  CGGCTGTCAA  AGTTGCAGGA  3840
```

```
                                              3,860                                    3,880                                    3,900
                                                |                                        |                                        |
Ancestral_Gamma_G1  AAAAGACATC  ACCATACCCG  CCTTAAGACA  GCTCAACCTC  CCCCGGAGAC  AGAGAAATGG  3900
GeneScaffold_3363   AAAAGACATC  ACCATACCCA  CCTTAAGACA  GCTCAACCTC  CCCCGGAGAC  AGAGAAATGG  3900
scaffold_1753       AAAAGACATC  ATCATACTCA  TATTAAGACA  GCACAACCTC  CCCCGGAGAC  AGAGAAATGG  3900
scaffold_13050      AAAAGACATC  ACCATACCCG  CCTTAAGACA  GCTCAACCTC  CCCCGGAGAC  AGAGAAATGG  3900
scaffold_24606      AAAAAAACATC ACCATACCCG  CCTTAAGACA  GCTCAACCTC  CCCCGGAGAC  AGAGAAATGG  3900
scaffold_10119      AAAAGACATC  ACCATACCTG  CCTTAAGACA  GCTCAACCTC  CCCCGGAGAC  AGAGAAATGG  3900
scaffold_23277      AAAAGACATC  ACCATACCCG  CCTTAAGACA  GCTCAACCTC  CCCCGGAGAC  AGAGAAATGG  3900
scaffold_12630      AAAAGACATC  ACCATACCCG  CCTTAAGACA  GCACAACCTC  CCCCGGAGAC  AGAGAAATGG  3900
scaffold_2151       AAAAGACATC  ACCATACCCG  CCTTAAGACA  GCTCAACCTC  CCCCGGAGAC  AGAGAAATGG  3900
scaffold_7237       AAAAGACATC  ACCATACCCG  CCTTAAGACA  GCTCAACCTC  CCCCGGAGAC  AGAGAAATGG  3900
scaffold_12163      AAAAGACATC  ACCATACCCG  CCTTAAGACA  GCTCAACCTC  CCCCGGAGAC  AGAGAAATGG  3900
scaffold_941        AAAAGACATC  ACCGTACTCG  CCTTAAGGCA  GCTCAACCTC  CCCCAGAAAA  GGAGAAATGG  3900
scaffold_22661      AAAAGACATC  ACCATACTCG  CCTTAAGGCA  GCTCAACCTC  CCCCAGAAAA  GGAGAAATGG  3900
scaffold_7076       AAAAGACATC  ACCATACTCG  CCTTAAGGCA  GCTCAACCTC  CCCCAGAAAA  GGAGAAATGG  3900
scaffold_22354      AAAAGACATC  ACCATACTCG  CCTTAAGGCA  GCTCAACCTC  CCCCAGAAAA  GGAGAAATGG  3900
scaffold_14383      AAAAGACATC  ACCATACTCG  CCTTAAGGCA  GCTCAACCTC  CCCCAGAAAA  GGAGAAATGG  3900

                                              3,920                                    3,940                                    3,960
                                                |                                        |                                        |
Ancestral_Gamma_G1  AAGGCAACAC  CCACAGAGGA  TCCACTCAAG  ATAAGACTCT  CGCGGGAATG  ATTATAATTG  3960
GeneScaffold_3363   AAGGCAACAC  CCACAGAGGA  TCCACTCAAG  ATAAGACTCT  CTCGGGAATA  ATTATAATTG  3960
scaffold_1753       AAGGCAACAC  CCACAGAGGA  TCCACTCAAG  ATAAGACTCT  CGCGGGAATA  ATTATAATTG  3960
scaffold_13050      AAGGCAACAC  CCACAGAGGA  TCCACTCAAG  ATAAGACTCT  CGCGGGAATG  ATTATAATTG  3960
scaffold_24606      AAGGCAACAC  CCACAGAGGA  TCCACTCAAG  ATAAGACTCT  CGCGGGAATG  ATTATAATTG  3960
scaffold_10119      AAGGCAACAC  CCACAGAGGA  TCCACTCAAG  ATAAGACTCT  CACGGGAATA  ATTATAATTG  3960
scaffold_23277      AAGGCAACAC  CCACAGAGGA  TCCACTCAAG  ATAAGACTCT  CGCGGGAATG  ATTATAATTG  3960
scaffold_12630      AAGGCAACAC  CCACAGAGGA  TCCACTCAAG  ATAAGACTCT  CGCGGGAATG  ATTATAATTG  3960
scaffold_2151       AAGGCAACAC  CCACAGAGGA  TCCACTCAAG  ATAAGACTCT  CGCGGGAATG  ATTATAATTG  3960
scaffold_7237       AAGGCAACAC  CCACAGAGGA  TCCACTCAAG  ATAAGACTCT  CGCGGGAATA  ATTATAATTG  3960
scaffold_12163      AAGGCAACAC  CCACAGAGGA  TCCACTCAAG  ATAAGATTCT  CGCGGGAATG  ATTATAATTG  3960
scaffold_941        AAAGCAACAC  CCACAGAGGA  TCCACTCAAG  ATAAGACTCT  CGCGGGAATA  ATTGTGTTAG  3960
scaffold_22661      AAAGCAACAC  CCACAGAGGA  TCCACTCAAG  ATAAGACTCT  CACGGGAATA  ATTGTGTTAG  3960
scaffold_7076       AAAGCAACAC  CCACAGAGGA  TCCACTCAAG  GATAGACTCT  CATGGGAATA  ATTGTGTTAG  3960
scaffold_22354      AAAGCAACAC  CCACAGAGGA  TCCACTCAAG  ATAAGACTCT  CGCGGGAATA  ATTGTGTTAG  3960
scaffold_14383      AAAGCAACAC  CCACAGAGGA  TCCACTCAAG  ATAAGACTCT  CGCGGGAATA  ATTGTGTTAG  3960

                                              3,980                                    4,000                                    4,020
                                                |                                        |                                        |
Ancestral_Gamma_G1  GGTTAATTGT  TATCACTAAT  GTAAAAGGGA  ACCCTCATCA  ACCTCAACAG  GCTAAATGGG  4020
GeneScaffold_3363   GGTTAATTGT  TATCAATAAT  GTAAAAGGGA  ACCCTCATCA  ACCTCAACAG  GCTAAATGGG  4020
scaffold_1753       GGTTAATTGT  TATCAATAAT  GTAAAAGGGA  ACCCTCATCA  ACCTCAACAG  GCTAAATGGG  4020
scaffold_13050      GGTTAATTGT  TATCAATAAT  GTAAAAGGGA  ACCCTCATCA  ACCTCAACAG  GCTAAATGGG  4020
scaffold_24606      GGTTAATTGT  TATCAATAAT  GTAAAAGGGA  ACCCTCATCA  ACCTCAACAG  GCTAAATGGG  4020
scaffold_10119      GGTTAATTGT  TATCAATAAT  GTAAAAGGGA  ACCCTCATCA  ACCTCAACAG  GCTAAATGGG  4020
scaffold_23277      GGTTAATTGT  TATCAATAAT  GTAAAAGGGA  ACCCTCATCA  ACCTCAACAG  GCTAAATGGG  4020
scaffold_12630      GGTTAATTGT  TATCAATAAT  GTAAAAGGGA  ACCCTCATCA  ACCTCAACAA  GCTAAATGGG  4020
scaffold_2151       GGTTAATTGT  TATCAATAAT  GTAAAAGGGA  ACCCTCATCA  ACCTCAACAG  GCTAAATGGG  4020
scaffold_7237       CGTTAATTGT  TATCAATAAT  GTAAAAGGGA  ACCCTCATCA  ACCTCAACAG  GCTAAATGGG  4020
scaffold_12163      GGTTAATTGT  TATCAATAAT  GTAAAAGGGA  ACCCTCATCA  ACCTCAACAG  GCTAAATGGG  4020
scaffold_941        GGTTAGTTGT  TATTACCAAT  GTACACGGGA  ACCCTCATCA  ACCTCAACAG  GCTCAATGGG  4020
scaffold_22661      GGTTAGTTGT  TATTACCAAT  GTACACGGGA  ACCCTCATCA  ACCTCAACAG  GCTCAATGGG  4020
scaffold_7076       GGTTAGTTGT  TATTACCAAT  GTACACGGGA  ACCCTCATCA  ACCTCAACAG  GCTCAATGGG  4020
scaffold_22354      GGTTAGTTGT  TATTACCAAT  GTACATGGGA  ACCCTCATCA  ACCTCAACAG  GCTCAATGGG  4020
scaffold_14383      GGTTAGTTGT  TATTACCAAT  GTACATGGGA  ACCCTCATCA  ACCTCAACAG  GCTCAATGGG  4020

                                              4,040                                    4,060                                    4,080
                                                |                                        |                                        |
Ancestral_Gamma_G1  AATTAATTAA  ATCAGAAACA  GGCATTCCCA  TATTAGTAAA  CCAGACTATG  TACCAGCCTA  4080
GeneScaffold_3363   AATTGATTAA  ATCAGAAACA  GACATCCCCA  TATTAGTAAA  CCAGACTATG  TACCAGCCTA  4080
scaffold_1753       AATTAATTAA  ATCAGAAACG  GGTATTCCCA  TATTAGTGAA  CCAGACTATG  TACCAGCCTA  4080
scaffold_13050      AATTAATTAA  ATCAGAAACA  GGCATTCCCA  TATTAGTAAA  CCAGACTATG  TACCAGCCTA  4080
scaffold_24606      AATTGATTAA  ATCAGAAACA  GGCATCCCCA  TATTAGTAAA  CCAGACTATG  TACCAGCCTA  4080
scaffold_10119      AATTAATTAA  ATCAGAAACA  GCCATCCCCA  TATTAGTAAA  CCAGACAATG  TACCAGCCTA  4080
scaffold_23277      AATTAATTAA  ATCAGAAATG  GGCATTCCCA  TATTAGTAAA  CCAGACTATG  TACCAGCCTA  4080
scaffold_12630      AATTAATTAA  ATCAGAAACA  GGCATTCCCA  TATTAGTAAA  TCAGACTATG  TACCAGCCTA  4080
scaffold_2151       AATTAATTAA  ATCAGAAACG  GGCATTCCCA  TATTAGTAAA  CCAGACTATG  TACCAGCCTA  4080
scaffold_7237       AATTAATTAA  ATCAGAAACA  GGCATCCCCA  TATTAGTAAA  CCAGACTATG  TACCAGCGTA  4080
scaffold_12163      AATTAATTAA  ATCAGAAACA  GGCATTCCCA  TATTAGTAAA  TCAGACTATG  TACCAGCCTA  4080
scaffold_941        AATTGATTAG  ATCAGAAACA  GGTATCTCCA  TATTAGTAAA  CCAGACTATG  TACCAGCCTA  4080
scaffold_22661      AATTGATTAA  ATCAGAAACA  GGTATCTCCA  TATTAGTAAA  CCAGACTATG  TACCAGCCTA  4080
scaffold_7076       AATTGATTAA  ATCAGAAACA  GGTATCTCCA  TATTAGTAAA  CCAGACTATG  TACCAGCCTA  4080
scaffold_22354      AATTGATTAA  ATCAGAAACA  GGTATCTCCA  TATTAGTAAA  CCAGACTATG  TACCAGCCTA  4080
scaffold_14383      AATTGATTAA  ATCAGAAACA  GGTATCTCCA  TATTAGTAAA  CCAGACTATG  TACCAGCCTA  4080
```

```
                                            4,100                          4,120                     4,140
                                                |                              |                         |
Ancestral_Gamma_G1  CATTTCATGT  GGATTTGTGT  GATATATTAG  GAAAGAAATT  TAATTTTCTA  AGTGATGATC  4140
  GeneScaffold_3363  CATTTCATGT  GGATTTGTGT  GATATATTAG  GAAAGAAATT  TAATTTTATA  AGTGATGATC  4140
      scaffold_1753  CATTTCATGT  AGATTTGTGT  GATATATTAG  GAAAGAAATT  TAATTTTATA  AGTGATGATC  4140
     scaffold_13050  CATTTCATGT  GGATTTGTGT  GATATATTAG  GAAAGAAATT  TAATTTTATA  AGTGATGATC  4140
     scaffold_24606  CATTTCATGT  GGATTTGTGT  GATATATTAG  GAAAGAAATT  TAATTTTATA  AGTAATGATC  4140
     scaffold_10119  CATTTCATGT  GGATTTGTAT  GATATATTAG  GAAAGAAATT  TAATTTTATA  AGTGATGATC  4140
     scaffold_23277  CATTTCATGT  GGATTTGTGT  GATATATTAG  GAAAGAAATT  TAATTTTATA  AGTGATGATC  4140
     scaffold_12630  CATTTCATGT  GGATTTGTGT  GATATATTAG  GAAAGAAATT  TAATTTTATA  AGTGAAGATC  4140
      scaffold_2151  CATTTCATGT  GGATTTGTGT  GACATATTAG  GAAAGAAATT  TAATTTTATA  AGTGATGATC  4140
      scaffold_7237  CATTTCATGT  GGATTTGTGT  GATATATTAG  GAAAGAAATT  TAATTTTATA  AGTGATGATC  4140
     scaffold_12163  CATTTCATGT  GGATTTGTGT  GATATATTAG  GAAAGAAATT  TAATTTTATA  AGTGATGATC  4140
       scaffold_941  CATTTCATGT  AGGTTTGTGT  AATATACTTG  GGGAGGAATT  AAATAAGCAA  ATAAAAGATC  4140
     scaffold_22661  CATTTCATGT  AGGTTTGTGT  AATATACTTG  GGGAGGAATT  AAATAAGCAA  ATAAAAGATC  4140
      scaffold_7076  CATTTCATGT  AGATTTGTGT  AATATACTTG  GGGAGGAATT  AAATAAGCAA  ACAAAAGATC  4140
     scaffold_22354  CATTTCATGT  AGATTAGTGT  AATATACTTG  GGGAGGAATT  AAATAAGCAA  ACAAAAGATC  4140
     scaffold_14383  CATTTCATGT  AGATTTGTGT  AATATACTTG  GGGAGGAACT  AAATAAGCAA  ACAAAAGATC  4140

                                            4,160                          4,180                     4,200
                                                |                              |                         |
Ancestral_Gamma_G1  TGGCCCCTGG  TGGGGTTGTG  GCAGGAGGAC  AAAGAAAGGG  GTATTCAACA  AGTACAGTTA  4200
  GeneScaffold_3363  TGGCCCCTGG  TATGGTTGTG  GCAGGAGGAC  AAAGAAAGAG  TTATTCAACA  AGTAGAGTTA  4200
      scaffold_1753  CGGCCCCTGG  TTTGGTTGTG  GCAAGAAGAC  AAAGAAAGGG  GCATCCAACA  AATACAGTTA  4200
     scaffold_13050  TGACCCCTGG  TATGGTTGTG  GCAGGAGGAC  AAAGAAAGAG  GTATTCAACA  AGTACAGTTA  4200
     scaffold_24606  TGGCCCCTGA  TATGGTTGTG  GCAGGAGGAC  AAAGAAAGAG  GTATTCAACA  AGTACAGTTA  4200
     scaffold_10119  TGGCCCCTGG  TATGGTTGTG  GCAGGAGGAC  AAAGAAAGAG  GTATTCAACA  AGTAGAGTTA  4200
     scaffold_23277  TGACCCCTGG  TATGGTTGTG  GCAGGAGGAC  AAAGAAAGAG  GTATTCAACA  AGTACAGTTA  4200
     scaffold_12630  TGGCCCCTGG  TATGGTTGTG  GCAGGAGGAC  AAAGAAAGAG  GTATTCAACA  AGTAGAGTTA  4200
      scaffold_2151  TGGCCCCTGG  TATGGTTGTG  GCAGGAGGAC  AAAGAAAGAG  GTATTCAACA  AGTACAGTTA  4200
      scaffold_7237  TGGCCCCTGG  TATGGTTGTG  GCAGGAGGAC  AAAGAAAGAG  GTATTCAACA  AGTACAGTTA  4200
     scaffold_12163  TGACCCCTGG  TATGGTTGTG  GCAGGAGGAC  AAAGAAAGAG  GTATTCAACA  AGTAGAGTTA  4200
       scaffold_941  AGGACCGTGG  TGGGGCTGTG  GAGTAAAAAT  TATGAAAAGG  GTATCCAACA  AGTGCAGTTA  4200
     scaffold_22661  AGGACCGTGG  TGGGGCTGTG  GAGTAAAAAT  TATGAAAAGG  GTATCCAACA  AGTGTAGTTA  4200
      scaffold_7076  AGGACCGTGG  TGGGGCTGTG  GAGTAAAAAT  TATGAAAAGG  GTATCCAACA  AGTGCAGTTA  4200
     scaffold_22354  AGGACCGTGG  TGGGGCTGTG  GAGTAAAAAT  TATGAAAAGG  GTATCCAACA  AGTGCAGTTA  4200
     scaffold_14383  AGGACCGTGG  TGGGGCTGTG  GAGTAAAAAT  TATGAAAAGG  GTATCCAACA  AGTGCAGTTA  4200

                                            4,220                          4,240                     4,260
                                                |                              |                         |
Ancestral_Gamma_G1  TATATGTGTC  CCAGAGAAGG  CCAAAGCACT  TGCCATAAGC  TAAACCAATA  TTATTGTGGT  4260
  GeneScaffold_3363  TATATGTGTC  CCAGAGAAGG  CCAAAGCACT  TGCCATAAGC  TAAACCAATA  TTATTGTGGT  4260
      scaffold_1753  TATATGTGTC  ACAGAGAAGG  CCAAAGCACT  TGCCATAAGC  TAAACCGATA  TTATTGTGGC  4260
     scaffold_13050  TATATGTGTC  CCAGAGAAGG  CCAAAGCACT  TGCCATAAGC  TAAACCAATA  TTATTGTGGC  4260
     scaffold_24606  TATATGTGTC  CCAGAGAAGG  CCAAAGCACT  TGCCATAAGC  TAAACCAATA  TTATTGTGGC  4260
     scaffold_10119  TATATGTGTC  CCAGAGAAGG  CCAAAGCACT  TGCCATAAGC  TAAACCAATA  TTATTGTGGT  4260
     scaffold_23277  TATATGTGTC  CCAGAGAAGG  CCAAAGCACT  TGCCATAAGC  TAAACCAATA  TTATTGTGGC  4260
     scaffold_12630  TATATGTGTC  CCAGAGAAGG  CCAAAGCACT  TGCCATAAGC  TAAACCAATA  TTATTGTGGC  4260
      scaffold_2151  TATATGTGTC  CCAGAGAAGG  CCAAAGCACT  TGCCATAAGC  TAAACCAATA  TTATTGTGGC  4260
      scaffold_7237  TATATGTGTC  CCAGAGAAGG  CCAAAGCACT  TGCCATAAGC  TAAACCAATA  TTATTGTGGC  4260
     scaffold_12163  TATATGTGTC  CCAGAGAAGG  CCAAAGCACT  TGCCATAAGC  TAAACCAATA  TTATTGTGGT  4260
       scaffold_941  TATATGTGTT  CTAGAGAAGA  TCAAAGCACT  TGCAATAAGC  CTAACCAATA  TTATTGTGGT  4260
     scaffold_22661  TATATGTGTC  CTAGAGAAGA  TCAAAGCACT  TGCAATAAGC  CTAACCAATA  TTATCGTGGT  4260
      scaffold_7076  TATATGTGTC  CTAGAGAAGA  TCAAAGCACT  TGCAATAAGC  CTAACCAATA  TTATTGTGGT  4260
     scaffold_22354  TATATGTGTC  CTAGAGAAGA  TCAAAGCACT  TGCAATAAGC  CTAACCAATA  TTATTGTGGT  4260
     scaffold_14383  TATATGTGTC  CTAGAGAAGA  TCAAAGCACT  TGCAATAAGC  CTAACCAATA  TTATTGTGGT  4260

                                            4,280                          4,300                     4,320
                                                |                              |                         |
Ancestral_Gamma_G1  CATTGGGGTT  GTGAGACCAT  AGCCCCTTGG  AAGAACACAG  ACCCCTTTCT  GACTCTTACA  4320
  GeneScaffold_3363  CATTGGGGTT  GTGAAACCAT  AGCCCCTTGG  AAGAACACAG  ACCCCTTTCT  GACTCTTACA  4320
      scaffold_1753  CATTGGGGTT  GTGAGACCAT  AGCCCCTTGG  GAGAACACAG  ACCCCTTTCT  GACTCTTACA  4320
     scaffold_13050  CATTGGGGTT  GTGAGACCAT  AGCCCCTTGG  AAGAACACAG  ACCCCTTTCT  GACTCTTACA  4320
     scaffold_24606  CATTGGGGTT  ATGAGACCAT  AGCCCCTTGG  GAGAACACAG  ACCCCTTTCT  GACTCTTACA  4320
     scaffold_10119  CATTGGGGTT  GTGAAACCAT  AGCCCCTTGG  AAGAACACAG  ACCCCTTTCT  GACTCTTACA  4320
     scaffold_23277  CATTGGGGTT  GTGAAACCAT  AGCCCCTTGG  AAGAACACAG  ACCCCTTTCT  GACTCTTACA  4320
     scaffold_12630  CATTGGGGTT  GTGAGACCAT  AGCCCCTTGG  GAGAACACAG  ACCCCTTTCT  GACTCTTACA  4320
      scaffold_2151  CATTGGGGTT  GTGAGACCAT  AGCCCCTTGG  GAGAACACAG  ACCCCTTTCT  GACTCTTACA  4320
      scaffold_7237  CATTGGGGTT  GTGAGACCAT  AGCCCCTTGG  GAGAACACAG  ACCCCTTTCT  GACTCTTACA  4320
     scaffold_12163  CATTGGGGTT  GTGAAACCAT  AGCCCCTTGG  AAGAACACAG  ACCCCTTTCT  GACTCTTACA  4320
       scaffold_941  CATTGGGGTT  GTGAGACCAT  AGCCCCTTGG  AAGAACACAG  ACCCCTTTCT  GACTCTTACA  4320
     scaffold_22661  CATTGGGGTT  GTGAGACCAT  AGCCCCTTGG  AAGAGCACAG  ACCCCTTTCT  GACTCTTACA  4320
      scaffold_7076  CATTGGGGTT  GTGAGACCAT  AGCCCCTTGG  AAGAGCACAG  ACCCCTTTCT  GACTCTTACA  4320
     scaffold_22354  CATTGGGGTT  GGGAGACCAT  AGCCCCTTGG  AAGAGCACAG  ACCCCTTTCT  GACTCTTACA  4320
     scaffold_14383  CATTGGGGTT  GTGAGACCAT  AGCCCCTTGG  AAGAGCACAG  ACCCCTTTCT  GACTCTTACA  4320
```

		4,340		4,360		4,380	
Ancestral_Gamma_G1	CGACCTAGTC	AGTCTTCCTG	TGCCACAGCA	GGGAAATGCA	ATCCTGTCAT	TTTTACTGTC	4380
GeneScaffold_3363	CGACCTAGTC	AGTCTTCCTG	TGCCACAGCA	GGGAAATGCA	ATCCTGTCAT	TTTTACTGTC	4380
scaffold_1753	CGACCTAGTC	AGTCTTCCTG	TGCCACAGCA	GGGAAATGCA	ATCCTGTCAT	TTTTACTGTC	4380
scaffold_13050	CGACCTAGTC	AGTCTTCCTG	TGCCACAACA	GGGAAATGCA	ATCCTGTCAT	TTTTACTGTC	4380
scaffold_24606	CGACCTAGTC	AGTCTTCCTG	TGCCACAGCA	GGGAAATGCA	ATCCTGTCAT	TTTTACTGTC	4380
scaffold_10119	CGACCTAGTC	AGTCCTCCTG	TGCCACAGCA	GGGAAATGCA	ATCCTATCAT	TTTTACTGTC	4380
scaffold_23277	CGACCTAGTC	AGTCTTCCTG	TGCCACAGCA	GGGAAATGCA	ATCCTGTCAT	TTTTACTGTC	4380
scaffold_12630	CGACCTAGTC	AGTCTTCCTG	TGCCACAGCA	GGGAAATACA	ATCCTGTCAT	TTTTACTGTC	4380
scaffold_2151	CGACCTAGTC	AGTCTTCCTG	TGCCACAGCA	GGGCAATGCA	ATCCTGTCAT	TTTTACTGTC	4380
scaffold_7237	CGACCTAGTC	AGTCTTCCTG	TGCCACAGCA	GGGAAATGCA	ATCCTGTCAT	TTTTACTGTC	4380
scaffold_12163	CGACCTAGTC	AGTCTTCCTG	TGCCACAGCA	GGGAAATGCA	ATCCTGTCAT	TTTTACTGTC	4380
scaffold_941	CGACCTAGTC	AGTCTTCCTG	TGCCACAGCA	GGGAAATGCA	ATCCTGTCGT	TTTTACTGTC	4380
scaffold_22661	CGACCTAGTC	AGTCTTCCTG	TGCCACAGCA	GGGAAATGCA	ATCCTGTCGT	TTTTACTGTC	4380
scaffold_7076	CGACCTAGTC	AGTCTTCCTG	TGCCACAGCA	GGGAAATGCA	ATCCTGTCGT	TTTTACTGTC	4380
scaffold_22354	CGACCTAGTC	AGTCTTCCTG	TGCCACAGCA	GGGAAATGCA	ATCCTGTCGT	TTTTACTGTC	4380
scaffold_14383	CGACCTAGTC	AGTCTTCCTG	TGCCACAGCA	GGGAAATGCA	ATCCTGTCGT	TTTTACTGTC	4380

		4,400		4,420		4,440	
Ancestral_Gamma_G1	AAAAATTGGG	AAGATCCATC	TTGGGTGATT	GGAAAAACCT	GGGGATTAAG	GTTGTATGTT	4440
GeneScaffold_3363	AAAAATTGGA	AAGATCCATC	TTGGGTGATT	GGAAAAACCT	GGGGATTAAG	GTTGTATGTT	4440
scaffold_1753	AAAAATTGGG	AAGATCCATC	TTGGGTGATT	GGAAAAACCT	GGGGATTAAG	GTTGTATGTT	4440
scaffold_13050	AAAAATTGGG	AAGATCCATC	TTGGGTGATT	GGAAAAACCT	GGGGATTAAG	GTTGTATGTT	4440
scaffold_24606	AAAAATTGAG	AAGATCCATC	TTGGGTGATT	GGAAAAACCT	GGGGATTAAG	GTTGTATGTT	4440
scaffold_10119	AAAAATTGGG	AAGATCCATC	TTGGGTGATT	GGAAAAACCT	GGGGATTAAG	GTTGTATGTT	4440
scaffold_23277	AAAAATTGGG	AAGATCCATC	TTGGGTGATT	GGAAAAACCT	GGGGATTAAG	GTTGTATGTT	4440
scaffold_12630	AAAAATTGGG	AAGATCCATC	TTGGGTGATT	GGAAAAACCT	GAGGATTAAG	GTTGTATGTT	4440
scaffold_2151	AAAAATTGGG	AAGATCCATC	TTGGGTGATT	GGAAAAACCT	AGGGATTAAG	GTTGTATGTT	4440
scaffold_7237	AAAAATTGGG	AAGATCCATC	TTGGGTGATT	GGAAAAACCT	GGGGATTAAG	GTTGTATGTT	4440
scaffold_12163	AAAAATTGGA	AAGATCCATC	TTGGGTGATT	GGAAAAACCT	GGGGATTAAG	GTTGTATGTT	4440
scaffold_941	AAAAATTGGG	AAGATCCATC	TTGGGTGACT	GGGAAAACTT	GGGGATTAAG	GTTGTATGTT	4440
scaffold_22661	AAAAATTGGG	AAGATCCATC	TTGGGTGACT	GGGAAAACTT	GAGGATTAAG	GTTGTATGTT	4440
scaffold_7076	AAAAATTGGG	AAGATCCATC	TTGGGGGACT	GGGAAAACTT	GAGGATTAAG	GTTGTATGTT	4440
scaffold_22354	AAAAATTGGG	AAGATCCATC	TTGGGTGACT	GGGAAAACTT	GGGGATTAAG	GTTGTATGTT	4440
scaffold_14383	AAAAATTGGG	AAGATCCATC	TTGGGTGACT	GGGAAAACTT	GAGGATTAAG	GTTGTATGTT	4440

		4,460		4,480		4,500	
Ancestral_Gamma_G1	TTTGGAACTG	ATCCTGGAGT	GTTAATAACA	ATACAGAAAA	AGCCTGTCAG	GATGCCACCG	4500
GeneScaffold_3363	TTTGGAACTG	ATCCTGGAGT	GTTAATAACA	ATACAGAAAA	AGCCTGTCAG	GATGCCACCA	4500
scaffold_1753	TTTGGAACTG	ATCCTGGAGT	GTTAATAACA	ATACAGAAAA	AGCCTGTCAG	GATGCCACCG	4500
scaffold_13050	TTTGGAACTG	ATCCTGGAGG	GTTAACAACA	ATACAGAAAA	AGCCTGTCAG	GATGCCACCG	4500
scaffold_24606	TTTGGAACTG	ATCCTGGAGT	GTTAATAACA	ATACAGAAAA	AGCCTGTCAG	GATTCCACCG	4500
scaffold_10119	TTTGGAACTG	ATCCTGGAGT	GTTAATAACA	ATACAGAAAA	AGCCTGTCAG	GATGCCACCA	4500
scaffold_23277	TTTGGAACTG	ATCCTGGAGT	GTTAATAACA	ATACAGAAAA	AGCCTGTCAG	GATGCCACCG	4500
scaffold_12630	TTTGGAACTG	ATCCTGGAGT	GTTAATAACA	ATACAGAAAA	AGCCTGTCAG	GATGCCACCG	4500
scaffold_2151	TTTGGAACTG	ATCCTGGAGT	GTTAATAACA	ATACAGGAAA	AGCCTGTCAG	GATGCCACCG	4500
scaffold_7237	TTTGGAACTG	ATCCTGGAGT	GTTAATAACA	ATACAGAAAA	AGCCTGTCAG	GATGCCACCA	4500
scaffold_12163	TTTGGAACTG	ATCCTGGAGT	GTTAATAACA	ATACAGAAAA	AGCCTGTCAG	GATGCCACCG	4500
scaffold_941	TCTGGAGATG	ATCCCGGAAT	GTTAATGACA	ATACAGAAAA	AGCCTGTTCG	GATACCGTCG	4500
scaffold_22661	TCTGGAGATG	ATCCCGGAAT	GTTAATGACA	ATACAAAAAA	AGCCTGTCCG	GATACCGTCG	4500
scaffold_7076	TCTGGAGATG	ATCCCGGAAT	GTTAATGACA	ATACAAAAAA	AGCCTGTCCG	GATACCGTCG	4500
scaffold_22354	TCTGGAGATG	ATCCCGGAAT	GTTAATGACA	ATACAAAAAA	AGCCTGTCCG	GATACCGTCG	4500
scaffold_14383	TCTGGAGATG	ATCCCGGAAT	GTTAATGATA	ATACAAAAAA	AGCCTGTCCG	GATACCGTCG	4500

		4,520		4,540		4,560	
Ancestral_Gamma_G1	TTAGTTCTAG	TCCTGTTAGG	CCTAATGACG	GAGGGTTAAA	CGCCTTAACT	CCTACCCAGT	4560
GeneScaffold_3363	TTAGTTCTAG	TCCTGTTAGG	CCTAATGACG	GAGGGTTAAA	CGCCTTAACT	CCTACACAGT	4560
scaffold_1753	TTAGTTCTAG	TCCTGTTAGG	CCTAATGACG	GAGGGTTAAA	CGCCTTAACT	CCTACACAGT	4560
scaffold_13050	TTAGTTCTAG	TCCTGTTAGG	CCTAATGACG	GAGGGTTAAA	CGCCTTAACT	CCTACACAGT	4560
scaffold_24606	TTAGTTCTAG	TCCTGTTAGG	CCTAATGACG	GAGGGTTAAA	CGCCTTAACT	CCTACACAGT	4560
scaffold_10119	TTAGTTCTAG	TCCTGTTAGG	CCTAATGACG	GAGGGTTAAA	CGCCTTAACT	CCTACACAGT	4560
scaffold_23277	TTAGTTCTAG	TCCTGTTAGG	CCTAATGACG	GAGGGTTAAA	CGCCTTAACT	CCTACACAGT	4560
scaffold_12630	TTAGTTCTAG	TCCTGTTAGG	CCTAATGACG	GAGGGTTAAA	CACCTTAACT	CCTACACAGT	4560
scaffold_2151	TTAGTTCTAG	TCCTGTTAGG	CCTAATGACG	GAGGGTTAAG	CGCCTTAACT	CCTACACAGT	4560
scaffold_7237	TTAGTTCTAG	TCCTGTTAGG	CCTAATGACG	GAGGGTTAAA	CGCCTTAACT	CCTACACAGT	4560
scaffold_12163	TTAGTTCTAG	TCCTGTTAGG	CCTAATGACG	GAGGGTTAAA	CGCCTTAACT	CCTACACAGT	4560
scaffold_941	ATAGCCATAG	TCCTGTCGGG	TCTGATAATG	AAGGGTTAAA	GGACTTAACT	CCTACCCAGT	4560
scaffold_22661	ATAGCCATAG	TCCTGTCGGG	TCTAATAATG	AAGGGTTAAA	GGACTTAACT	CCTATCCAGT	4560
scaffold_7076	ATAGCCATAG	TCCTGTCGGG	TCTAATAATG	AAGGGTTAAA	GGACTTAACT	CCTACCCAGT	4560
scaffold_22354	ATAGCCATAG	TCCTGTCGGG	TCTAATAATG	AAGGGTTAAA	GGACTTAACT	CCTACCCAGT	4560
scaffold_14383	ATAGCCATAG	TCCTGTCGGG	TCTAATAATG	AAGGGTTAAA	GGACTTAACT	CCTACCCAGT	4560

```
                                              4,580                          4,600                        4,620
                                                  |                              |                            |
Ancestral_Gamma_G1  CAGCAATTAC  TAGGAGCACT  AATAATGCCT  CTTAATTCTT  TAGCTGAAGT  AGTACTGCAG  4620
 GeneScaffold_3363  CAGCAATTAC  TAGGAGCACT  AATAATGCCT  CTTAATTCTT  TAGCTGAAGT  AGTACTGCAG  4620
     scaffold_1753  CAGCAATTAC  TAGGAGCACT  AATAATGCCT  CTTAATTCTT  TAGCTGAAGT  AGTACTGCAG  4620
    scaffold_13050  CAGCAATTAC  TAGGAGCACT  AATAATGCCT  CTTAATTCTT  TAGCTGAAGT  AGTACTGCAG  4620
    scaffold_24606  CAGCAATTAC  TAGGAGCACT  AATAATGCCT  CTTAATTCTT  TAGCTGAAGT  AGTACTGCAG  4620
    scaffold_10119  CAGCAATTAC  TAGGAGCACT  AATAATGCCT  CTTAATTCTT  TAGCTGAAGT  AGTACTACAA  4620
    scaffold_23277  CAGCAATTAC  TAGGAGCACT  AATAATGCCT  CTTAATTCTT  TAGCTGAAGT  AGTACTGCAG  4620
    scaffold_12630  CAGCAATTAC  TAGGAGCACT  AATAATGCCT  CTTAATTCTT  TAGCTGAAGT  AGTACTGCAG  4620
     scaffold_2151  CAGCAATTAC  TAGGAGCACT  AATAATGCCT  CTTAATTCTT  TAGCTGAAGT  AGTACAGCAG  4620
     scaffold_7237  CAGCAATTAC  TAGGAGCACT  AATAATGCCT  CTTAATTCTT  TAGCTGAAGT  AGTACTGCAG  4620
    scaffold_12163  CAGCAATTAC  TAGGAGCACT  AATAATGCCT  CTTAATTCTT  TAGCTGAAGT  AGTACTGCAG  4620
      scaffold_941  TAACAACTAT  TAGGAATACT  AATAACATCT  CTTAATTCCT  TAGCTGAAGT  AGTACTGCAG  4620
    scaffold_22661  TAACAACTAT  TAGGAATACT  AATAACATCT  CTTAATTCCT  TAGCTGAAGT  AGTACTGCAG  4620
     scaffold_7076  TAACAACTAT  TAGGAATACT  AATAACATCT  CTTAATTCCT  TAGCTGAAGT  AGTACTGCAG  4620
    scaffold_22354  TAACAACTAT  TAGGAATACT  AATAACATCT  CTTAATTCCT  TAGCTGAAGT  AGTACTGCAG  4620
    scaffold_14383  TAACAACTAT  TAGGAATACT  AATAACATCT  CTTAATTCCT  TAGCTGAAGT  AGTACTACAG  4620

                                              4,640                          4,660                        4,680
                                                  |                              |                            |
Ancestral_Gamma_G1  AATAGAAGAG  GTTTAGACCT  CTTATTTCTT  AAGCAAGGAG  GTTTGTGTGT  AGCACTAGGA  4680
 GeneScaffold_3363  AATAGAAGAG  GTTTAAACCT  CCTATTTCTT  AAGCTAGGAG  GTTTGTGTGT  AGCACTAGGA  4680
     scaffold_1753  AATAGAAGAG  GTTTAGACCT  CTTATTTCTT  AAGCAAGGAG  GTTTGTGTGT  AGCACTAGGA  4680
    scaffold_13050  AATAGAAGAG  GTTTAGACCT  CTTATTTCTT  AAGCAAGGAG  GTTTGTGTGT  AGCACTAGGA  4680
    scaffold_24606  AATAGAAGAG  GTTTAGACCT  CTTATTTCTT  AAGCAAGGAG  GTTTGTGTGT  AGCACTAGGA  4680
    scaffold_10119  AATAGAAGAG  GTTTAGACCT  CTTATTTCTT  AAGCAAGGAG  GTTTGTGTGT  AACACTAGGA  4680
    scaffold_23277  AATAGAAGAG  GTTTAGACCT  CTTATTTGTT  AAGCAAGGAG  GTTTGTGTGT  AGCACTAGGA  4680
    scaffold_12630  AATAGAAGAG  GTTTAGACCT  CTTATTTCTT  AAGCAAGGAG  GTTTGTGTGT  AGCACTAGGA  4680
     scaffold_2151  AATAGAAGAG  GTTTAGACCT  CTTATTTCTT  AAGCAAGGAG  GTTTGTGTGT  AGCACTAGGA  4680
     scaffold_7237  AATAGAAGAG  GTTTAGACCT  CTTATTTCTT  AAGCAAGGAG  GTTTGTGTGT  AGCACTAGGA  4680
    scaffold_12163  AATAGAAGAG  GTTTAGACCT  CTTATTTCTT  AAGCAAGGAG  TTTTGTATGC  AGCACTAGGA  4680
      scaffold_941  AATAGGAGAG  GTTTAGATCT  CTTATTTCTT  AAGCAAGGAG  GTTTGTGTGT  AGCATTAGGA  4680
    scaffold_22661  AATAGGAGAG  GTTTAGATCT  CTTATTTCTT  AAGCAAGGAG  GTTTGTGTGT  AGCATTAGGA  4680
     scaffold_7076  AATAGGAGAG  GTTTAGATCT  CTTATTTCTT  AAGCAAGGAG  GTTTGTGTGT  AGCATTAGGA  4680
    scaffold_22354  AATAGGAGAG  GTTTAGATCT  CTTATTTCTT  AAGCAAGGAG  GTTTGTGTGT  AGCATTAGGA  4680
    scaffold_14383  AATAGGAGAG  GTTTAGATCT  CTTATTTCTT  AAGCAAGGAG  GTTTGTGTGT  AGCATTAGGA  4680

                                              4,700                          4,720                        4,740
                                                  |                              |                            |
Ancestral_Gamma_G1  GAAACTTGCT  GTTTTTATGT  TAATCATTCT  GGAATTATTA  GGGATAGTTT  AGCATTAATT  4740
 GeneScaffold_3363  GAAACTTGCT  GCTTTTATGT  TAATCATTCT  GGAATTATTA  GGGATAGTTT  AGCATTAATT  4740
     scaffold_1753  GAAACTTGCT  GCTTTTATGT  TAATCATTCT  GGTATTATTA  GGGATAGTTT  AGCATTAATT  4740
    scaffold_13050  GAAACTTGCT  GTTTTTATGT  TAATCATTCT  GGAATTATTA  GGGATAGTTT  AGCATTAATT  4740
    scaffold_24606  GAAACTTGCT  GTTTTTATGT  TAATCATTCT  GGAATTATTA  GGGATAGTTT  AGCATTAATT  4740
    scaffold_10119  GAAACTTGCT  GTTTTTATGT  TAATCATTCT  GGAATTATTA  GGGATAGTTT  AGCATTAATT  4740
    scaffold_23277  GAAACTTGCT  GTTTTTATGT  TAATCATTCT  GGAATTATTA  GGGATAGTTT  AGCATTAATT  4740
    scaffold_12630  GAAACGTGCT  GTTTTTATGT  TAATCATTCT  GGAATTATTA  GGGATAGTTT  AGCATTAATT  4740
     scaffold_2151  GAAACTTGCT  GTTTTTATGT  TAATCATTCT  GGAATTATTA  GGGATAGTTT  AGCATTAATT  4740
     scaffold_7237  GAAACTTGCT  GTTTTTATGT  TAATCATTCT  GGAATTATTA  GGGATAGTTT  AGCATTAATT  4740
    scaffold_12163  GAAACTTGCT  GTTTTTATGT  TAATCATTCT  GGAATTATTA  GGGATAGTTT  AGCATTAATT  4740
      scaffold_941  GAAACTTGCT  GTTTTTTTGT  TAATCATTCT  GGTATTATTA  GGGATAGTTT  AGCATTAATT  4740
    scaffold_22661  GAAACTTGCT  GTTTTTATGT  TAATCATTCT  GGTATTATTA  GGGATAGTTT  AGCATTAATT  4740
     scaffold_7076  GAAACTTGCT  GTTTCTATGT  TAATCATTCT  GGTATTATTA  GGGATAGTTT  AGCATTAATT  4740
    scaffold_22354  GAAACTTGCT  GTTTTTATGT  TAATCATTCT  GGTATTATTA  GGGATAGGTT  AGCATTAATT  4740
    scaffold_14383  GAAACTTGCT  GTTTTTATGT  TAATCATTCT  GGTATTATTA  GGGATAGTTT  AGCATTAATT  4740

                                              4,760                          4,780                        4,800
                                                  |                              |                            |
Ancestral_Gamma_G1  AGACAAAGGG  TAAAAGACAG  AAATGAGAAG  TTAAAACAAG  GCAGCAATGG  TATGAATCTT  4800
 GeneScaffold_3363  AGACAAAGAG  TAAAAGACAG  AAATAAGAAG  TTAAAACAAG  GAAGCAATGG  TATGAATCTT  4800
     scaffold_1753  AGACGAAGAG  TAAAAGACAG  AAATGAGAAG  TTAAAACAAG  GAAGCAATGG  TATGAATCTT  4800
    scaffold_13050  AGACAAAGAG  TAAAAGACAG  AAATGAGAAG  TTAAAACAAG  GAAGCAATGG  TATGAATCTT  4800
    scaffold_24606  AGACAAAGAG  TAAAAGACAG  AAATGAGAAG  TTAAAACAAG  GAAGCAATGG  TATGAATCTT  4800
    scaffold_10119  AGACAAAGAG  TAAAAGACAA  AAATGAGAAG  TTAAAACAAG  GAAGCAATGG  TATGAATCTT  4800
    scaffold_23277  AGACAAAGAG  TAAAAGACAG  AAATGAGAAG  TTAAAACAAG  GAAGCAATGG  TATGAATCTT  4800
    scaffold_12630  AGACAAAGAG  TAAAAGACAA  AAATGAGAAG  TTAAAACAAG  GAAACAATGG  TATGAATCTT  4800
     scaffold_2151  AGACAAAGAG  TAAAAGACAG  AAATGAGAAG  TTAAAACAAG  GAAGCAATGG  TATGAATCTT  4800
     scaffold_7237  AGACAAAGAG  TAAAAGACAG  AAATGAGAAG  TTAAAACAAG  GAAGCAATGG  TATAAATCTT  4800
    scaffold_12163  AGACAAAGAG  TAAAAGACAG  AAATGAGAAG  TTAAAACAAG  GAAGCAATGG  TATGAATCTT  4800
      scaffold_941  AGACAGAGGG  TGAAGGATAG  AAATGAAAAA  TTAAGGCAGG  GCAGTAATGG  TATGAGTCTT  4800
    scaffold_22661  AGACAGAGGG  TGAAGGATAG  AAATAAAAAA  TTAAGGCAGG  GCAATAATGG  TATGAGTCTT  4800
     scaffold_7076  AGACAGAGGG  TGAAGGATAG  AAATAAAAAA  TTAAGGCAGG  GCAGTAATGG  TATGAGTCTT  4800
    scaffold_22354  AGACAGAGGG  TGAAGGATAG  AAATAAAAAA  TTAAGGCAGG  GCAGTAATGG  TATGAGTCTT  4800
    scaffold_14383  AGACAGAGGG  TGAAGGATAG  AAATAAAAAA  TTAAGGCAGG  GCAGTAATGG  TATGAGTCTT  4800
```

```
                                              4,820                            4,840                           4,860
                                                |                                |                               |
Ancestral_Gamma_G1  GGTTTAATGG  AAACTCTTGG  TTAACCACGT  TATTGTCGGC  TATAGCAGGA  CCATTGATTA  4860
 GeneScaffold_3363  GGTTTAATGG  AAACTCTTGG  TTAACCACGT  TATTGTCGGC  TATAGCAGGA  CCATTGATAA  4860
     scaffold_1753  GGTTTAATGG  AAACTCTTGG  TTAACCACGT  TATTGTCGGC  TATAGCAGGA  CCATTGATAA  4860
    scaffold_13050  GGTTTAATGG  AAACTCTTGG  TTAACCACGT  TATTGTCGGC  TATAGCAGGA  CCATTGATAA  4860
    scaffold_24606  GGTTTAATAA  AAACTCTTGA  TTAACCACGT  TATTGTCGGC  TATAGCAGGA  CCATTGATAA  4860
    scaffold_10119  GGTTTAATGG  AAACTCTTGG  TTAACCACGT  TATTGTCGGC  TATAGCAGGA  CCATTGATAA  4860
    scaffold_23277  GGTTTAATGG  AAACTCTTGG  TTAACCACGT  TATTGTCGGC  TATAGCAGGA  CCATTGATAA  4860
    scaffold_12630  GGTTTAATGG  AAACTCTTGG  TTAACCACGT  TATTGTCGGC  TATAGCAGGA  CCATTGATAA  4860
     scaffold_2151  GATTTAATGG  AAACTCTTGG  TTAACCACGT  TATTGTCGGC  TATAGCAGGA  CCATTGATAA  4860
     scaffold_7237  GGTTTAATGG  AAACTCTTGG  TTAACCACGT  TATTGTCGGC  TATAGCAGGA  CCATTGATAA  4860
    scaffold_12163  GGTTTAATAG  AAACTCTTGG  TTAACCACGT  TATTGTCGGC  TATAGCAGGA  CCATTGATAA  4860
      scaffold_941  GGTTTAATGA  AAATTCCTGG  CTAACCATGC  TGTTGTCAGC  CATAGCAGGG  CCATTAATTA  4860
    scaffold_22661  GGTTTAATGA  AAATTCCTGG  CTAACCACGC  TATTGTCAGC  CATAGCAGGG  CCATTAATTA  4860
     scaffold_7076  GGTTTAATGA  AAATTCCTGG  CTAACCACGC  TATTGTCAGC  CATAGCAGGG  CCATTAATTA  4860
    scaffold_22354  GATTTAATGA  AAATTCCTGG  CTAACCACGC  TATTGTCAGC  CATAGCAGGG  CCATTAATTA  4860
    scaffold_14383  GGTTTAATGA  AAATTCCTGG  CTAACCACGC  TATTGTCAGC  CATAGCAGGG  CCATTAATTA  4860

                                              4,880                            4,900                           4,920
                                                |                                |                               |
Ancestral_Gamma_G1  TACTGCTGTT  ATTACCTTTG  GGCCCTGCTT  GATCAAATGG  GTTAGGAGTT  TAATCCAATA  4920
 GeneScaffold_3363  TTCTGCTGTT  ATTACCTTTG  GGCCATGCTT  GATCAAATGG  GTTAAGAGTT  TAATCCAATA  4920
     scaffold_1753  TTCTGCTGTT  ATTACCTTTG  GGCCATGCTT  GAGCAAATGG  GTTAAGAGTT  TAATCCAATA  4920
    scaffold_13050  TTCTGCTGTT  ATTACCTTTA  GGCCATGCTT  GATCAAATGG  GTTAAGAGTT  TAATCCAATA  4920
    scaffold_24606  TTCTGCTGTT  ATTACCTTTG  GGCCATGCTT  GATCAAATGG  GTTAAGAGTT  TAATCCAATA  4920
    scaffold_10119  TTCTGCGGTT  ATTACCTTTG  GGCCATGCTT  GATCAAATGG  GTTAAGAGTT  TAATCCAATA  4920
    scaffold_23277  TTCTGCTGTT  ATTACCTTTG  GGCCATGCTT  GATCAAATGG  GTTAAGAGTT  TAATCCAATA  4920
    scaffold_12630  TTCTGCTGTT  ATTACCTTTG  GGCCATGCTT  GATCAAATGG  ATTAAGAGTT  TAATCCAATA  4920
     scaffold_2151  TTCTGCTGTT  ATTACCTTTG  GGCCATGCTT  GATCAAATGG  GTTAAGAGTT  TAATCCAATA  4920
     scaffold_7237  TTCTGCTGTT  ATTACCTTTG  GGCCATGCTT  GATCAAATGG  GTTAAGAGTT  TAATCCAATA  4920
    scaffold_12163  TACTGCTGTT  ATTACCTTTG  GGCCATGCTT  GATCAAATGG  GTTAAGAGTT  TAATCCAATA  4920
      scaffold_941  TGCTGATATT  ATTATTTTTG  GACCCTGCTT  ACTAAAGTGG  CTTAGAAGTT  TAGTCTCACA  4920
    scaffold_22661  TGCTGATATT  ATTATTTTTG  GACCCTGCTT  ACTAAAGTGG  CTTAGAAGTT  TAGTCTCACA  4920
     scaffold_7076  TGCTGATATT  ATTATTTTTG  GACCCTGCTT  AATAAAGTGG  CTTAGAAGTT  TAGTCTCACA  4920
    scaffold_22354  TGCTGATATT  ATTATTTTTG  GACCCTGCTT  ACTAAAGTGG  CTTAGAAGTT  TAGTCTCACA  4920
    scaffold_14383  TGCTGATATT  ATTATTTTTG  GACTCTGCTT  ACTAAAGTGG  CTTAGAAGTT  TAGTCTCACA  4920

                                              4,940                            4,960                           4,980
                                                |                                |                               |
Ancestral_Gamma_G1  TAACTGGGGC  AAAGATTTTA  ATTTTAGGAG  CGTCCCGACC  CATTGGCCGG  AAATCCCGAC  4980
 GeneScaffold_3363  TAACTGGGGC  AAAGATTTTA  ATTTTAGGAG  CGTCCCGACC  CATTGGCCAG  AAATCCCGAC  4980
     scaffold_1753  TAACTGGGGC  AAAGATTTTA  ATTTTAGGAG  CGTCCCGACC  CATTGGCCAG  AAATCCCGAC  4980
    scaffold_13050  TAACTGGGGC  AAAGATTTTA  ATTTTAGGAG  CGTCCCGACC  CATTGGCCAG  AAATCCCGAC  4980
    scaffold_24606  TAACTGGGGC  AAAGATTTTA  ATTTTAGGAG  CGTCCCGACC  CATTGGCCAG  AAATCCCGAC  4980
    scaffold_10119  TAACTGGGGC  AAAGATTTTA  ATTTTAGGAG  CGTCCCGACC  CATTGGCCAG  AAATCCCGAC  4980
    scaffold_23277  TAACTGGGGC  AAAGATTTTA  ATTTTAGGAG  CGTCCCGACC  CATTGGCCAG  AAATCCCGAC  4980
    scaffold_12630  TAACTGGGGC  AAAGATTTTA  ATTTTAGGAG  CGTCCCGACC  CATTGGCCAG  AAATCCCGAC  4980
     scaffold_2151  TAACTGGGGC  AAAGATTTTA  ATTTTAGGAG  CGTCCCGACC  CATTGGCCAG  AAATCCCGAC  4980
     scaffold_7237  TAACTGGGGC  AAAGATTTTA  ATTTTAGGAG  CGTCCCGACC  CATTAGCCAG  AAATCCCGAC  4980
    scaffold_12163  TAACTGGGGC  AAAGATTTTA  ATTGTAGGAG  CGTCCCGACC  CATTGGCCAG  AAATCCCGAC  4980
      scaffold_941  TGACCGGGGC  AAAGATTTTA  ATTTTAGGAG  CATCCCGACG  AGTTGGCCGA  GAATTCCAAC  4980
    scaffold_22661  TGACCGGGAC  AAAGATTTTA  ATTTTAGGAG  CATCCCGACG  AGTTGGCCGA  GAATTCCAAC  4980
     scaffold_7076  TGACCGGGGC  AAAGATTTTA  ATTTTAGGAG  CATCCCGACG  AGTTGGCCGA  GAGTTCCAAC  4980
    scaffold_22354  TGACCGGGGC  AAAGATTTTA  ATTTTAGGAG  CATCCCGACG  AGTTGGCTGA  GAATTCCAAC  4980
    scaffold_14383  TGACCGGGGC  AAAGATTTTA  ATTTTAGGAG  CATCCCGACG  AGTTGGCCGA  GAGTTCCAAC  4980

                                              5,000                            5,020
                                                |                                |
Ancestral_Gamma_G1  TTCTGACTCG  AAGATTGAGT  CATTCTAAAG  AAAACCAGTG  GGGAA  5025
 GeneScaffold_3363  TTCTGACTCG  AAGATTGAGT  CATTCTAAAG  AAAACCAGTG  GGGAA  5025
     scaffold_1753  TTCTGACTCG  AAGATTGAGT  CATTCTAAAG  AAAACCAGTG  GGGAA  5025
    scaffold_13050  TTCTGACTCG  AAGATTGAGT  CATTCTAAAG  AAAACCAGTG  GGGAA  5025
    scaffold_24606  TTCTGACTCG  AAGATTGAGT  CATTCTAAAG  AAAACCAGTG  GGGAA  5025
    scaffold_10119  TTCTGACTCG  AAGATTGAGT  CATTCTAAAG  AAAACCAGTG  GGGAA  5025
    scaffold_23277  TTCTGACTCG  AAGATTGAGT  CATTCTAAAG  AAAACCAGTG  GGGAA  5025
    scaffold_12630  TTCTGACTCG  AAGATTGAGT  CATTCTAAAG  AAAACCAGTG  GGGAA  5025
     scaffold_2151  TTCTGACTCG  AAGATTGAGT  CATTCTAAAA  AAAACCAGTG  GGGAA  5025
     scaffold_7237  TTCTGACTCG  AAGATTGAGT  CATTCTAAAG  AAAACCAGTG  GGGAA  5025
    scaffold_12163  TTCTGACTCG  AAGATTGAGT  CATTCTAAAG  AAAACCAGTG  GGGAA  5025
      scaffold_941  TTCTGACTCG  AAGATTGAGT  CACTGTAAAG  AAAACCAGTG  GGGAA  5025
    scaffold_22661  TTCTGACTCG  AAGATTGAGT  CACTGTAAAG  AAAACCAGTG  GGGAA  5025
     scaffold_7076  TTCTGACTCG  AAGATTGAGT  CACTGTAAAG  AAAACCAGTG  GGGAA  5025
    scaffold_22354  TTCTGACTCG  AAGATTGAGT  CACTGTAAAG  AAAACCAGTG  GGGAA  5025
    scaffold_14383  TTCTGACTCG  AAGATTGAGT  CACTGTAAAG  AAAACCAGTG  GGGAA  5025
```
