# Supplementary material for: Differential Evolution of Antiretroviral Restriction Factors in Pteropid Bats as Revealed by APOBEC3 Gene Complexity
Source: Mol Biol Evol. 2018 Mar 29;35(7):1626–37. doi: 10.1093/molbev/msy048 (PMC5995163; doi:10.1093/molbev/msy048)
Supplement: Supplementary Data [file msy048_supp.zip › Supplementary Figures.docx]

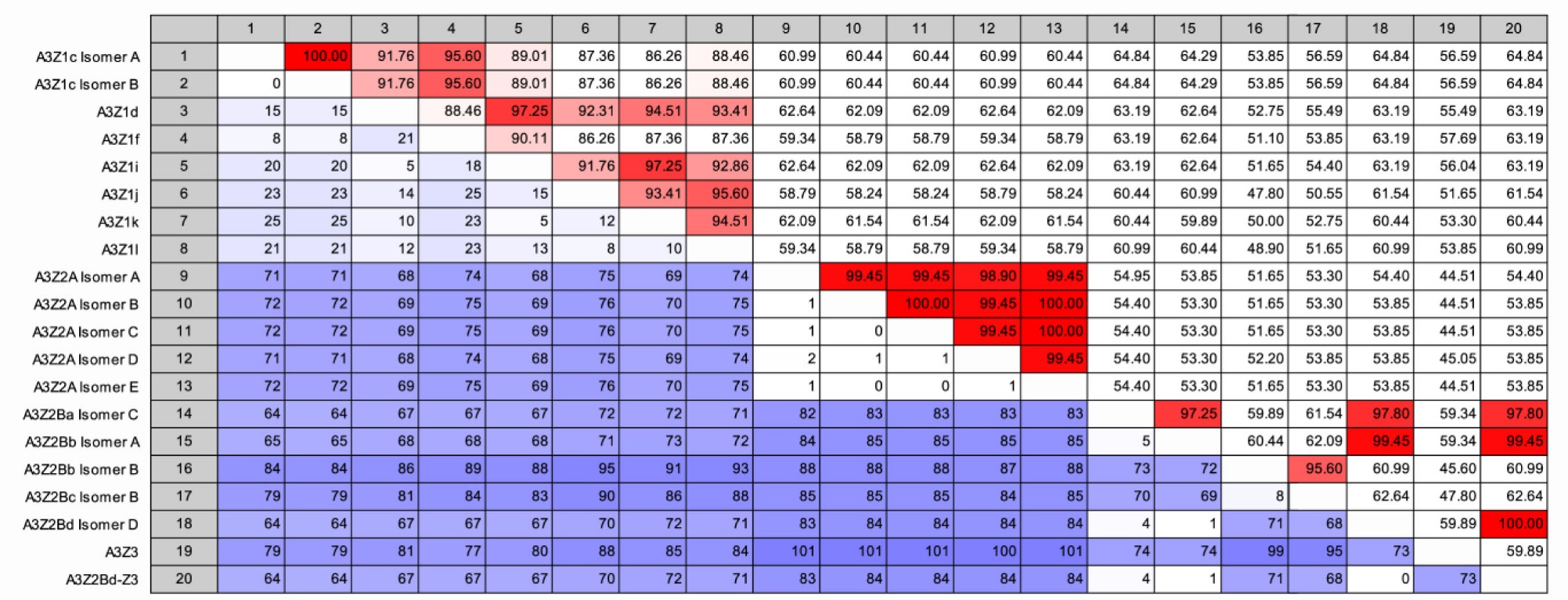


Fig. S1. Pairwise comparisons of the *A3* gene products identified in the *Pteropus alecto* cDNA library. Values above the diagonal represent nucleotide percentage identity between sequence pairs, colored with a white to red gradient indicating increasing similarity as red intensity increases. Values below the diagonal represent the number of nucleotide differences between sequence pairs, colored with a white to blue gradient indicating increasing difference as blue intensity increases.


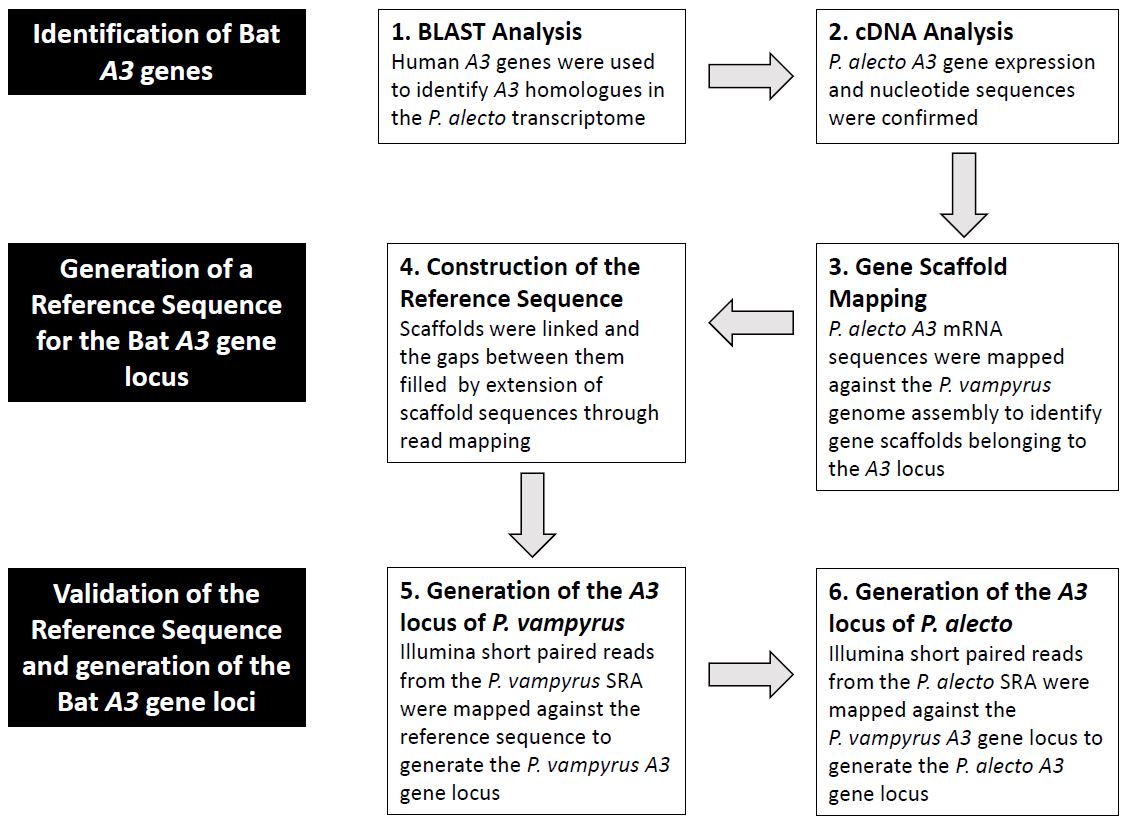


Fig. S2. The step-wise generation of the *Pteropus vampyrus* and *P. alecto* A3 gene loci. A3, APOBEC3; SRA, Sequence Read Archive.


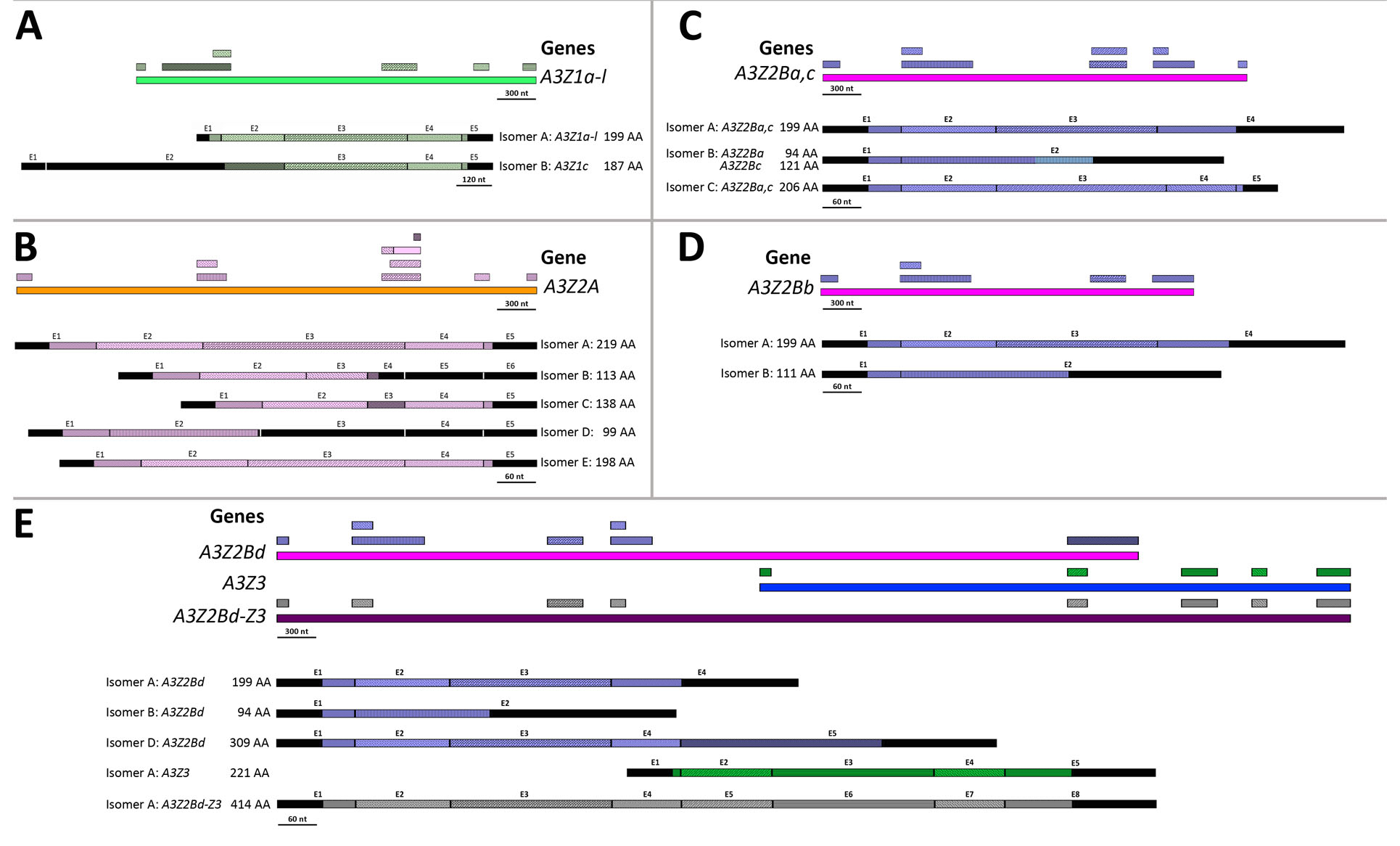


Fig. S3. Splicing schemes of *Pteropus alecto* *A3* genes. For each *A3* gene type, the known exons and resultant splicing schemes are depicted. For each splice isomer the exons are numbered and separated by vertical black and white lines. Colored segments represent the protein coding region of the mRNA, and black regions denote the 5' and 3' untranslated regions. Exons are numbered in order as E1, E2, E3, etc. and are patterned to delineate the genomic regions contributing to each splice isomer. A) The *A3Z1* splicing scheme is depicted. B) The splicing scheme of *A3Z2A*. C) The *A3Z2Ba* and *A3Z2Bc* genes have the same structure however the long form of the second exon contains a stop codon at different positions in each gene as a result of a frameshift mutation since the divergence of these genes. The light blue region in isomer B indicates the extended coding region of *A3Z2Bc* relative to the isomer B of *A3Z2Ba*. D) The *A3Z2Bb* gene lacks a fifth coding exon possessed by the other *A3Z2B* genes. E) The *A3Z2Bd* and *A3Z3* genes overlap. Each generates its own gene products and together produce the double Z-domain *A3Z2Bd-Z3*. The amino acid (AA) length of the encoded protein is provided for all isomers.


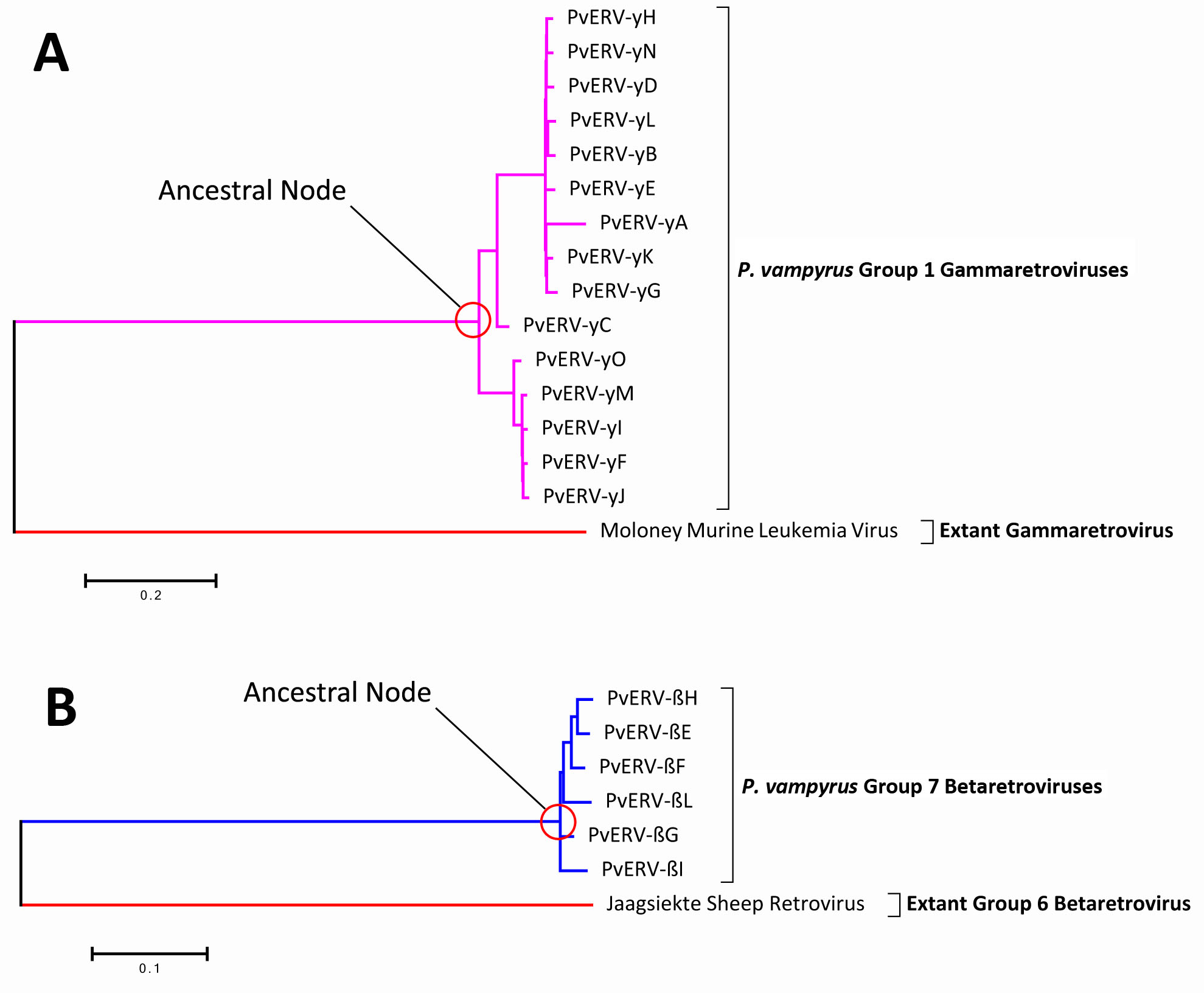


Fig. S4. Ancestral retroviral genome sequences were extrapolated from closely related groups of endogenous retroviruses. A) endogenous gammaretroviruses and B) endogenous betaretroviruses were extracted from the genome of *Pteropus vampyrus*. Their phylogenetic relationships were inferred using the maximum likelihood method and T92+G model. The scale represents the number of nucleotide substitutions per site. Retroviral genome sequences representing the ancestor at the indicated nodes were extrapolated for use in a subsequent hypermutation analysis.


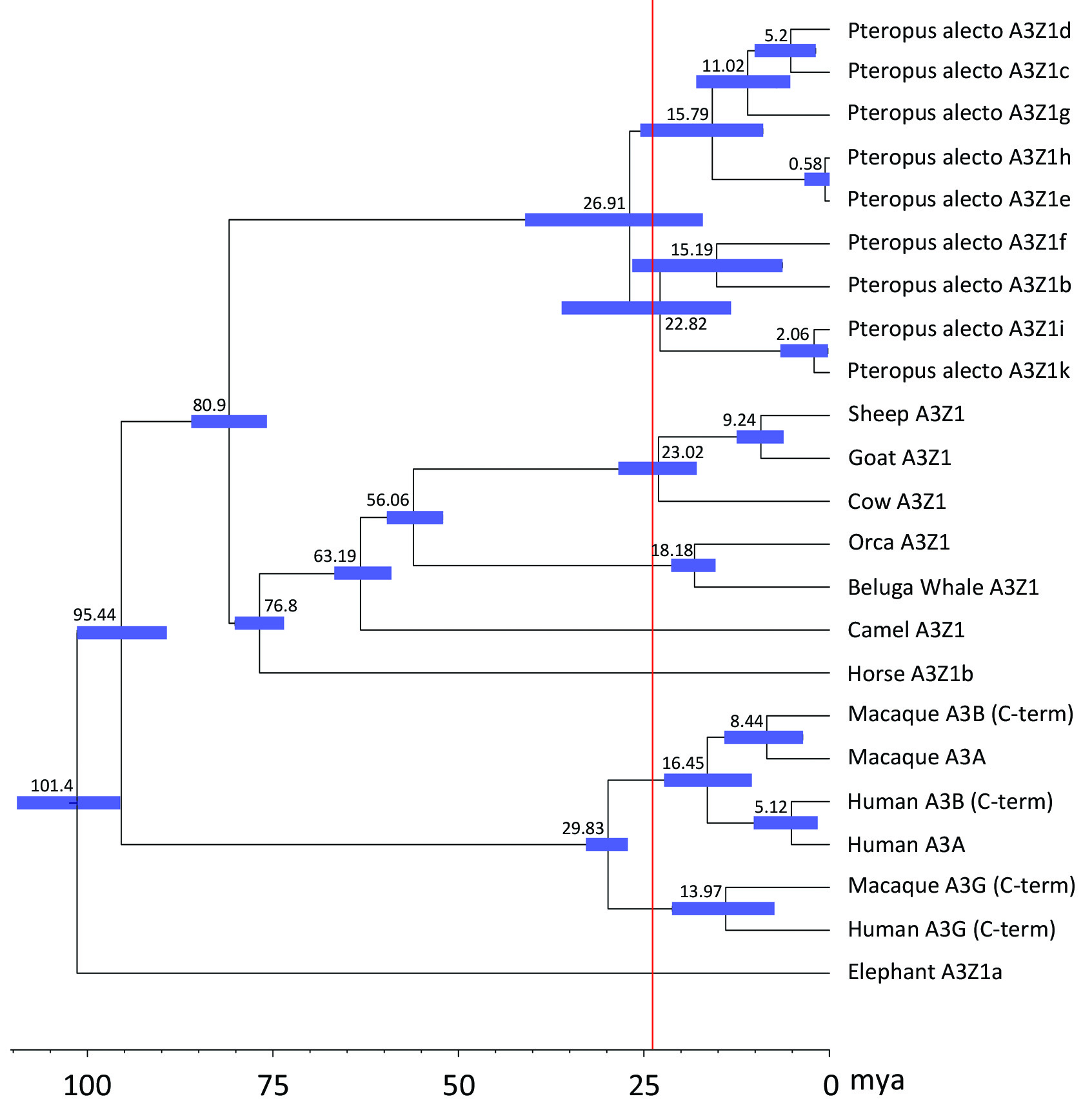


Fig. S5. Molecular clock estimations of mammalian *A3Z1* divergence times. Estimated times of divergence (in millions of years ago; mya) are indicated at the phylogenetic nodes, with the 95% confidence interval (CI) shown by the blue bars. . The estimated date of pteropid bat LINE-1 retroelement extinction of approximately 24 mya is shown by the vertical red line.
