# Supplementary material for: Differential Evolution of Antiretroviral Restriction Factors in Pteropid Bats as Revealed by APOBEC3 Gene Complexity
Source: Mol Biol Evol. 2018 Mar 29;35(7):1626–37. doi: 10.1093/molbev/msy048 (PMC5995163; doi:10.1093/molbev/msy048)
Supplement: Supplementary Data [file msy048_supp.zip › Supplementary Tables.docx]

Table S1. The presence of *A3* gene products in the cDNA library and transcriptome of *Pteropus alecto*.

|  |  |  |  |  |  |  |  |  |  |  |
| --- | --- | --- | --- | --- | --- | --- | --- | --- | --- | --- |
|  | **Pteropid A3 gene products** | | | |  |  | **Transcriptome analysis** | | |  |
|  |  |  | **Z-domain** | **ORF** | **Expression** |  | **Query** | **Number** | **Lowest** |  |
|  | **Gene** | **Isomer** | **subtype** | **length** | **verified ^†^** |  | **Sequence** | **of Hits** | **e-value** |  |
|  | *A3Z1a* | *A* | Z1 | 231 | No |  | Human A3A | 109 | 1.13x10^-41^ |  |
|  | *A3Z1b* | *A* | Z1 | 199 | No |  | Human A3C | 108 | 7.16x10^-55^ |  |
|  | *A3Z1c* | *A* | Z1 | 199 | Yes |  | Human A3H | 104 | 2.31x10^-55^ |  |
|  |  | *B* | Z1 | 187 | Yes |  |  |  |  |  |
|  | *A3Z1d* | *A* | Z1 | 199 | Yes |  | † *Pteropus alecto* A3 cDNA were mapped against the gene locus to identify the genes that expressed each cDNA product. A3 genes with homologous sequence structure are predicted to express homologous gene products and these are included in the list, differentiated by their expression-verification status. ORF, open reading frame. | | |  |
|  | *A3Z1e* | *A* | Z1 | 199 | No |  |  |  |  |  |
|  | *A3Z1f* | *A* | Z1 | 199 | Yes |  |  |  |  |  |
|  | *A3Z1g* | *A* | Z1 | 199 | No |  |  |  |  |  |
|  | *A3Z1h* | *A* | Z1 | 199 | No |  |  |  |  |  |
|  | *A3Z1i* | *A* | Z1 | 199 | Yes |  |  |  |  |  |
|  | *A3Z1j* | *A* | Z1 | 199 | Yes |  |  |  |  |  |
|  | *A3Z1k* | *A* | Z1 | 199 | Yes |  |  |  |  |  |
|  | *A3Z1l* | *A* | Z1 | 199 | Yes |  |  |  |  |  |
|  | *A3Z2A* | *A* | Z2/Z2A | 219 | Yes |  |  |  |  |  |
|  |  | *B* | Z2/Z2A | 113 | Yes |  |  |  |  |  |
|  |  | *C* | Z2/Z2A | 138 | Yes |  |  |  |  |  |
|  |  | *D* | Z2/Z2A | 99 | Yes |  |  |  |  |  |
|  |  | *E* | Z2/Z2A | 198 | Yes |  |  |  |  |  |
|  | *A3Z2Ba* | *A* | Z2B | 199 | No |  |  |  |  |  |
|  |  | *B* | Z2B | 94 | No |  |  |  |  |  |
|  |  | *C* | Z2B | 206 | Yes |  |  |  |  |  |
|  | *A3Z2Bb* | *A* | Z2B | 199 | Yes |  |  |  |  |  |
|  |  | *B* | Z2B | 111 | Yes |  |  |  |  |  |
|  | *A3Z2Bc* | *A* | Z2B | 199 | No |  |  |  |  |  |
|  |  | *B* | Z2B | 121 | Yes |  |  |  |  |  |
|  |  | *C* | Z2B | 206 | No |  |  |  |  |  |
|  | *A3Z2Bd* | *A* | Z2B | 199 | No |  |  |  |  |  |
|  |  | *B* | Z2B | 94 | No |  |  |  |  |  |
|  |  | *D* | Z2B | 309 | Yes |  |  |  |  |  |
|  | *A3Z3* | *A* | Z3 | 221 | Yes |  |  |  |  |  |
|  | *A3Z2Bd-Z3* | *A* | Z2B+Z3 | 414 | Yes |  |  |  |  |  |
|  |  | | | | |  |  |  |  |  |

Table S2. Accession numbers and genome locations

|  |  |  |  |  |  |  |
| --- | --- | --- | --- | --- | --- | --- |
|  | **Retrovirus** | |  |  |  |  |
|  |  | **Abbrieviation** | **GenBank Accession** | **Ensembl location** | **EMBL-EBI Nucleotide Archive** |  |
|  |  | **JSRV** | NC_001494.1 |  |  |  |
|  |  | **MLV** | NC_001501.1 |  |  |  |
|  |  |  |  |  |  |  |
|  | ***P. vampyrus* ERV (pteVam1 Release 67; http://asia.ensembl.org/Pteropus_vampyrus/Info/Index)** | | | |  |  |
|  |  | **PvERV-βE** |  | Scaffold 9648; 43764-35887 |  |  |
|  |  | **PvERV-βF** |  | Scaffold 17393; 20588-12812 |  |  |
|  |  | **PvERV-βG** |  | Scaffold 12793; 22017-29493 |  |  |
|  |  | **PvERV-βH** |  | GeneScaffold 1344; 357039-349197 |  |  |
|  |  | **PvERV-βI** |  | Scaffold 2273; 57766-7814 |  |  |
|  |  | **PvERV-βL** |  | Scaffold 12699; 17135-23787 |  |  |
|  |  | **PvERV-γA** |  | GeneScaffold_3363; 7385-14458 |  |  |
|  |  | **PvERV-γB** |  | scaffold_10119; 43308-50382 |  |  |
|  |  | **PvERV-γC** |  | scaffold_12163; 6846-14031 |  |  |
|  |  | **PvERV-γD** |  | scaffold_12630; 21480-28541 |  |  |
|  |  | **PvERV-γE** |  | scaffold_13050; 363-7426 |  |  |
|  |  | **PvERV-γF** |  | scaffold_14383; 9608-16822 |  |  |
|  |  | **PvERV-γG** |  | scaffold_1753; 121553-128630 |  |  |
|  |  | **PvERV-γH** |  | scaffold_2151; 113490-120546 |  |  |
|  |  | **PvERV-γI** |  | scaffold_22354; 327-7552 |  |  |
|  |  | **PvERV-γJ** |  | scaffold_22661; 960-8169 |  |  |
|  |  | **PvERV-γK** |  | scaffold_23277; 819-7847 |  |  |
|  |  | **PvERV-γL** |  | scaffold_24606; 118-7126 |  |  |
|  |  | **PvERV-γM** |  | scaffold_7076; 70738-77953 |  |  |
|  |  | **PvERV-γN** |  | scaffold_7237; 40439-47516 |  |  |
|  |  | **PvERV-γO** |  | scaffold_941; 109017-116205 |  |  |
|  |  |  |  |  |  |  |
|  | **Datasets** | |  |  |  |  |
|  |  | **Pteropus alecto transcriptome** | SRP008674 |  |  |  |
|  |  | **Pteropus alecto genome** | PRJNA171993 |  |  |  |
|  |  | **Pteropus vampyrus genome** | PRJNA20325 |  |  |  |
|  |  |  |  |  |  |  |
|  | **APOBEC3** | |  |  |  |  |
|  |  | **Goat A3Z1** | KM266657 |  |  |  |
|  |  | **Camel A3Z1** | XM_014551722 |  |  |  |
|  |  | **Beluga Whale A3Z1** | XM_022596460 |  |  |  |
|  |  | **Orca A3Z1** | XM_012536651 |  |  |  |
|  |  | **Sheep A3Z1** |  |  | EU864541 |  |
|  |  | **Sheep A3Z2** |  |  | EU864542 |  |
|  |  | **Sheep A3Z3** |  |  | EU864543 |  |
|  |  | **Cow A3Z1** |  |  | EU864534 |  |
|  |  | **Cow A3Z2** |  |  | EU864535 |  |
|  |  | **Cow A3Z3** |  |  | EU864536 |  |
|  |  | **Elephant A3Z1a** |  | ENSLAFT00000005276 |  |  |
|  |  | **Horse A3Z1b** |  |  | FJ532287 |  |
|  |  | **Horse A3Z2e** |  |  | FJ532288 |  |
|  |  | **Horse A3Z3** |  |  | FJ532289 |  |
|  |  | **Cat A3Z2a** |  |  | AY971954 |  |
|  |  | **Cat A3Z3** |  |  | EU011792 |  |
|  |  | **Pig A3Z2** |  |  | EU864539 |  |
|  |  | **Pig A3Z3** |  |  | EU864540 |  |
|  |  | **Mouse A3Z2-Z3** |  | ENSMUST00000109620 |  |  |
|  |  | **Macaque A3A** |  |  | JF714484 |  |
|  |  | **Macaque A3B** |  |  | JF714485 |  |
|  |  | **Macaque A3G** |  |  | AY331716 |  |
|  |  | **Human A3A** |  | ENST00000402255 |  |  |
|  |  | **Human A3B** |  | ENST00000333467 |  |  |
|  |  | **Human A3C** |  | ENST00000361441 |  |  |
|  |  | **Human A3G** |  | ENST00000407997 |  |  |
|  |  | **Human A3H** |  | ENST00000348946 |  |  |
|  |  | **Pteropus alecto A3Z1c Isomer A** | KX241551 |  |  |  |
|  |  | **Pteropus alecto A3Z1c Isomer B** | KX241552 |  |  |  |
|  |  | **Pteropus alecto A3Z1d** | KX241553 |  |  |  |
|  |  | **Pteropus alecto A3Z1f** | KX241554 |  |  |  |
|  |  | **Pteropus alecto A3Z1i** | KX241555 |  |  |  |
|  |  | **Pteropus alecto A3Z1j** | KX241556 |  |  |  |
|  |  | **Pteropus alecto A3Z1k** | KX241557 |  |  |  |
|  |  | **Pteropus alecto A3Z1l** | KX241558 |  |  |  |
|  |  | **Pteropus alecto Z2A Isomer A** | KX241559 |  |  |  |
|  |  | **Pteropus alecto Z2A Isomer B** | KX241560 |  |  |  |
|  |  | **Pteropus alecto Z2A Isomer C** | KX241561 |  |  |  |
|  |  | **Pteropus alecto Z2A Isomer D** | KX241562 |  |  |  |
|  |  | **Pteropus alecto Z2A Isomer E** | KX241563 |  |  |  |
|  |  | **Pteropus alecto Z2Ba Isomer C** | KX241564 |  |  |  |
|  |  | **Pteropus alecto Z2Bb Isomer A** | KX241565 |  |  |  |
|  |  | **Pteropus alecto Z2Bb Isomer B** | KX241566 |  |  |  |
|  |  | **Pteropus alecto Z2Bc Isomer B** | KX241567 |  |  |  |
|  |  | **Pteropus alecto Z2Bd Isomer D** | KX241568 |  |  |  |
|  |  | **Pteropus alecto A3Z2Bd-Z3** | KX241569 |  |  |  |
|  |  | **Pteropus alecto A3Z3** | KX241570 |  |  |  |
|  |  |  |  |  |  |  |
|  | **Loci** | |  |  |  |  |
|  |  | **Pteropus vampyrus A3 Locus** | KX241572 |  |  |  |
|  |  | **Pteropus alecto A3 locus** | KX241571 |  |  |  |
|  |  |  |  |  |  |  |
